# Biogeography of soda lake microbiome and uneven cross-continent transition rates

**DOI:** 10.3389/fmicb.2025.1614302

**Published:** 2025-07-24

**Authors:** Minglei Ren, Jianjun Wang

**Affiliations:** State Key Laboratory of Lake and Watershed Science for Water Security, Nanjing Institute of Geography and Limnology, Chinese Academy of Sciences, Nanjing, China

**Keywords:** soda lakes, microbial biogeography, geographical endemism, habitat transition, metagenome

## Abstract

Microbial dark matter in soda lakes has been increasingly illuminated, however, much remains unknown about microbial biogeography at the global scale and underlying mechanisms. To study microbial biogeography and dispersal patterns, we analyzed 51 soda lake metagenomes collected from key global regions, including 37 from the Kulunda Steppe in South Siberia, Mongolia, and the Cariboo Plateau in Canada, as well as 14 newly sequenced samples from the East African Rift Valley. We found that there were 575 widespread taxa such as the dominant archaeal Haloarchaeota and actinobacterial *Nitriliruptor* persistently inhabiting global soda lakes. We further identified 1,217 region-specific taxa, with Africa containing the highest proportion of geographical endemism (66.72%). Such effects of dispersal limitation on microbial assembly of global soda lakes were supported by the significant distance-decay relationships for taxonomic and functional composition, and genomic similarity. For example, microbial genomic divergence was closely associated with their geographical distance, showing that both inter- and intraspecies genome similarities decayed with distance. This concurs with the uneven dispersal history among continental microbiomes, indicated by the at least one order of magnitude lower transition rates between Africa and other continents than between Asia and North America. Our results revealed that the global biogeography of soda lake microbial communities across three continents and their distinct transition history between continents. These findings highlight the critical role of microbial evolutionary history associated with dispersal limitation in shaping their geographical distribution in extreme environments.

## 1 Introduction

Soda lakes, also known as alkaline lakes, are characterized by high pH (9.0–12.0) and large amounts of soda, typically sodium carbonate (Grant et al., 1990; Jones et al., 1998). They are widely distributed across the globe, including China, North America, Russia, and the East African Rift Valley (Sultanpuram and Mothe, 2019). As one of the most productive ecosystems, soda lakes have high productivity rates of 4,000–6,000 g O_2_ m^−2^ per day when compared to other aquatic ecosystems (800–2000 g for rivers and lakes) (Schagerl and Burian, 2016), owing to the dominance of microphytes fueled by high carbonate in the ecosystem (Grant and Jones, 2016). Such geochemistry supports the growth of a large number of microorganisms, which play an important role in the elemental cycling of the ecosystem (Antony Paul et al., 2013). Microbial taxonomic and functional composition of soda lakes has been greatly revealed through traditional cultivation and high-throughput sequencing. For example, the insightful understanding of physiological characteristics and their hyperalkaline adaptation mechanisms benefit from strain isolation in the lab (Sorokin et al., 2022), and a high-resolution genetic inventory and metabolic capacity of prokaryotic communities are illustrated by high-throughput omics techniques (Vavourakis et al., 2016; Zorz et al., 2019; Zhao et al., 2020). However, the biogeography of soda lake microorganisms at a global scale and their evolutionary history remain understudied.

The similarities in microbial profiles among soda lakes across geographic regions are increasingly observed. For example, diverse lineages within Bacteria and Archaea, such as phototrophs and sulfur oxidizers, are commonly detected in soda lakes across Africa, North America and Eurasia (Antony Paul et al., 2013), and core microbiomes are identified in soda lakes distributed in Asia and Canada across 8,000 km (Zorz et al., 2019). The microbial compositional similarity patterns in regional studies raise an important question about the evolution of microbiomes in global soda lakes. That is whether a core microbiome is shared among soda lakes worldwide, or whether the microbes in regional lakes evolved independently under the influence of geochemical factors. Alkaline lakes are proposed to have existed throughout the geological record of Earth (Jones et al., 1998; Haas et al., 2024), likely predating the late Archean continents 2.72 billion years ago according to nitrogen isotope evidence (Stüeken et al., 2015). Therefore, ancient history provides an opportunity to answer these questions about the evolutionary origin of soda lake microbial lineages.

Here, we compiled 51 metagenomic sediment and water samples from soda lakes across three continents to comprehensively evaluate microbial diversity and their global biogeographical patterns. We related the variation in microbial taxonomic and functional composition, as well as inter- and intra-genomic similarities, to the increasing geographical distance of soda lakes. We further modeled the inter-continent transition for microbial communities based on the phylogeny inferred from 1,330 species-level genomes. We aimed to answer three questions: (i) Is there a core microbiome shared by soda lakes across three continents? (ii) How does dispersal limitation shape microbiomes in terms of taxonomic and functional composition and genome divergence? (iii) How does their evolutionary history (i.e., the cross-continent transition) contribute to the biogeography? Our results revealed the global biogeography of soda lake microbial communities across three continents, and that their distinct transition history between continents plays an important role in shaping microbial diversity and its biogeographical patterns.

## 2 Materials and method

### 2.1 Sampling and sequencing

Surface water and sediment samples were collected from seven alkaline lakes in East Africa during February 2020, with pH values ranging from 9 to 10.1 (Supplementary Table S1). Details about the description of the lakes and the collection of samples were provided previously (Ren et al., 2024). The total DNA of each sample was extracted using the PowerSoil DNA Isolation Kit (QIAGEN, Germany) under sterile conditions. For the sediment, about 0.4 g of dried soil was used for DNA extraction, whereas the microorganisms in water samples were enriched using filtering membranes (0.22 μm, Millipore Sigma, USA). The DNA was then subject to metagenomic sequencing according to the manufacturing protocol as follows. Genomic DNA was first fragmented into segments ranging from 250 bp to 350 bp using ultrasonication. The libraries were prepared using the NEB Next Ultra DNA Library Prep Kit and sequenced on the Illumina NovaSeq 6,000 platform using a 2*150 bp paired-end sequencing strategy.

### 2.2 Global soda lake metagenome

**Figure 1:**
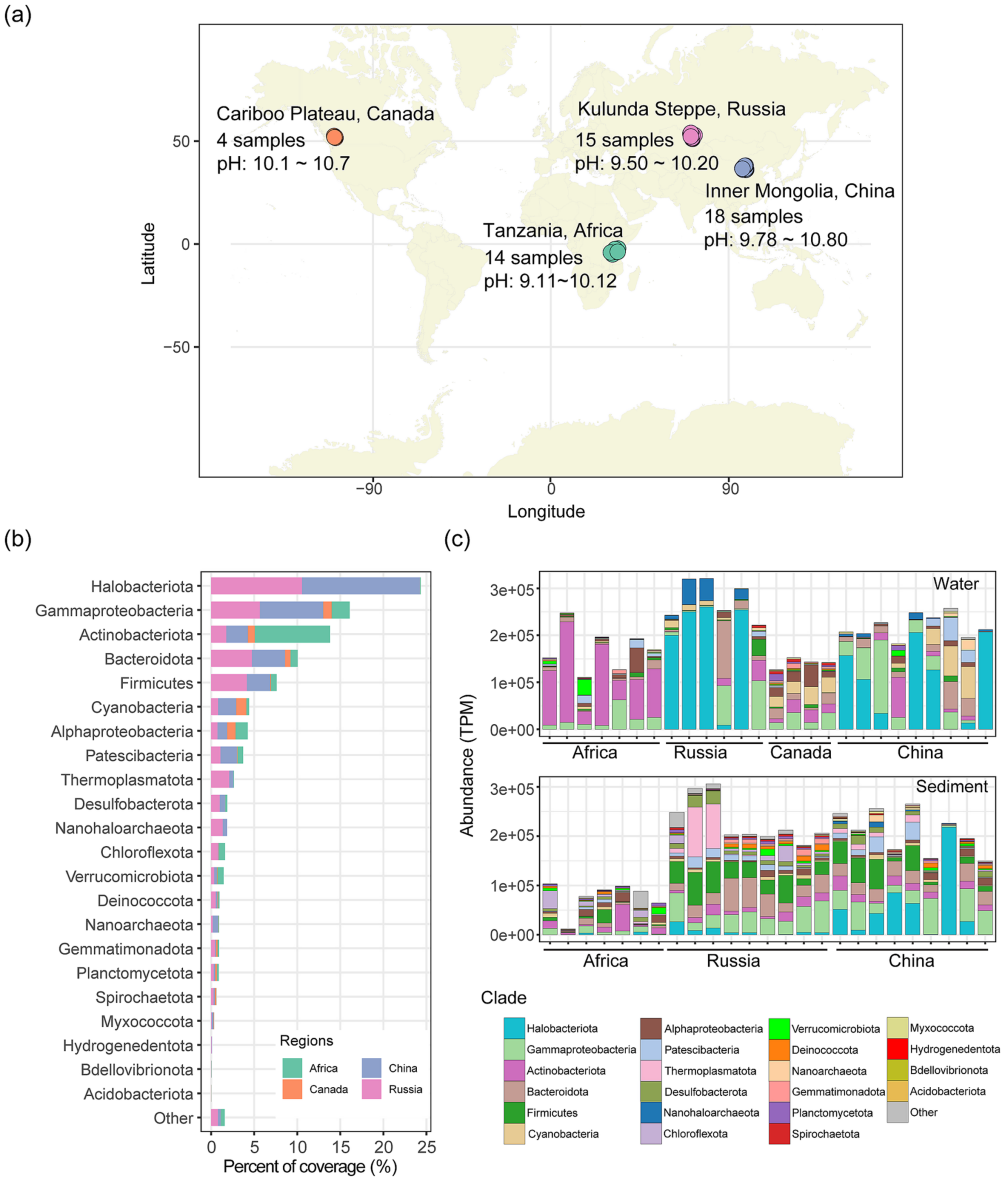
The geographic distribution of soda lakes and microbial composition. **(a)** The geographic location of soda lake metagenomes used in the study, including the new samples collected from East Africa. Soda lakes from four geographic regions, namely Africa, China, Canada and Russia, were highlighted with distinct colors. The detailed accession numbers for these metagenomes were shown in Supplementary Table S1. **(b)** The average total coverage percentage of microbial species at the phylum level across four regions. **(c)** The abundance distribution of microbial species at the phylum level across water and sediment samples from four regions. The calculation of microbial species abundance was shown in the Methods section, and the relative abundance was calculated as transcripts per million (TPM), a normalized unit widely used in metagenome read recruitment approaches. The group ‘Other’ in **(b,c)** includes the phyla with fewer than five species.

To comprehensively evaluate global soda lake microbial diversity and metabolic potential, 37 metagenomic samples from global soda lakes were collected from NCBI SRA and IMG databases (Figure 1 and Supplementary Table S1), including six water and nine sediment samples from soda lakes in the Kulunda Steppe, South-Western Siberia (Vavourakis et al., 2016; Vavourakis et al., 2018; Vavourakis et al., 2019), four water samples from soda lakes in the Cariboo Plateau region of British Columbia, Canada (Zorz et al., 2019), and nine water and nine sediment samples from soda lakes in the southwest of Inner Mongolia Autonomous Region, China (Zhao et al., 2020). The details about the accession number and physiochemical parameters for these samples were shown in the Supplementary Table S1.

### 2.3 Taxonomic profiling of prokaryotes

Taxonomic profiling of the prokaryotic community was performed based on the conserved ribosomal protein *rpS3* genes through a modified pipeline described previously (Diamond et al., 2019). Briefly, all prokaryotic *rpS3* genes were first identified from metagenomic assembly with hmmsearch v3.2.1 (Eddy, 2011) using a custom Hidden Markov Model (HMM) database (Diamond et al., 2019) and clustered at 99% similarity with USEARCH v11.0.667 (Edgar, 2010), with each *rpS3* cluster representing a species. To quantify their relative abundance across samples, the longest contigs containing *rpS3* were selected as the representative sequence for each species. Clean reads were aligned against these representative sequences using Bowtie2 v2.3.5.1 (Langmead and Salzberg, 2012), and the mapped reads with ≥ 99% identity were filtered and counted using the ‘depth’ module of Samtools v1.15.1 (Li et al., 2009). The final abundance of a species in a sample was calculated as the total mapped bases normalized by the length of the representative sequence and the total number of sequencing bases in the sample.

Species taxonomy was determined using the sequence alignment approach followed by phylogeny inference. Firstly, the query *rpS3* genes were aligned against a custom *rpS3* reference database using BlastP with e-value ≤ 1e-3 and identity ≥ 50%, where the *rpS3* database was retrieved from the RefSeq prokaryotic genome database (~27,000 genomes, downloading date: 2019–07). The taxonomy of the top hit in the database was assigned to the query *rpS3* species. Secondly, to validate and correct the alignment-based results, the *rpS3* gene tree was constructed as described previously (Ren et al., 2019; Ren and Wang, 2022). The representative as well as the reference *rpS3* genes were aligned using MAFFT v7.427 (Katoh et al., 2005), and trimmed using trimAl v1.4.1 with the ‘-automated1’ option (Capella-Gutiérrez et al., 2009). An approximately maximum-likelihood tree was built by FastTree v2.1.11 (Price et al., 2010). The taxonomies of species that had no hits in the reference database and branched deeply in microbial lineages in the *rpS3* tree were designated as the ‘Unassigned’ group.

### 2.4 Function profiling of prokaryotic communities across soda lakes

To obtain the normalized abundance of functional genes across samples with uneven sequencing depth, 10 million clean reads were randomly retrieved from each sample using the ‘sample’ module of Seqtk 1.4-r122 (Li, 2013). These reads were then functionally annotated using SUPER-FOCUS v1.7 (Silva et al., 2015), which efficiently aligns short sequences against the protein-coding genes clustered at 98% identity in the SEED database using MMseqs2 v14-7e284 as a search engine (Steinegger and Söding, 2017). The SEED subsystem classifies the functional genes into three levels of biological pathways with similar functions (Overbeek et al., 2005). The functional annotation results were used to evaluate the functional composition of communities across soda lakes (see the ‘Statistical analysis’ section below).

### 2.5 Metagenome assembly and binning

All soda lake metagenomic reads were processed using the custom pipeline as described previously (Ren and Wang, 2022). Briefly, the read quality was checked using FastQC v0.11.8 (Andrews, 2010), then trimmed using Trimmomatic v0.39 (Bolger et al., 2014), discarding reads with an average Phred score lower than 25 using a 4-bp-wide sliding window and reads shorter than 50 bp. Clean reads were assembled individually using MEGAHIT v1.2.8 (Li et al., 2015), with the parameter ‘--presets meta-large --min-contig-len 1000’. The metagenome-assembled genomes (MAGs) were reconstructed by DAS Tool v1.1.1 (Sieber et al., 2018), which determines optimized MAGs through a strategy of dereplicating, aggregating and scoring the preliminary MAGs from multiple binning algorithms, including MaxBin2 v2.2.6 (Wu et al., 2016), MetaBat1 v0.24.1 (Kang et al., 2015), MetaBat2 v2.12.1 (Kang et al., 2019) and CONCOCT v1.1.0 (Alneberg et al., 2014). The MAG’s completeness and contamination were evaluated using CheckM v1.0.13 (Parks et al., 2015). A total of redundant 2,227 genomes with the completeness ≥ 70% and the contamination ≤ 10% were subject to downstream analyses (Supplementary Table S3). Taxonomic assignment for these MAGs was performed by the “classify_wf” module of GTDB-Tk v2.0.0 (Chaumeil et al., 2019) using the r207 version of the Genome Taxonomy Database database (GTDB).

The genomes for the representative species were further identified as described previously (Diamond et al., 2019; Ren et al., 2024). In total, 1,330 representative genomes were determined from global soda lakes based the *rpS3*-containing contig sequences shared between the redundant MAGs and the *rpS3*-based representative species (Supplementary Table S3). Note that not all species genomes were recovered using metagenome binning methods partly due to the great microbial diversity in environmental samples and limitation of computational methods (Quince et al., 2017). Detailed statistics about the clean reads and assembly for each sample were shown in Supplementary Table S1, along with the MAGs in Supplementary Table S3.

### 2.6 Function annotation of the representative MAGs

The protein-coding genes of each genome were predicted by Prodigal v2.6.3 (Hyatt et al., 2010) and annotated against the Kyoto Encyclopedia of Genes and Genomes database (KEGG, release 92) and the eggNOG database v5.0 (Huerta-Cepas et al., 2018). For each gene, the KEGG Orthology (KOs) assignment was achieved using KOFamScan v1.3.0 (Aramaki et al., 2019), which performs homology searches against a database of hidden Markov models with precomputed score thresholds for each KOs. The annotation against eggNOG was performed using eggNOG mapper v2.1.6 (Cantalapiedra et al., 2021), with DIAMOND v2.1.8 (Buchfink et al., 2015) as a search engine.

### 2.7 Phylogenomic tree construction of the representative species

The maximum likelihood phylogeny of the species-level MAGs was constructed using the concatenation of the conserved marker genes as described previously (Ren et al., 2019). Specifically, the HMM profiles for the conserved marker genes used in CheckM v1.0.13 were extracted, and then searched against each MAG using hmmsearch v3.2.1 (Eddy, 2011) with sequence e-value of ‘1e-35’. The HMM coverage lower than 0.35 was discarded. If the same region of the sequence was hit by more than one HMM, the hit having the lowest e-value was kept. For each gene family, the amino acid sequences of gene members were extracted from each genome, independently aligned using MAFFT v7.427 (Katoh et al., 2005), and trimmed by trimAl v1.4.1 (Capella-Gutiérrez et al., 2009) with the ‘automated1’ option. The alignments were then concatenated together, on which the maximum likelihood phylogenomic tree was built using IQTREE v1.6.11 (Nguyen et al., 2015) with the optimal substitution model for each gene family determined by ModelFinder (Kalyaanamoorthy et al., 2017) among the four models: WAG, LG, JTT and JTTCDMut. The phylogenetic tree was constructed with the edge-linked partition model and 1,000 replicates using an ultrafast bootstrap approximation. Unless stated explicitly, the default parameters were used in all programs mentioned above.

### 2.8 The inference of evolutionary transition using BayesTraits

We estimated microbial transition rates across continents using the ancestral state reconstructions through the Markov chain Monte Carlo (MCMC) approach implemented in BayesTraits v4.0.0 (Pagel et al., 2004). The BayesTraits analyses were performed on the full phylogenetic tree using the MultiState module, which is applied to traits with two or more discrete states, such as three continents where soda lakes are distributed in the study. As suggested in the program manual, the tree was scaled to have a mean branch length of 0.1 to avoid very small rates in results.

Firstly, we performed model tests by constraining the forward and reverse transition rates among continents to be equal (i.e., *q_AB_* = *q_BA_*, from continent A to continent B and vice versa), or separately constraining each of the rates to be zero (*q_AB_* = 0 or *q_BA_* = 0). These constrained models were compared to select the best-fit model based on the log-Bayes factors in MCMC analyses, with a difference of 10 log marginal likelihood units as very strong evidence for a model over another. The marginal likelihood of MCMC analyses was estimated by the stepping stone sampler method using 100 stones and 1,000 iterations per stone (Xie et al., 2010). Additionally, we also compared several prior distributions for transition rates, including uniform, exponential, gamma and their hyper-prior versions, and found that the gamma hyper-prior distribution was the most optimal and therefore used in further analyses. All preliminary analyses were conducted with the following settings: 10,100,000 iterations, 100,000 iterations as burn-in and sampling every 1,000 iterations. The final MCMC analyses were repeated three times to check the congruence of independent runs.

The phylogenetic construction and transition inference for soda lake microbiomes considers all species-level genomes and their occurrence patterns, which were not biased by uneven number of samples from different continents. For example, the phylogenetic tree was built based on the species-level genomes, which were clustered from all microbial genomes reconstructed from soda lakes all over the world, rather than a single continent. Before modeling the transition rates using BayesTrait, the state of each species could be a single continent, or more than ones based on based on the relative abundance around three continents.

### 2.9 Statistical analyses

Two types of microbial taxonomic profiling datasets were explored in the study: the 3,526 *rpS3*-based species table and the 1,330 species-level representative genome table. The *rpS3*-based species abundance table was used to calculate the relative abundance of microbial species, community composition and identification of core/flexible species. The functional profiling table generated by SuperFocus (see above) was used to evaluate functional composition across global soda lakes. Taxonomic and functional composition of microbial communities were evaluated using nonmetric multidimensional scaling (NMDS) using Bray-Curtis and Euclidean distance, respectively, as implemented in the ‘metaMDS’ function in the VEGAN package v2.6–4. The distance-decay relationships between Bray–Curtis dissimilarity of microbial community and geographic distance were fitted using the linear regression model.

Genomic similarity was evaluated by the genome-wide average nucleotide identity (ANI) at both the species and strain levels, which was calculated using fastANI v1.34 (Jain et al., 2018). To access genomic similarity across geographic regions, the pairwise genome-wide ANI of the 1,330 representative genomes were used for the species-level similarity, and the genome pairs with genome-wide ANI ≥ 95% were used for the strain-level similarity.

The core and flexible species and genes were further evaluated by their occurrences in soda lake samples across each of the four geographical regions. For the species, the 3,526 *rpS3*-based species table was used to evaluate their occurrence patterns. For the genes, the KO-based functional annotation results of 2,227 redundant genomes were used. First, a set of 1,015 singleton KOs (present in only one MAG) was discarded to avoid potential bias associated with genome assembly and functional annotation. Therefore, there were 8,645 KOs present in at least two genomes, representing functional composition of global soda lake microbial communities.

To measure the geographic distribution of a species across global soda lakes, an index of species range size was calculated as one minus the standard deviation of the abundance percentage of the species across geographical regions. The relationships between species range size and their genome size were fitted using a linear regression model. All statistical analyses were performed using R language v4.2.2.

## 3 Results and discussion

### 3.1 A vast uncultured microbial diversity across global soda lakes

We collected 51 metagenomes of soda lakes, including 14 newly sequenced ones from the East African Rift Valley in this study, and the remaining 37 from Canada, China, and Russia (Figure 1a and Supplementary Table S1). These samples were characterized by high pH values ranging from 9.1 to 11.0, and salinity concentrations ranging from 5.5‰ to 8,532‰. We performed taxonomic profiling of prokaryotic community based on the clustering of the conserved ribosomal protein S3 gene (*rpS3*) from metagenomic assemblies (See the details in Methods), considering the fact that the majority of microorganisms with low abundance are poorly represented by the metagenomic-assembled genomes (Diamond et al., 2019). In total, there were 3,066 bacterial and 438 archaeal species defined by the assembled *rpS3* genes from soda lakes across the four geographic regions. We detected 74 phylum-level lineages across soda lakes, with more than half phyla (*n* = 40, ~54.0%) represented by no more than five species, according to the Genome Taxonomy Database (GTDB) classification framework (Parks et al., 2021). Among these phyla, the top four with the highest species number included Bacteroidota (*n* = 445), Gammaproteobacteria (408), Actinobacteroita (388) and Firmicutes (352) within the Bacteria, and Halobacteriota (217), Nanoarchaeota (118), Thermoplasmatota (55) and Nanohaloarchaeota (24) within the Archaea (Supplementary Figure S1a).

In agreement with species numbers, the relative abundance of these bacterial and archaeal phyla dominated microbial community of global soda lakes (Figures 1b,c). For example, the top three most abundant phyla, including Halobacteriota (24.3%), Gammaproteobacteria (16.1%) and Actinobacteriota (13.8%), accounted for more than half of the total abundance according to the metagenomic read recruitment approach. Moreover, these phyla showed variation in their abundance across geographical regions (Figure 1b). For example, the microbial planktonic community of East African soda lakes was dominated by Actinobacteriota with its average abundance of 58.9% (SD, 25.8%). In the soda lakes of China and Russia, the most abundant phylum was Halobacteriota, with average abundances of 55.1% (SD, 41.3%) and 42.6% (SD, 38.1%), respectively. The identification of these dominant bacterial phyla here well explains the fact that more than 60% of culturable strains isolated from soda lakes belong to the phyla Gammaproteobacteria and Firmicutes (Supplementary Figure S1b, Supplementary Table S2). In addition, the large number of microbial species identified through metagenomic survey demonstrates a high proportion of uncultured species in soda lakes, consistent with the previous argument about the uncultured microbes across biomes (Steen et al., 2019). The observed difference in the number of species between metagenomic survey and experimental culturing also provides a candidate species list and potential cultivation strategies for strain isolation in soda lakes (Lewis et al., 2021).

### 3.2 The core taxa and functional genes across global soda lakes

**Figure 2:**
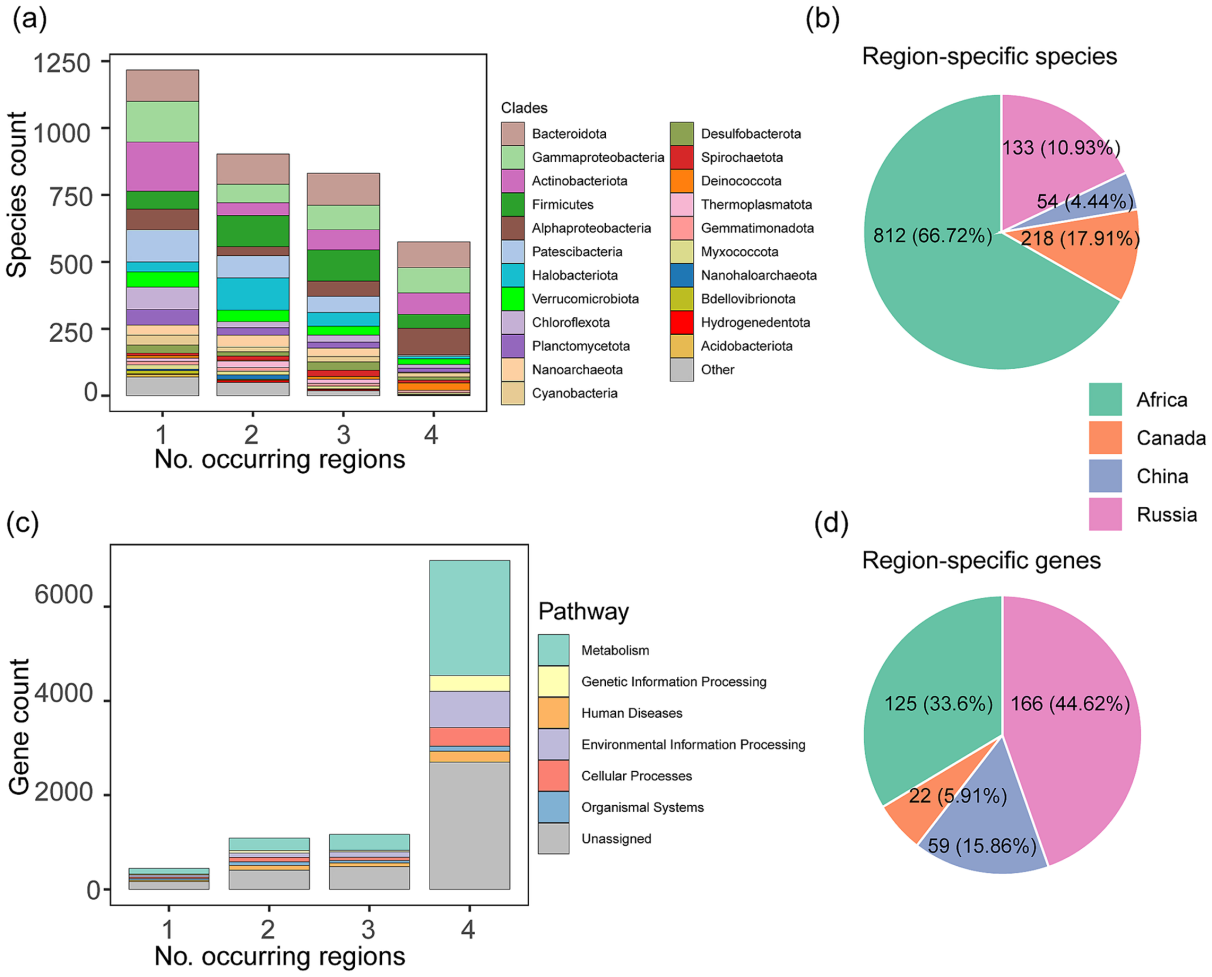
The occurrence distribution of microbial species and genes in soda lakes. **(a)** The count of microbial species occurring in different geographic regions. The phyla were highlighted with distinct colors, consistent with the color scheme in Figure 1b. **(b)** The geographic distribution of the endemic species, i.e., those restricted in one region. **(c)** The count of KO-represented functional genes occurring in different regions. The functional categories of these genes followed the first level pathway system of the KEGG and were highlighted with distinct colors. **(d)** The geographic distribution of endemic KOs.

A core microbiome was shared among soda lakes across four geographical regions, indicative of their strong dispersal abilities. Specifically, there were 575 species (16.30% of the total species) present in at least one sample of each region, with the dominant archaeal Haloarchaeota and actinobacterial Nitriliruptor as examples (Figure 2a). These widespread species showed a wide range of taxonomic distribution, with four phyla dominating the community, including Alphaproteobacteria (*n* = 100), Bacteriodota (96), Gammaproteobacteria (95), Actinobacteriota (80). In contrast, more than twice as many species, namely 1,217 (34.50%), were present in only one of the four regions. Among these regional-specific or endemic species, more than two-thirds (*n* = 812, 66.72%) were detected from the African region (Figure 2b), indicating the great unexplored microbial diversity in the soda lake ecosystem on the second-largest continent of the Earth. Our findings extend the results of a previous study focusing on the comparison of soda lakes in Canada and Russia (Zorz et al., 2019), and demonstrate that the core microbiome of soda lakes has likely assembled at a global scale. The occurrence of the core microbiome is likely associated with the efficient dispersal for microbial communities (Hanson et al., 2012), and implies a scenario of their common and ancient evolutionary origin for the soda lake communities, rather than independent origins.

The distribution of microbial functional genes showed contrast patterns across four geographical regions compared to the species distribution outlined earlier. For example, most functional genes (6,344 KOs, 73.40% of the total gene families) were shared across all four regions, whereas only a minor number of genes (372, 4.30%) were present in one region, referred to as the region-specific genes (Figures 2c,d). The predominance of the shared genes confirmed the relatively stable functional composition across soda lakes at a global scale shown as below. For regional-specific genes, microbes in soda lakes from Russia accounted for half of the total (44.62%), and these genes were mainly involved in the KEGG functional categories of “Metabolism” (123) and “Environmental Information Processing” (45). These region-specific genes are likely used to cope with the specific substrate resources or stress present in one of these geographical regions.

### 3.3 Taxonomic, functional and genomic biogeography of soda lake microbiome

**Figure 3:**
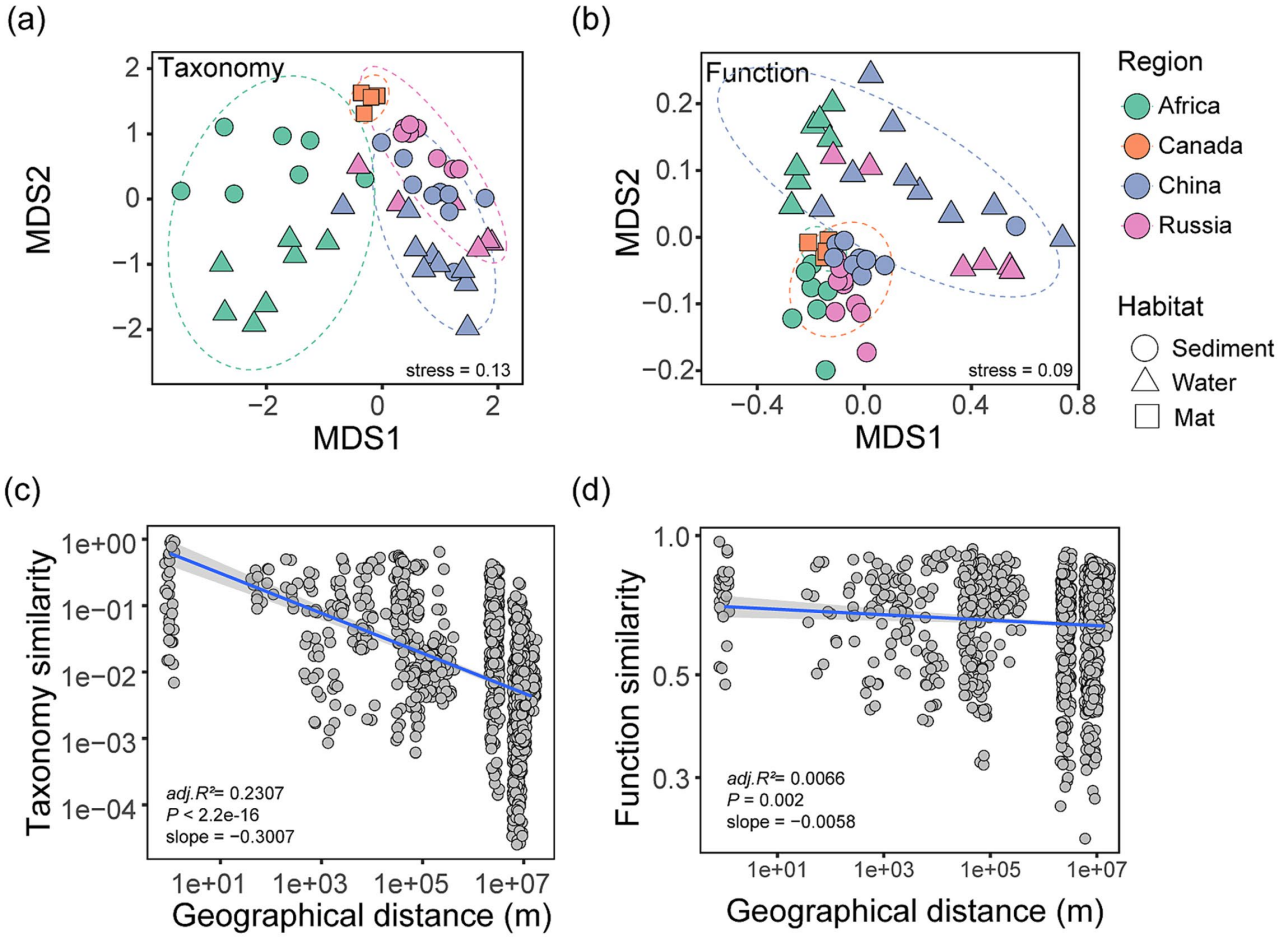
Taxonomic and functional biogeography in global soda lakes. **(a,b)** Nonmetric multidimensional scaling (NMDS) ordination plot of soda lake microbial taxonomic and functional composition across four geographic regions. **(c,d)** The distance-decay relationship (DDR) between geographical distance and microbial community similarity and functional composition similarity, respectively. Microbial community similarity was represented by Bray-Curtis distance of the *rpS3*-represented abundance table, and the similarity in functional composition was represented by Euclidean distance of the SEED-based function across all samples (see the Methods). The DDRs were fitted using linear regression model, and the adjusted coefficients of regression models were shown at the subpanels, where both X and Y axes were log10-transformed.

Despite core taxa and genes, we found that dispersal limitation consistently shapes the biogeography of soda lake microbial communities in terms of taxonomic and functional composition, as well as genome divergence. The taxonomic biogeography was supported by two lines of evidence (Figure 3). Firstly, the non-metric multidimensional scaling analysis (NMDS) revealed that microbial communities were clustered by geographic regions regardless of their habitats (NMDS stress = 0.13; PERMANOVA *R^2^* = 0.17, *p* = 0.001, 999 permutations, Figure 3a), indicating a higher similarity of within-region microbial community than between regions. Secondly, the similarity of microbial community composition decayed with the geographical distance between soda lakes (Figure. 3c, *P* < 2.2e-16, *R^2^_adj_* = 0.23), suggesting a distance-decay relationship (DDR), a fundamental pattern in ecology (Zhou and Ning, 2017). These results demonstrate that microbial communities in these extreme environments also follow the macroecology patterns.

Microbial functional composition of soda lakes showed consistent distance-decay relationships at a global scale. Specifically, functional composition was represented by functional genes curated in SEED annotation system (Overbeek et al., 2005). The global soda lake functional composition was structured into distinct clusters, in line with their geographical regions (NMDS stress = 0.09; PERMANOVA *R^2^* = 0.22, *p* = 0.001, Figure 3b). In addition, there was functional difference in microbial communities between water and sediments, consistent with their distinct taxonomic composition. Similarly, we also observed a decay of microbial function similarity with increasing geographical distance, indicating functional biogeography of soda microbial communities (Figures 3d, *P* = 2.06e-3, *R^2^_adj_* = 0.01). Furthermore, we found that the decreasing rate of functional DDR (slope = 0.01) was less than taxonomic DDR across soda lakes (slope = 0.30, Figures 3c,d), indicating functional composition similarity decay slower than taxonomic at similar geographical distances. These results demonstrated a relatively higher spatial turnover rate of taxonomic composition and a stable functional composition for global soda lake communities. In contrast, a recent study of marine bacterial functional biogeography shows a higher turnover rate of functional profiles than taxonomic profiles in Southern and Atlantic Ocean (Dlugosch et al., 2022). These distinct biogeography patterns may be attributed to the differences in microbial dispersal ability, spatial scales and sampling efforts between these studies (Meyer et al., 2018).

**Figure 4:**
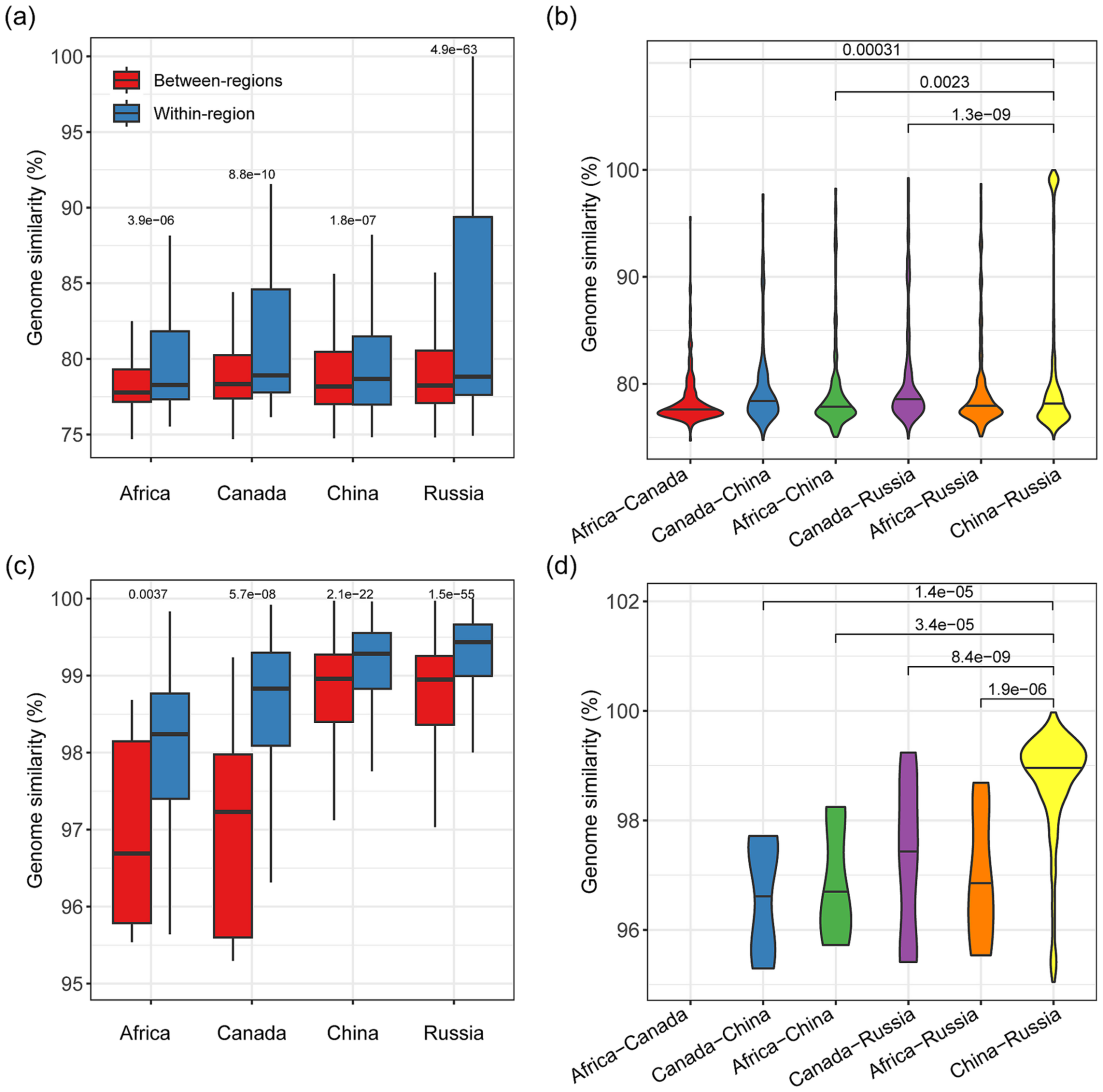
Microbial genomic similarities of soda lakes across geographic regions. **(a)** The comparison of between-region and within-region genomic similarities at the species level. Genomic similarity was measured as the genome-wide average nucleotide identity (ANI). For each of the four regions, the difference in between-region and within-region similarity was tested using the Wilcoxon test. **(b)** The distribution of the between-region genomic similarities at the species level. **(c)** The comparison of the between-region and within-region genomic similarities at the strain level, namely, specifically including genome pairs with ANI values greater than 95%. **(d)** The distribution of the between-region genomic similarity at the strain level. The region pairs in the subpanels **(b,d)** were arranged by their geographical distance, with the longest ‘Africa-Canada’ on the leftmost and the shortest ‘China-Russia’ on the rightmost. Noted that there were no strain-level genome pairs in the “Africa-Canada” pair owing to the low number of strain-level genomes reconstructed from Canada soda lakes in the study.

The divergence of soda lake microbial genomes at the species and strain levels was closely associated with their geographical distance. There were 2,227 non-redundant metagenome-assembled genomes (MAGs) initially reconstructed from lakes across four regions, with 381 in Africa, 629 in Asia, 989 in Russia, and 228 in Canada (Supplementary Table S3). Then 1,330 species-level genomes were determined based on the occurrence of contigs containing the representative *rpS3* cluster for each species (See the Methods). Overall, we found that microbial genomic similarity, represented by genomic ANI, decreases with geographic distance (Figure 4). Specifically, for each of the four regions, soda lake microbial genomes consistently showed a significantly higher within-region similarity than between-region based on the species-level genomes (*n* = 1,330, *p* = 3.9e-6 ~ 4.9e-63, Wilcoxon test, Figure 4a) and the strain-level genomes (*n* = 2,227, *p* = 3.7e-3 ~ 1.5e-55, Wilcoxon test, Figure 4b). These improved within-region genome similarities indicate relatively lower genomic divergence in soda lakes within region than between regions. The genomic similarity patterns associated with geological regions could be likely explained by the effect of dispersal limitation on microbial community assembly, population and their gene flow. When comparing the genomic similarity between any two of the four regions, we found that there was significant difference among these region pairs, with the shortest paired regions (i.e., China-Russia) having the highest genomic similarity (Figure 4c), especially based on the strain-level genomes (BH-adjusted *p* = 1.4e-5 ~ 8.4e-9, Wilcoxon test, Figure 4d).

Such observed decay in functional composition and genomes shows microbial divergence associated with dispersal limitation over continental-scale distances, implying the importance of evolutionary history and/or environmental selection in shaping soda lake microbial communities. The similar geography-related divergence has been found in microbial lineages or communities, such as a widespread freshwater *Polynucleobacter* population consisting of 113 strains across a geographic range over 3,000 km (Hoetzinger et al., 2021) and the river and lake microbiome across a 2,500-km transect in China (Cheng et al., 2024). Considering the large population size and high dispersal ability, microorganisms are reported to have higher habitat transition rates than anticipated, such as crossing the salt barrier between marine and freshwater (Paver et al., 2018). It has been shown recently that bacteria dispersal across continents is facilitated by dust particles, e.g., the terrestrial and dust-associated bacteria over Atlantic and Pacific (Lang-Yona et al., 2022), and hitchhike with migratory waterfowl (Conklin et al., 2022). Although the dispersal pathways for soda lake microbiomes around the world cannot be determined based on cultivation-independent sequencing technologies, here we provide the evidence for the geography-related divergence at a global scale based on the decay of functional composition and genome similarity.

### 3.4 Wide-spread microbes showed larger genome size than the endemism

**Figure 5:**
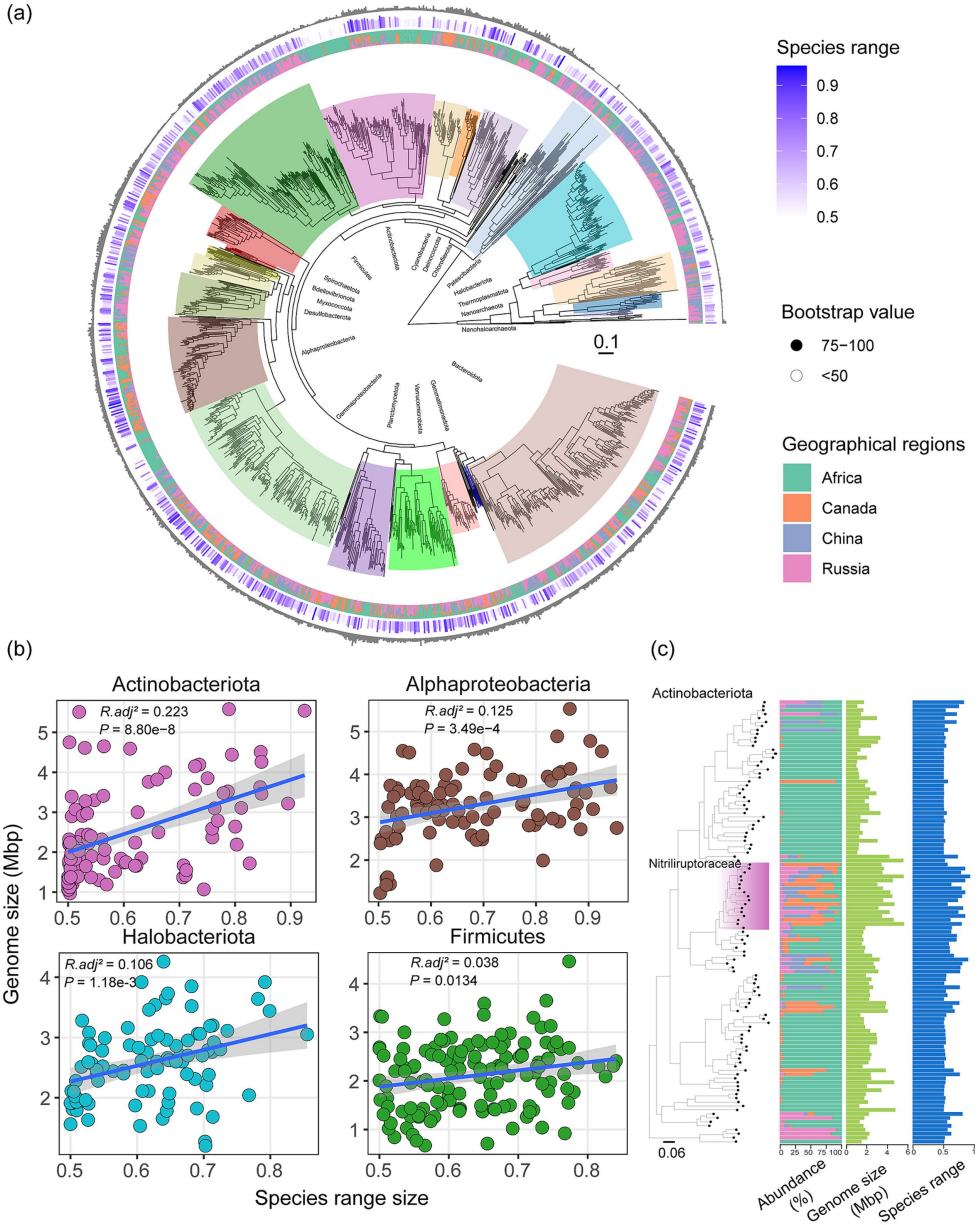
Microbial genome size increased with their geographic range. **(a)** The maximum-likelihood phylogeny of the representative species genomes from global lakes. For each species, genome size, geographic range and abundance distribution across four regions were annotated from outer to inner circles, respectively. The species geographic range was calculated as one minus the standard deviation of the percentage of the species abundance across geographical regions. The method for building tree sees the Method section. **(b)** The linear relationships between genome size and species range size across major phyla. The adjusted coefficients and significance results of regression models were shown at the subpanels. **(c)** The illustration of genome size and species range association using the phylum Actinobacteriota. The family Nitriliruptoraceae within Actinobacteriota were highlighted in the tree.

The phylogenomic tree of the 1,330 species showed that the majority of soda lake microbes were distributed across more than one geographical region, whereas few geographical endemism was scattered in several lineages (Figure 5a). Their phylogenetic relatedness of microbial lineages across geographically distant soda lakes further supported the scenario above, that soda lake microbial communities likely have a common and ancient evolutionary origin, rather than independently evolve multiple times in geographically isolated regions. For geographical endemism, several lineages, such as Acidimicrobiales and Actinomycetales within the phylum Actinobacteriota, and one lineage within Gammaproteobacteria were dominantly present in East African soda lakes, whereas the lineages of Haloarchaeota were predominantly detected in Russia and China (Figures 1c, 5a).

To better characterize the species dispersal ability and their associations with genomic features, we calculated an index of species range size to indicate the species distribution across geographical regions. The index considers the number of geographical regions where a species occurs and the variation in its relative abundance across regions. In our case, the index changes from 0.5 to 1.0, with the minimum value meaning a species exclusively restricted in one geographical region, and the maximum meaning its evenly distribution across multiple regions. We found that species range size was significantly and positively correlated with their genome size (*p* = 6.40e-12, *R^2^_adj_* = 0.03, Supplementary Figure S2). The positive correlation between species range size and genome size was consistently observed across the major phyla, such as Haloarchaeota (*p* = 1.15e-3, *R^2^_adj_* = 0.12) and Actinobacteria (*p* = 8.87e-8, *R^2^_adj_* = 0.23, Figures 5b,c). The close relationships of bacterial range size and genome size have been found at a wide range of scales. For example, the microbes inhabiting a greater range of environments have larger and potentially more versatile genomes than those with restricted distributions through a study of the spatial distribution of soil microbes in about 600 soil samples within a park (Barberán et al., 2014). When expanding at a much larger spatial scales, there is a linkage between latitudinal range size distribution and microbial genome size of biofilm bacterial communities in about 200 streams across a 1,000 km latitudinal gradient (Lear et al., 2017). Bacterial range size may be impacted by their capacity to cope with or tolerate environmental change, as microbes with larger genomes expectedly exhibit greater metabolic versatility to environmental change (Bentkowski et al., 2015). These positive relationships here imply that either microbial genome reduction may have occurred in these endemic species, or genome expansion was closely associated with geographical dispersal owing to regional adaptation. Although there was unfortunately no additional evidence for supporting any of the directions or both, soda lake microbial communities have a complex evolutionary history accompanied with their dispersal across continents.

### 3.5 Uneven transition of soda lake microbiome across continents

Considering that the occurrence of phylogenetically-related lineages in global soda lakes, we asked how often the cross-continent transitions have occurred during their evolutionary history. The estimation of microbial transition history might be insightful to explain the observed microbial diversity and biogeography across continents (Louca, 2022). For example, the soda lake microbial communities on one continent would exhibit similar taxonomic and functional composition to those on the other if transition rates between two continents were relatively high over evolutionary time. With the phylogeny of 1,330 species-level genomes, we inferred the global patterns and rates of habitat transition for soda lake microbial communities across continents with ancestral state reconstruction using Markov chain Monte Carlo methods (Pagel et al., 2004).

**Figure 6:**
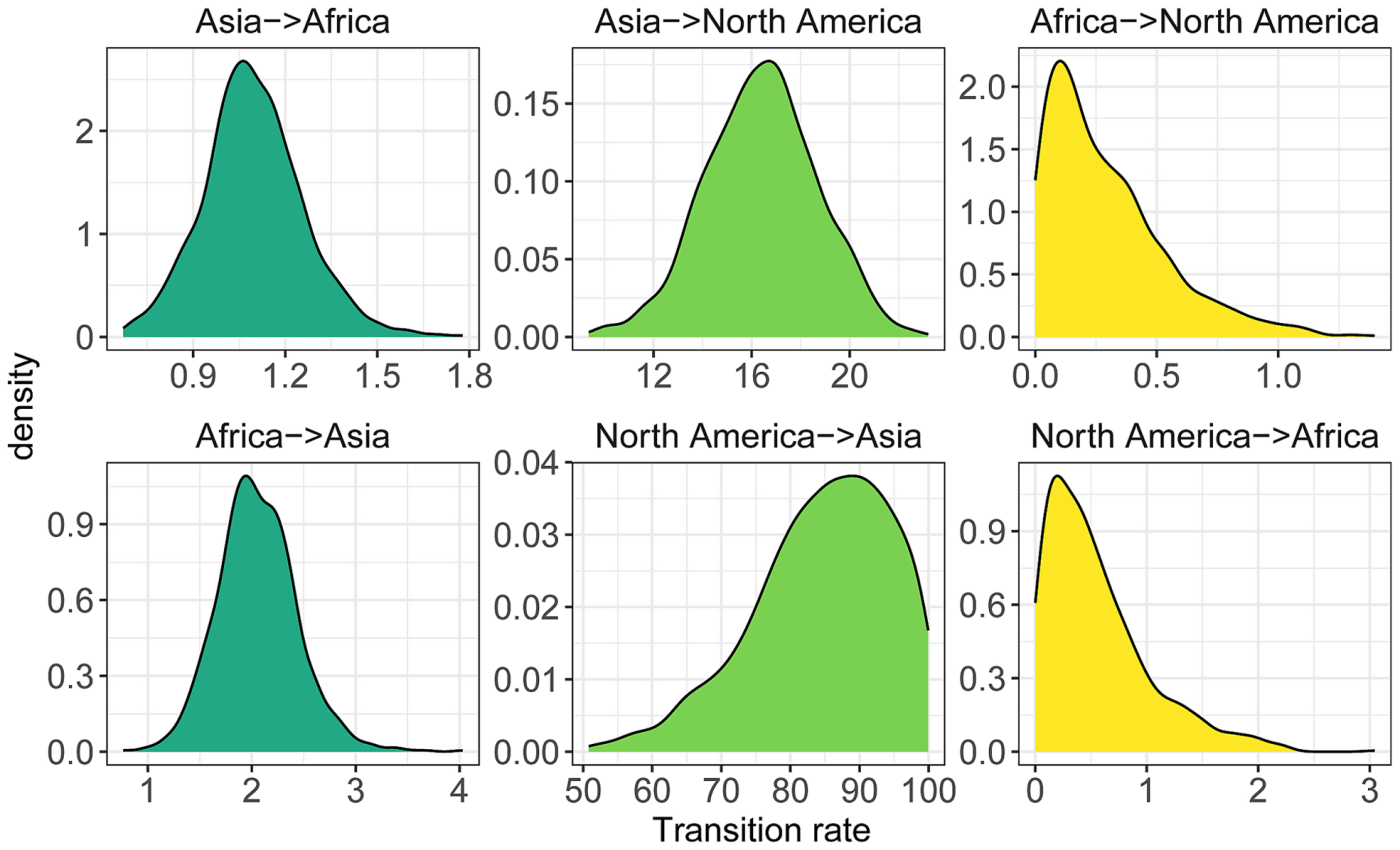
The cross-continent evolutionary transition rates of soda lake microbial communities. Posterior probability distributions of the transition rates for microbial communities in global soda lakes were estimated using ancestral state reconstruction method implemented by BayesTraits (see the Method section). The transition rate indicates the overall speed at which microbial transition between different pairs of continents (African, Asia and North America) has occurred. Both directions between any pair of continents were considered when modeling transition rates along the phylogeny.

The model results revealed that soda lake communities exhibited the highest rates of transition between Asia and North America, followed by Asia and Africa, and then North America and Africa (Figure 6). The same transition patterns were observed when modeling the reverse direction (Asia-to-North America and the reverse, see the Methods). Particularly, the transition rates between Asia and North America were at least one or two orders of magnitude higher than between the other two pairs of continents, despite the much longer geographical distance between Asia and North America compared to Africa. The uneven transition patterns were consistent with earlier observations that the highest proportion of geographical endemism in the African region (Figure 2b), and the higher genomic divergence between Africa and other regions (Figure 4c). Besides, we further noted that substantial variation in the rates of continent-level transition and their reverse direction, such as nearly five times transition rates from North America to Asia higher than the reverse direction. These asymmetry in transition rates could be contributed by multiple factors, including the difficulty of dispersing geological barrier from both directions, and/or extinction of some microbial lineages over evolutionary time.

The paleogeography patterns of the continents likely explain such lower transition rates between Africa and other two continents. Given the distance among contemporary continents, geographical factors alone fail to explain the inferred transition patterns. However, these patterns are likely associated with the paleogeography patterns of these continents over deep time, considering the ancient history of soda lakes (Stüeken et al., 2015). For example, the continents of Asia and North America are parts of the Laurasia supercontinent, which is connected to the Siberia (northern Asia) for nearly 1.2 billion years (Ernst et al., 2016). The long-lived connection likely provides an opportunity for the higher transition rates between soda lakes of Asia and North America. In contrast, the Africa continent breaks away from the Gondwana supercontinent, which is separated from the Laurasia at the 200 Mya ago (Mitchell et al., 2021). Note that owing to the limited fossil record for microbial species, we cannot date these evolutionary events across the phylogeny and further place them within a geologic time scale. However, the paleography of these continents provides possible transition pathways and barriers for microbial dispersal over long distance, shaping the diversity and evolutionary history of soda lake microbial communities across continents.

## 4 Conclusion

By integrating 14 newly sequenced metagenomes from the East African soda lakes with 37 globally available samples, we revealed substantial uncultured microbial diversity in these extreme environments and identified multiple phylogenetically related lineages exhibiting broad geographic distributions. These widespread microbes are characterized by larger genome size than geographical endemism, suggesting a complex evolutionary history accompanied with their dispersal and colonization across continents. The distribution of widespread species and their phylogenetic relatedness support an evolutionary scenario of an ancient common origin for global soda lake microbial communities.

Soda lakes could be used as ideal ecosystems for studying microbial biogeography. For example, the extreme conditions such as high pH and/or salinity create a filter for microbial survival, and soda lakes are globally distributed but occur in isolated, inland basins. Our study of global soda lake metagenomes here shows that dispersal limitation plays an important role in shaping functional composition and microbial speciation in these extreme environments. This hypothesis is supported by the general biogeographic patterns for microbial taxonomic and functional composition, as well as genomic divergence. These biogeographic patterns are consistent with the geographic isolation of archaeal and bacterial populations in non-extreme environments (Whitaker et al., 2003; Hoetzinger et al., 2021). To the best of our knowledge, this is the first report on the decay of functional composition and genome similarity in soda lakes at a global scale. These biogeographical patterns are likely associated with the uneven frequency of the cross-continent transitions during their evolutionary history. These results improve the understanding of microbial biogeography at a global scale, and provide novel insights into the mechanisms underlying the geographical distribution when considering microbial transition evolutionary history.

## Data Availability

The sequence for metagenomic reads and MAGs in the study has been deposited in the NCBI database under the accession number PRJNA857294, respectively. The codes and scripts for sequence analyses in the study are available at the FigShare (10.6084/m9.figshare.28152113).
