# Supplementary material for: Biogeography of soda lake microbiome and uneven cross-continent transition rates
Source: Front Microbiol. 2025 Jul 24;16:1614302. doi: 10.3389/fmicb.2025.1614302 (PMC12330390; doi:10.3389/fmicb.2025.1614302)
Supplement: Supplementary file 1 [file Supplementary_file_1.pdf]

Table S1. The acession number and sequencing statistics of metagenomic samples of global soda lakes used in the study.

Sample	total_bases (Gbase)	total_reads (Million)	Geological region	Lake name	Sample type	pH	salinity	Latitude	Longitude	Accession number (NCBI/IMG	Reference
AF02	20.69	159.19	Africa	small Momela Lake	sediment	9.6	60.6	-3.2195	36.8958	SRR20069368	This study
AF03	18.73	132.39	Africa	Lake Manyara	sediment	9.23	80	-3.7202	35.8287	SRR20069404	
AF05	19.26	137.84	Africa	Lake Kindai	sediment	9.17	428.2	-4.8352	34.7348	SRR20069382	
AF08	20.06	140.87	Africa	Lake Balangita	sediment	9.93	8532	-4.4	35.3025	SRR20069370	
AF09	18.82	136.54	Africa	Big Momela Lake	sediment	10.12	206.3	-3.2254	36.9086	SRR20069367	
AF10	20.55	147.06	Africa	Lake Jipe	sediment	9.11	5.7	-3.6311	37.7564	SRR20069403	
AF20	23.15	163.56	Africa	Lake Eyasi	sediment	9.85	1014	-3.5669	35.249	SRR20069394	
AF23	17.36	125.28	Africa	small Momela Lake	water	9.6	60.6	-3.2195	36.8958	SRR20069379	
AF24	18.82	135.49	Africa	Big Momela Lake	water	10.12	206.3	-3.2254	36.9086	SRR20069378	
AF25	19.14	134.84	Africa	Lake Jipe	water	9.11	5.7	-3.6311	37.7564	SRR20069377	
AF29	25.56	188.26	Africa	Lake Eyasi	water	9.85	1014	-3.5669	35.249	SRR20069373	
AF30	18.18	126.26	Africa	Lake Manyara	water	9.23	80	-3.7202	35.8287	SRR20069391	
AF32	21.44	148.65	Africa	Lake Kindai	water	9.17	428.2	-4.8352	34.7348	SRR20069389	
AF34	37.78	273.57	Africa	Lake Balangita	water	9.93	8532	-4.4	35.3025	SRR20069387	
Gb0053562	13.89	151.95	Russia	Lake Bitter-1 (B1)	sediment	10.2	40	51.67	79.91	Gb0053562	(Vavourakis, 2016, Front Microbiol)
Gb0053563	16.17	188.46	Russia	Lake Tanatar-3	sediment	10	11	51.67	79.81	Gb0053563	
Gb0053563	31.52	268.29	Russia	Lake Tanatar-3	sediment	10	11	51.67	79.81	Gb0053563	
Gb0054152	26.48	247.34	Russia	Picturesque Lake	water	9.5	25	51.73	79.87	Gb0054152	
Gb0054153	14.04	159.09	Russia	Lake Bitter-1	water	10.2	40	51.67	79.91	Gb0054153	
Gb0054153	23.15	252.46	Russia	Lake Bitter-1	water	10.2	40	51.67	79.91	Gb0054153	
Gb0054154	32.73	298.56	Russia	Lake Tanatar-5	water	9.9	17	51.62	79.84	Gb0054154	
Gb0054155	24.92	236.57	Russia	ke Tanatar trona crystalli	water	9.9	30	51.65	79.75	Gb0054155	
Gb0054156	9.23	97.13	Russia	Cock Soda Lake	sediment	10.1	7	52.11	79.17	Gb0054156	(Vavourakis, 2018, Microbiome)
Gb0054156	27.08	228.2	Russia	Cock Soda Lake	sediment	10.1	7	52.11	79.17	Gb0054156	
Gb0054157	26.73	276.66	Russia	Lake Tanatar-3	sediment	9.9	40	51.67	79.9	Gb0054157	
Gb0054158	25.83	234.79	Russia	Cock Soda Lake	sediment	10.2	10	51.66	79.79	Gb0054158	
SRR7083929	65.88	486.01	Russia	Cock Soda Lake	sediment	9.9	10	52.11	79.17	SRR7083929	(Vavourakis, 2019, BMC)
SRR7083930	74.04	533.41	Russia	Cock Soda Lake	water	9.9	5.5	52.11	79.17	SRR7083930	
SRR7083934	75.28	548.25	Russia	Cock Soda Lake	sediment	9.9	10	52.11	79.17	SRR7083934	
SRR7901803	21.38	155.4	Canada	Deer Lake	water	10.4	NA	51.35416	-121.24527	SRR7901803	(Zorz, 2019, Nat Comm)
SRR7901804	24.81	177.77	Canada	Good Enough Lake	water	10.4	NA	51.32972	-121.641389	SRR7901804	
SRR7901805	23.9	170.75	Canada	Last Chance Lake	water	10.7	NA	51.3275	-121.63305	SRR7901805	
SRR7901806	24.2	174.21	Canada	Probe Lake	water	10.3	NA	51.45055	-121.3875	SRR7901806	
SRR9330139	14.85	105.46	China	Hutong Qagan Lake	sediment	9.87	22.4	39.1833	109.0042	SRR9330139	(Zhao, 2022, Front Microbiol)
SRR9330140	8.14	57.29	China	Hutong Qagan Lake	sediment	10.01	17	39.1825	109.0019	SRR9330140	
SRR9330141	14.77	105.18	China	Hutong Qagan Lake	sediment	9.78	27	39.2056	109.0044	SRR9330141	
SRR9330142	14.93	106.87	China	Hutong Qagan Lake	sediment	9.95	26	39.2039	109.0022	SRR9330142	
SRR9330143	12.53	91.32	China	Habor Lake	sediment	9.93	20.7	39.4217	108.6808	SRR9330143	
SRR9330144	18.83	132.53	China	Habor Lake	sediment	10	15	39.4161	108.6803	SRR9330144	
SRR9330145	18.91	132.87	China	Habor Lake	sediment	10.8	32	39.4211	108.6817	SRR9330145	
SRR9330146	13.91	97.59	China	Habor Lake	sediment	9.89	27.3	39.4214	108.6811	SRR9330146	
SRR9330147	14.55	103.59	China	Hutong Qagan Lake	water	9.95	26	39.2039	109.0022	SRR9330147	
SRR9330148	12.2	90.58	China	Hutong Qagan Lake	water	9.87	22.4	39.1833	109.0042	SRR9330148	
SRR9330149	12.09	89.89	China	Hutong Qagan Lake	water	10.01	17	39.1825	109.0019	SRR9330149	
SRR9330150	14.46	102.76	China	Hutong Qagan Lake	water	10.08	5.5	39.1903	108.9983	SRR9330150	
SRR9330151	15.79	112.34	China	Habor Lake	water	9.89	27.3	39.4214	108.6811	SRR9330151	
SRR9330152	14.87	105.76	China	Habor Lake	water	9.93	20.7	39.4217	108.6808	SRR9330152	
SRR9330153	10.73	79.53	China	Habor Lake	water	10	15	39.4161	108.6803	SRR9330153	
SRR9330154	15.95	111.87	China	Hutong Qagan Lake	water	9.78	27	39.2056	109.0044	SRR9330154	
SRR9330155	15.77	112.25	China	Hutong Qagan Lake	sediment	10.08	5.5	39.1903	108.9983	SRR9330155	
SRR9330156	17.29	128.29	China	Habor Lake	water	10.8	32	39.4211	108.6817	SRR9330156	

Table S2. The list of culturable strains isolated from global soda lakes.

Strain_name	Phylum	Source	Reference
Candidatus Chloroploca asiatica	Chloroflexi	Soda lakes, Transbaikal, Russia, and Mongolia	(Grant and Jones, 2016)
Candidatus Contubernalis alkalaceticum	Firmicutes	Lake Khatyn, Kulunda Steppe, Russia	(Grant and Jones, 2016)
Candidatus Syntrophonatronum acetioxidans	Firmicutes	Bitter Lake, Kulunda Steppe, Russia	(Grant and Jones, 2016)
Alcalilimnicola halodurans	Gammaproteobacteria	Lake Natron, Tanzania	(Grant and Jones, 2016)
Alkalibacillus haloalkaliphilus	Firmicutes	Sambhar Lake, India	(Grant and Jones, 2016)
Alkalibacter saccharofermentans	Firmicutes	Lake Nizhnee Beloe, Russia	(Grant and Jones, 2016)
Alkaliflexus imshenetskii	Bacteroidota	Lake Verkhnee Beloe, Russia	(Grant and Jones, 2016)
Alkalilacustris brevis	Alphaproteobacteria	a soda lake in Jilin, China	This study
Alkalilimnicola ehrlichii	Gammaproteobacteria	Mono Lake, CA, USA	(Grant and Jones, 2016)
Alkalimonas amylolytica	Gammaproteobacteria	Lake Chahannor, Inner Mongolia	(Grant and Jones, 2016)
Alkalimonas delamerensis	Gammaproteobacteria	Lake Elmenteita, Kenya	(Grant and Jones, 2016)
Alkaliphilus peptidofermentans	Firmicutes	Lake Verkhnee Beloe, Russia	(Grant and Jones, 2016)
Alkalitalea saponilacus	Firmicutes	Soap Lake, WA, USA	(Grant and Jones, 2016)
Amphibacillus fermentum	Firmicutes	Lake Magadi, Kenya	(Grant and Jones, 2016)
Amphibacillus haojiensis	Firmicutes	Haoji soda lake, Inner Mongolia	(Grant and Jones, 2016)
Amphibacillus jilinensis	Firmicutes	Soda lake, Jilin, China	(Grant and Jones, 2016)
Amphibacillus tropicus	Firmicutes	Lake Magadi, Kenya	(Grant and Jones, 2016)
Anaerobacillus alkalilacustre	Firmicutes	Lake Khatyn, Russia	(Grant and Jones, 2016)
Anaerobranca gottschalkii	Firmicutes	Lake Magadi, Kenya	(Grant and Jones, 2016)
Anaerovirgula multivorans	Firmicutes	Owens Lake, CA, USA	(Grant and Jones, 2016)
Anoxynatronum sibiricum	Firmicutes	Lake Nizhnee Beloe, Russia	(Grant and Jones, 2016)
Bacillus alkalicola	Firmicutes	Lake Zabuye, Tibet	(Grant and Jones, 2016)
Bacillus alkalidiazotrophicus	Firmicutes	Soda soils, Mongolia	(Grant and Jones, 2016)
Bacillus alkalisediminis	Firmicutes	Soda ponds, Kiskunsag, Hungary	(Grant and Jones, 2016)
Bacillus arsenicoselenatis	Firmicutes	Mono Lake, CA, USA	(Grant and Jones, 2016)
Bacillus aurantiacus	Firmicutes	Soda lake, Hungary	(Grant and Jones, 2016)
Bacillus beveridgei	Firmicutes	Mono Lake, CA, USA	(Grant and Jones, 2016)
Bacillus bogoriensis	Firmicutes	Lake Bogoria, Kenya	(Grant and Jones, 2016)
Bacillus caseinilyticus	Firmicutes	Lonar Lake, India	(Grant and Jones, 2016)
Bacillus chagannorensis	Firmicutes	Lake Chagannor, Inner Mongolia	(Grant and Jones, 2016)
Bacillus daliensis	Firmicutes	Dali Lake, Inner Mongolia	(Grant and Jones, 2016)
Bacillus daqingensis	Firmicutes	Soda soil, Daqing, China	(Grant and Jones, 2016)
Bacillus locisalis	Firmicutes	Soda lakes, China, Kenya, Tanzania	(Grant and Jones, 2016)
Bacillus lonarensis	Firmicutes	Lonar Lake, India	(Grant and Jones, 2016)
Bacillus selenitireducens	Firmicutes	Mono Lake, CA, USA	(Grant and Jones, 2016)
Belliella buryatensis	Firmicutes	water of the alkaline brackish Lake Solenoe, Buryatia, Russia	This study
Belliella kenyensis	Firmicutes	Lake Elmenteita, Kenya	(Grant and Jones, 2016)
Bogoriella caseilytica	Actinobacteriota	Lake Bogoria, Kenya	(Grant and Jones, 2016)
Candidatus Phormidium alkaliphilum	Cyanobacteria	Cariboo Plateau soda lakes, Canada	This study
Cecembia lonarensis	Bacteroidota	Lonar Lake, India	(Grant and Jones, 2016)

Cellulomonas bogoriensis	Actinobacteriota	Lake Bogoria, Kenya	(Grant and Jones, 2016)
Cohaesibacter haloalkalitolerans	Alphaproteobacteria	Lonar Lake, India	(Grant and Jones, 2016)
Desulfitispora alkaliphila	Firmicutes	Soda lakes, Kulunda, Russia	(Grant and Jones, 2016)
Desulfitispora elongata	*Bacillota*	sediments of hypersaline alkaline lakes in Kulunda Steppe, A	This study
Desulfobotulus alkaliphilus	Deltaproteobacteria	Soda lakes, Kulunda, Russia	(Grant and Jones, 2016)
Desulfobulbus alkaliphilus	Deltaproteobacteria	Soda lake, Kulunda, Russia	(Grant and Jones, 2016)
Desulfohalophilus alkaliarsenatis	Deltaproteobacteria	Searles Lake, CA, USA	(Grant and Jones, 2016)
Desulfonatronobacter acidivorans	Deltaproteobacteria	Soda lake, Kulunda, Russia	(Grant and Jones, 2016)
Desulfonatronospira delicata	Deltaproteobacteria	Wadi Natrun, Egypt	(Grant and Jones, 2016)
Desulfonatronospira sulfatiphila	*Deltaproteobacteria*	sediments of hypersaline alkaline lakes in Kulunda Steppe, A	This study
Desulfonatronospira thiodismutans	Deltaproteobacteria	Soda lakes, Kulunda, Russia	(Grant and Jones, 2016)
Desulfonatronovibrio halophilus	Deltaproteobacteria	Soda lake, Kulunda, Russia	(Grant and Jones, 2016)
Desulfonatronovibrio hydrogenovorans	Deltaproteobacteria	Lake Magadi, Kenya	(Grant and Jones, 2016)
Desulfonatronovibrio magnus	Deltaproteobacteria	Soda lakes, Kulunda, Russia	(Grant and Jones, 2016)
Desulfonatronovibrio thiodismutans	Deltaproteobacteria	Soda lakes, Kulunda, Russia	(Grant and Jones, 2016)
Desulfonatronum buryatense	Deltaproteobacteria	Alkaline lake, Buryatia, Russia	(Grant and Jones, 2016)
Desulfonatronum cooperativum	Deltaproteobacteria	Lake Khatyn, Russia	(Grant and Jones, 2016)
Desulfonatronum lacustre	Deltaproteobacteria	Lake Khatyn, Russia	(Grant and Jones, 2016)
Desulfonatronum thioautotrophicum	Deltaproteobacteria	Soda lakes, Kulunda, Russia	(Grant and Jones, 2016)
Desulfonatronum thiodismutans	Deltaproteobacteria	Mono Lake, CA, USA	(Grant and Jones, 2016)
Desulfonatronum thiosulfatophilum	Deltaproteobacteria	Soda lake, Kulunda, Russia	(Grant and Jones, 2016)
Desulfonatronum zhilinea	Deltaproteobacteria	Lake Alginskoe, Transbaikal, Russia	(Grant and Jones, 2016)
Desulfuribacillus alkaliarsenatis	Deltaproteobacteria	Soda lake, Kulunda, Russia	(Grant and Jones, 2016)
Desulfurispirillum alkaliphilum	Chrysiogenetes	Mixed sample, bioreactor	(Grant and Jones, 2016)
Desulfurivibrio alkaliphilus	Deltaproteobacteria	Wadi Natrun, Egypt	(Grant and Jones, 2016)
Dethiobacter alkaliphilus	Firmicutes	Soda lake, Mongolia	(Grant and Jones, 2016)
Dietzia natronolimnaea	Actinobacteriota	Lake Nakuru, Kenya	(Grant and Jones, 2016)
Ectothiorhodosinus mongolicus	Gammaproteobacteria	Lake Dzun Ulziin, Mongolia	(Grant and Jones, 2016)
Ectothiorhodospira haloalkaliphila	Gammaproteobacteria	Wadi Natrun, Egypt	(Grant and Jones, 2016)
Ectothiorhodospira magna	Gammaproteobacteria	Soda lake, Transbaikal, Russia	(Grant and Jones, 2016)
Ectothiorhodospira shaposhnikovii	Gammaproteobacteria	Soda lakes, Transbaikal, Russia	(Grant and Jones, 2016)
Ectothiorhodospira vacuolata	Gammaproteobacteria	Kenyan and Egyptian soda lakes	(Grant and Jones, 2016)
Ectothiorhodospira variabilis	Gammaproteobacteria	Wadi Natrun, Egypt; Siberia, Mongolia	(Grant and Jones, 2016)
Fuchsiella alkaliacetigena	Firmicutes	Lake Tanatar III, Altai, Russia	(Grant and Jones, 2016)
Fuchsiella ferrireducens	Firmicutes	Lake Tanatar III, Altai, Russia	(Grant and Jones, 2016)
Geoalkalibacter ferrihydriticus	Deltaproteobacteria	Lake Khatyn, Kulunda Steppe, Russia	(Grant and Jones, 2016)
Georgenia satyanarayanai	Actinobacteriota	Lonar Lake, India	(Grant and Jones, 2016)
Halalkaliarchaeum desulfuricum	*Halobacteriota*	alkaline Searles Lake in south-western Siberia	This study
Halanaerobium hydrogenoformans	Firmicutes	Soap Lake, WA, USA	(Grant and Jones, 2016)
Halarsenatibacter silvermanii	Firmicutes	Searles Lake, CA, USA	(Grant and Jones, 2016)
Halolactibacillus alkaliphilus	Firmicutes	Xiarinaoer Lake, Inner Mongolia	(Grant and Jones, 2016)
Halomonas alkalitolerans	Gammaproteobacteria	Soda meadow, Heilongjiang, China	(Grant and Jones, 2016)
Halomonas campaniensis	Gammaproteobacteria	Alkaline mineral pool, Italy	(Grant and Jones, 2016)
Halomonas campisalis	Gammaproteobacteria	Alkali Lake, WA, USA, Lake Magadi, Kenya	(Grant and Jones, 2016)

Halomonas chromatireducens	Gammaproteobacteria	Soda soil, Kulunda, Russia	(Grant and Jones, 2016)
Halomonas kenyensis	Gammaproteobacteria	Lake Magadi, Kenya	(Grant and Jones, 2016)
Halomonas magadiensis	Gammaproteobacteria	Lake Magadi, Kenya	(Grant and Jones, 2016)
Halomonas mongoliensis	Gammaproteobacteria	Soda lake, Mongolia	(Grant and Jones, 2016)
Halomonas montanilacus	*Gammaproteobacteria*	hypersaline Lake Pengyanco on the Tibetan Plateau	This study
Halonatronum saccharophilum	Firmicutes	Lake Magadi, Kenya; Mongolia	(Grant and Jones, 2016)
Halorhodospira abdelmalekii	Gammaproteobacteria	Wadi Natrun, Egypt	(Grant and Jones, 2016)
Halorhodospira halochloris	Gammaproteobacteria	Wadi Natrun, Egypt	(Grant and Jones, 2016)
Halorhodospira halophila	Gammaproteobacteria	Wadi Natrun, Egypt	(Grant and Jones, 2016)
Halostagnicola alkaliphila	Halobacteriota	commercial rock salt	This study
Halostagnicola bangensis	Halobacteriota	sediment of the soda lake Bange in the region of Tibet, China	This study
Halostagnicola larsenii	Halobacteriota	a saline lake in Inner Mongolia, China.	This study
Heliorestis acidaminivorans	Firmicutes	Wadi Natrun, Egypt	(Grant and Jones, 2016)
Heliorestis baculata	Firmicutes	Lake Ostozhe, Russia	(Grant and Jones, 2016)
Heliorestis convoluta	Firmicutes	Wadi Natrun, Egypt	(Grant and Jones, 2016)
Heliorestis daurensis	Firmicutes	Soda lake, Siberia	(Grant and Jones, 2016)
Indibacter alkaliphilus	Bacteroidota	Lonar Lake, India	(Grant and Jones, 2016)
Jonesia quinghaiensis	Actinobacteriota	Soda lake, Qinghai, China	(Grant and Jones, 2016)
Litoribacter ruber	Bacteroidota	Soda lake, Yongsheng, China	(Grant and Jones, 2016)
Longimonas haloalkaliphila	*Rhodothermaeota*	sediments of hypersaline alkaline lakes in Kulunda Steppe, A	This study
Lunatibacter salilacus	*Bacteroidota*	a sediment sample of alkaline lake, Qinghai Lake, Qinghai, C	This study
Lunatimonas lonarensis	Bacteroidota	Lonar Lake, India	(Grant and Jones, 2016)
Marinospirillum alkaliphilum	Gammaproteobacteria	Haoji Lake, Inner Mongolia	(Grant and Jones, 2016)
Marinospirillum celere	Gammaproteobacteria	Mono Lake, CA, USA	(Grant and Jones, 2016)
Methanonatronarchaeum thermophilum	*Halobacteriota*	sediments from hypersaline chloride–sulfate and soda lakes i	This study
Methanosalsum natronophilum	*Halobacteriota*	mixed sediments of Kulunda Steppe soda lakes, Altai, Russia	This study
Methylohalomonas lacus	Gammaproteobacteria	Soda lake, Kulunda, Russia	(Grant and Jones, 2016)
Methylomicrobium alcaliphilum	Gammaproteobacteria	Soda lake, Tuva, Russia	(Grant and Jones, 2016)
Methylomicrobium buryatense	Gammaproteobacteria	Soda lakes, Transbaikal, Russia	(Grant and Jones, 2016)
Methylomicrobium kenyense	Gammaproteobacteria	Soda lakes, Kenya	(Grant and Jones, 2016)
Methylonatrum kenyense	Gammaproteobacteria	Lake Magadi, Kenya	(Grant and Jones, 2016)
Methylophaga alcalica	Gammaproteobacteria	Soda lake, Mongolia	(Grant and Jones, 2016)
Methylophaga lonarensis	Gammaproteobacteria	Lonar Lake, India	(Grant and Jones, 2016)
Methylophaga natronica	Gammaproteobacteria	Soda lake, Transbaikal, Russia	(Grant and Jones, 2016)
Mongolicoccus roseus	Bacteroidota	Lake Horsemeno, Mongolia	(Grant and Jones, 2016)
Mongoliitalea lutea	Bacteroidota	Lake Dugerno, Mongolia	(Grant and Jones, 2016)
Natranaerobaculum magadiense	Firmicutes	Lake Magadi, Kenya	(Grant and Jones, 2016)
Natranaerobius thermophilus	Firmicutes	Wadi Natrun, Egypt	(Grant and Jones, 2016)
Natranaerobius trueperi	Firmicutes	Wadi Natrun, Egypt	(Grant and Jones, 2016)
Natranaerovirga hydrolytica	Firmicutes	Soda lake, Kulunda, Russia	(Grant and Jones, 2016)
Natranaerovirga pectinivora	Firmicutes	Soda lake, Kulunda, Russia	(Grant and Jones, 2016)
Natroniella acetigena	Firmicutes	Lake Magadi, Kenya	(Grant and Jones, 2016)
Natroniella sulfidigena	Firmicutes	Wadi Natrun, Kulunda Steppe	(Grant and Jones, 2016)
Natronincola ferrireducens	Firmicutes	Lake Verkhnee Beloe, Russia	(Grant and Jones, 2016)

Natronincola histidinovorans	Firmicutes	Lake Magadi, Kenya	(Grant and Jones, 2016)
Natronincola peptidovorans	Firmicutes	Lake Verkhnee Beloe, Russia	(Grant and Jones, 2016)
Natronobacillus azotifigens	Firmicutes	Soda soils, Siberia, Libya, Egypt, Mongolia	(Grant and Jones, 2016)
Natronocella acetinitrilica	Gammaproteobacteria	Soda lake, Kulunda, Russia	(Grant and Jones, 2016)
Natronoflexus pectinivorans	Firmicutes	Soda lake, Kulunda, Russia	(Grant and Jones, 2016)
Natronolimnobius sulfurireducens	*Halobacteriota*	hypersaline alkaline lakes in south-western Siberia	This study
Natronospira proteinivora	*Gammaproteobacteria*	Kulunda Steppe soda lakes, Altai, Russia	This study
Natronotalea proteinilytica	*Rhodothermaeota*	sediments of hypersaline alkaline lakes in Kulunda Steppe, A	This study
Natronovirga wadinatrunensis	Firmicutes	Wadi Natrun, Egypt	(Grant and Jones, 2016)
Nesterenkonia aethiopica	Actinobacteriota	Lake Abijatta, Ethiopia	(Grant and Jones, 2016)
Nesterenkonia cremea	Actinobacteriota	Lonar soda lake in India	This study
Nesterenkonia natronophila	Actinobacteriota	soda lake sediment of Lake Magadi, Tanzania	This study
Nesterenkonia suensis	Actinobacteriota	Alkaline salt pan, Botswana	(Grant and Jones, 2016)
Nitriliruptor alkaliphilus	Actinobacteriota	Soda lakes, Kulunda, Russia	(Grant and Jones, 2016)
Nitrincola alkalilacustris	*Gammaproteobacteria*	soda pans in the Kiskunság National Park, Hungary	This study
Nitrincola alkalisediminis	*Gammaproteobacteria*	the alkaline Lonar Lake in Maharashtra, India	This study
Nitrincola lacisaponensis	Gammaproteobacteria	Soap Lake, WA, USA	(Grant and Jones, 2016)
Nitrincola nitratireducens	*Gammaproteobacteria*	Lonar Lake, Buldhana district, India.	This study
Nitrincola tibetensis	*Gammaproteobacteria*	an alkaline lake Lake XuguoCo on the Tibetan Plateau.	This study
Nitritalea halalkaliphila	Bacteroidota	Lonar Lake, India	(Grant and Jones, 2016)
Nitrobacter alkalicus	Alphaproteobacteria	Soda lakes, Kenya, Russia	(Grant and Jones, 2016)
Nitrosomonas halophila	Betaproteobacteria	Soda lakes, Mongolia	(Grant and Jones, 2016)
Paracoccus bogoriensis	Alphaproteobacteria	Lake Bogoria, Kenya	(Grant and Jones, 2016)
Proteinivorax tanatarense	Firmicutes	Lake Tanatar III, Altai, Russia	(Grant and Jones, 2016)
Rhodobaca barguzinensis	Alphaproteobacteria	Soda lake, Barguzin, Russia	(Grant and Jones, 2016)
Rhodobaca bogoriensis	Alphaproteobacteria	Lake Bogoria, Kenya	(Grant and Jones, 2016)
Rhodobaculum claviforme	Alphaproteobacteria	Transbaikal steppe lakes, Russia	(Grant and Jones, 2016)
Rhodovulum steppense	Alphaproteobacteria	Steppe soda lake, Russia	(Grant and Jones, 2016)
Rhodovulum tesquicola	Alphaproteobacteria	Steppe soda lake, Russia	(Grant and Jones, 2016)
Romboutsia sedimentorum	Firmicutes	Alkaline–saline lake, Daqing, China	(Grant and Jones, 2016)
Roseibacula alcaliphilum	Alphaproteobacteria	Lake Doroninskoe, Russia, Nuyanzina-Boldareva and Gorlen	(Grant and Jones, 2016)
Roseinatronobacter monicus	Alphaproteobacteria	Mono Lake, CA, USA	(Grant and Jones, 2016)
Roseinatronobacter monicus	Alphaproteobacteria	Mono Lake, CA, USA	(Grant and Jones, 2016)
Roseinatronobacter thiooxidans	Alphaproteobacteria	Soda lake, Kunkuskaya Steppe, Russia	(Grant and Jones, 2016)
Roseococcus suduntuyensis	Alphaproteobacteria	Lake Shuluutai, Russia	(Grant and Jones, 2016)
Rubribacterium polymorphum	Alphaproteobacteria	Soda lake, Barguzin, Russia	(Grant and Jones, 2016)
Ruminiclostridium alkalicellulosi	Firmicutes	Lake Verkhnee Beloe, Russia	(Grant and Jones, 2016)
Salinicoccus alkaliphilus	Firmicutes	Baer Lake, Inner Mongolia	(Grant and Jones, 2016)
Salinicoccus halitifaciens	Firmicutes	Lonar Lake, India	(Grant and Jones, 2016)
Salinicoccus kekensis	Firmicutes	Lake Xiaokule, China	(Grant and Jones, 2016)
Salisediminibacterium haloalkalitolerans	Firmicutes	Lonar Lake, India	(Grant and Jones, 2016)
Salisediminibacterium halotolerans	Firmicutes	Xiarinaoer Lake, Inner Mongolia	(Grant and Jones, 2016)
Salsuginibacillus halophilus	Firmicutes	Xiarinaoer Lake, Inner Mongolia	(Grant and Jones, 2016)
Salsuginibacillus kocurii	Firmicutes	Lake Chagannor, Inner Mongolia	(Grant and Jones, 2016)

Spiribacter halalkaliphilus	*Gammaproteobacteria*	the soda-saline lakes in Inner Mongolia, China.	This study
Spirochaeta africana	Spirochaeta	Lake Magadi, Kenya	(Grant and Jones, 2016)
Spirochaeta alkalica	Spirochaeta	Lake Magadi, Kenya	(Grant and Jones, 2016)
Spirochaeta americana	Spirochaeta	Mono Lake, CA, USA	(Grant and Jones, 2016)
Spirochaeta asiatica	Spirochaeta	Lake Khatyn, Russia	(Grant and Jones, 2016)
Spirochaeta dissipatitropha	Spirochaeta	Searles Lake, CA, USA	(Grant and Jones, 2016)
Spirochaeta sphaeroplastigenens	Spirochaeta	Lonar Lake, India	(Grant and Jones, 2016)
Streptomyces alkaliphilus	Actinobacteriota	Lake Elmenteita, Kenya	(Grant and Jones, 2016)
Streptomyces alkalithermotolerans	Actinobacteriota	Lonar Lake, India	(Grant and Jones, 2016)
Sulfurospirillum alkalitolerans	Epsilonproteobacteria	Mixed sample, bioreactor	(Grant and Jones, 2016)
Szabonella alba	Alphaproteobacteria	a soda lake Neusiedler See, Hungary	This study
Tabrizicola alkalilacus	Alphaproteobacteria	the alkaline Lake Dajiaco on the Tibetan Plateau	This study
Texcoconibacillus texcoconensis	Firmicutes	Lake Texcoco, Mexico	(Grant and Jones, 2016)
Thioalkalibacter halophilus	Gammaproteobacteria	Soda lakes, Kulunda Steppe, Siberia	(Grant and Jones, 2016)
Thioalkalicoccus limnaeus	Gammaproteobacteria	Soda lakes, Russia	(Grant and Jones, 2016)
Thioalkalimicrobium aerophilum	Gammaproteobacteria	Soda lakes, Siberia and Kenya	(Grant and Jones, 2016)
Thioalkalimicrobium cyclicum	Gammaproteobacteria	Mono Lake, CA, USA	(Grant and Jones, 2016)
Thioalkalimicrobium microaerophilum	Gammaproteobacteria	Soap Lake, WA, USA	(Grant and Jones, 2016)
Thioalkalimicrobium sibericum	Gammaproteobacteria	Soda lakes, Siberia	(Grant and Jones, 2016)
Thioalkalispira microaerophila	Gammaproteobacteria	Wadi Natrun, Egypt	(Grant and Jones, 2016)
Thioalkalivibrio denitrificans	Gammaproteobacteria	Lake Bogoria, Kenya	(Grant and Jones, 2016)
Thioalkalivibrio halophilus	Gammaproteobacteria	Soda lake, Altai Steppe, Russia	(Grant and Jones, 2016)
Thioalkalivibrio jannaschii	Gammaproteobacteria	Mono Lake, CA, USA	(Grant and Jones, 2016)
Thioalkalivibrio nitratireducens	Gammaproteobacteria	Wadi Natrun, Egypt	(Grant and Jones, 2016)
Thioalkalivibrio nitratis	Gammaproteobacteria	Lake Nakuru, Kenya	(Grant and Jones, 2016)
Thioalkalivibrio nitratis ALJ2	*Gammaproteobacteria*	an East African Rift Valley soda lake	This study
Thioalkalivibrio paradoxus	Gammaproteobacteria	Mixed samples, Kenya and Egypt	(Grant and Jones, 2016)
Thioalkalivibrio sulfidiphilus	Gammaproteobacteria	Lake Elmenteita, Kenya	(Grant and Jones, 2016)
Thioalkalivibrio thiocyanodenitrificans	Gammaproteobacteria	Mixed samples, Russia, Egypt	(Grant and Jones, 2016)
Thioalkalivibrio thiocyanoxidans	Gammaproteobacteria	Mixed samples, Siberia, Kenya, Egypt	(Grant and Jones, 2016)
Thioalkalivibrio versutus	Gammaproteobacteria	Lake Bogoria, Kenya; soda lakes, Siberia	(Grant and Jones, 2016)
Thioalkalivibrio versutus AL2	*Gammaproteobacteria*	a soda lake in southeast Siberia	This study
Thiocapsa imhoffii	Gammaproteobacteria	Soap Lake, WA, USA	(Grant and Jones, 2016)
Thiohalophilus thiocyanoxidans	Gammaproteobacteria	Soda lake, Kulunda, Russia	(Grant and Jones, 2016)
Thiorhodospira sibirica	Gammaproteobacteria	Lake Malye Kasytui, Russia	(Grant and Jones, 2016)
Tindallia californiensis	Firmicutes	Mono Lake, CA, USA	(Grant and Jones, 2016)
Tindallia magadiensis	Firmicutes	Lake Magadi, Kenya	(Grant and Jones, 2016)
Tindallia texcoconensis	Firmicutes	Lake Texcoco, Mexico	(Grant and Jones, 2016)

Table S3. The statistics of the metagenome-assembled genomes (MAGs) reconstructed from soda lakes.

mag_id	representative	completeness	contamination	genome_size	no_contig	contig_n50	GC	coding_density	gene_count	gtdb_taxonomy
AF02MAG001	Yes	86.88	2.48	2983659	271	20080	58	87.72	2962	d Bacteria;p Desulfobacterota I;c Desulfovibrionia;o Desulfovibrionales;f Desulfonatronaceae;g Desulfonatronum;s
AF02MAG002	Yes	97.51	0.88	4466963	427	16737	38	84.21	4305	d Bacteria;p Cyanobacteria;c Cyanobacteriia;o Cyanobacteriales;f Microcystaceae;g ;s
AF02MAG003	Yes	92.75	3	4084402	547	11727	59.1	83.23	4317	d Bacteria;p Proteobacteria;c Gammaproteobacteria;o HK1;f JABDQX01;g JAGTVB01;s
AF02MAG004	Yes	90.35	3	3353960	532	9105	66	87.88	3583	d Bacteria;p Proteobacteria;c Gammaproteobacteria;o Ectothiorhodospirales;f Thioalkalivibrionaceae;g Thioalkalivibrio B;s
AF02MAG005	Yes	93.23	2.58	3562738	470	10665	57.6	88.85	3519	d Bacteria;p Desulfobacterota;c Desulfobacteria;o Desulfobacterales;f Desulfosarcinaceae;g Desulfosarcina;s
AF02MAG006	No	82.66	4.26	3269365	890	4309	63.5	90.51	3834	d Bacteria;p Proteobacteria;c Alphaproteobacteria;o Rhodobacterales;f Rhodobacteraceae;g Roseinatronobacter;s
AF02MAG007	Yes	92.21	3.1	3244215	652	6483	58.6	90.91	3496	d Bacteria;p Chloroflexota;c Chloroflexia;o Thermomicrobiales;f CFX8;g JACCZH01;s
AF02MAG008	Yes	70.17	1.59	2183499	536	4812	68	89.42	2803	d Bacteria;p Cyanobacteria;c Cyanobacteriia;o PCC-6307;f Cyanobiaceae;g NIES-981;s
AF02MAG009	Yes	73.76	6.57	1480306	515	3027	37	88.88	1715	d Bacteria;p Actinobacteriota;c Humimicrobiia;o Humimicrobiales;f Humimicrobiaceae;g ;s
AF02MAG010	Yes	96.46	3.18	6630836	783	12522	60	89.5	6283	d Bacteria;p Chloroflexota;c Anaerolineae;o Caldilineales;f Caldilineaceae;g Caldilinea;s
AF02MAG011	Yes	70.67	6.12	4021649	1297	3279	55.6	88.01	4259	d Bacteria;p Planctomycetota;c Planctomycetia;o Pirellulales;f Pirellulaceae;g ;s
AF02MAG012	Yes	83.65	1.7	3301054	880	4400	64.6	89.83	3867	d Bacteria;p Actinobacteriota;c Actinomycetia;o Nanopelagicales;f UBA10799;g ;s
AF02MAG019	Yes	77.11	4.27	3059868	795	4441	56.2	90.82	3354	d Bacteria;p Chloroflexota;c Anaerolineae;o Promineofilales;f Promineofilaceae;g ;s
AF02MAG052	Yes	73.88	9.66	3274610	927	3903	45.7	88.07	3667	d Bacteria;p Firmicutes D;c Dethiobacteria;o DTU022;f DTU022;g ;s
AF03MAG001	Yes	89.19	3.06	2690579	691	4805	53.7	92.76	3075	d Bacteria;p Firmicutes C;c Negativicutes;o UBA1444;f UBA1444;g UBA1444;s
AF03MAG002	Yes	90.29	1.71	6782125	597	15591	55.5	91.43	5710	d Bacteria;p Acidobacteriota;c Blastocatellia;o Pyrinomonadales;f Pyrinomonadaceae;g PSRF01;s
AF05MAG001	Yes	96.09	3.35	4780097	99	94778	30.6	89.56	4071	d Bacteria;p Bacteroidota;c Ignavibacteria;o Ignavibacteriales;f Melioribacteraceae;g GCA-2746605;s
AF05MAG002	Yes	97.24	2.38	3398468	139	44612	53.2	86.6	3022	d Bacteria;p Desulfobacterota;c Desulfobulbia;o Desulfobulbales;f Desulfobulbaceae;g Desulfobulbus;s
AF05MAG003	Yes	81.86	1.82	3107326	177	26372	63.3	92.77	2915	d Bacteria;p Proteobacteria;c Gammaproteobacteria;o Xanthomonadales;f Wenzhouxiangellaceae;g Wenzhouxiangella;s
AF05MAG004	Yes	98.05	3.22	4634464	460	14685	48.6	82.57	4230	d Bacteria;p Proteobacteria;c Gammaproteobacteria;o Methylococcales;f Methylomonadaceae;g Methylotuvimicrobium;s
AF05MAG005	Yes	89.7	2.36	1651173	116	25462	34.7	91.61	1709	d Bacteria;p Firmicutes;c Bacilli;o Erysipelotrichales;f Erysipelotrichaceae;g UBA6182;s
AF05MAG006	Yes	98.26	1.3	3681298	124	51077	62	91.14	3649	d Bacteria;p Proteobacteria;c Alphaproteobacteria;o Rhizobiales;f Aestuariivirgaceae;g Anderseniella;s
AF05MAG007	Yes	91.45	2.33	3015054	151	31384	67.1	89.88	2853	d Bacteria;p Proteobacteria;c Alphaproteobacteria;o Caulobacterales;f Hyphomonadaceae;g ;s
AF05MAG008	Yes	96.21	1.57	4421260	422	19421	39.1	88.81	4127	d Bacteria;p Bacteroidota;c Bacteroidia;o Cytophagales;f Cyclobacteriaceae;g Cecembia;s
AF05MAG009	Yes	92.6	0	2108101	112	26235	58.9	86.89	2111	d Archaea;p Halobacteriota;c Methanosarcinia;o Methanotrichales;f Methanotrichaceae;g Methanothrix A;s
AF05MAG010	Yes	96.96	1.53	3023655	139	44391	71.1	92.45	2970	d Bacteria;p Actinobacteriota;c Actinomycetia;o Jiangellales;f ;g ;s
AF05MAG011	Yes	94.89	0	3289334	201	26260	69.6	90.16	2758	d Bacteria;p Planctomycetota;c Phycisphaerae;o Phycisphaerales;f UBA1924;g ;s
AF05MAG012	Yes	91.09	1.98	3644076	376	15504	39	87.35	3049	d Bacteria;p Bacteroidota;c Bacteroidia;o Chitinophagales;f Saprospiraceae;g JADJGA01;s
AF05MAG013	Yes	99.53	2.34	2011770	108	48080	42.3	93.23	2120	d Bacteria;p Firmicutes;c Bacilli;o Erysipelotrichales;f Erysipelotrichaceae;g UBA2227;s
AF05MAG014	Yes	96.68	2.15	4271537	312	23589	48	90.48	3502	d Bacteria;p Bacteroidota;c Bacteroidia;o Bacteroidales;f UBA7960;g SKYG01;s
AF05MAG015	Yes	92.86	3.89	2965874	337	11986	45.4	92.73	2644	d Bacteria;p Bacteroidota;c Bacteroidia;o Bacteroidales;f VadinHA17;g SLNP01;s
AF05MAG016	Yes	90.86	3.86	3839802	180	39065	42.4	90.35	3132	d Bacteria;p Bacteroidota;c Bacteroidia;o Bacteroidales;f JAAYJT01;g JAAYJT01;s
AF05MAG017	Yes	93.01	2.17	4612936	353	25874	41.5	90.76	4044	d Bacteria;p Bacteroidota;c Bacteroidia;o Bacteroidales;f Prolixibacteraceae;g UBA1413;s
AF05MAG018	Yes	92.97	1.5	3089401	89	60830	62.8	89.53	2883	d Bacteria;p Proteobacteria;c Alphaproteobacteria;o Rhizobiales;f Rhizobiaceae;g Salaquimonas;s
AF05MAG019	Yes	93.18	8.74	4054560	632	9117	55.4	90.78	4163	d Bacteria;p Chloroflexota;c Anaerolineae;o Anaerolineales;f EnvOPS12;g UBA8950;s
AF05MAG021	Yes	95.6	1.1	3834067	222	28179	69.4	92.35	3277	d Bacteria;p Krumholzibacteriota;c Krumholzibacteria;o LZORAL124-64-63;f LZORAL124-64-63;g ;s
AF05MAG022	Yes	88.98	2.58	3457116	835	4901	63.9	88.49	3623	d Bacteria;p Desulfobacterota;c Desulfobacteria;o Desulfobacterales;f JAABRJ01;g JAABRJ01;s
AF05MAG024	Yes	92.73	3.02	3305824	187	24489	68.2	90.94	3342	d Bacteria;p Proteobacteria;c Alphaproteobacteria;o Rhodobacterales;f Rhodobacteraceae;g Muriiphilus;s
AF05MAG025	Yes	97.47	1.21	3827851	265	25704	48.1	90.4	3506	d Bacteria;p Bacteroidota;c Bacteroidia;o Flavobacteriales;f Flavobacteriaceae;g Maribacter;s
AF05MAG026	Yes	86.07	4.23	3798704	607	8553	62.2	89.45	4037	d Bacteria;p Proteobacteria;c Gammaproteobacteria;o Competibacterales;f Competibacteraceae;g REEE01;s
AF05MAG027	Yes	79.29	3.15	3190129	878	4120	48.1	88.23	3377	d Bacteria;p Bdellovibrionota;c Bdellovibrionia;o Bdellovibrionales;f Bdellovibrionaceae;g ;s
AF05MAG028	Yes	90.11	5.66	4653812	676	9495	69.6	91.06	4373	d Bacteria;p Gemmatimonadota;c Gemmatimonadetes;o Longimicrobiales;f UBA6960;g ;s
AF05MAG029	Yes	92.71	2.27	2912779	182	27288	62.8	91.11	2605	d Bacteria;p Planctomycetota;c Phycisphaerae;o Phycisphaerales;f UBA1924;g REDD01;s
AF05MAG030	Yes	83.49	6.65	2984409	733	4905	38.2	90.62	2937	d Bacteria;p Bacteroidota;c Bacteroidia;o Bacteroidales;f Tenuifilaceae;g UBA5261;s
AF05MAG031	Yes	84.06	2.99	2571038	575	5457	36.9	92.38	2712	d Bacteria;p Bacteroidota;c Bacteroidia;o Flavobacteriales;f Flavobacteriaceae;g Paucihalobacter;s
AF05MAG033	Yes	73.04	5.18	4055856	1356	3226	65.8	89.08	4550	d Bacteria;p Chloroflexota;c Anaerolineae;o JAAEKA01;f JAAEKA01;g JAAEKA01;s
AF05MAG035	Yes	91.84	2.2	3247089	374	11893	47.3	86.95	3049	d Bacteria;p Bdellovibrionota C;c UBA2361;o UBA2361;f JAAYZI01;g ;s
AF05MAG036	Yes	75.55	8.9	2922872	679	5015	51.3	91.86	3128	d Bacteria;p Chloroflexota;c Anaerolineae;o Anaerolineales;f Anaerolineaceae;g SpSt-1165;s
AF05MAG037	Yes	78.25	7.83	2108690	284	10008	47.1	92.46	2203	d Bacteria;p Chloroflexota;c Anaerolineae;o Anaerolineales;f ;g ;s
AF05MAG039	Yes	83.51	3.41	2571014	655	4503	68.1	91.34	2738	d Bacteria;p Planctomycetota;c Phycisphaerae;o Phycisphaerales;f UBA1924;g SLFH01;s
AF05MAG040	Yes	79.77	3.65	3416904	799	5191	64.2	85.31	3198	d Bacteria;p Planctomycetota;c Planctomycetia;o Pirellulales;f Thermoguttaceae;g ;s
AF05MAG041	Yes	82.27	3.64	5003121	1299	4517	62.3	89.25	4790	d Bacteria;p Chloroflexota;c Anaerolineae;o Promineofilales;f Promineofilaceae;g WTJY01;s
AF05MAG042	Yes	82.99	6.93	6654033	762	10688	62	90.64	5931	d Bacteria;p Verrucomicrobiota;c Verrucomicrobiae;o Pedosphaerales;f ;g ;s
AF05MAG046	Yes	76.58	3.01	3488035	948	4169	63	91.38	3361	d Bacteria;p Bacteroidota;c SZUA-365;o SZUA-365;f SZUA-365;g SZUA-365;s
AF05MAG047	Yes	87.21	6.06	3338184	700	5582	65.1	91.59	3784	d Bacteria;p Proteobacteria;c Alphaproteobacteria;o Rhodobacterales;f Rhodobacteraceae;g EhC02;s
AF05MAG053	Yes	79.32	5.72	4613365	1385	3646	66.7	91.2	5618	d Bacteria;p Actinobacteriota;c Acidimicrobiia;o UBA5794;f ;g ;s
AF05MAG054	Yes	71.78	2.93	2467795	754	3562	68.4	93.02	2946	d Bacteria;p Proteobacteria;c Gammaproteobacteria;o Burkholderiales;f Burkholderiaceae;g Hydrogenophaga;s Hydrogenophaga sp006227825
AF05MAG055	Yes	85.22	6	4546010	325	22882	66.9	89.88	4698	d Bacteria;p Proteobacteria;c Alphaproteobacteria;o Rhodobacterales;f Rhodobacteraceae;g IMCC34051;s
AF08MAG001	No	89.88	1.96	2264533	138	34789	47.4	86.74	2221	d Bacteria;p Desulfobacterota I;c Desulfovibrionia;o Desulfovibrionales;f Desulfonatronovibrionaceae;g Desulfonatronovibrio;s Desulfonatronovibrio sp003566455
AF08MAG002	No	94.87	2.66	2278978	201	16856	63.4	91.39	2269	d Bacteria;p Actinobacteriota;c Acidimicrobiia;o Acidimicrobiales;f UBA8139;g M30B73;s
AF08MAG003	Yes	87.92	3.6	2366881	141	45466	52.9	84.32	2266	d Bacteria;p Firmicutes D;c Dethiobacteria;o DTU022;f UBA8154;g CSSED11-181;s
AF08MAG005	Yes	96.47	1.54	2323763	234	15898	70	91.44	2595	d Bacteria;p Cyanobacteria;c Cyanobacteriia;o PCC-6307;f Cyanobiaceae;g NIES-981;s
AF08MAG006	No	82.91	0.43	2074292	124	27840	71.7	89.69	1936	d Bacteria;p Actinobacteriota;c Actinomycetia;o Euzebyales;f Egibacteraceae;g T1Sed10-49;s
AF08MAG007	Yes	83.92	3.5	2204848	327	9154	56.9	92.43	2197	d Bacteria;p Firmicutes A;c Clostridia;o Saccharofermentanales;f UBA5734;g SLHU01;s
AF08MAG008	Yes	70.38	1.72	565502	83	9057	36.5	93.42	609	d Bacteria;p Patescibacteria;c Dojkabacteria;o SC72;f SC72;g UBA2177;s
AF08MAG009	Yes	93.9	3.44	2719267	567	5642	55.9	86.85	2785	d Bacteria;p Desulfobacterota;c Syntrophobacteria;o Syntrophobacterales;f Syntrophobacteraceae;g SLCH01;s
AF08MAG010	Yes	88.43	4.69	3297442	471	10636	65.9	87.65	3469	d Bacteria;p Proteobacteria;c Gammaproteobacteria;o Ectothiorhodospirales;f Thioalkalivibrionaceae;g Thioalkalivibrio B;s Thioalkalivibrio B sp007119005
AF08MAG011	Yes	94.63	4.6	2811064	340	12279	66.7	88.55	2824	d Bacteria;p Proteobacteria;c Gammaproteobacteria;o Ectothiorhodospirales;f Thioalkalivibrionaceae;g Thioalkalivibrio B;s
AF08MAG012	Yes	70.39	0.42	1295800	267	6103	66.9	92.63	1506	d Bacteria;p Actinobacteriota;c Coriobacteriia;o OPB41;f SLCP01;g ;s
AF08MAG013	Yes	82.81	8.58	2084754	377	7000	45.5	86.68	2236	d Bacteria;p Firmicutes D;c Dethiobacteria;o DTU022;f UBA8154;g SLJK01;s
AF08MAG014	Yes	79	9.75	1712185	301	6370	53.7	87.85	1815	d Bacteria;p Firmicutes D;c Dethiobacteria;o DTU022;f UBA8154;g SKMY01;s
AF08MAG017	No	80.76	0.87	4697848	580	9897	60.9	89.44	3917	d Bacteria;p Planctomycetota;c Planctomycetia;o Pirellulales;f Pirellulaceae;g UBA6163;s UBA6163 sp007127095
AF08MAG024	Yes	77.23	2.1	2311550	792	3012	37.1	88.76	2642	d Bacteria;p Firmicutes D;c Dethiobacteria;o PWVN01;f ;g ;s
AF08MAG026	Yes	72.48	6.23	1365878	352	4753	42.1	89.31	1623	d Bacteria;p Firmicutes D;c Dethiobacteria;o SKNC01;f SKNC01;g SKLX01;s
AF08MAG027	Yes	73.29	5.9	2618235	894	3094	44.6	88.5	3023	d Bacteria;p Desulfobacterota;c Desulfobacteria;o Desulfobacterales;f SKZT01;g SKZT01;s
AF08MAG029	Yes	73.35	6.5	1395563	224	9233	50.8	89.08	1422	d Bacteria;p Firmicutes D;c Dethiobacteria;o DTU022;f DTU022;g B1SED10-74M;s
AF08MAG031	No	70.47	5.72	2216378	779	2960	49.6	91.65	2664	d Bacteria;p Firmicutes F;c Halanaerobiia;o Halanaerobiales;f Halarsenatibacteraceae;g Halarsenatibacter;s
AF08MAG039	Yes	82.57	4.6	3452358	712	6007	65.7	89.2	3677	d Bacteria;p Proteobacteria;c Alphaproteobacteria;o Rhizobiales;f Beijerinckiaceae;g Salinarimonas;s
AF08MAG048	Yes	84.75	8.67	2951630	403	12152	61.8	91.04	3091	d Bacteria;p Proteobacteria;c Alphaproteobacteria;o Rhodobacterales;f Rhodobacteraceae;g Roseinatronobacter;s
AF08MAG062	No	75.77	4.17	2402044	627	4469	44.2	83.76	2439	d Bacteria;p Firmicutes F;c Halanaerobiia;o Halanaerobiales;f Halarsenatibacteraceae;g T1SED10-84;s
AF09MAG001	Yes	97.44	3.85	3392081	115	54495	63.7	92.64	3475	d Bacteria;p Actinobacteriota;c Acidimicrobiia;o UBA5794;f ZC4RG35;g SZUA-217;s
AF09MAG002	Yes	87.54	1.34	2948257	456	8664	44.3	90.59	2790	d Bacteria;p Bacteroidota;c Bacteroidia;o Bacteroidales;f UBA7960;g UBA7960;s
AF09MAG003	Yes	85.88	7.9	2954160	627	5659	65.7	88.28	3300	d Bacteria;p Proteobacteria;c Gammaproteobacteria;o Ectothiorhodospirales;f Thioalkalivibrionaceae;g Thioalkalivibrio B;s Thioalkalivibrio B sp003550945
AF09MAG004	Yes	89.36	2.29	2002631	216	14905	65.8	93.32	2216	d Bacteria;p Actinobacteriota;c Acidimicrobiia;o UBA5794;f ZC4RG35;g ZC4RG35;s
AF09MAG005	Yes	90.85	1.97	3976130	554	10140	40.1	89.2	3934	d Bacteria;p Bacteroidota;c Bacteroidia;o Cytophagales;f Cyclobacteriaceae;g Mariniradius;s
AF09MAG007	Yes	82.87	5.88	5806043	1023	6681	52.3	88	5357	d Bacteria;p Planctomycetota;c Planctomycetia;o Pirellulales;f Pirellulaceae;g Aureliella;s

AF09MAG008	Yes	70.77	2.12	3542726	1087	3612	67.9	89.97	4290	d Bacteria;p Proteobacteria;c Alphaproteobacteria;o Geminicoccales;f Geminicoccaceae;g SLRI01;s
AF09MAG010	Yes	85.65	1.79	3109695	412	10265	61.2	87.35	2981	d Bacteria;p Verrucomicrobiota;c Verrucomicrobiae;o Verrucomicrobiales;f SLCJ01;g SLCJ01;s
AF09MAG013	Yes	81.19	2.82	2667336	522	6277	63.3	90.58	2583	d Bacteria;p Verrucomicrobiota;c Kiritimatiellae;o LD1-PB3;f Lenti-01;g ;s
AF09MAG014	Yes	75.36	4.82	2292828	677	3765	68.9	91.76	2774	d Bacteria;p Chloroflexota;c Limnocylindria;o Limnocylindrales;f CSP1-4;g ;s
AF09MAG016	No	86.01	3.33	2868278	206	27201	59	90.85	2929	d Bacteria;p Proteobacteria;c Alphaproteobacteria;o Rhodobacterales;f Rhodobacteraceae;g Roseinatronobacter;s Roseinatronobacter sp007125005
AF10MAG001	Yes	95.54	5.49	5477765	145	97032	47.8	91.18	4336	d Bacteria;p KSB1;c UBA2214;o UBA2214;f Zgenome-0027;g Zgenome-0027;s
AF10MAG002	Yes	99.67	3.01	1813679	91	36162	54	89.26	1953	d Archaea;p Halobacteriota;c Methanomicrobia;o Methanomicrobiales;f Methanoregulaceae;g MVRE01;s
AF10MAG003	Yes	94.55	6.06	3834241	255	24061	57.1	91.29	3574	d Bacteria;p Chloroflexota;c Anaerolineae;o Anaerolineales;f Anaerolineaceae;g Ch10;s
AF10MAG004	Yes	93.93	2.88	3241379	132	53920	41.5	90.47	3176	d Archaea;p Asgardarchaeota;c Thorarchaeia;o Thorarchaeales;f Thorarchaeaceae;g MP8T-1;s
AF10MAG005	Yes	88.49	0.71	1787401	313	7859	60.4	87.57	2020	d Archaea;p Halobacteriota;c Methanosarcinia;o Methanotrichales;f Methanotrichaceae;g Methanothrix A;s Methanothrix A harundinacea B
AF10MAG006	Yes	99.84	1.61	2077158	81	68593	63.7	90.93	2107	d Archaea;p Thermoplasmatota;c Thermoplasmata;o Methanomassiliicoccales;f UBA472;g ;s
AF10MAG007	Yes	89.83	0	1839220	59	57016	65.2	89.43	1743	d Bacteria;p Bipolaricaulota;c Bipolaricaulia;o Bipolaricaulales;f Bipolaricaulaceae;g Bipolaricaulis;s
AF10MAG008	Yes	96.86	3.02	7010504	392	29958	58.2	84.4	6236	d Bacteria;p Chloroflexota;c Chloroflexia;o Chloroflexales;f Chloroflexaceae;g ;s
AF10MAG009	Yes	92.24	1.1	6886235	760	16635	46.5	83.81	5576	d Bacteria;p KSB1;c UBA2214;o ;f ;g ;s
AF10MAG010	Yes	93.09	3.43	4277707	510	13598	61.8	84.21	4334	d Bacteria;p Proteobacteria;c Gammaproteobacteria;o Methylococcales;f Methylococcaceae;g Methylotetracoccus;s
AF10MAG011	Yes	93.41	3.85	2553855	363	9666	66.8	92.23	2444	d Bacteria;p Krumholzibacteriota;c Krumholzibacteria;o Krumholzibacteriales;f Krumholzibacteriaceae;g SSS58A;s
AF10MAG012	Yes	84.4	4	2448548	507	6126	31.2	89.42	2628	d Archaea;p Thermoplasmatota;c E2;o DHVEG-1;f DHVEG-1;g SG8-52-3;s
AF10MAG013	Yes	83.66	1.96	1558226	378	4683	51.3	90.25	1815	d Archaea;p Halobacteriota;c Methanosarcinia;o Methanotrichales;f Methanotrichaceae;g ;s
AF10MAG014	Yes	96.77	3.59	2709369	84	58710	58	89.02	2585	d Bacteria;p Desulfobacterota;c Syntrophia;o Syntrophales;f Fen-1087;g ;s
AF10MAG015	Yes	89.01	2.2	4465583	476	13429	49.5	91.83	3937	d Bacteria;p KSB1;c UBA2214;o UBA2214;f ;g ;s
AF10MAG016	Yes	73.48	3.01	3640500	978	4422	65.1	87.21	4084	d Bacteria;p Proteobacteria;c Gammaproteobacteria;o Burkholderiales;f Rhodocyclaceae;g CG2-30-68-42;s
AF10MAG017	Yes	89.87	2.61	1909576	128	22648	58.6	87.15	2032	d Archaea;p Halobacteriota;c Syntropharchaeia;o ANME-1;f ANME-1;g JACGMN01;s
AF10MAG018	Yes	78.46	0.09	3537281	897	4566	66.5	88.6	3748	d Bacteria;p Chloroflexota;c Chloroflexia;o Chloroflexales;f Chloroflexaceae;g JACAEO01;s
AF10MAG020	Yes	91.1	8.06	1913166	156	25768	60.8	86.29	2000	d Archaea;p Halobacteriota;c Methanomicrobia;o Methanomicrobiales;f Methanoregulaceae;g UBA9949;s
AF10MAG021	Yes	85.45	1.55	7247777	607	15596	58.7	88.94	6810	d Bacteria;p Chloroflexota;c Anaerolineae;o 4572-78;f NAK82;g ;s
AF10MAG023	Yes	89.55	3.09	2357573	456	6941	57.6	90.12	2605	d Bacteria;p Nitrospirota;c Thermodesulfovibrionia;o Thermodesulfovibrionales;f Dissulfurispiraceae;g PNNP01;s
AF10MAG024	Yes	74.69	4.12	2746426	761	4047	61.6	91.93	2950	d Bacteria;p Bacteroidota;c UBA10030;o UBA10030;f UBA10030;g ;s
AF10MAG025	Yes	83.63	4.95	2160266	300	9229	51.2	87.19	2251	d Bacteria;p Chloroflexota;c Dehalococcoidia;o SZUA-161;f SZUA-161;g JACWAE01;s
AF10MAG026	Yes	95.21	3.03	4362753	519	11289	57.1	89.41	3893	d Bacteria;p Chloroflexota;c Anaerolineae;o Promineofilales;f Promineofilaceae;g JACSRF01;s
AF10MAG028	Yes	73.07	1.28	2424651	638	4345	70.4	92.59	2881	d Bacteria;p Actinobacteriota;c Acidimicrobiia;o UBA5794;f S012-128;g ;s
AF10MAG030	Yes	85.45	3.94	4729144	782	8296	62.3	89.37	4768	d Bacteria;p Chloroflexota;c Anaerolineae;o Promineofilales;f Promineofilaceae;g JAACFF01;s
AF10MAG033	Yes	78.14	1.57	1142657	403	2920	52.4	88.53	1395	d Bacteria;p Chloroflexota;c Dehalococcoidia;o Dehalococcoidales;f Fen-1064;g ;s
AF10MAG040	Yes	74.97	1.1	2816915	812	3833	55.5	87.64	2744	d Bacteria;p TA06 A;c DG-26 A;o ;f ;g ;s
AF10MAG042	Yes	85.48	9.17	3931983	462	12348	50	85.56	3992	d Bacteria;p Desulfobacterota;c BSN033;o BSN033;f UBA1163;g ;s
AF10MAG043	Yes	70.6	4.56	6527242	1714	4244	43.1	84.16	5241	d Bacteria;p KSB1;c UBA2214;o ;f ;g ;s
AF10MAG045	Yes	95.06	9.26	5169763	657	16903	58	85.15	5505	d Bacteria;p Proteobacteria;c Gammaproteobacteria;o Methylococcales;f Methylococcaceae;g ;s
AF10MAG046	Yes	96.6	2.64	4071015	745	7370	48.2	84.04	4110	d Bacteria;p Proteobacteria;c Gammaproteobacteria;o Methylococcales;f Methylomonadaceae;g Methylotuvimicrobium;s
AF10MAG055	Yes	87.1	7.74	3592583	219	43521	57.3	88.59	3361	d Bacteria;p Desulfobacterota;c DSM-4660;o Desulfatiglandales;f HGW-15;g JAFGDM01;s
AF10MAG057	Yes	75.82	1.1	3463057	372	12842	40.4	83.76	2807	d Bacteria;p KSB1;c UBA2214;o AABM5-25-91;f JAFGDW01;g ;s
AF20MAG001	Yes	87.95	2.62	1615000	117	26344	38.8	86.93	1620	d Bacteria;p Firmicutes;c Bacilli;o Culicoidibacterales;f ZOR0006;g ZOR0006;s
AF20MAG002	Yes	94.11	1.89	2215848	125	42769	59.1	94.31	2388	d Bacteria;p Actinobacteriota;c Actinomycetia;o Nanopelagicales;f S36-B12;g S36-B12;s
AF20MAG003	Yes	93.04	0	4854447	351	24766	48.5	87.67	4074	d Bacteria;p Planctomycetota;c Planctomycetia;o Pirellulales;f Pirellulaceae;g Mariniblastus;s
AF20MAG004	Yes	96.12	2.55	4039774	456	13244	63.8	89.91	3587	d Bacteria;p Verrucomicrobiota;c Verrucomicrobiae;o Verrucomicrobiales;f Akkermansiaceae;g Luteolibacter;s
AF20MAG005	Yes	82.87	0	4694373	182	50834	62.8	89.4	3518	d Bacteria;p Planctomycetota;c Planctomycetia;o Pirellulales;f Pirellulaceae;g UBA6163;s
AF20MAG006	Yes	89.66	2.04	3054835	261	18611	61.1	87.07	2889	d Bacteria;p Verrucomicrobiota;c Verrucomicrobiae;o Verrucomicrobiales;f SLCJ01;g SLCJ01;s
AF20MAG007	Yes	87.58	1.76	5647692	781	9574	51.5	89.07	4957	d Bacteria;p Planctomycetota;c Planctomycetia;o Pirellulales;f Pirellulaceae;g Pirellula B;s
AF20MAG008	Yes	77.72	2.38	4046828	1052	4534	66.1	88.72	4276	d Bacteria;p Verrucomicrobiota;c Verrucomicrobiae;o Verrucomicrobiales;f DEV007;g Arctic95D-9;s
AF20MAG009	Yes	93.61	2.3	3557821	486	10128	68.2	91.09	3897	d Bacteria;p Proteobacteria;c Alphaproteobacteria;o Rhodobacterales;f Rhodobacteraceae;g UBA996;s
AF20MAG010	No	83.34	6.91	2304652	504	5596	37.8	91.4	2549	d Bacteria;p Bacteroidota;c Bacteroidia;o Flavobacteriales;f Flavobacteriaceae;g Planktosalinus;s
AF20MAG011	Yes	93.56	2.79	3717875	643	7825	36	84.11	3808	d Bacteria;p Cyanobacteria;c Cyanobacteriia;o Cyanobacteriales;f Cyanobacteriaceae;g PCC-10605;s
AF20MAG012	Yes	75.89	1.28	4006653	712	7102	54.5	83.26	3344	d Bacteria;p Planctomycetota;c Planctomycetia;o Pirellulales;f Pirellulaceae;g Mariniblastus;s
AF20MAG016	Yes	83.6	2.52	4285384	591	8663	48	85.8	3949	d Bacteria;p Cyanobacteria;c Cyanobacteriia;o Cyanobacteriales;f Phormidiaceae A;g ;s
AF20MAG017	Yes	73.28	1.75	4749420	879	6869	65.1	83.98	5548	d Bacteria;p Actinobacteriota;c Rubrobacteria;o Rubrobacterales;f Rubrobacteraceae;g ;s
AF20MAG019	Yes	89.43	4.34	4808379	1324	4137	34.3	82.45	5579	d Bacteria;p Cyanobacteria;c Cyanobacteriia;o Cyanobacteriales;f Cyanobacteriaceae;g Geminocystis;s
AF20MAG021	Yes	81.51	4.15	6666055	1555	4835	47.8	83.28	6803	d Bacteria;p Cyanobacteria;c Cyanobacteriia;o Cyanobacteriales;f Coleofasciculaceae;g FACHB-SPT15;s
AF20MAG022	Yes	79.43	3.76	4054144	1196	3665	59.6	88.04	3760	d Bacteria;p Planctomycetota;c Planctomycetia;o Pirellulales;f Pirellulaceae;g M30B19;s
AF20MAG024	Yes	87.87	3.95	3197342	318	15382	63.6	90.53	3279	d Bacteria;p Proteobacteria;c Alphaproteobacteria;o Rhodobacterales;f Rhodobacteraceae;g Roseinatronobacter;s
AF20MAG030	Yes	81.28	4.04	3293629	398	14157	59.4	90.71	3522	d Bacteria;p Proteobacteria;c Alphaproteobacteria;o Rhodobacterales;f Rhodobacteraceae;g Roseinatronobacter;s Roseinatronobacter sp007117255
AF20MAG040	Yes	70.21	1.93	3485267	1149	3241	59.4	88.49	3748	d Bacteria;p Chloroflexota;c Chloroflexia;o 54-19;f JADMIH01;g JADMIH01;s
AF23MAG001	Yes	94.92	0.65	2834305	160	32735	39.7	90.36	2583	d Bacteria;p Bacteroidota;c Bacteroidia;o Cytophagales;f Cyclobacteriaceae;g Cecembia;s
AF23MAG002	Yes	87.66	1	2157356	170	25879	32.7	90.74	2180	d Bacteria;p Bacteroidota;c Bacteroidia;o Flavobacteriales;f Flavobacteriaceae;g Flavobacterium;s
AF23MAG003	Yes	92.72	1.94	2038168	139	24453	59.3	95.44	1981	d Bacteria;p Proteobacteria;c Gammaproteobacteria;o Xanthomonadales;f Wenzhouxiangellaceae;g Wenzhouxiangella;s
AF23MAG004	Yes	92.24	1.29	1755002	225	11388	72.2	94.44	2007	d Bacteria;p Actinobacteriota;c Thermoleophilia;o Miltoncostaeales;f Miltoncostaeaceae;g SYFI01;s
AF23MAG005	Yes	91.01	1.22	2048382	110	30411	58.9	93.64	2036	d Bacteria;p Proteobacteria;c Gammaproteobacteria;o Burkholderiales;f Burkholderiaceae;g UBA2463;s
AF23MAG006	Yes	95.79	1.09	2949593	205	23233	30.1	89.62	2650	d Bacteria;p Bacteroidota;c Kapabacteria;o Kapabacteriales;f UBA2268;g ;s
AF23MAG007	Yes	86.32	0.85	2299933	95	45054	64.1	94.81	2304	d Bacteria;p Actinobacteriota;c Acidimicrobiia;o Acidimicrobiales;f Ilumatobacteraceae;g Casp-actino8;s
AF23MAG008	Yes	87.07	0.08	1207379	90	21319	65.4	94.55	1282	d Bacteria;p Actinobacteriota;c Actinomycetia;o Actinomycetales;f Microbacteriaceae;g Pontimonas;s
AF23MAG009	Yes	81.33	0.67	843472	80	17352	40	94.28	913	d Bacteria;p Firmicutes;c Bacilli;o Izemoplasmatales;f Izemoplasmataceae;g CSBR16-87;s
AF23MAG010	No	77.99	2.5	1503622	240	12528	42.6	95.38	1738	d Bacteria;p Actinobacteriota;c Actinomycetia;o Nanopelagicales;f AcAMD-5;g ATZT02;s
AF23MAG011	Yes	76.08	0.93	1576701	392	4360	56.4	94.35	1773	d Bacteria;p Armatimonadota;c Fimbriimonadia;o Fimbriimonadales;f Fimbriimonadaceae;g UBA2387;s
AF23MAG012	Yes	96.12	2.87	2106171	139	34539	71.6	93.89	2156	d Bacteria;p Actinobacteriota;c Thermoleophilia;o Gaiellales;f F1-60-MAGs149;g ;s
AF23MAG013	Yes	90.06	7.39	2203169	140	34112	60.7	95.95	2333	d Bacteria;p Actinobacteriota;c Actinomycetia;o Nanopelagicales;f S36-B12;g ;s
AF23MAG014	Yes	85.61	0.82	2338254	181	19109	44.4	91.69	2136	d Bacteria;p Bacteroidota;c Rhodothermia;o Balneolales;f HLUCCA01;g UBA11400;s
AF23MAG015	No	82.62	2.03	2396791	414	7161	54.9	92.34	2646	d Bacteria;p Proteobacteria;c Gammaproteobacteria;o Burkholderiales;f Burkholderiaceae;g Algicoccus;s
AF23MAG016	Yes	95.73	4.7	2972456	186	35058	66.3	95.23	3100	d Bacteria;p Actinobacteriota;c Acidimicrobiia;o Acidimicrobiales;f Ilumatobacteraceae;g ;s
AF23MAG017	Yes	87.81	7.2	1998562	379	6718	57.7	93.62	2318	d Bacteria;p Proteobacteria;c Gammaproteobacteria;o Burkholderiales;f Burkholderiaceae;g ;s
AF23MAG018	Yes	78.34	0.9	1379694	144	19865	55.5	95.46	1475	d Bacteria;p Actinobacteriota;c Actinomycetia;o Nanopelagicales;f AcAMD-5;g VGAH01;s
AF23MAG019	Yes	93.33	1.28	1714941	74	60617	68.1	96.03	1765	d Bacteria;p Actinobacteriota;c Acidimicrobiia;o Acidimicrobiales;f Ilumatobacteraceae;g UBA3006;s
AF23MAG020	Yes	89.79	0.85	2178472	281	11110	70.5	94.17	2239	d Bacteria;p Deinococcota;c Deinococci;o Deinococcales;f Trueperaceae;g JAABTL01;s
AF23MAG021	Yes	71.55	0	539382	22	58878	43.1	91.1	604	d Bacteria;p Patescibacteria;c UBA1384;o 2-12-FULL-50-11;f 2-12-FULL-50-11;g BOKZ01;s
AF23MAG022	Yes	92.31	7.98	2194284	360	8647	63.4	94.82	2373	d Bacteria;p Actinobacteriota;c Acidimicrobiia;o Acidimicrobiales;f Ilumatobacteraceae;g CSBr16-110;s
AF23MAG023	Yes	75.78	3.45	1239297	309	4937	66.9	94.41	1441	d Bacteria;p Actinobacteriota;c Thermoleophilia;o Solirubrobacterales;f 70-9;g 67-14;s
AF23MAG024	Yes	72.26	1.72	827666	101	11852	36.1	92.23	948	d Bacteria;p Patescibacteria;c WWE3;o 2-01-FULL-39-13;f 2-01-FULL-39-13;g ;s
AF23MAG025	Yes	92.45	9.92	2963952	485	10230	57.3	92.39	3779	d Bacteria;p Actinobacteriota;c Actinomycetia;o Actinomycetales;f Microbacteriaceae;g Pontimonas;s
AF23MAG026	Yes	94.59	4.02	1713752	156	19725	60.7	95.43	1827	d Bacteria;p Actinobacteriota;c Acidimicrobiia;o Acidimicrobiales;f Ilumatobacteraceae;g F1-60-MAGs027;s
AF23MAG028	Yes	93.92	4.24	2106607	305	9133	65	90.92	2237	d Bacteria;p Verrucomicrobiota;c Verrucomicrobiae;o Chthoniobacterales;f JACTMZ01;g JACTMZ01;s
AF23MAG029	Yes	80.03	3.57	2200382	426	6498	67.3	94.38	2516	d Bacteria;p Actinobacteriota;c Actinomycetia;o Nanopelagicales;f S36-B12;g Mxb001;s
AF23MAG030	No	86.21	0	2768620	754	4277	71.1	89.18	2765	d Bacteria;p Planctomycetota;c Planctomycetia;o Pirellulales;f UBA1268;g QWPN01;s
AF23MAG031	Yes	84.09	0.77	1205868	102	24044	52.3	95.45	1307	d Bacteria;p Actinobacteriota;c Actinomycetia;o Actinomycetales;f Microbacteriaceae;g Rhodoluna;s
AF23MAG034	Yes	94.17	1.89	1028043	115	13175	49.8	94.88	1140	d Bacteria;p Firmicutes;c Bacilli;o Erysipelotrichales;f Erysipelotrichaceae;g UBA2212;s

AF23MAG035	Yes	84.25	4.99	2316102	536	5296	66.6	93.3	2609	d Bacteria;p Actinobacteriota;c Acidimicrobiia;o Acidimicrobiales;f Ilumatobacteraceae;g CSBr16-110;s
AF23MAG036	Yes	83.24	4.78	2435731	614	4661	47.4	90.43	2632	d Bacteria;p Bacteroidota;c Bacteroidia;o UBA7662;f UBA7662;g PNNG01;s
AF23MAG037	Yes	72.01	5.11	2005683	523	4230	61.6	94.67	2376	d Bacteria;p Proteobacteria;c Gammaproteobacteria;o Steroidobacterales;f Steroidobacteraceae;g UBA964;s
AF23MAG038	Yes	75.7	1.25	859623	99	12908	29.4	93.4	1123	d Archaea;p Nanoarchaeota;c Nanoarchaeia;o Woesearchaeales;f 21-14-0-10-32-9;g SKIS01;s
AF23MAG040	Yes	87.1	0.99	1797151	206	13360	64.1	94.39	1855	d Bacteria;p Deinococcota;c Deinococci;o Deinococcales;f Trueperaceae;g JAABTL01;s
AF23MAG046	Yes	71.64	0.85	1507925	328	5889	74.4	93.33	1740	d Bacteria;p Actinobacteriota;c Actinomycetia;o Nitriliruptorales;f Nitriliruptoraceae;g CSBr16-57R1;s
AF23MAG047	Yes	92.4	7.91	1737788	251	8653	74.3	92.98	1817	d Bacteria;p Actinobacteriota;c Actinomycetia;o Nitriliruptorales;f Nitriliruptoraceae;g ;s
AF23MAG049	Yes	71.97	2.46	2055697	559	4084	51.7	88.14	2150	d Bacteria;p Bacteroidota;c Rhodothermia;o Balneolales;f HLUCCA01;g HLUCCA01;s
AF23MAG051	Yes	73.97	3.57	2031856	469	5290	37.6	90.83	2454	d Bacteria;p Bacteroidota;c Bacteroidia;o Flavobacteriales;f Flavobacteriaceae;g Flavobacterium;s
AF23MAG054	Yes	83.32	1.62	1779267	113	28235	63	96.25	1871	d Bacteria;p Actinobacteriota;c Actinomycetia;o Nanopelagicales;f S36-B12;g ;s
AF24MAG001	Yes	93.58	3.49	2293810	98	49617	38	91.65	2233	d Bacteria;p Bacteroidota;c Bacteroidia;o Flavobacteriales;f Flavobacteriaceae;g Planktosalinus;s
AF24MAG002	Yes	93.37	0.81	2247476	126	28457	42.1	91.74	2064	d Bacteria;p Bacteroidota;c Bacteroidia;o Flavobacteriales;f Schleiferiaceae;g REDE01;s
AF24MAG003	No	96.45	0.82	2928929	104	46992	47.5	86.91	2547	d Bacteria;p Bacteroidota;c Rhodothermia;o Balneolales;f PXAI01;g Cyclonatronum;s
AF24MAG004	Yes	93.72	1.64	2422745	105	36530	46.6	89.91	2166	d Bacteria;p Bacteroidota;c Rhodothermia;o Balneolales;f Balneolaceae;g UBA2664;s
AF24MAG005	Yes	94.02	0.95	2224015	163	26404	65.8	93.65	2224	d Bacteria;p Actinobacteriota;c Acidimicrobiia;o Acidimicrobiales;f Ilumatobacteraceae;g CSBr16-110;s
AF24MAG006	No	96.58	1.28	1718275	112	39852	59.2	93.82	1754	d Bacteria;p Actinobacteriota;c Actinomycetia;o Nitriliruptorales;f Nitriliruptoraceae;g SLDB01;s
AF24MAG007	Yes	95.57	1.49	2845979	389	11630	53.8	90.44	2886	d Bacteria;p Verrucomicrobiota;c Verrucomicrobiae;o Opitutales;f DSM-45221;g SKFT01;s
AF24MAG008	Yes	84.85	2.32	2040800	312	8026	49.7	91.3	2035	d Bacteria;p Bacteroidota;c Rhodothermia;o Balneolales;f HLUCCA01;g HLUCCA01;s
AF24MAG009	Yes	94.87	2.56	2425727	168	42028	68.2	92.39	2363	d Bacteria;p Actinobacteriota;c Acidimicrobiia;o Acidimicrobiales;f UBA8139;g M30B73;s
AF24MAG010	Yes	84.41	0.42	1913162	118	29367	48.8	91.77	1852	d Bacteria;p Proteobacteria;c Gammaproteobacteria;o Enterobacterales;f Alteromonadaceae;g Aliidiomarina;s
AF24MAG012	Yes	94.87	4.7	2206727	187	21433	64.6	94.87	2313	d Bacteria;p Actinobacteriota;c Acidimicrobiia;o Acidimicrobiales;f Ilumatobacteraceae;g M30B72;s
AF24MAG013	Yes	94.78	4.37	2253667	179	22062	64.9	91.91	2207	d Bacteria;p Actinobacteriota;c Acidimicrobiia;o Acidimicrobiales;f UBA8139;g M30B73;s
AF24MAG014	Yes	93.5	7.11	3018156	297	24559	34.5	87.4	3289	d Bacteria;p Bacteroidota;c Bacteroidia;o Flavobacteriales;f Flavobacteriaceae;g Psychroflexus;s
AF24MAG015	Yes	83.12	2.66	2313834	289	12429	35.2	88.75	2277	d Bacteria;p Bacteroidota;c Bacteroidia;o Flavobacteriales;f Flavobacteriaceae;g Psychroflexus;s
AF24MAG016	Yes	74.67	5.33	892500	159	7881	40	94.15	1000	d Bacteria;p Firmicutes;c Bacilli;o Izemoplasmatales;f Izemoplasmataceae;g CSBR16-87;s
AF24MAG017	Yes	93.6	3.74	1782610	217	11760	50.1	92.02	1861	d Bacteria;p Proteobacteria;c Gammaproteobacteria;o Nitrosococcales;f Methylophagaceae;g Methylophaga;s
AF24MAG018	Yes	78.04	0.93	766944	65	20990	31.7	93.45	946	d Archaea;p Nanoarchaeota;c Nanoarchaeia;o Woesearchaeales;f 21-14-0-10-32-9;g SKIS01;s
AF24MAG019	Yes	92.89	3.54	2275293	150	22958	56.1	93.23	2260	d Bacteria;p Proteobacteria;c Gammaproteobacteria;o Burkholderiales;f Burkholderiaceae;g Algicoccus;s
AF24MAG021	No	84.59	9.63	3184978	811	4710	67.3	90.26	3561	d Bacteria;p Proteobacteria;c Alphaproteobacteria;o Rhodobacterales;f Rhodobacteraceae;g Pararhodobacter;s Pararhodobacter sp007117005
AF24MAG022	No	75.12	5.5	1631674	261	8158	58.9	94.7	1855	d Bacteria;p Actinobacteriota;c Actinomycetia;o Nanopelagicales;f S36-B12;g S36-B12;s
AF24MAG024	Yes	93.8	4.45	3996876	577	10941	61	90.55	4428	d Bacteria;p Proteobacteria;c Alphaproteobacteria;o Rhodobacterales;f Rhodobacteraceae;g Roseinatronobacter;s
AF24MAG025	Yes	71.97	6.55	1569597	327	6043	69.4	92.23	1728	d Bacteria;p Actinobacteriota;c Acidimicrobiia;o Acidimicrobiales;f UBA8139;g M30B73;s
AF24MAG026	No	85.23	8.03	2643786	691	4398	54.7	91.92	3149	d Bacteria;p Proteobacteria;c Gammaproteobacteria;o Burkholderiales;f Burkholderiaceae;g Algicoccus;s
AF24MAG034	No	77.2	2.62	3409323	1094	3359	60.7	93	3934	d Bacteria;p Actinobacteriota;c Actinomycetia;o Nanopelagicales;f S36-B12;g M55B157;s
AF25MAG001	Yes	97.85	0	2486452	17	192939	55.3	93.57	2388	d Bacteria;p Proteobacteria;c Alphaproteobacteria;o Rickettsiales;f SXRF01;g ;s
AF25MAG002	Yes	98.15	0.62	2856648	105	42756	64.2	90.51	2626	d Bacteria;p Proteobacteria;c Gammaproteobacteria;o Burkholderiales;f Burkholderiaceae;g UBA3064;s UBA3064 sp016868175
AF25MAG003	Yes	90.24	4.32	1715741	237	14593	58.8	95.37	1883	d Bacteria;p Proteobacteria;c Gammaproteobacteria;o Burkholderiales;f Burkholderiaceae;g UBA2463;s UBA2463 sp016868255
AF25MAG004	Yes	100	0.1	1569333	91	32430	53.3	89.98	1620	d Bacteria;p Proteobacteria;c Alphaproteobacteria;o ;f ;g ;s
AF25MAG005	No	80.31	0.34	2427240	40	97177	41.9	86.8	2281	d Bacteria;p Proteobacteria;c Gammaproteobacteria;o Pseudomonadales;f Moraxellaceae;g Acinetobacter;s Acinetobacter johnsonii
AF25MAG006	Yes	82.12	0.58	1404101	83	45316	54.5	94.24	1469	d Bacteria;p Proteobacteria;c Gammaproteobacteria;o Burkholderiales;f Rhodocyclaceae;g Fluviibacter;s
AF25MAG007	Yes	73.98	3.45	834084	37	44741	27.8	85.99	804	d Bacteria;p Patescibacteria;c WWE3;o UBA101185;f UBA10185;g ;s
AF25MAG009	Yes	96.45	0.82	2216103	81	50733	61.4	96.09	2065	d Bacteria;p Bacteroidota;c Rhodothermia;o Balneolales;f HLUCCA01;g ;s
AF25MAG010	Yes	87.01	2.22	2089389	233	13086	69.5	93.68	2145	d Bacteria;p Actinobacteriota;c Actinomycetia;o Nanopelagicales;f AcAMD-5;g REEB473;s REEB473 sp018969715
AF25MAG011	Yes	96.55	0.86	1894461	46	84526	72.6	93.98	1942	d Bacteria;p Actinobacteriota;c Thermoleophilia;o Miltoncostaeales;f Miltoncostaeaceae;g SYFI01;s
AF25MAG012	Yes	90.3	6.8	3327373	817	4928	48.1	91.03	3946	d Bacteria;p Firmicutes;c Bacilli;o Exiguobacterales;f Exiguobacteraceae;g Exiguobacterium A;s Exiguobacterium A indicum
AF25MAG014	Yes	75.55	1.72	983493	16	87368	33.4	90.82	941	d Bacteria;p Patescibacteria;c Microgenomatia;o UBA1406;f GWC2-37-13;g UBA1450;s
AF25MAG016	Yes	87.96	6.4	2215384	357	9026	59	94.32	2374	d Bacteria;p Proteobacteria;c Gammaproteobacteria;o Burkholderiales;f Burkholderiaceae;g UBA2463;s
AF25MAG017	Yes	97.65	0	2760143	40	146395	40.2	95.27	2660	d Bacteria;p Spirochaetota;c Leptospirae;o Leptospirales;f Leptospiraceae;g ;s
AF25MAG018	Yes	94.41	0.73	4689654	293	24318	42.6	81.92	4817	d Bacteria;p Cyanobacteria;c Cyanobacteriia;o Cyanobacteriales;f Microcystaceae;g Microcystis;s Microcystis aeruginosa F
AF25MAG020	Yes	77.34	0	678646	24	60562	40.7	84.89	673	d Bacteria;p Patescibacteria;c Paceibacteria;o UBA9983 A;f ;g ;s
AF25MAG021	Yes	90.87	1.39	2608728	464	8325	68.6	89.58	3190	d Bacteria;p Cyanobacteria;c Cyanobacteriia;o PCC-6307;f Cyanobiaceae;g UBA5018;s
AF25MAG022	Yes	95.32	3.29	3741786	355	16973	67	90.48	3748	d Bacteria;p Proteobacteria;c Alphaproteobacteria;o Sphingomonadales;f Sphingomonadaceae;g Sphingomonas;s Sphingomonas hankookensis
AF25MAG025	Yes	81.16	0.89	1230495	122	15861	55	89.15	1363	d Bacteria;p Proteobacteria;c Alphaproteobacteria;o UBA1280;f UBA6156;g RFNJ01;s
AF25MAG026	Yes	73.12	1.88	795798	96	14057	32.1	92.84	916	d Bacteria;p Patescibacteria;c WWE3;o UBA101185;f ;g ;s
AF25MAG027	Yes	85.94	3.96	2902400	486	8501	68.4	94.09	3240	d Bacteria;p Actinobacteriota;c Actinomycetia;o Nanopelagicales;f S36-B12;g Mxb001;s Mxb001 sp016870425
AF25MAG028	Yes	91.56	2.06	3084121	906	3906	70.3	88.09	3546	d Bacteria;p Deinococcota;c Deinococci;o Deinococcales;f Deinococcaceae;g Deinococcus;s Deinococcus indicus
AF25MAG029	Yes	81.92	0.9	2077369	577	3972	44.8	85.28	2241	d Bacteria;p Cyanobacteria;c Cyanobacteriia;o CAIUCS01;f CAIUCS01;g CAIUCS01;s CAIUCS01 sp903897715
AF25MAG030	Yes	96.21	6.11	3067943	147	38154	70.1	91.84	3069	d Bacteria;p Actinobacteriota;c Actinomycetia;o Actinomycetales;f Microbacteriaceae;g Microbacterium;s
AF25MAG032	Yes	84.68	1.35	1444843	360	4870	37.9	93.03	1567	d Bacteria;p Chlamydiota;c Chlamydiia;o Chlamydiales;f JACRBE01;g ;s
AF25MAG033	Yes	77.76	0.88	1060743	196	6937	51.7	95.51	1256	d Bacteria;p Actinobacteriota;c Actinomycetia;o Actinomycetales;f Microbacteriaceae;g Aquiluna;s
AF25MAG034	Yes	75.58	4.04	1657793	592	2861	43.1	93.61	2113	d Bacteria;p Bdellovibrionota;c FAC87;o UBA2466;f UBA2466;g RFNO01;s
AF25MAG035	Yes	86.02	2.04	2509119	583	5167	61.7	88.04	2961	d Bacteria;p Proteobacteria;c Alphaproteobacteria;o Rhizobiales;f Beijerinckiaceae;g Methylocystis;s
AF25MAG036	Yes	79.1	8.69	1892100	475	4608	63.2	92.26	2209	d Bacteria;p Verrucomicrobiota;c Verrucomicrobiae;o Chthoniobacterales;f Terrimicrobiaceae;g UBA967;s
AF25MAG037	Yes	75.63	2.56	1373615	269	6151	69.3	96.07	1629	d Bacteria;p Actinobacteriota;c Acidimicrobiia;o Acidimicrobiales;f Ilumatobacteraceae;g UBA3006;s
AF25MAG038	Yes	83.46	6.72	1124927	256	5304	34.3	87.72	1300	d Bacteria;p Patescibacteria;c Paceibacteria;o SW-4-49-11;f SW-4-49-11;g ;s
AF25MAG039	Yes	84.71	6.56	3275298	348	12434	70.3	92.3	3411	d Bacteria;p Actinobacteriota;c Actinomycetia;o Actinomycetales;f Microbacteriaceae;g Microbacterium;s
AF25MAG041	Yes	85.85	1.82	3989192	641	7927	64.4	88.51	3760	d Bacteria;p Chloroflexota;c Anaerolineae;o Caldilineales;f Caldilineaceae;g Litorilinea;s
AF25MAG042	Yes	91.04	4.26	5511818	1103	6436	65	92.01	6150	d Bacteria;p Actinobacteriota;c Actinomycetia;o Mycobacteriales;f Mycobacteriaceae;g Rhodococcus B;s Rhodococcus B sp000954115
AF25MAG043	Yes	93.49	0.99	2656449	354	9523	52.8	91.26	3003	d Bacteria;p Firmicutes;c Bacilli;o Exiguobacterales;f Exiguobacteraceae;g Exiguobacterium;s Exiguobacterium chiriqhucha
AF29MAG001	Yes	91.45	1.38	2914462	100	55075	65.5	95.32	2984	d Bacteria;p Actinobacteriota;c Acidimicrobiia;o Acidimicrobiales;f Ilumatobacteraceae;g ;s
AF29MAG002	Yes	96.58	2.23	2316075	61	68145	68.9	92.54	2203	d Bacteria;p Actinobacteriota;c Acidimicrobiia;o Acidimicrobiales;f UBA8139;g M30B73;s
AF29MAG003	Yes	83.03	1.08	1316070	115	20320	42.8	95.63	1437	d Bacteria;p Actinobacteriota;c Actinomycetia;o Nanopelagicales;f AcAMD-5;g ATZT02;s
AF29MAG004	Yes	98.29	1.28	1847868	57	134538	59.2	93.7	1813	d Bacteria;p Actinobacteriota;c Actinomycetia;o Nitriliruptorales;f Nitriliruptoraceae;g SLDB01;s
AF29MAG005	Yes	95.73	0.27	2324024	55	71353	62.2	94.4	2242	d Bacteria;p Actinobacteriota;c Actinomycetia;o Nanopelagicales;f S36-B12;g M55B157;s
AF29MAG006	No	92.1	2.44	1788776	203	13861	72.4	94.7	1999	d Bacteria;p Actinobacteriota;c Thermoleophilia;o Miltoncostaeales;f Miltoncostaeaceae;g SYFI01;s
AF29MAG007	No	86.63	1.63	2461429	289	12078	65.3	93.67	2572	d Bacteria;p Proteobacteria;c Gammaproteobacteria;o PWYM01;f PWYM01;g PWYM01;s
AF29MAG009	Yes	97.7	0	4073528	336	20300	70.5	89.3	3330	d Bacteria;p Planctomycetota;c Planctomycetia;o Pirellulales;f UBA1268;g QWPN01;s
AF29MAG010	Yes	96.58	3.07	2152087	161	22795	68.5	91.85	2158	d Bacteria;p Actinobacteriota;c Acidimicrobiia;o Acidimicrobiales;f UBA8139;g M30B73;s
AF29MAG011	No	83.02	3.47	2453627	479	6043	54.8	94.65	2645	d Bacteria;p Spirochaetota;c Spirochaetia;o DSM-27196;f Alkalispirochaetaceae;g Alkalispirochaeta;s
AF29MAG012	No	94.02	3.16	2658413	398	10332	65.7	93.47	2800	d Bacteria;p Actinobacteriota;c Acidimicrobiia;o Acidimicrobiales;f Ilumatobacteraceae;g CSBr16-110;s
AF29MAG014	Yes	92.81	5.86	2872674	303	14193	63.4	91.98	3046	d Bacteria;p Proteobacteria;c Alphaproteobacteria;o Rhodobacterales;f Rhodobacteraceae;g Roseicyclus;s
AF29MAG016	No	88.35	0.42	2165063	235	14027	70.7	94.19	2171	d Bacteria;p Deinococcota;c Deinococci;o Deinococcales;f Trueperaceae;g JAABTL01;s
AF29MAG017	Yes	97.34	2.99	2439865	229	21512	66.5	95.08	2496	d Bacteria;p Actinobacteriota;c Acidimicrobiia;o Acidimicrobiales;f Ilumatobacteraceae;g CSBr16-110;s
AF29MAG018	Yes	89.4	2.91	2725565	202	37086	54.4	92.13	2856	d Bacteria;p Proteobacteria;c Gammaproteobacteria;o Burkholderiales;f Burkholderiaceae;g Algicoccus;s
AF29MAG019	Yes	87.49	4.83	3291009	751	5445	61	93.04	4052	d Bacteria;p Proteobacteria;c Alphaproteobacteria;o Sphingomonadales;f Sphingomonadaceae;g Erythrobacter;s
AF29MAG022	Yes	83.22	0.82	2263653	177	19493	43.8	90.5	2066	d Bacteria;p Bacteroidota;c Rhodothermia;o Balneolales;f HLUCCA01;g UBA11400;s
AF29MAG023	Yes	88.28	7.94	2084220	407	6275	58.3	95.2	2190	d Bacteria;p Proteobacteria;c Gammaproteobacteria;o Xanthomonadales;f Wenzhouxiangellaceae;g Wenzhouxiangella;s
AF29MAG024	No	93.68	5.54	2232298	192	17567	70.1	92.13	2444	d Bacteria;p Cyanobacteria;c Cyanobacteriia;o PCC-6307;f Cyanobiaceae;g NIES-981;s
AF29MAG027	Yes	80.22	2.72	2518737	687	4222	56.7	92.5	2613	d Bacteria;p Verrucomicrobiota;c Verrucomicrobiae;o Verrucomicrobiales;f Akkermansiaceae;g Luteolibacter;s
AF29MAG028	Yes	73.13	4.84	2375096	695	3979	69.1	90.4	3185	d Bacteria;p Cyanobacteria;c Cyanobacteriia;o PCC-6307;f Cyanobiaceae;g NIES-981;s

AF29MAG031	Yes	90.03	1.71	1843977	63	52725	73.9	92.99	1732	d	Bacteria;p	Actinobacteriota;c	Actinomycetia;o	Nitriliruptorales;f	Nitriliruptoraceae;g	;s	
AF29MAG032	Yes	73.2	9.4	1899824	640	3087	66.8	95.19	2345	d	Bacteria;p	Actinobacteriota;c	Acidimicrobiia;o	Acidimicrobiales;f	Ilumatobacteraceae;g	CSBr16-110;s	
AF29MAG034	No	86.7	1.25	2391423	578	4929	39.7	90.97	2570	d	Bacteria;p	Bacteroidota;c	Bacteroidia;o	Cytophagales;f	Cyclobacteriaceae;g	Cecembia;s	
AF30MAG001	Yes	80.1	7.16	2283962	719	3394	35.1	92.06	2753	d	Bacteria;p	Bacteroidota;c	Bacteroidia;o	Flavobacteriales;f	Flavobacteriaceae;g	Flavobacterium;s	Flavobacterium sp018335925
AF30MAG002	Yes	94.45	2.84	3676649	333	18313	43.9	90.96	3468	d	Bacteria;p	Bacteroidota;c	Bacteroidia;o	Cytophagales;f	Cyclobacteriaceae;g	Algoriphagus;s	Algoriphagus sp018336015
AF30MAG003	Yes	95.05	1.97	3487267	125	45976	63.4	92.35	3460	d	Bacteria;p	Proteobacteria;c	Gammaproteobacteria;o	Burkholderiales;f	SG8-41;g	Ga0077527;s	
AF30MAG005	Yes	94.44	2.4	1740872	62	61519	60.3	95.35	1767	d	Bacteria;p	Proteobacteria;c	Gammaproteobacteria;o	Burkholderiales;f	Burkholderiaceae;g	;s	
AF30MAG006	Yes	97.13	1.36	1860635	86	36872	57.2	95.17	1745	d	Bacteria;p	Bacteroidota;c	Bacteroidia;o	Flavobacteriales;f	Schleiferiaceae;g	TMED14;s	
AF30MAG007	Yes	95.27	1.67	3013275	373	11847	38.8	87.95	3145	d	Bacteria;p	Proteobacteria;c	Gammaproteobacteria;o	Pseudomonadales;f	Moraxellaceae;g	Acinetobacter;s	Acinetobacter junii
AF30MAG008	Yes	93.29	3.1	1722518	143	41912	59.5	95.64	1812	d	Bacteria;p	Proteobacteria;c	Gammaproteobacteria;o	Burkholderiales;f	Burkholderiaceae;g	UBA2463;s	
AF30MAG010	Yes	88.45	4.14	2531622	365	9368	61.2	94.3	2716	d	Bacteria;p	Proteobacteria;c	Gammaproteobacteria;o	Burkholderiales;f	Rhodocyclaceae;g	Azonexus;s	
AF30MAG011	Yes	78.17	2.19	1239460	287	5110	44.8	94.49	1473	d	Bacteria;p	Actinobacteriota;c	Actinomycetia;o	Nanopelagicales;f	UBA5976;g	UBA5976;s	
AF30MAG012	Yes	71.09	6.63	970628	275	3980	62.7	95.05	1156	d	Bacteria;p	Actinobacteriota;c	Actinomycetia;o	Actinomycetales;f	Microbacteriaceae;g	;s	
AF30MAG013	Yes	91.27	1.79	1591169	125	26635	60.2	95.46	1677	d	Bacteria;p	Actinobacteriota;c	Actinomycetia;o	Nanopelagicales;f	UBA5976;g	;s	
AF30MAG014	Yes	95.39	4.03	1469544	89	28048	45.5	93.21	1542	d	Bacteria;p	Proteobacteria;c	Gammaproteobacteria;o	Burkholderiales;f	Methylophilaceae;g	RFPI01;s	RFPI01 sp009926205
AF30MAG016	Yes	83.57	5.1	2623792	528	6446	49.8	93.41	2713	d	Bacteria;p	Bacteroidota;c	Bacteroidia;o	UBA7662;f	UBA7662;g	PNNG01;s	
AF30MAG017	Yes	77.76	7.74	2464205	780	3474	66.6	93.74	2970	d	Bacteria;p	Proteobacteria;c	Alphaproteobacteria;o	Sphingomonadales;f	Sphingomonadaceae;g	Sandarakinorhabdus;s	
AF30MAG018	Yes	81.07	4.76	2720848	461	8890	56.2	93.31	2827	d	Bacteria;p	Proteobacteria;c	Gammaproteobacteria;o	Pseudomonadales;f	Pseudohongiellaceae;g	Pseudohongiella;s	
AF30MAG019	Yes	87.43	6.2	1751920	281	8670	58.1	94.66	1936	d	Bacteria;p	Proteobacteria;c	Gammaproteobacteria;o	Burkholderiales;f	Burkholderiaceae;g	;s	
AF30MAG020	Yes	88.75	3.02	1825666	141	25835	56.2	95.25	1885	d	Bacteria;p	Proteobacteria;c	Gammaproteobacteria;o	Burkholderiales;f	Burkholderiaceae;g	;s	
AF30MAG024	Yes	85.31	7.47	3746775	928	4675	68.9	90.9	3765	d	Bacteria;p	Verrucomicrobiota;c	Verrucomicrobiae;o	Opitutales;f	Opitutaceae;g	IMCC26134 A;s	
AF30MAG025	Yes	72.86	2.49	2158500	256	11747	62.8	94.63	2269	d	Bacteria;p	Proteobacteria;c	Gammaproteobacteria;o	Burkholderiales;f	Burkholderiaceae;g	UBA997;s	
AF30MAG028	Yes	89.48	8.41	2863265	371	10716	64.1	93.37	3053	d	Bacteria;p	Proteobacteria;c	Gammaproteobacteria;o	Burkholderiales;f	Burkholderiaceae;g	Rubrivivax;s	
AF30MAG030	Yes	73.67	3.25	1057445	99	17255	48.4	95.99	1117	d	Bacteria;p	Actinobacteriota;c	Actinomycetia;o	Nanopelagicales;f	Nanopelagicaceae;g	MAG-120802;s	
AF30MAG032	Yes	83.61	4.87	1244525	67	50355	54.7	95.26	1316	d	Bacteria;p	Actinobacteriota;c	Actinomycetia;o	Actinomycetales;f	Microbacteriaceae;g	Rhodoluna;s	
AF30MAG036	Yes	83.22	4.68	2473879	371	8485	65.9	94.16	2599	d	Bacteria;p	Proteobacteria;c	Gammaproteobacteria;o	Burkholderiales;f	Burkholderiaceae;g	RFTU01;s	
AF30MAG039	Yes	76.59	4.22	3998252	557	10304	62.5	92.07	4189	d	Bacteria;p	Proteobacteria;c	Gammaproteobacteria;o	Burkholderiales;f	Rhodocyclaceae;g	Azoarcus C;s	Azoarcus C communis
AF30MAG044	Yes	77.53	3.6	1173792	116	17025	48.8	95.89	1258	d	Bacteria;p	Actinobacteriota;c	Actinomycetia;o	Nanopelagicales;f	Nanopelagicaceae;g	Planktophila;s	
AF30MAG050	Yes	78.16	2.75	1061483	79	24704	52.1	95.01	1104	d	Bacteria;p	Actinobacteriota;c	Actinomycetia;o	Actinomycetales;f	Microbacteriaceae;g	Rhodoluna;s	
AF30MAG060	Yes	87.82	1.56	1123052	80	27395	52.4	95.8	1177	d	Bacteria;p	Actinobacteriota;c	Actinomycetia;o	Actinomycetales;f	Microbacteriaceae;g	Rhodoluna;s	
AF30MAG066	No	75.86	6.9	1484803	329	5426	74.3	92.98	1648	d	Bacteria;p	Actinobacteriota;c	Actinomycetia;o	Nitriliruptorales;f	Nitriliruptoraceae;g	;s	
AF30MAG070	Yes	70.76	6.67	1230479	212	6784	47.7	96.23	1402	d	Bacteria;p	Actinobacteriota;c	Actinomycetia;o	Nanopelagicales;f	Nanopelagicaceae;g	Planktophila;s	
AF30MAG071	Yes	77.42	9.64	3241465	761	5334	64.7	92.37	3823	d	Bacteria;p	Proteobacteria;c	Alphaproteobacteria;o	Sphingomonadales;f	Sphingomonadaceae;g	Novosphingobium;s	
AF30MAG092	Yes	86.82	8.35	2916831	357	12861	68	93.34	3079	d	Bacteria;p	Proteobacteria;c	Gammaproteobacteria;o	Burkholderiales;f	Burkholderiaceae;g	;s	
AF32MAG001	Yes	85.71	0.38	2599912	50	127940	43.6	90.35	2329	d	Bacteria;p	Bacteroidota;c	Bacteroidia;o	Cytophagales;f	Cyclobacteriaceae;g	Algoriphagus;s	
AF32MAG002	Yes	88.89	1.28	1819845	69	89712	63	95.02	1818	d	Bacteria;p	Actinobacteriota;c	Acidimicrobiia;o	Acidimicrobiales;f	Ilumatobacteraceae;g	UBA2093;s	
AF32MAG003	Yes	86.23	2.73	1646558	117	17737	46.6	95.81	1607	d	Bacteria;p	Bacteroidota;c	Bacteroidia;o	Flavobacteriales;f	Flavobacteriaceae;g	UBA3478;s	
AF32MAG004	Yes	83.84	2.07	1339997	24	193570	41.6	95.89	1377	d	Bacteria;p	Actinobacteriota;c	Actinomycetia;o	Nanopelagicales;f	AcAMD-5;g	ATZT02;s	
AF32MAG005	Yes	85.84	1.07	2660646	47	117474	63.9	91.16	2503	d	Bacteria;p	Proteobacteria;c	Alphaproteobacteria;o	Sphingomonadales;f	Sphingomonadaceae;g	Erythrobacter;s	Erythrobacter sanguineus
AF32MAG006	Yes	98.29	2.14	2967323	87	81386	67.1	93.12	2848	d	Bacteria;p	Actinobacteriota;c	Acidimicrobiia;o	Acidimicrobiales;f	Ilumatobacteraceae;g	;s	
AF32MAG007	Yes	78.46	1.91	2392361	82	52756	64.4	90.77	2307	d	Bacteria;p	Proteobacteria;c	Alphaproteobacteria;o	Sphingomonadales;f	Sphingomonadaceae;g	Erythrobacter;s	
AF32MAG008	Yes	91.74	1.68	2963188	125	42452	33.7	88.49	2735	d	Bacteria;p	Bdellovibrionota C;c	UBA2361;o	SZUA-149;f	SZUA-149;g	;s	
AF32MAG009	Yes	89.33	0.57	1022600	42	40491	33.2	94.71	1007	d	Bacteria;p	Firmicutes;c	Bacilli;o	Acholeplasmatales;f	Acholeplasmataceae;g	UBA8290;s	
AF32MAG011	Yes	87.32	5.6	2217595	190	32204	53.6	94.05	2138	d	Bacteria;p	Proteobacteria;c	Gammaproteobacteria;o	Xanthomonadales;f	Wenzhouxiangellaceae;g	Wenzhouxiangella;s	
AF32MAG012	Yes	90.93	4.09	1563097	180	14876	63	96.1	1722	d	Bacteria;p	Proteobacteria;c	Gammaproteobacteria;o	Burkholderiales;f	Burkholderiaceae;g	UBA2463;s	
AF32MAG013	No	90.73	0.34	3277971	47	143538	67.2	90.24	3058	d	Bacteria;p	Proteobacteria;c	Alphaproteobacteria;o	Sphingomonadales;f	Sphingomonadaceae;g	Sphingomonas;s	Sphingomonas hankookensis
AF32MAG014	Yes	79.4	1.39	2477103	336	10378	67.4	91.09	2560	d	Bacteria;p	Proteobacteria;c	Alphaproteobacteria;o	Rhodobacterales;f	Rhodobacteraceae;g	Rhodovulum;s	
AF32MAG016	Yes	92.52	8.31	3455618	429	17653	43.9	92.99	4288	d	Bacteria;p	Proteobacteria;c	Gammaproteobacteria;o	Burkholderiales;f	Methylophilaceae;g	RFPI01;s	
AF32MAG017	Yes	90.77	1.97	1687088	159	15985	53.9	94.85	1808	d	Bacteria;p	Proteobacteria;c	Gammaproteobacteria;o	Burkholderiales;f	Burkholderiaceae;g	UBA2463;s	
AF32MAG019	Yes	85.65	2.98	2275316	708	3601	52.7	90.86	2896	d	Bacteria;p	Firmicutes;c	Bacilli;o	Exiguobacterales;f	Exiguobacteraceae;g	Exiguobacterium;s	Exiguobacterium mexicanum
AF32MAG020	Yes	77.34	3.03	953412	47	223099	46.3	89.98	1091	d	Bacteria;p	Patescibacteria;c	Paceibacteria;o	UBA9983 A;f	UBA2103;g	1-14-0-10-45-20;s	
AF32MAG022	Yes	92.08	6.3	2343393	315	14873	47	92.14	2180	d	Bacteria;p	Bacteroidota;c	Rhodothermia;o	Balneolales;f	Balneolaceae;g	;s	
AF32MAG023	Yes	78.59	4.2	2248481	556	4620	44.2	83.13	2305	d	Bacteria;p	Proteobacteria;c	Gammaproteobacteria;o	Pseudomonadales;f	Moraxellaceae;g	Psychrobacter;s	
AF32MAG024	Yes	84.78	2.61	1392050	155	13363	59.2	94.56	1495	d	Bacteria;p	Actinobacteriota;c	Actinomycetia;o	Actinomycetales;f	Microbacteriaceae;g	Pontimonas;s	
AF32MAG025	Yes	91.15	5.67	2861117	384	9701	41.6	87.03	3026	d	Bacteria;p	Proteobacteria;c	Gammaproteobacteria;o	Pseudomonadales;f	Moraxellaceae;g	Acinetobacter;s	Acinetobacter johnsonii
AF32MAG028	Yes	74.82	2.44	1467949	447	3361	50	94.69	1762	d	Bacteria;p	Proteobacteria;c	Gammaproteobacteria;o	Enterobacterales;f	Alteromonadaceae;g	Pseudidiomarina;s	
AF32MAG029	No	85.97	5.53	3601885	440	12113	65.4	91.87	3881	d	Bacteria;p	Proteobacteria;c	Alphaproteobacteria;o	Rhodobacterales;f	Rhodobacteraceae;g	EhC02;s	
AF32MAG031	Yes	78.85	2.02	2401887	582	4921	70.6	94.76	2954	d	Bacteria;p	Actinobacteriota;c	Actinomycetia;o	Actinomycetales;f	Microbacteriaceae;g	Rhodoglobus;s	Rhodoglobus sp001725325
AF32MAG032	Yes	75.96	3.84	1882881	576	3621	57.3	92.87	2315	d	Bacteria;p	Proteobacteria;c	Alphaproteobacteria;o	Rhodobacterales;f	Rhodobacteraceae;g	M30B80;s	
AF32MAG033	No	93.92	0.31	2871213	123	43530	38.5	87.53	2810	d	Bacteria;p	Proteobacteria;c	Gammaproteobacteria;o	Pseudomonadales;f	Moraxellaceae;g	Acinetobacter;s	Acinetobacter junii
AF32MAG035	Yes	71.68	6.66	4753268	717	11622	53.2	90.73	5697	d	Bacteria;p	Patescibacteria;c	Paceibacteria;o	UBA9983 A;f	UBA918;g	OLB19;s	
AF32MAG037	Yes	76.13	3.32	1541718	230	9881	57.6	94.06	1682	d	Bacteria;p	Proteobacteria;c	Gammaproteobacteria;o	Burkholderiales;f	Burkholderiaceae;g	;s	
AF32MAG040	Yes	76.31	3.61	556988	90	7319	39	90.71	643	d	Bacteria;p	Patescibacteria;c	Paceibacteria;o	UBA9983 A;f	CSBR16-119;g	CSBR16-119;s	
AF32MAG044	Yes	75.79	8.68	1830798	380	6296	65.8	94.51	1999	d	Bacteria;p	Actinobacteriota;c	Actinomycetia;o	Nanopelagicales;f	;g	;s	
AF32MAG046	Yes	73.69	3.15	2234000	637	3792	39.2	93.48	2647	d	Bacteria;p	Bacteroidota;c	Bacteroidia;o	NS11-12g;f	UBA955;g	VMCP01;s	
AF32MAG049	Yes	92.54	7.68	1980320	213	35504	53.8	95.03	2028	d	Bacteria;p	Actinobacteriota;c	Actinomycetia;o	Actinomycetales;f	Microbacteriaceae;g	Aquiluna;s	
AF32MAG051	Yes	85.96	7	4132323	571	10680	60.9	89.93	4618	d	Bacteria;p	Proteobacteria;c	Alphaproteobacteria;o	Sphingomonadales;f	Sphingomonadaceae;g	Erythrobacter;s	
AF32MAG056	Yes	83.38	7.21	1377165	108	30763	55.8	96.05	1394	d	Bacteria;p	Actinobacteriota;c	Actinomycetia;o	Actinomycetales;f	Microbacteriaceae;g	Aquiluna;s	
AF32MAG059	Yes	82.67	6.65	3238460	416	14523	52	93.07	3636	d	Bacteria;p	Proteobacteria;c	Gammaproteobacteria;o	Burkholderiales;f	Burkholderiaceae;g	Algicoccus;s	
AF32MAG076	Yes	87.03	9.3	2710009	463	7956	54.4	92.25	2922	d	Bacteria;p	Proteobacteria;c	Gammaproteobacteria;o	Burkholderiales;f	Burkholderiaceae;g	Algicoccus;s	
AF34MAG001	Yes	97.33	0	1125678	27	237059	41.9	94.09	1107	d	Bacteria;p	Firmicutes;c	Bacilli;o	Izemoplasmatales;f	Izemoplasmataceae;g	CSBR16-87;s	
AF34MAG002	Yes	95.73	1.71	2242471	39	132855	63.4	91.56	2087	d	Bacteria;p	Actinobacteriota;c	Acidimicrobiia;o	Acidimicrobiales;f	UBA8139;g	M30B73;s	
AF34MAG003	Yes	90.35	0.82	1881910	58	79838	55.6	95.94	1870	d	Bacteria;p	Proteobacteria;c	Gammaproteobacteria;o	Nitrococcales;f	Nitrococcaceae;g	Spiribacter;s	
AF34MAG004	Yes	95.14	3.78	3860003	192	51007	35.2	90.22	3478	d	Bacteria;p	Bacteroidota;c	Bacteroidia;o	Flavobacteriales;f	Crocinitomicaceae;g	CSBr16-161;s	CSBr16-161 sp007117355
AF34MAG005	Yes	94.89	0.54	2538421	111	37566	44.5	90.77	2312	d	Bacteria;p	Bacteroidota;c	Bacteroidia;o	Flavobacteriales;f	Schleiferiaceae;g	CSBr16-58;s	
AF34MAG006	Yes	91.35	0.05	3023340	190	26704	35.5	91.11	2782	d	Bacteria;p	Bacteroidota;c	Bacteroidia;o	Flavobacteriales;f	Crocinitomicaceae;g	;s	
AF34MAG007	Yes	95.78	4.36	2946358	216	22224	55.3	89.69	2763	d	Bacteria;p	Verrucomicrobiota;c	Kiritimatiellae;o	SLAD01;f	SLAD01;g	SLAD01;s	
AF34MAG008	Yes	96.77	0.94	2581426	210	18838	64	88.99	2407	d	Bacteria;p	Proteobacteria;c	Alphaproteobacteria;o	SMXQ01;f	SMXQ01;g	;s	
AF34MAG009	No	94.57	0.68	2324537	201	18133	70.1	91.9	2587	d	Bacteria;p	Cyanobacteria;c	Cyanobacteriia;o	PCC-6307;f	Cyanobiaceae;g	NIES-981;s	
AF34MAG010	No	96.58	1.28	1714413	78	57682	59.3	93.75	1714	d	Bacteria;p	Actinobacteriota;c	Actinomycetia;o	Nitriliruptorales;f	Nitriliruptoraceae;g	SLDB01;s	
AF34MAG011	Yes	90.87	3.26	2950547	226	19468	62.9	88.34	2928	d	Bacteria;p	Proteobacteria;c	Alphaproteobacteria;o	Rhodobacterales;f	Rhodobacteraceae;g	Roseovarius;s	
AF34MAG012	Yes	98.84	5.49	2887239	57	173928	61.3	93.89	2427	d	Bacteria;p	Gemmatimonadota;c	Gemmatimonadetes;o	Longimicrobiales;f	UBA6960;g	SKNW01;s	
AF34MAG013	Yes	97.54	2.51	3573520	272	28256	47.9	87.1	3205	d	Bacteria;p	Bacteroidota;c	Rhodothermia;o	Balneolales;f	PXAI01;g	Cyclonatronum;s	
AF34MAG014	Yes	90.68	3.81	3431350	371	15820	71.1	92.65	3410	d	Bacteria;p	Deinococcota;c	Deinococci;o	Deinococcales;f	Trueperaceae;g	CSSed10-48;s	
AF34MAG015	Yes	80.14	0	934518	37	51129	36.8	93.78	1047	d	Archaea;p	Nanoarchaeota;c	Nanoarchaeia;o	Woesearchaeales;f	JAFGUC01;g	;s	
AF34MAG016	Yes	95.63	1.37	2762692	222	22577	46.7	89.91	2455	d	Bacteria;p	Bacteroidota;c	Rhodothermia;o	Balneolales;f	Balneolaceae;g	UBA2664;s	
AF34MAG018	No	87.73	3.2	2438802	133	28966	54.9	95.16	2353	d	Bacteria;p	Spirochaetota;c	Spirochaetia;o	DSM-27196;f	Alkalispirochaetaceae;g	Alkalispirochaeta;s	
AF34MAG019	Yes	83.05	3.18	2965465	363	10532	53.2	94.42	2716	d	Bacteria;p	Verrucomicrobiota;c	Verrucomicrobiae;o	Opitutales;f	Verruco-01;g	;s	
AF34MAG020	No	80.77	5.56	2130423	164	22410	64.6	95.05	2186	d	Bacteria;p	Actinobacteriota;c	Acidimicrobiia;o	Acidimicrobiales;f	Ilumatobacteraceae;g	M30B72;s	
AF34MAG021	Yes	81.31	0.93	1067073	38	44980	47.9	91.28	1326	d	Archaea;p	Nanoarchaeota;c	Nanoarchaeia;o	Woesearchaeales;f	SKIA01;g	SKIA01;s	

AF34MAG022	Yes	100	7.76	5013350	624	33299	64.9	92.38	5845	d	Bacteria;p	Proteobacteria;c Gammaproteobacteria;o PWYM01;f PWYM01;g PWYM01;s
AF34MAG023	No	70.77	1.12	599044	11	92091	36.8	88.11	614	d	Bacteria;p	Patescibacteria;c Paceibacteria;o UBA9983 A;f CSBR16-119;g CSBR16-119;s CSBR16-119 sp007117675
AF34MAG024	Yes	97.8	2.31	3860143	143	47324	64.4	90.36	3536	d	Bacteria;p	Proteobacteria;c Gammaproteobacteria;o Pseudomonadales;f Spongiibacteraceae;g ;s
AF34MAG025	Yes	93.59	4.86	3041121	368	14341	64.3	88.69	3293	d	Bacteria;p	Proteobacteria;c Gammaproteobacteria;o Ectothiorhodospirales;f Thioalkalivibrionaceae;g Thioalkalivibrio;s Thioalkalivibrio sp000376865
AF34MAG026	Yes	96.66	6.01	3566748	359	40457	42.4	89.77	3494	d	Bacteria;p	Bacteroidota;c Rhodothermia;o Balneolales;f Balneolaceae;g UBA2664;s
AF34MAG027	No	72.26	0	763419	65	17639	40.6	92.08	742	d	Bacteria;p	Patescibacteria;c JAEDAM01;o Absconditabacterales;f X112;g ;s
AF34MAG028	Yes	89.36	1.35	2316879	445	6415	56.9	88.65	2256	d	Bacteria;p	Verrucomicrobiota;c Verrucomicrobiae;o Opitutales;f Puniceicoccaceae;g ;s
AF34MAG029	Yes	84.01	2.1	4365844	665	8402	48.3	87.94	4282	d	Bacteria;p	Bdellovibrionota;c Oligoflexia;o Oligoflexales;f Oligoflexaceae;g ;s
AF34MAG030	Yes	87.37	7.98	3239850	196	31216	43.4	90.3	2875	d	Bacteria;p	Bacteroidota;c Rhodothermia;o Balneolales;f Balneolaceae;g UBA2664;s
AF34MAG031	Yes	84.58	0.93	1123631	47	39714	39.9	90.55	1181	d	Archaea;p	Nanoarchaeota;c Nanoarchaeia;o Woesearchaeales;f ;g ;s
AF34MAG032	No	89.25	4	2412666	507	6055	56	93.18	2656	d	Bacteria;p	Proteobacteria;c Gammaproteobacteria;o Burkholderiales;f Burkholderiaceae;g Algicoccus;s
AF34MAG033	Yes	80.47	2.03	2875423	686	5193	57.3	93.72	2933	d	Bacteria;p	Verrucomicrobiota;c Verrucomicrobiae;o Opitutales;f ;g ;s
AF34MAG034	Yes	91.67	5.38	3190352	533	9000	42.8	88.16	3086	d	Bacteria;p	Bacteroidota;c Bacteroidia;o Flavobacteriales;f Salibacteraceae;g Salibacter;s
AF34MAG035	Yes	77.98	2.56	3255298	860	4426	50.2	90.38	3316	d	Bacteria;p	Verrucomicrobiota;c Verrucomicrobiae;o Opitutales;f JAFGAQ01;g ;s
AF34MAG036	Yes	84.29	1.1	2685022	636	4981	71.4	91.89	2724	d	Bacteria;p	Gemmatimonadota;c Gemmatimonadetes;o Longimicrobiales;f UBA6960;g REBX01;s
AF34MAG037	Yes	95.14	7.69	3692803	404	28896	50.3	89.22	3348	d	Bacteria;p	Bacteroidota;c Rhodothermia;o Balneolales;f Balneolaceae;g ;s
AF34MAG039	Yes	90.95	3.01	2975530	68	155548	41.1	90.25	2552	d	Bacteria;p	Bacteroidota;c Rhodothermia;o Balneolales;f Balneolaceae;g UBA2664;s
AF34MAG041	Yes	80.05	6.83	2396401	212	16368	45.6	90.14	2278	d	Bacteria;p	Bacteroidota;c Rhodothermia;o Balneolales;f Balneolaceae;g SLAU01;s
AF34MAG042	Yes	81.97	8.01	2629326	374	9271	46.5	90.11	2519	d	Bacteria;p	Bacteroidota;c Rhodothermia;o Balneolales;f Balneolaceae;g SLAU01;s
AF34MAG050	Yes	83.97	8	1654779	213	10734	57.8	94.34	1796	d	Bacteria;p	Actinobacteriota;c Actinomycetia;o Actinomycetales;f Microbacteriaceae;g Pontimonas;s
AF34MAG055	Yes	86.48	2.42	2574399	284	14027	54.1	90.99	2298	d	Bacteria;p	Bacteroidota;c Rhodothermia;o Balneolales;f Natronogracilivirgulaceae;g SKNL01;s
AF34MAG058	Yes	77.1	1.48	1785386	568	3461	55.1	92.61	1940	d	Bacteria;p	Bacteroidota;c Rhodothermia;o Balneolales;f Balneolaceae;g ;s
AF34MAG060	Yes	82.01	9.83	1950586	372	6630	53.7	93.82	2100	d	Bacteria;p	Proteobacteria;c Gammaproteobacteria;o Pseudomonadales;f Natronospirillaceae;g Natronospirillum;s
AF34MAG061	Yes	70.33	5.74	2282480	825	2851	52.6	90.52	2360	d	Bacteria;p	Bacteroidota;c Rhodothermia;o Balneolales;f Natronogracilivirgulaceae;g SLLW01;s
AF34MAG065	Yes	74.88	3.05	1628966	467	3832	54.5	91.81	1879	d	Bacteria;p	Proteobacteria;c Gammaproteobacteria;o Ectothiorhodospirales;f Thioalkalivibrionaceae;g ;s
Gb0053562MAG001	Yes	98.69	2.29	1821216	105	66609	50.7	89.46	1985	d	Archaea;p	Halobacteriota;c Methanomicrobia;o Methanomicrobiales;f Methanocorpusculaceae;g Methanocalculus;s Methanocalculus sp003838065
Gb0053562MAG002	Yes	97.04	2.96	2922240	111	58579	34.6	85.03	2387	d	Bacteria;p	Bacteroidota;c Bacteroidia;o Bacteroidales;f UBA7960;g PUKZ01;s PUKZ01 sp003550725
Gb0053562MAG003	Yes	100	0.73	2454883	149	42587	51.6	85.42	2339	d	Bacteria;p	Desulfobacterota I;c Desulfovibrionia;o Desulfovibrionales;f Desulfonatronovibrionaceae;g Desulfonatronospira;s Desulfonatronospira sp003551155
Gb0053562MAG004	Yes	96.7	2.73	4412565	228	41057	44.1	91.33	3996	d	Bacteria;p	Bacteroidota;c T3Sed10-11;o T3Sed10-11;f T3Sed10-11;g T3Sed10-11;s T3Sed10-11 sp003568415
Gb0053562MAG005	Yes	98.69	1.96	1865728	83	39058	52.5	90.66	1965	d	Archaea;p	Halobacteriota;c Methanomicrobia;o Methanomicrobiales;f Methanocorpusculaceae;g Methanocalculus;s Methanocalculus sp003838185
Gb0053562MAG006	No	98.5	1.95	2667320	138	35916	66.2	89.44	2745	d	Archaea;p	Halobacteriota;c Halobacteria;o Halobacteriales;f Haloferacaceae;g Halorubrum;s Halorubrum sp003554605
Gb0053562MAG007	Yes	98.55	0	1427675	34	89853	50.4	92.7	1418	d	Bacteria;p	Firmicutes;c Bacilli;o Izemoplasmatales;f Izemoplasmataceae;g T1SED10-81;s T1SED10-81 sp003552925
Gb0053562MAG008	No	92.88	0.1	2309660	155	62103	42.3	92.84	2067	d	Bacteria;p	CG03;c SLGR01;o SLGR01;f SLGR01;g PWEK01;s PWEK01 sp003553445
Gb0053562MAG009	Yes	89.19	1.2	1956099	115	42814	51.2	88.47	1878	d	Bacteria;p	Firmicutes D;c Dethiobacteria;o DTU022;f DTU022;g B1SED10-74M;s B1SED10-74M sp003554395
Gb0053562MAG010	Yes	93.83	0.56	2735324	227	27409	42.5	89.01	2735	d	Bacteria;p	Firmicutes F;c Halanaerobiia;o B1SED10-174;f B1SED10-174;g T1SED10-26;s T1SED10-26 sp003550445
Gb0053562MAG011	Yes	98.79	0.81	1499713	64	82349	62.8	91.84	1561	d	Archaea;p	Thermoplasmatota;c Thermoplasmata;o Methanomassiliicoccales;f Methanomethylophilaceae;g PWHV01;s PWHV01 sp003550345
Gb0053562MAG012	No	93.55	1.55	2318318	96	49682	48.4	88.06	2187	d	Bacteria;p	Firmicutes F;c Halanaerobiia;o B1SED10-174;f B1SED10-174;g T1SED10-26;s T1SED10-26 sp003554145
Gb0053562MAG013	Yes	92.74	0.99	1690174	103	26218	58.1	88.04	1723	d	Bacteria;p	Chloroflexota;c Dehalococcoidia;o GIF9;f AB-539-J10;g E29-bin54;s E29-bin54 sp003556385
Gb0053562MAG014	Yes	97.54	2.19	3354929	114	59141	50.3	90.45	2770	d	Bacteria;p	Bacteroidota;c Rhodothermia;o Balneolales;f Natronogracilivirgulaceae;g Natronogracilivirgula;s Natronogracilivirgula saccharolytica
Gb0053562MAG015	Yes	96.53	3.2	2430103	154	33396	40.2	91.23	2390	d	Archaea;p	Thermoplasmatota;c Thermoplasmata;o PWKY01;f PWKY01;g B1SED10-34;s B1SED10-34 sp003551065
Gb0053562MAG016	No	97.18	2.07	3013061	209	32658	66.2	86.8	2963	d	Bacteria;p	Proteobacteria;c Gammaproteobacteria;o Ectothiorhodospirales;f Thioalkalivibrionaceae;g Thioalkalivibrio B;s Thioalkalivibrio B sp003563455
Gb0053562MAG017	Yes	89.6	0.8	2202318	124	49186	44.2	92.53	1965	d	Archaea;p	Thermoplasmatota;c Thermoplasmata;o PWKY01;f PWKY01;g Natronoplasma;s Natronoplasma halalkaliphilum
Gb0053562MAG018	Yes	98.25	1.4	3279339	205	29812	61.1	88.52	3343	d	Archaea;p	Halobacteriota;c Halobacteria;o Halobacteriales;f Natrialbaceae;g Tc-Br11;s Tc-Br11 sp003552325
Gb0053562MAG019	Yes	97.62	2.84	3313040	305	20294	64.3	90.19	2917	d	Bacteria;p	Planctomycetota;c PUPC01;o PUPC01;f PUPC01;g PUPC01;s PUPC01 sp003553185
Gb0053562MAG020	No	93.13	1.1	2916725	166	37280	44.6	80.54	2524	d	Bacteria;p	Firmicutes F;c Halanaerobiia;o Halanaerobiales;f Halarsenatibacteraceae;g T1SED10-84;s T1SED10-84 sp003554225
Gb0053562MAG021	No	93.53	1.88	3607913	448	12954	59.6	90.58	3817	d	Bacteria;p	Proteobacteria;c Alphaproteobacteria;o Rhodobacterales;f Rhodobacteraceae;g Roseinatronobacter;s Roseinatronobacter monicus
Gb0053562MAG022	Yes	96.51	4.84	2985040	207	22108	49.3	88.59	2426	d	Bacteria;p	Bacteroidota;c Bacteroidia;o Bacteroidales;f UBA7960;g PUPG01;s PUPG01 sp003556325
Gb0053562MAG023	No	95.65	7.54	4553845	575	16242	65.1	86.99	4818	d	Bacteria;p	Proteobacteria;c Gammaproteobacteria;o Ectothiorhodospirales;f Thioalkalivibrionaceae;g Thioalkalivibrio B;s Thioalkalivibrio B sp007116035
Gb0053562MAG024	Yes	91.42	0.99	2715354	272	14185	55.7	88.08	2730	d	Bacteria;p	Chloroflexota;c Dehalococcoidia;o RBG-13-53-26;f RBG-13-53-26;g PUNJ01;s PUNJ01 sp003551705
Gb0053562MAG025	Yes	85.99	1.43	2423973	464	6767	44.6	90.64	2667	d	Bacteria;p	Firmicutes F;c Halanaerobiia;o B1SED10-174;f B1SED10-174;g B1SED10-174;s B1SED10-174 sp003551905
Gb0053562MAG026	No	97.8	4.4	4064845	222	37876	67.2	90.27	3411	d	Bacteria;p	Gemmatimonadota;c Gemmatimonadetes;o Longimicrobiales;f UBA6960;g PWLA01;s PWLA01 sp007121775
Gb0053562MAG027	Yes	97.2	2.5	3295286	230	25915	68.9	91.42	3321	d	Bacteria;p	Proteobacteria;c Alphaproteobacteria;o Rhodobacterales;f Rhodobacteraceae;g Rhodobaculum;s Rhodobaculum sp003550665
Gb0053562MAG028	Yes	88.14	1.69	1749089	57	51916	62.4	90.61	1758	d	Bacteria;p	Bipolaricaulota;c Bipolaricaulia;o Bipolaricaulales;f Bipolaricaulaceae;g T3SED10-47;s
Gb0053562MAG029	No	96.59	2.27	2941998	596	6389	47.5	89.38	3073	d	Bacteria;p	Planctomycetota;c Phycisphaerae;o Sedimentisphaerales;f SG8-4;g T1Sed10-97;s T1Sed10-97 sp003553245
Gb0053562MAG030	No	96.91	6.26	3492541	375	15034	64.5	91.01	3640	d	Bacteria;p	Proteobacteria;c Alphaproteobacteria;o Rhodobacterales;f Rhodobacteraceae;g Roseinatronobacter;s Roseinatronobacter sp003561595
Gb0053562MAG031	No	94.64	1.28	2543220	169	26231	54.9	90.54	2697	d	Archaea;p	Halobacteriota;c Halobacteria;o Halobacteriales;f Haloarculaceae;g Halovenus;s Halovenus sp003551945
Gb0053562MAG032	Yes	96.67	8.24	2210264	179	25223	62	89.18	2441	d	Archaea;p	Halobacteriota;c Halobacteria;o Halobacteriales;f Haloferacaceae;g PL-Br10-E2g29;s PL-Br10-E2g29 sp001563965
Gb0053562MAG034	Yes	98.28	2.72	5307829	355	25642	55.2	83.34	5076	d	Bacteria;p	Cyanobacteria;c Cyanobacteriia;o Phormidesmiales;f Phormidesmiaceae;g Nodosilinea;s Nodosilinea sp007135385
Gb0053562MAG035	Yes	94.56	1.93	2625278	279	42744	68.9	92.92	2645	d	Bacteria;p	Proteobacteria;c Gammaproteobacteria;o Nitrococcales;f Halorhodospiraceae;g Halorhodospira;s Halorhodospira sp003552345
Gb0053562MAG036	No	90.79	1.75	2353963	140	41354	40.4	89.88	2279	d	Bacteria;p	Firmicutes F;c Halanaerobiia;o Halanaerobiales;f Halarsenatibacteraceae;g T1SED10-84;s T1SED10-84 sp003553485
Gb0053562MAG038	Yes	92.81	2.82	2782004	242	21072	45.5	89.44	2781	d	Bacteria;p	Firmicutes F;c Halanaerobiia;o B1SED10-174;f B1SED10-174;g T1SED10-26;s T1SED10-26 sp003551665
Gb0053562MAG039	Yes	90.55	2.6	1259972	91	30538	55.7	93.22	1266	d	Bacteria;p	Firmicutes;c Bacilli;o Izemoplasmatales;f Izemoplasmataceae;g T1SED10-81;s T1SED10-81 sp003552275
Gb0053562MAG041	No	85.09	2.68	2514473	379	9009	68.9	88.48	2795	d	Archaea;p	Halobacteriota;c Halobacteria;o Halobacteriales;f Natrialbaceae;g Tc-Br11-E2g8;s Tc-Br11-E2g8 sp003552885
Gb0053562MAG042	No	87.7	1.52	2304749	506	5387	52	90.87	2842	d	Archaea;p	Halobacteriota;c Halobacteria;o Halobacteriales;f Haloferacaceae;g Halalkalirubrum;s Halalkalirubrum sp003551725
Gb0053562MAG043	Yes	80.94	3.7	2704341	669	4633	64	88.32	2781	d	Bacteria;p	Planctomycetota;c Phycisphaerae;o SM23-33;f SM23-33;g PUND01;s PUND01 sp003551645
Gb0053562MAG044	No	98.25	8.77	1450020	44	125389	56.3	91.4	1444	d	Bacteria;p	Firmicutes;c Bacilli;o Izemoplasmatales;f Izemoplasmataceae;g T1SED10-81;s T1SED10-81 sp003551005
Gb0053562MAG045	Yes	82.29	6.54	2643185	650	4565	42.6	88.47	2741	d	Bacteria;p	Bacteroidota;c Bacteroidia;o Bacteroidales;f PUMT01;g PUMT01;s PUMT01 sp003551495
Gb0053562MAG047	No	89.74	6.98	2870566	461	12481	75.1	90.81	2857	d	Bacteria;p	Actinobacteriota;c Actinomycetia;o Euzebyales;f Egibacteraceae;g PUKE01;s PUKE01 sp003550425
Gb0053562MAG049	No	71.83	0	1913337	597	3453	57.1	89.85	2170	d	Bacteria;p	Planctomycetota;c Phycisphaerae;o Sedimentisphaerales;f Anaerohalosphaeraceae;g T3Sed10-213;s T3Sed10-213 sp007121565
Gb0053562MAG050	No	91.22	5.96	3264610	733	7241	51.9	89.42	2940	d	Bacteria;p	Bacteroidota;c Bacteroidia;o Bacteroidales;f UBA7960;g PUPG01;s PUPG01 sp003553005
Gb0053562MAG052	Yes	80.15	1.72	762226	138	6916	39.4	91.05	871	d	Bacteria;p	Patescibacteria;c Paceibacteria;o Paceibacterales;f PWPS01;g PWPS01;s PWPS01 sp003551845
Gb0053562MAG054	No	86.31	1.18	2061673	724	3405	47.7	89.1	2538	d	Bacteria;p	Thermotogota;c Thermotogae;o Petrotogales;f Kosmotogaceae;g UBA9910;s UBA9910 sp003561215
Gb0053562MAG055	Yes	77.89	7.73	1874205	481	4522	57.6	87.89	2204	d	Bacteria;p	Chloroflexota;c Dehalococcoidia;o GIF9;f AB-539-J10;g SKVJ01;s SKVJ01 sp003552125
Gb0053562MAG057	Yes	78.38	1.72	1946174	222	12511	59.1	90.32	2263	d	Archaea;p	Halobacteriota;c Halobacteria;o Halobacteriales;f Haloferacaceae;g Halohasta;s
Gb0053562MAG059	No	80.66	1.72	3515072	970	4258	55.5	86.97	3883	d	Bacteria;p	Firmicutes G;c Limnochordia;o DTU010;f B1SED10-159;g B1SED10-159;s B1SED10-159 sp003551965
Gb0053562MAG063	No	85.25	3.57	2285215	483	5673	57.6	89.15	2754	d	Archaea;p	Halobacteriota;c Halobacteria;o Halobacteriales;f Haloferacaceae;g Halohasta;s Halohasta sp001563795
Gb0053562MAG064	No	91.93	8.2	2616154	670	4574	74.3	91.22	2954	d	Bacteria;p	Actinobacteriota;c Actinomycetia;o Euzebyales;f Egibacteraceae;g T1Sed10-49;s T1Sed10-49 sp003553795
Gb0053562MAG069	Yes	78.93	2.31	1967099	539	4081	56.6	86.51	2241	d	Bacteria;p	Firmicutes E;c SLMV01;o SLMV01;f PUMD01;g PUMD01;s PUMD01 sp003551215
Gb0053562MAG070	No	73.37	4.24	1765932	501	4034	42.3	86.31	1976	d	Bacteria;p	Firmicutes D;c Dethiobacteria;o DTU022;f PWGO01;g B1SED10-75;s B1SED10-75 sp003550565
Gb0053562MAG071	No	72	3.2	1800373	105	25576	46.9	93.72	1653	d	Archaea;p	Thermoplasmatota;c Thermoplasmata;o PWKY01;f PWKY01;g PWKY01;s PWKY01 sp003553085
Gb0053562MAG072	No	83.2	9.27	3678361	385	12893	57.7	87.82	4096	d	Archaea;p	Halobacteriota;c Halobacteria;o Halobacteriales;f Haloferacaceae;g Halohasta;s Halohasta sp003554115
Gb0053562MAG073	No	75.84	3.95	2658946	1222	2409	59	92.35	3682	d	Bacteria;p	Proteobacteria;c Alphaproteobacteria;o Rhodobacterales;f Rhodobacteraceae;g Yoonia;s Yoonia sp003551805
Gb0053562MAG114	Yes	88.02	2	2491030	122	29337	62.3	93.4	2202	d	Bacteria;p	Spirochaetota;c Spirochaetia;o DSM-27196;f SLAA01;g B1Sed10-166;s B1Sed10-166 sp003552185
Gb0053563-1MAG001	Yes	96.57	1.31	1267973	46	44443	42.5	87.19	1349	d	Archaea;p	Halobacteriota;c Methanonatronarchaeia;o Methanonatronarchaeales;f Methanonatronarchaeaceae;g Methanonatronarchaeum;s Methanonatronarchaeum sp004212035
Gb0053563-1MAG002	No	93.64	3.39	1837583	100	36104	43.6	90.18	1907	d	Bacteria;p	Firmicutes D;c Dethiobacteria;o SKNC01;f SKNC01;g SKLX01;s SKLX01 sp007121375
Gb0053563-1MAG003	Yes	98.78	0.07	2695555	123	35552	66.1	89.14	2763	d	Archaea;p	Halobacteriota;c Halobacteria;o Halobacteriales;f Haloferacaceae;g Halorubrum;s Halorubrum sp003554605
Gb0053563-1MAG004	No	99.48	3.05	3073391	187	28724	66.2	86.97	3029	d	Bacteria;p	Proteobacteria;c Gammaproteobacteria;o Ectothiorhodospirales;f Thioalkalivibrionaceae;g Thioalkalivibrio B;s Thioalkalivibrio B sp003563455
Gb0053563-1MAG005	No	94.89	1.61	3271830	264	21808	35.3	84.59	2795	d	Bacteria;p	Bacteroidota;c Bacteroidia;o Bacteroidales;f UBA7960;g PUKZ01;s
Gb0053563-1MAG006	Yes	97.21	0	1419028	44	155894	49.1	91.87	1398	d	Bacteria;p	Firmicutes;c Bacilli;o Izemoplasmatales;f Izemoplasmataceae;g T1SED10-81;s T1SED10-81 sp003554025
Gb0053563-1MAG007	Yes	98.67	0	1264108	17	125324	56.9	91.93	1241	d	Bacteria;p	Firmicutes;c Bacilli;o Izemoplasmatales;f Izemoplasmataceae;g T1SED10-81;s T1SED10-81 sp003551005

Gb0053563-1MAG008	Yes	97.22	4.38	2236756	64	108783	42.6	86.86	1935	d Bacteria;p Firmicutes D;c Dethiobacteria;o DTU022;f PWGO01;g B1SED10-75;s B1SED10-75 sp003550565
Gb0053563-1MAG009	Yes	98.67	0	1480699	46	84565	52.4	91.79	1458	d Bacteria;p Firmicutes;c Bacilli;o Izemoplasmatales;f Izemoplasmataceae;g T1SED10-81;s T1SED10-81 sp003553945
Gb0053563-1MAG010	Yes	97.81	3.01	3887868	257	26575	54	89.06	3344	d Bacteria;p Bacteroidota;c Rhodothermia;o Balneolales;f Natronogracilivirgulaceae;g SKNL01;s SKNL01 sp007120555
Gb0053563-1MAG011	Yes	97.54	1.91	4261496	489	22480	45.2	85.66	4144	d Bacteria;p Bacteroidota;c Rhodothermia;o Balneolales;f Balneolaceae;g QGGB01;s QGGB01 sp007118525
Gb0053563-1MAG012	Yes	99.08	0.61	3744098	207	63471	59.7	90.77	3805	d Bacteria;p Proteobacteria;c Alphaproteobacteria;o Rhodobacterales;f Rhodobacteraceae;g Roseinatronobacter;s Roseinatronobacter monicus
Gb0053563-1MAG013	Yes	95.55	4.31	2382335	201	29974	41.1	89.11	2423	d Bacteria;p Firmicutes D;c Dethiobacteria;o SKNC01;f SKNC01;g PWHX01;s PWHX01 sp003555415
Gb0053563-1MAG014	No	98.26	4.03	3217118	312	16427	66.1	90.46	3196	d Bacteria;p Proteobacteria;c Gammaproteobacteria;o Pseudomonadales;f Halomonadaceae;g Halomonas;s Halomonas sp003553625
Gb0053563-1MAG015	No	93.83	9.68	2936704	182	45320	48.3	88.28	2834	d Bacteria;p Firmicutes F;c Halanaerobiia;o B1SED10-174;f B1SED10-174;g T1SED10-26;s T1SED10-26 sp003554145
Gb0053563-1MAG016	No	95.63	2.14	2716806	311	13006	74	90.54	2678	d Bacteria;p Actinobacteriota;c Actinomycetia;o Euzebyales;f Egibacteraceae;g PUKE01;s PUKE01 sp003553565
Gb0053563-1MAG017	Yes	98.68	0.44	3103291	162	78728	39	89.09	2949	d Bacteria;p Firmicutes F;c Halanaerobiia;o Halanaerobiales;f Halarsenatibacteraceae;g T1SED10-84;s T1SED10-84 sp003553485
Gb0053563-1MAG018	Yes	94.76	2.4	2688511	122	34765	42.8	89.42	2260	d Bacteria;p Bacteroidota;c Bacteroidia;o Bacteroidales;f PUMT01;g PUMT01;s PUMT01 sp003554865
Gb0053563-1MAG019	No	80.42	0.7	1683228	44	90540	41.6	91.11	1683	d Bacteria;p Firmicutes A;c Clostridia;o Peptostreptococcales;f T1SED10-28;g T1SED10-28;s T1SED10-28 sp003554105
Gb0053563-1MAG020	Yes	87.85	0	678433	26	50104	43.4	92.53	777	d Archaea;p Aenigmatarchaeota;c Aenigmatarchaeia;o PWEA01;f PWEA01;g PWEA01;s PWEA01 sp003554845
Gb0053563-1MAG021	No	82.45	1.31	1481205	234	8987	34.3	86.8	1633	d Archaea;p Halobacteriota;c Methanosarcinia;o Methanosarcinales;f Methanosarcinaceae;g Methanosalsum;s Methanosalsum natronophilum
Gb0053563-1MAG022	Yes	78.29	0	820600	34	66284	33.2	93.04	840	d Bacteria;p Patescibacteria;c Dojkabacteria;o SC72;f T1SED10-24;g T1SED10-24;s T1SED10-24 sp003554185
Gb0053563-1MAG024	Yes	82.89	1.72	617546	26	37040	40.4	91.19	659	d Bacteria;p Patescibacteria;c Paceibacteria;o Paceibacterales;f PWPS01;g PWHW01;s PWHW01 sp003555075
Gb0053563-1MAG025	No	79.42	0	1720893	394	5334	41.8	93.87	1901	d Bacteria;p CG03;c SLGR01;o SLGR01;f SLGR01;g PWEK01;s PWEK01 sp003553445
Gb0053563-1MAG026	Yes	96.05	0.53	2789032	104	71295	39.3	85.85	2417	d Bacteria;p Firmicutes F;c Halanaerobiia;o Halanaerobiales;f CSSED10-376;g T1SED10-99;s T1SED10-99 sp003553225
Gb0053563-1MAG027	No	92.08	4.13	3241489	487	10032	47.3	87.73	3058	d Bacteria;p Bacteroidota;c Rhodothermia;o Balneolales;f PXAI01;g Cyclonatronum;s Cyclonatronum sp003555145
Gb0053563-1MAG028	Yes	97.9	1.75	2737891	110	62653	42	87.14	2678	d Bacteria;p Firmicutes A;c Clostridia;o Peptostreptococcales;f T1SED10-28;g Isachenkonia;s Isachenkonia sp003553525
Gb0053563-1MAG029	Yes	95.63	3.42	3628000	203	34612	71.8	88.58	3466	d Bacteria;p Actinobacteriota;c Actinomycetia;o Nitriliruptorales;f Nitriliruptoraceae;g T1Sed10-7;s T1Sed10-7 sp003554005
Gb0053563-1MAG030	No	93.42	1.32	2296332	104	44375	49.5	91.3	2231	d Bacteria;p Firmicutes F;c Halanaerobiia;o Halanaerobiales;f Halarsenatibacteraceae;g Halarsenatibacter;s
Gb0053563-1MAG031	Yes	78.58	1.72	647316	17	70044	40.9	93.43	708	d Bacteria;p Patescibacteria;c Paceibacteria;o Paceibacterales;f PWPS01;g T1SED10-78;s T1SED10-78 sp003553505
Gb0053563-1MAG034	No	81.17	1.72	1018664	206	8243	46	84.39	1120	d Bacteria;p Patescibacteria;c Paceibacteria;o UBA9983 A;f CSBR16-193;g ;s
Gb0053563-1MAG035	No	87.85	0	733314	31	39842	46	92.6	840	d Archaea;p Aenigmatarchaeota;c Aenigmatarchaeia;o PWEA01;f PWEA01;g PWEA01;s PWEA01 sp003555465
Gb0053563-1MAG036	Yes	80.46	0	670985	46	23496	40.6	89.22	730	d Bacteria;p Patescibacteria;c Paceibacteria;o Paceibacterales;f PWPS01;g PWPS01;s PWPS01 sp003554915
Gb0053563-1MAG037	No	90.69	1.31	1201140	97	20704	34.9	79.05	1235	d Archaea;p Halobacteriota;c Methanonatronarchaeia;o Methanonatronarchaeales;f ;g ;s
Gb0053563-1MAG038	No	75.68	0.56	693699	28	28587	30.7	92.45	705	d Bacteria;p Patescibacteria;c ABY1;o BM507;f UBA12465;g PWHG01;s PWHG01 sp003554785
Gb0053563-1MAG039	No	98.25	4.9	2437699	212	22071	35.8	90.85	2491	d Bacteria;p Firmicutes F;c Halanaerobiia;o Halanaerobiales;f Halarsenatibacteraceae;g SLSL01;s SLSL01 sp007130465
Gb0053563-1MAG040	No	94.19	2.78	3029137	320	13704	54.5	87.72	2804	d Bacteria;p Desulfobacterota;c Desulfobacteria;o Desulfobacterales;f SURF-3;g B1SED10-16;s B1SED10-16 sp003551985
Gb0053563-1MAG041	No	85.47	2.56	2459042	862	3547	73.9	90.6	2993	d Bacteria;p Actinobacteriota;c Actinomycetia;o Euzebyales;f Egibacteraceae;g T1Sed10-49;s T1Sed10-49 sp003553795
Gb0053563-1MAG042	Yes	96.77	1.61	1513191	146	20398	61.9	91.31	1662	d Archaea;p Thermoplasmatota;c Thermoplasmata;o Methanomassiliicoccales;f Methanomethylophilaceae;g PWHV01;s PWHV01 sp003555025
Gb0053563-1MAG043	No	81.99	3.83	2391031	117	40343	47.3	87.63	2210	d Bacteria;p Firmicutes D;c Dethiobacteria;o DTU022;f DTU022;g B1SED10-74M;s
Gb0053563-1MAG044	Yes	93.35	8.11	2821698	214	22655	45	81.48	2524	d Bacteria;p Firmicutes F;c Halanaerobiia;o Halanaerobiales;f Halarsenatibacteraceae;g T1SED10-84;s T1SED10-84 sp003554225
Gb0053563-1MAG045	Yes	84.58	0	604805	53	18798	39.5	89.07	744	d Archaea;p B1Sed10-29;c B1Sed10-29;o B1Sed10-29;f B1Sed10-29;g B1Sed10-29;s
Gb0053563-1MAG046	No	79.51	5.11	1570341	431	4826	44.2	81.84	1995	d Archaea;p Hadarchaeota;c Hadarchaeia;o ;f ;g ;s
Gb0053563-1MAG047	Yes	98.31	0.15	2060014	156	18453	48.4	86.72	1916	d Bacteria;p T1Sed10-126;c T1Sed10-126;o T1Sed10-126;f T1Sed10-126;g T1Sed10-126;s T1Sed10-126 sp003554945
Gb0053563-1MAG048	Yes	86.57	9.52	4124756	887	5621	55.5	86.89	4157	d Bacteria;p Desulfobacterota;c Desulfobacteria;o Desulfobacterales;f SURF-3;g B1SED10-16;s
Gb0053563-1MAG049	No	91.57	5.81	3243009	492	10662	34.6	84.92	3102	d Bacteria;p Bacteroidota;c Bacteroidia;o Bacteroidales;f UBA7960;g PUKZ01;s PUKZ01 sp003550725
Gb0053563-1MAG050	No	85.1	7.4	2925130	570	6443	49.9	90.26	2956	d Bacteria;p Bacteroidota;c Rhodothermia;o Balneolales;f Natronogracilivirgulaceae;g Natronogracilivirgula;s Natronogracilivirgula saccharolytica
Gb0053563-1MAG052	No	79.91	0	603169	14	75015	42.1	92.04	679	d Archaea;p Aenigmatarchaeota;c Aenigmatarchaeia;o PWEA01;f PWEA01;g PWEA01;s PWEA01 sp003553905
Gb0053563-1MAG054	No	78.5	4.67	711800	30	102500	44.5	92.94	776	d Archaea;p Aenigmatarchaeota;c Aenigmatarchaeia;o PWEA01;f PWEA01;g PWEA01;s PWEA01 sp003550625
Gb0053563-1MAG055	Yes	83.64	0	641918	55	17355	38.5	89.35	766	d Archaea;p B1Sed10-29;c B1Sed10-29;o B1Sed10-29;f B1Sed10-29;g B1Sed10-29;s B1Sed10-29 sp003553825
Gb0053563-1MAG056	Yes	93.2	2	2018366	174	18485	46.2	92.61	2048	d Archaea;p Thermoplasmatota;c Thermoplasmata;o PWKY01;f PWKY01;g PWKY01;s PWKY01 sp003553085
Gb0053563-1MAG057	No	82.19	3.25	2341370	143	39725	44	88.59	2327	d Bacteria;p Firmicutes F;c Halanaerobiia;o B1SED10-174;f B1SED10-174;g T1SED10-26;s T1SED10-26 sp007122335
Gb0053563-1MAG058	No	86.45	6.07	669151	60	18488	33	90.39	790	d Archaea;p B1Sed10-29;c B1Sed10-29;o B1Sed10-29;f B1Sed10-29;g B1Sed10-29;s B1Sed10-29 sp003551125
Gb0053563-1MAG059	No	88.42	9.06	2558580	580	5746	59.5	89.25	3232	d Archaea;p Halobacteriota;c Halobacteria;o Halobacteriales;f Haloferacaceae;g Halohasta;s Halohasta sp003554115
Gb0053563-1MAG060	No	77.53	1.91	3309389	622	10440	44.5	86.69	3722	d Bacteria;p Firmicutes D;c Dethiobacteria;o DTU022;f DTU022;g T3SED10-7;s T3SED10-7 sp003564505
Gb0053563-1MAG062	No	81.41	4.45	2711224	569	5703	48.5	86.27	2882	d Bacteria;p Firmicutes D;c Dethiobacteria;o DTU022;f UBA8154;g SKMY01;s
Gb0053563-1MAG063	No	77.58	3.9	2447064	667	4321	68.1	87.28	2957	d Archaea;p Halobacteriota;c Halobacteria;o Halobacteriales;f Natrialbaceae;g Tc-Br11-E2g8;s Tc-Br11-E2g8 sp001564115
Gb0053563-1MAG064	No	90.55	4.56	1575450	166	13396	48.6	91.48	1686	d Bacteria;p Firmicutes;c Bacilli;o Izemoplasmatales;f Izemoplasmataceae;g B1SED10-225;s B1SED10-225 sp003554735
Gb0053563-1MAG065	Yes	89.35	6.43	2892023	376	12730	44.2	87.02	3056	d Bacteria;p Firmicutes F;c Halanaerobiia;o B1SED10-174;f B1SED10-174;g T1SED10-26;s T1SED10-26 sp003554585
Gb0053563-1MAG067	No	71.67	0.67	1010316	296	3869	41.9	91.38	1274	d Bacteria;p Firmicutes;c Bacilli;o Izemoplasmatales;f Izemoplasmataceae;g B1SED10-225;s
Gb0053563-1MAG069	Yes	88.8	3.2	2565212	252	24578	44.4	91.97	2308	d Archaea;p Thermoplasmatota;c Thermoplasmata;o PWKY01;f PWKY01;g Natronoplasma;s Natronoplasma sp003555395
Gb0053563-1MAG073	No	72.8	5.94	1655040	151	18296	40.5	92.18	1741	d Archaea;p Thermoplasmatota;c Thermoplasmata;o PWKY01;f PWKY01;g B1SED10-34;s
Gb0053563-1MAG074	No	75.74	2.84	2045357	720	3297	47.4	89.05	2486	d Bacteria;p Planctomycetota;c Phycisphaerae;o Sedimentisphaerales;f SG8-4;g T1Sed10-97;s T1Sed10-97 sp003553245
Gb0053563-1MAG079	No	87.28	9.06	3034228	448	9856	44.5	82.63	2916	d Bacteria;p Firmicutes F;c Halanaerobiia;o Halanaerobiales;f Halarsenatibacteraceae;g T1SED10-84;s T1SED10-84 sp007134885
Gb0053563-1MAG082	Yes	91.38	6.79	1791445	320	7857	50.1	91.69	1966	d Bacteria;p Firmicutes;c Bacilli;o Izemoplasmatales;f Izemoplasmataceae;g B1SED10-225;s B1SED10-225 sp003555335
Gb0053563-1MAG084	No	88.21	2.35	1621589	207	10899	64.8	95.03	1737	d Bacteria;p Proteobacteria;c Gammaproteobacteria;o Nitrococcales;f Nitrococcaceae;g Spiribacter;s Spiribacter sp009676705
Gb0053563-1MAG085	No	76.14	5.69	2832858	557	8745	50.3	91.38	2661	d Bacteria;p Bacteroidota;c Bacteroidia;o Bacteroidales;f UBA7960;g PYGY01;s PYGY01 sp007129945
Gb0053563-1MAG090	No	84.38	4.99	2815120	674	5084	63.8	84.52	3302	d Archaea;p Halobacteriota;c Halobacteria;o Halobacteriales;f Natrialbaceae;g Tc-Br11;s Tc-Br11 sp001564275
Gb0053563-2MAG001	No	95.92	1.31	1238690	43	56139	42.5	87	1322	d Archaea;p Halobacteriota;c Methanonatronarchaeia;o Methanonatronarchaeales;f Methanonatronarchaeaceae;g Methanonatronarchaeum;s Methanonatronarchaeum sp004212035
Gb0053563-2MAG002	No	99.36	3.65	3159634	224	24303	66.1	86.99	3135	d Bacteria;p Proteobacteria;c Gammaproteobacteria;o Ectothiorhodospirales;f Thioalkalivibrionaceae;g Thioalkalivibrio B;s Thioalkalivibrio B sp003563455
Gb0053563-2MAG003	No	94.35	2.69	3255424	270	20964	35.5	84.8	2792	d Bacteria;p Bacteroidota;c Bacteroidia;o Bacteroidales;f UBA7960;g PUKZ01;s
Gb0053563-2MAG004	No	98.67	1.33	1315855	16	142906	56.6	91.89	1294	d Bacteria;p Firmicutes;c Bacilli;o Izemoplasmatales;f Izemoplasmataceae;g T1SED10-81;s T1SED10-81 sp003551005
Gb0053563-2MAG005	No	98.67	0	1475302	49	95185	52.1	91.77	1446	d Bacteria;p Firmicutes;c Bacilli;o Izemoplasmatales;f Izemoplasmataceae;g T1SED10-81;s T1SED10-81 sp003553945
Gb0053563-2MAG006	No	95.52	4.24	2305330	70	92218	42.6	86.67	2020	d Bacteria;p Firmicutes D;c Dethiobacteria;o DTU022;f PWGO01;g B1SED10-75;s B1SED10-75 sp003550565
Gb0053563-2MAG007	No	97.21	0	1448558	47	86995	49.1	92.48	1434	d Bacteria;p Firmicutes;c Bacilli;o Izemoplasmatales;f Izemoplasmataceae;g T1SED10-81;s T1SED10-81 sp003554025
Gb0053563-2MAG008	No	90.95	2.38	2692695	82	56945	42.8	89.25	2232	d Bacteria;p Bacteroidota;c Bacteroidia;o Bacteroidales;f PUMT01;g PUMT01;s PUMT01 sp003554865
Gb0053563-2MAG009	No	95.63	3.42	3354582	194	35364	71.8	88.86	3194	d Bacteria;p Actinobacteriota;c Actinomycetia;o Nitriliruptorales;f Nitriliruptoraceae;g T1Sed10-7;s T1Sed10-7 sp003554005
Gb0053563-2MAG010	No	95.63	1.91	3438423	198	29557	54.5	89.45	2915	d Bacteria;p Bacteroidota;c Rhodothermia;o Balneolales;f Natronogracilivirgulaceae;g SKNL01;s SKNL01 sp007120555
Gb0053563-2MAG011	Yes	93.83	5.08	4455809	605	12965	41.6	83.53	4460	d Bacteria;p Firmicutes D;c Dethiobacteria;o DTU022;f PWGO01;g T1SED10-157;s T1SED10-157 sp003554535
Gb0053563-2MAG012	No	98.98	0.98	3720195	245	48983	59.9	90.94	3810	d Bacteria;p Proteobacteria;c Alphaproteobacteria;o Rhodobacterales;f Rhodobacteraceae;g Roseinatronobacter;s Roseinatronobacter monicus
Gb0053563-2MAG013	Yes	99.15	2.56	3216891	279	23515	74.5	90.35	3157	d Bacteria;p Actinobacteriota;c Actinomycetia;o Euzebyales;f Egibacteraceae;g T1Sed10-49;s T1Sed10-49 sp003553795
Gb0053563-2MAG014	Yes	89.72	0.47	931709	114	27135	46.3	92.2	1106	d Archaea;p Aenigmatarchaeota;c Aenigmatarchaeia;o PWEA01;f PWEA01;g PWEA01;s PWEA01 sp003555465
Gb0053563-2MAG015	Yes	93.95	1.31	1649758	158	20064	34.6	81.24	1759	d Archaea;p Halobacteriota;c Methanonatronarchaeia;o Methanonatronarchaeales;f ;g ;s
Gb0053563-2MAG016	No	95.7	2.59	3003002	331	13289	68.5	87.58	3197	d Archaea;p Halobacteriota;c Halobacteria;o Halobacteriales;f Natrialbaceae;g Tc-Br11-E2g8;s Tc-Br11-E2g8 sp001564115
Gb0053563-2MAG017	No	98.2	1.28	2731398	138	46742	74	90.46	2546	d Bacteria;p Actinobacteriota;c Actinomycetia;o Euzebyales;f Egibacteraceae;g PUKE01;s PUKE01 sp003553565
Gb0053563-2MAG018	No	79.03	1.13	1661357	138	36602	41	88.79	1714	d Bacteria;p Firmicutes D;c Dethiobacteria;o SKNC01;f SKNC01;g PWHX01;s PWHX01 sp003555415
Gb0053563-2MAG019	Yes	93.86	3.39	1771846	98	30554	43.9	90.56	1811	d Bacteria;p Firmicutes D;c Dethiobacteria;o SKNC01;f SKNC01;g SKLX01;s SKLX01 sp007121375
Gb0053563-2MAG020	Yes	93.83	4.1	2687529	155	39520	48.6	88.16	2566	d Bacteria;p Firmicutes F;c Halanaerobiia;o B1SED10-174;f B1SED10-174;g T1SED10-26;s T1SED10-26 sp003554145
Gb0053563-2MAG021	No	93.42	0.44	3509841	408	49594	39	87.7	3575	d Bacteria;p Firmicutes F;c Halanaerobiia;o Halanaerobiales;f Halarsenatibacteraceae;g T1SED10-84;s T1SED10-84 sp003553485
Gb0053563-2MAG022	No	95.16	2.69	2793511	198	27929	34.6	85.24	2331	d Bacteria;p Bacteroidota;c Bacteroidia;o Bacteroidales;f UBA7960;g PUKZ01;s PUKZ01 sp003550725
Gb0053563-2MAG023	No	97.54	2.46	4024188	329	19583	45	86.66	3680	d Bacteria;p Bacteroidota;c Rhodothermia;o Balneolales;f Balneolaceae;g QGGB01;s QGGB01 sp007118525
Gb0053563-2MAG024	No	82.52	1.4	2110208	105	75624	42.2	90.09	2120	d Bacteria;p Firmicutes A;c Clostridia;o Peptostreptococcales;f T1SED10-28;g T1SED10-28;s T1SED10-28 sp003554105
Gb0053563-2MAG025	No	97.33	4.67	1434213	167	14280	42.3	91.92	1552	d Bacteria;p Firmicutes;c Bacilli;o Izemoplasmatales;f Izemoplasmataceae;g B1SED10-225;s B1SED10-225 sp003551405
Gb0053563-2MAG026	No	87.85	1.94	1283497	178	22438	43.8	92.68	1662	d Archaea;p Aenigmatarchaeota;c Aenigmatarchaeia;o PWEA01;f PWEA01;g PWEA01;s PWEA01 sp003554845
Gb0053563-2MAG027	Yes	82.17	0	629932	121	6132	43.8	92.67	781	d Archaea;p Aenigmatarchaeota;c Aenigmatarchaeia;o PWEA01;f PWEA01;g PWEA01;s PWEA01 sp003554235
Gb0053563-2MAG028	No	90.32	0.81	1320700	26	81666	62	91.33	1380	d Archaea;p Thermoplasmatota;c Thermoplasmata;o Methanomassiliicoccales;f Methanomethylophilaceae;g PWHV01;s PWHV01 sp003555025
Gb0053563-2MAG029	No	96.82	3.73	2818158	135	35124	65.9	88.98	2870	d Archaea;p Halobacteriota;c Halobacteria;o Halobacteriales;f Haloferacaceae;g Halorubrum;s Halorubrum sp003554605

Gb0053563-2MAG030	No	82.89	5.17	662764	29	97518	40.3	90.76	702	d	Bacteria;p Patescibacteria;c Paceibacteria;o Paceibacterales;f PWPS01;g PWHW01;s PWHW01 sp003555075
Gb0053563-2MAG032	Yes	89.59	3.67	2371783	169	21939	46.6	85.48	2214	d	Bacteria;p Firmicutes D;c Dethiobacteria;o DTU022;f PWGO01;g PWGO01;s PWGO01 sp003554525
Gb0053563-2MAG033	No	96.05	0.53	2950908	129	51311	39.3	86.6	2546	d	Bacteria;p Firmicutes F;c Halanaerobiia;o Halanaerobiales;f CSSED10-376;g T1SED10-99;s T1SED10-99 sp003553225
Gb0053563-2MAG034	Yes	92.88	0	1954608	102	36291	42.3	94.38	1760	d	Bacteria;p CG03;c SLGR01;o SLGR01;f SLGR01;g PWEK01;s PWEK01 sp003553445
Gb0053563-2MAG035	Yes	96.93	1.54	2458580	128	36078	49.4	91.23	2408	d	Bacteria;p Firmicutes F;c Halanaerobiia;o Halanaerobiales;f Halarsenatibacteraceae;g Halarsenatibacter;s
Gb0053563-2MAG036	Yes	98.86	0.64	2296134	189	18347	47.5	88.54	2202	d	Bacteria;p Planctomycetota;c Phycisphaerae;o Sedimentisphaerales;f SG8-4;g T1Sed10-97;s T1Sed10-97 sp003553245
Gb0053563-2MAG037	Yes	94.54	2.46	3300370	358	14286	47.5	87.81	2911	d	Bacteria;p Bacteroidota;c Rhodothermia;o Balneolales;f PXAI01;g Cyclonatronum;s Cyclonatronum sp003555145
Gb0053563-2MAG038	No	92.9	6.96	3373475	419	10386	53.7	86.96	3308	d	Bacteria;p Desulfobacterota;c Desulfobacteria;o Desulfobacterales;f SURF-3;g B1SED10-16;s B1SED10-16 sp003551985
Gb0053563-2MAG039	Yes	99.19	2.29	3143835	192	25793	55.6	86.09	2888	d	Bacteria;p Desulfobacterota;c Syntrophobacteria;o Syntrophobacterales;f Syntrophobacteraceae;g SLCH01;s SLCH01 sp003566995
Gb0053563-2MAG040	No	78.58	1.72	702298	19	59567	40.5	93.94	786	d	Bacteria;p Patescibacteria;c Paceibacteria;o Paceibacterales;f PWPS01;g T1SED10-78;s T1SED10-78 sp003553505
Gb0053563-2MAG043	No	96.61	0	2089675	71	51638	48.6	86.96	1843	d	Bacteria;p T1Sed10-126;c T1Sed10-126;o T1Sed10-126;f T1Sed10-126;g T1Sed10-126;s T1Sed10-126 sp003554945
Gb0053563-2MAG044	Yes	97.6	6.4	4784104	452	17860	61.3	87.35	4402	d	Bacteria;p Spirochaetota;c Spirochaetia;o DSM-27196;f SKKC01;g PWGF01;s PWGF01 sp003554375
Gb0053563-2MAG045	Yes	98.38	2.74	3276599	275	21516	66.1	90.61	3210	d	Bacteria;p Proteobacteria;c Gammaproteobacteria;o Pseudomonadales;f Halomonadaceae;g Halomonas;s Halomonas sp003553625
Gb0053563-2MAG046	Yes	86	7.47	1734249	87	31683	44.5	82.34	1844	d	Archaea;p Hadarchaeota;c Hadarchaeia;o ;f ;g ;s
Gb0053563-2MAG047	No	78.29	0	1641894	234	15421	33.3	89.57	2027	d	Bacteria;p Patescibacteria;c Dojkabacteria;o SC72;f T1SED10-24;g T1SED10-24;s T1SED10-24 sp003554185
Gb0053563-2MAG048	No	97.18	1.55	3555421	316	19018	62.6	88.24	3270	d	Bacteria;p Bacteroidota;c Rhodothermia;o Rhodothermales;f Salinibacteraceae;g Tc-Br11-B2g6-7;s Tc-Br11-B2g6-7 sp001564055
Gb0053563-2MAG049	Yes	93.21	1.37	3650869	528	8835	55.6	86.67	3639	d	Bacteria;p Firmicutes G;c Limnochordia;o DTU010;f B1SED10-159;g B1SED10-159;s B1SED10-159 sp003551965
Gb0053563-2MAG050	Yes	93.07	2.97	2616154	284	13311	58.6	86.35	2684	d	Bacteria;p Firmicutes E;c SLMV01;o SLMV01;f PUMD01;g PWGR01;s PWGR01 sp003554565
Gb0053563-2MAG051	No	96	2.79	1340296	130	19584	54.7	92.12	1390	d	Bacteria;p Firmicutes;c Bacilli;o Izemoplasmatales;f Izemoplasmataceae;g T1SED10-81;s T1SED10-81 sp003568315
Gb0053563-2MAG052	No	89.25	5.81	2517956	287	13593	47.6	90.7	2243	d	Bacteria;p Bacteroidota;c Bacteroidia;o Bacteroidales;f UBA7960;g PUPG01;s PUPG01 sp007124575
Gb0053563-2MAG053	Yes	99.47	4	3757340	480	12323	61.2	91.84	3693	d	Bacteria;p Spirochaetota;c Spirochaetia;o DSM-27196;f SLAA01;g B1Sed10-166;s B1Sed10-166 sp007126415
Gb0053563-2MAG054	Yes	93.49	6.78	1979279	288	10472	64.5	94.5	2191	d	Bacteria;p Proteobacteria;c Gammaproteobacteria;o Nitrococcales;f Nitrococcaceae;g Spiribacter;s Spiribacter sp009676705
Gb0053563-2MAG056	Yes	98.5	4.03	3196360	140	34022	64	84.86	3168	d	Archaea;p Halobacteriota;c Halobacteria;o Halobacteriales;f Natrialbaceae;g Tc-Br11;s Tc-Br11 sp001564275
Gb0053563-2MAG057	No	93.2	4	2346236	184	21088	46.2	92.87	2231	d	Archaea;p Thermoplasmatota;c Thermoplasmata;o PWKY01;f PWKY01;g PWKY01;s PWKY01 sp003553085
Gb0053563-2MAG059	No	76.15	2.74	918465	141	10573	46.4	83.26	960	d	Bacteria;p Patescibacteria;c Paceibacteria;o UBA9983 A;f CSBR16-193;g ;s
Gb0053563-2MAG060	No	98.25	7.53	2532221	238	18103	35.9	90.63	2605	d	Bacteria;p Firmicutes F;c Halanaerobiia;o Halanaerobiales;f Halarsenatibacteraceae;g SLSL01;s SLSL01 sp007130465
Gb0053563-2MAG061	No	82.71	0	667715	16	73055	41.7	92.18	770	d	Archaea;p Aenigmatarchaeota;c Aenigmatarchaeia;o PWEA01;f PWEA01;g PWEA01;s PWEA01 sp003553905
Gb0053563-2MAG062	Yes	91.53	1.14	2259004	265	12395	48.1	91.12	2239	d	Bacteria;p Spirochaetota;c Spirochaetia;o PWKH01;f PWKH01;g PWKH01;s
Gb0053563-2MAG063	No	71.26	2.19	2885928	528	9183	44.9	87.15	3275	d	Bacteria;p Firmicutes D;c Dethiobacteria;o DTU022;f DTU022;g T3SED10-7;s T3SED10-7 sp003564505
Gb0053563-2MAG064	No	79.11	2.68	1728229	177	13434	53.4	86.11	1713	d	Bacteria;p Desulfobacterota I;c Desulfovibrionia;o Desulfovibrionales;f Desulfonatronovibrionaceae;g Desulfonatronospira;s Desulfonatronospira sp003554505
Gb0053563-2MAG065	No	94.41	1.75	2785309	131	67987	41.9	86.87	2739	d	Bacteria;p Firmicutes A;c Clostridia;o Peptostreptococcales;f T1SED10-28;g Isachenkonia;s Isachenkonia sp003553525
Gb0053563-2MAG066	No	83.64	9.88	1209331	171	17587	33.1	87.41	1585	d	Archaea;p B1Sed10-29;c B1Sed10-29;o B1Sed10-29;f B1Sed10-29;g B1Sed10-29;s B1Sed10-29 sp003551125
Gb0053563-2MAG067	Yes	90.36	3.3	1554529	217	10555	46.4	88.67	1707	d	Bacteria;p Chloroflexota;c Dehalococcoidia;o Dehalococcoidales;f CSSed11-197;g CSSed11-197;s CSSed11-197 sp003552785
Gb0053563-2MAG068	Yes	92.1	7.19	2714537	159	34636	47.5	87.75	2488	d	Bacteria;p Firmicutes D;c Dethiobacteria;o DTU022;f DTU022;g B1SED10-74M;s
Gb0053563-2MAG069	Yes	94.94	4.07	3985700	640	9345	58.7	91.51	4517	d	Bacteria;p Proteobacteria;c Alphaproteobacteria;o Rhodobacterales;f Rhodobacteraceae;g Yoonia;s Yoonia sp003551805
Gb0053563-2MAG071	No	88.96	7.33	2323764	168	27139	45.6	83.06	2075	d	Bacteria;p Firmicutes F;c Halanaerobiia;o Halanaerobiales;f Halarsenatibacteraceae;g T1SED10-84;s T1SED10-84 sp003554225
Gb0053563-2MAG073	No	84.11	8.88	882579	138	14472	39.3	87.95	1101	d	Archaea;p B1Sed10-29;c B1Sed10-29;o B1Sed10-29;f B1Sed10-29;g B1Sed10-29;s
Gb0053563-2MAG076	Yes	83.52	3.97	2532110	433	7495	49.9	88.48	2336	d	Bacteria;p Bacteroidota;c Bacteroidia;o Bacteroidales;f UBA7960;g PUPG01;s PUPG01 sp003552075
Gb0053563-2MAG077	No	80.12	5.87	2126159	206	24331	44.7	92.13	2029	d	Archaea;p Thermoplasmatota;c Thermoplasmata;o PWKY01;f PWKY01;g Natronoplasma;s Natronoplasma sp003555395
Gb0053563-2MAG078	No	81.78	0.93	841123	115	13544	38.1	89.28	1045	d	Archaea;p B1Sed10-29;c B1Sed10-29;o B1Sed10-29;f B1Sed10-29;g B1Sed10-29;s B1Sed10-29 sp003553825
Gb0053563-2MAG079	No	90.68	5.37	2397964	144	33595	44.3	89.06	2384	d	Bacteria;p Firmicutes F;c Halanaerobiia;o B1SED10-174;f B1SED10-174;g T1SED10-26;s T1SED10-26 sp007122335
Gb0053563-2MAG080	No	85.21	6.21	2825021	192	34597	44.9	88.55	2827	d	Bacteria;p Firmicutes F;c Halanaerobiia;o B1SED10-174;f B1SED10-174;g T1SED10-26;s T1SED10-26 sp003554585
Gb0053563-2MAG081	Yes	95.52	5.34	2308801	249	13360	46.4	84.7	2211	d	Bacteria;p Firmicutes D;c Dethiobacteria;o DTU022;f PWGO01;g ;s
Gb0053563-2MAG087	Yes	80.52	2.25	2594172	477	7105	42.2	87.5	2430	d	Bacteria;p T1SED10-198M;c T1SED10-198M;o T1SED10-198M;f T1SED10-198M;g T1SED10-198M;s T1SED10-198M sp003554345
Gb0053563-2MAG088	No	70.87	0.93	2452236	392	7152	34.2	88	2446	d	Archaea;p Nanoarchaeota;c Nanoarchaeia;o Woesearchaeales;f DSVV01;g ;s
Gb0053563-2MAG099	No	92.29	6.8	3366557	420	10911	62.3	84.48	3646	d	Archaea;p Halobacteriota;c Halobacteria;o Halobacteriales;f Natrialbaceae;g ;s
Gb0053563-2MAG102	Yes	73.83	3.02	3205910	891	4078	49.6	85.7	3298	d	Bacteria;p Bacteroidota;c Rhodothermia;o Balneolales;f PXAI01;g Cyclonatronum;s
Gb0053563-2MAG111	Yes	74.4	0.8	1543338	45	114403	42.4	92.37	1444	d	Archaea;p Thermoplasmatota;c Thermoplasmata;o PWKY01;f PWKY01;g Natronoplasma;s Natronoplasma sp003554965
Gb0053563-2MAG119	No	72.89	2.69	2091731	345	7764	45.5	83.89	2072	d	Bacteria;p Firmicutes F;c Halanaerobiia;o Halanaerobiales;f Halarsenatibacteraceae;g T1SED10-84;s T1SED10-84 sp003554685
Gb0053563-2MAG121	Yes	78.54	7.09	3304893	709	5780	35.8	88.8	3612	d	Bacteria;p Firmicutes F;c Halanaerobiia;o Halanaerobiales;f Halarsenatibacteraceae;g SLSL01;s SLSL01 sp003552825
Gb0053563-2MAG123	Yes	86.89	5.74	3445478	482	9775	49.5	90.02	3211	d	Bacteria;p Bacteroidota;c Rhodothermia;o Balneolales;f Natronogracilivirgulaceae;g SLLW01;s SLLW01 sp007133875
Gb0053563-2MAG124	No	94.43	4.64	3490482	242	91405	50.3	90.37	2996	d	Bacteria;p Bacteroidota;c Rhodothermia;o Balneolales;f Natronogracilivirgulaceae;g Natronogracilivirgula;s Natronogracilivirgula saccharolytica
Gb0053563-2MAG140	Yes	80.51	0.87	2649528	526	6095	44	87.89	2820	d	Bacteria;p Firmicutes A;c Clostridia;o Peptostreptococcales;f T1SED10-28;g Isachenkonia;s Isachenkonia alkalipeptolytica
Gb0054152-2MAG001	No	93.2	1.12	1740433	130	24080	55.2	96.3	1835	d	Bacteria;p Proteobacteria;c Gammaproteobacteria;o Nitrococcales;f Nitrococcaceae;g Spiribacter;s
Gb0054152-2MAG002	No	95.06	6.91	2947972	135	31953	65.8	88.84	3019	d	Archaea;p Halobacteriota;c Halobacteria;o Halobacteriales;f Haloferacaceae;g Halorubrum;s Halorubrum sp003554605
Gb0054152-2MAG003	Yes	95.3	1.54	2087103	295	9924	60.4	88.18	2076	d	Bacteria;p Desulfobacterota;c Desulfobulbia;o Desulfobulbales;f Desulfurivibrionaceae;g Desulfurivibrio;s Desulfurivibrio sp003557565
Gb0054152-2MAG004	No	92.47	3	2924725	346	12473	68.7	87.89	3141	d	Archaea;p Halobacteriota;c Halobacteria;o Halobacteriales;f Natrialbaceae;g Tc-Br11-E2g8;s Tc-Br11-E2g8 sp001564115
Gb0054152-2MAG005	Yes	93.94	2.9	3056904	204	27197	58	88.46	3236	d	Archaea;p Halobacteriota;c Halobacteria;o Halobacteriales;f Haloarculaceae;g Halovenus;s
Gb0054152-2MAG006	No	92.15	1.52	2535998	158	24967	52	90.92	2751	d	Archaea;p Halobacteriota;c Halobacteria;o Halobacteriales;f Haloferacaceae;g Halalkalirubrum;s Halalkalirubrum sp003551725
Gb0054152-2MAG007	No	86.02	3.18	2251842	196	18860	50.5	87.53	2217	d	Bacteria;p Firmicutes D;c Dethiobacteria;o DTU022;f DTU022;g B1SED10-74M;s B1SED10-74M sp003554395
Gb0054152-2MAG008	No	93.32	3.36	3207556	164	34088	63.6	84.85	3185	d	Archaea;p Halobacteriota;c Halobacteria;o Halobacteriales;f Natrialbaceae;g Tc-Br11;s Tc-Br11 sp001564275
Gb0054152-2MAG009	Yes	95.04	9.26	2761575	128	42242	64.3	87.86	2817	d	Archaea;p Halobacteriota;c Halobacteria;o Halobacteriales;f Haloferacaceae;g Halorubrum;s Halorubrum sp001564205
Gb0054152-2MAG010	Yes	95.36	4.01	3215010	356	14753	44.1	88.58	2985	d	Bacteria;p Bacteroidota;c Rhodothermia;o Balneolales;f Balneolaceae;g SW132;s SW132 sp007129085
Gb0054152-2MAG013	No	94.78	4.82	3338424	154	33350	62.3	84.59	3451	d	Archaea;p Halobacteriota;c Halobacteria;o Halobacteriales;f Natrialbaceae;g Natronococcus;s
Gb0054152-2MAG015	Yes	74.07	2.41	751415	120	7416	42.3	91.02	999	d	Archaea;p Nanohaloarchaeota;c Nanosalinia;o Nanosalinales;f Nanosalinaceae;g PL-Br10-U2g16;s PL-Br10-U2g16 sp001563995
Gb0054152-2MAG016	No	83.49	4.2	1760567	116	31003	62.1	88.87	1921	d	Archaea;p Halobacteriota;c Halobacteria;o Halobacteriales;f Haloferacaceae;g PL-Br10-E2g29;s PL-Br10-E2g29 sp001563965
Gb0054152-2MAG017	Yes	85.11	9.68	3113352	532	7610	62.7	88.73	3665	d	Archaea;p Halobacteriota;c Halobacteria;o Halobacteriales;f Haloarculaceae;g Natronomonas;s
Gb0054152-2MAG018	No	90.61	2.19	1934221	184	16199	44.8	82.16	1821	d	Bacteria;p Firmicutes F;c Halanaerobiia;o Halanaerobiales;f Halarsenatibacteraceae;g T1SED10-84;s T1SED10-84 sp007134885
Gb0054152-2MAG021	Yes	88.71	5.04	2456674	268	14479	45	82.61	2306	d	Bacteria;p Firmicutes F;c Halanaerobiia;o Halanaerobiales;f Halarsenatibacteraceae;g T1SED10-84;s T1SED10-84 sp003554685
Gb0054152-2MAG022	No	89.65	3.01	3383337	622	6860	70.9	88.31	3535	d	Bacteria;p Actinobacteriota;c Actinomycetia;o Nitriliruptorales;f Nitriliruptoraceae;g T1Sed10-7;s
Gb0054152-2MAG023	No	87.28	4.73	2940875	258	18731	61.1	88.46	3087	d	Archaea;p Halobacteriota;c Halobacteria;o Halobacteriales;f Natrialbaceae;g Tc-Br11;s Tc-Br11 sp003552325
Gb0054152-2MAG024	Yes	84.31	5.2	2786002	604	6004	67.1	88.61	3251	d	Archaea;p Halobacteriota;c Halobacteria;o Halobacteriales;f Haloferacaceae;g Halorubrum;s
Gb0054152-2MAG026	No	83.47	3.53	2916197	716	4808	68.6	90.77	3353	d	Bacteria;p Proteobacteria;c Alphaproteobacteria;o Rhodobacterales;f Rhodobacteraceae;g Rhodobaculum;s Rhodobaculum sp003550665
Gb0054152-2MAG033	Yes	85.18	6.67	2701638	371	10981	55.6	90.02	2967	d	Archaea;p Halobacteriota;c Halobacteria;o Halobacteriales;f Haloarculaceae;g Halovenus;s
Gb0054153-1MAG001	Yes	98.68	2.69	3274278	318	18008	67.9	90.67	3204	d	Bacteria;p Proteobacteria;c Gammaproteobacteria;o Pseudomonadales;f Halomonadaceae;g Halomonas;s Halomonas sp003552795
Gb0054153-1MAG003	No	96.67	2	1431116	80	35346	42.5	92.02	1478	d	Bacteria;p Firmicutes;c Bacilli;o Izemoplasmatales;f Izemoplasmataceae;g B1SED10-225;s B1SED10-225 sp003551405
Gb0054153-1MAG004	No	81.46	4.05	909552	30	56542	38.8	89.74	1060	d	Archaea;p Nanohaloarchaeota;c Nanosalinia;o Nanosalinales;f Nanosalinaceae;g B1-Br10-U2g21;s
Gb0054153-1MAG005	No	90.36	4.47	3090201	248	21893	68.1	87.66	3267	d	Archaea;p Halobacteriota;c Halobacteria;o Halobacteriales;f Natrialbaceae;g Tc-Br11-E2g8;s Tc-Br11-E2g8 sp003552885
Gb0054153-1MAG006	No	93.86	3.68	2733540	217	22323	51.8	90.33	2968	d	Archaea;p Halobacteriota;c Halobacteria;o Halobacteriales;f Haloferacaceae;g Halalkalirubrum;s Halalkalirubrum sp003551725
Gb0054153-1MAG007	Yes	98.61	3.99	3650825	215	30508	61.8	84.24	3779	d	Archaea;p Halobacteriota;c Halobacteria;o Halobacteriales;f Natrialbaceae;g Natronococcus;s
Gb0054153-1MAG008	Yes	85.9	0	974826	41	97137	40.8	91.51	1144	d	Archaea;p Nanohaloarchaeota;c Nanosalinia;o Nanosalinales;f Nanosalinaceae;g B1-Br10-U2g19;s B1-Br10-U2g19 sp001563905
Gb0054153-1MAG009	No	92.72	2.13	2970791	197	27447	54.5	89.44	3123	d	Archaea;p Halobacteriota;c Halobacteria;o Halobacteriales;f Haloarculaceae;g Halovenus;s Halovenus sp003551945
Gb0054153-1MAG010	Yes	96.33	6.27	3535118	282	22610	59.9	88.36	3773	d	Archaea;p Halobacteriota;c Halobacteria;o Halobacteriales;f Natrialbaceae;g Natrarchaeobaculum;s Natrarchaeobaculum sp001563885
Gb0054153-1MAG011	Yes	96.53	2.84	3726086	238	35559	57.8	88.07	4076	d	Archaea;p Halobacteriota;c Halobacteria;o Halobacteriales;f Natrialbaceae;g ;s
Gb0054153-1MAG012	Yes	89.04	1.24	2436216	147	41338	56.8	88.51	2603	d	Archaea;p Halobacteriota;c Halobacteria;o Halobacteriales;f Haloferacaceae;g Halohasta;s Halohasta sp001563795
Gb0054153-1MAG013	Yes	70.17	2.88	779646	61	29072	41.3	91.44	952	d	Archaea;p Nanohaloarchaeota;c Nanosalinia;o Nanosalinales;f Nanosalinaceae;g B1-Br10-U2g19;s
Gb0054153-1MAG015	Yes	85.44	2.18	878049	42	51245	40.1	92.9	1075	d	Archaea;p Nanohaloarchaeota;c Nanosalinia;o Nanosalinales;f Nanosalinaceae;g B1-Br10-U2g21;s B1-Br10-U2g21 sp001564145
Gb0054153-1MAG016	No	71.42	0	736455	43	31548	40.6	92.27	921	d	Archaea;p Nanohaloarchaeota;c Nanosalinia;o Nanosalinales;f Nanosalinaceae;g B1-Br10-U2g21;s
Gb0054153-1MAG017	No	93.45	2.53	3270146	275	21918	60.8	88.59	3413	d	Archaea;p Halobacteriota;c Halobacteria;o Halobacteriales;f Natrialbaceae;g Tc-Br11;s Tc-Br11 sp003552325
Gb0054153-1MAG018	No	87.09	3.27	2595266	158	34315	64.5	88.23	2708	d	Archaea;p Halobacteriota;c Halobacteria;o Halobacteriales;f Natrialbaceae;g Tc-Br11-E2g1;s Tc-Br11-E2g1 sp001564255

Gb0054153-1MAG01	No	83.82	1.52	1778491	116	27367	62.2	89.19	1949	d	Archaea;p Halobacteriota;c Halobacteria;o Halobacteriales;f Haloferacaceae;g PL-Br10-E2g29;s PL-Br10-E2g29 sp001563965
Gb0054153-1MAG02	No	78.24	6.12	2053335	253	11444	58.7	89.49	2372	d	Archaea;p Halobacteriota;c Halobacteria;o Halobacteriales;f Haloferacaceae;g Halohasta;s
Gb0054153-2MAG00	No	97.16	2.16	3535089	340	19176	67	90.06	3428	d	Bacteria;p Proteobacteria;c Gammaproteobacteria;o Pseudomonadales;f Halomonadaceae;g Halomonas;s Halomonas sp003552795
Gb0054153-2MAG00	Yes	94.85	1.27	2862463	226	23519	68.3	87.86	3003	d	Archaea;p Halobacteriota;c Halobacteria;o Halobacteriales;f Natrialbaceae;g Tc-Br11-E2g8;s Tc-Br11-E2g8 sp003552885
Gb0054153-2MAG00	No	87.47	2.6	3437396	191	30946	62	84.3	3540	d	Archaea;p Halobacteriota;c Halobacteria;o Halobacteriales;f Natrialbaceae;g ;s
Gb0054153-2MAG00	No	94.59	3.55	2671247	199	22291	51.9	90.7	2892	d	Archaea;p Halobacteriota;c Halobacteria;o Halobacteriales;f Haloferacaceae;g Halalkalirubrum;s Halalkalirubrum sp003551725
Gb0054153-2MAG00	No	88.67	1.33	1339774	62	42361	42.4	91.91	1377	d	Bacteria;p Firmicutes;c Bacilli;o Izemoplasmatales;f Izemoplasmataceae;g B1SED10-225;s B1SED10-225 sp003551405
Gb0054153-2MAG00	No	93.73	1.94	2752503	114	49923	65.5	88.73	2796	d	Archaea;p Halobacteriota;c Halobacteria;o Halobacteriales;f Haloferacaceae;g Halorubrum;s Halorubrum sp003554605
Gb0054153-2MAG00	Yes	72.35	0	770362	41	37165	40.5	92.35	959	d	Archaea;p Nanohaloarchaeota;c Nanosalinia;o Nanosalinales;f Nanosalinaceae;g B1-Br10-U2g21;s
Gb0054153-2MAG01	Yes	92.25	3.2	2662871	139	33871	64.7	88.46	2724	d	Archaea;p Halobacteriota;c Halobacteria;o Halobacteriales;f Natrialbaceae;g Tc-Br11-E2g1;s Tc-Br11-E2g1 sp001564255
Gb0054153-2MAG01	No	77.82	1.59	2742057	1027	3229	68.3	90.51	3456	d	Bacteria;p Proteobacteria;c Alphaproteobacteria;o Rhodobacterales;f Rhodobacteraceae;g Rhodobaculum;s Rhodobaculum sp003550665
Gb0054153-2MAG01	Yes	79.13	7.79	1578742	194	15527	38.9	91.88	1801	d	Archaea;p Nanohaloarchaeota;c Nanosalinia;o Nanosalinales;f Nanosalinaceae;g B1-Br10-U2g21;s B1-Br10-U2g21 sp001563915
Gb0054153-2MAG01	Yes	82.09	2.34	1161825	149	51159	40.5	86.25	1386	d	Archaea;p Nanohaloarchaeota;c Nanosalinia;o Nanosalinales;f Nanosalinaceae;g B1-Br10-U2g21;s
Gb0054153-2MAG01	Yes	97.42	7.59	4259448	440	15794	53.4	89.56	4784	d	Archaea;p Halobacteriota;c Halobacteria;o Halobacteriales;f Haloarculaceae;g Halovenus;s Halovenus sp003551945
Gb0054153-2MAG01	No	91.6	9.67	3326334	152	42079	58.1	88.4	3618	d	Archaea;p Halobacteriota;c Halobacteria;o Halobacteriales;f Natrialbaceae;g ;s
Gb0054153-2MAG01	No	77.02	0	774150	37	35570	40.1	92.61	967	d	Archaea;p Nanohaloarchaeota;c Nanosalinia;o Nanosalinales;f Nanosalinaceae;g B1-Br10-U2g21;s B1-Br10-U2g21 sp001564145
Gb0054153-2MAG01	No	84.69	6.95	2786662	190	24757	60.3	88.55	2951	d	Archaea;p Halobacteriota;c Halobacteria;o Halobacteriales;f Natrialbaceae;g Natrarchaeobaculum;s Natrarchaeobaculum sp001563885
Gb0054153-2MAG02	No	84.73	8.3	2277902	125	39294	57.3	88.82	2479	d	Archaea;p Halobacteriota;c Halobacteria;o Halobacteriales;f Haloferacaceae;g Halohasta;s Halohasta sp001563795
Gb0054153-2MAG02	No	90.73	4.67	3098194	241	21785	60.8	88.39	3192	d	Archaea;p Halobacteriota;c Halobacteria;o Halobacteriales;f Natrialbaceae;g Tc-Br11;s Tc-Br11 sp003552325
Gb0054153-2MAG02	No	74.74	1.98	2146340	334	10952	58.5	88.48	2487	d	Archaea;p Halobacteriota;c Halobacteria;o Halobacteriales;f Haloferacaceae;g Halohasta;s
Gb0054153-2MAG02	Yes	85.42	6.48	3193075	614	7106	59.1	88.3	3745	d	Archaea;p Halobacteriota;c Halobacteria;o Halobacteriales;f Natrialbaceae;g ;s
Gb0054154MAG001	No	96.97	1.52	2845157	122	43333	51.8	90.36	3022	d	Archaea;p Halobacteriota;c Halobacteria;o Halobacteriales;f Haloferacaceae;g Halalkalirubrum;s Halalkalirubrum sp003551725
Gb0054154MAG002	Yes	89.36	0.57	2568027	152	36660	33.3	88.45	2342	d	Bacteria;p Bacteroidota;c Bacteroidia;o Flavobacteriales;f Flavobacteriaceae;g Psychroflexus;s
Gb0054154MAG003	Yes	96.45	4.28	3101208	203	26558	62.4	92.53	2967	d	Bacteria;p Proteobacteria;c Gammaproteobacteria;o Nitrococcales;f Aquisalimonadaceae;g ;s
Gb0054154MAG004	Yes	96.36	4.03	2624790	216	21001	66.4	89.49	2594	d	Bacteria;p Proteobacteria;c Gammaproteobacteria;o Ectothiorhodospirales;f Thioalkalivibrionaceae;g Thioalkalivibrio;s Thioalkalivibrio versutus
Gb0054154MAG005	Yes	94.62	0.54	1440772	84	77907	42.7	88.03	1408	d	Bacteria;p Proteobacteria;c Alphaproteobacteria;o Caedimonadales;f ;g ;s
Gb0054154MAG006	Yes	98.67	1.33	1497361	84	52505	43.8	93.4	1522	d	Bacteria;p Firmicutes;c Bacilli;o Izemoplasmatales;f Izemoplasmataceae;g CSBR16-87;s
Gb0054154MAG007	Yes	96.72	3.39	4784593	379	22515	61	88.92	4584	d	Bacteria;p Proteobacteria;c Alphaproteobacteria;o DSM-16000;f Inquilinaceae;g ;s
Gb0054154MAG008	No	90.03	6.86	2305150	386	13136	55.8	94.23	2561	d	Bacteria;p Proteobacteria;c Gammaproteobacteria;o Nitrococcales;f Nitrococcaceae;g Spiribacter;s
Gb0054154MAG009	No	98.37	5.91	4697865	492	35998	59.4	89.9	5194	d	Bacteria;p Proteobacteria;c Alphaproteobacteria;o Rhodobacterales;f Rhodobacteraceae;g Roseinatronobacter;s Roseinatronobacter monicus
Gb0054154MAG010	No	94.8	6.13	3637362	234	33628	66.8	91.03	3683	d	Bacteria;p Proteobacteria;c Alphaproteobacteria;o Rhodobacterales;f Rhodobacteraceae;g Rhodobaculum;s
Gb0054154MAG011	Yes	90.26	4.19	3657805	614	7762	57.4	88.64	3500	d	Bacteria;p Verrucomicrobiota;c Verrucomicrobiae;o Opitutales;f T3Sed10-336;g ;s
Gb0054154MAG012	Yes	94.99	2.73	3624197	394	15020	41.1	88.88	3428	d	Bacteria;p Bacteroidota;c Rhodothermia;o Balneolales;f Balneolaceae;g ;s
Gb0054154MAG013	Yes	73.19	0.55	1892791	514	3902	50.1	91.03	2096	d	Bacteria;p Bacteroidota;c Rhodothermia;o Balneolales;f Balneolaceae;g ;s
Gb0054154MAG014	No	79.78	1.12	778558	100	10658	38.7	89.19	821	d	Bacteria;p Dependentiae;c Babeliae;o Babeliales;f Babeliaceae;g ;s
Gb0054154MAG015	No	97.44	8.15	3872489	196	41585	70.9	88.11	3686	d	Bacteria;p Actinobacteriota;c Actinomycetia;o Nitriliruptorales;f Nitriliruptoraceae;g T1Sed10-7;s
Gb0054154MAG017	No	97.36	4.27	3346507	155	29779	71.4	90.51	3147	d	Bacteria;p Actinobacteriota;c Actinomycetia;o Nitriliruptorales;f Nitriliruptoraceae;g PWLR01;s
Gb0054154MAG018	No	80.59	7.83	1651896	287	9315	59.1	95.81	1775	d	Bacteria;p Proteobacteria;c Gammaproteobacteria;o Xanthomonadales;f Wenzhouxiangellaceae;g Wenzhouxiangella;s
Gb0054154MAG019	No	87.83	2.11	3926280	342	21087	65.2	88.87	3802	d	Bacteria;p Proteobacteria;c Alphaproteobacteria;o Rhizobiales;f Beijerinckiaceae;g Salinarimonas;s Salinarimonas sp003567055
Gb0054154MAG020	Yes	74.89	1.1	2212633	676	3584	70.2	92.12	2376	d	Bacteria;p Gemmatimonadota;c Gemmatimonadetes;o Longimicrobiales;f UBA6960;g REBX01;s
Gb0054154MAG022	Yes	96.09	5.87	3697334	242	26913	67.7	89.23	3582	d	Bacteria;p Proteobacteria;c Alphaproteobacteria;o Rhodobacterales;f Rhodobacteraceae;g PUOA01;s PUOA01 sp007121115
Gb0054154MAG023	No	84.8	7.09	3505232	865	5145	55.1	92.36	3531	d	Bacteria;p Verrucomicrobiota;c Verrucomicrobiae;o Opitutales;f Verruco-01;g ;s
Gb0054154MAG024	Yes	84.74	2.65	3177515	384	9945	65.4	91.63	3015	d	Bacteria;p Proteobacteria;c Gammaproteobacteria;o Pseudomonadales;f HTCC2089;g ;s
Gb0054154MAG026	Yes	96.33	3.32	3103795	245	42283	58.5	91.15	3133	d	Bacteria;p Proteobacteria;c Gammaproteobacteria;o Pseudomonadales;f Natronospirillaceae;g Natronospirillum;s
Gb0054154MAG030	No	94.4	3.38	3892745	532	17347	62.9	89.11	4008	d	Bacteria;p Proteobacteria;c Gammaproteobacteria;o Nitrococcales;f Aquisalimonadaceae;g SKGQ01;s SKGQ01 sp007117515
Gb0054154MAG037	Yes	89.66	8.34	4544420	627	11307	65.4	88.7	4779	d	Bacteria;p Proteobacteria;c Alphaproteobacteria;o Rhodobacterales;f Rhodobacteraceae;g Roseinatronobacter;s Roseinatronobacter sp003564655
Gb0054154MAG042	Yes	82.27	9.27	3677360	408	11496	64.5	87.76	3696	d	Bacteria;p Proteobacteria;c Alphaproteobacteria;o Rhizobiales;f Beijerinckiaceae;g Salinarimonas;s
Gb0054154MAG046	Yes	88.53	0.8	2750114	141	28911	69.9	91.5	2639	d	Bacteria;p Proteobacteria;c Alphaproteobacteria;o Rhodobacterales;f Rhodobacteraceae;g PUOA01;s PUOA01 sp003551925
Gb0054155MAG001	No	94.12	3.93	2876193	216	22536	51.7	90.67	3078	d	Archaea;p Halobacteriota;c Halobacteria;o Halobacteriales;f Haloferacaceae;g Halalkalirubrum;s Halalkalirubrum sp003551725
Gb0054155MAG002	No	96.89	0.56	3757675	141	42280	62.3	87.82	3275	d	Bacteria;p Bacteroidota;c Rhodothermia;o Rhodothermales;f Salinibacteraceae;g Tc-Br11-B2g6-7;s Tc-Br11-B2g6-7 sp001564055
Gb0054155MAG003	No	86.39	2.55	2262050	114	39487	58.4	89.21	2428	d	Archaea;p Halobacteriota;c Halobacteria;o Halobacteriales;f Haloferacaceae;g PL-Br10-E2g29;s
Gb0054155MAG004	No	94.43	2.42	3232775	109	42578	62.3	84.39	3266	d	Archaea;p Halobacteriota;c Halobacteria;o Halobacteriales;f Natrialbaceae;g Natronococcus;s
Gb0054155MAG005	Yes	86.43	4.19	5420553	279	33132	60.3	86.29	4244	d	Bacteria;p Myxococcota;c Bradymonadia;o Bradymonadales;f Bradymonadaceae;g SLJM01;s
Gb0054155MAG006	No	89.71	3.6	1900280	128	24861	62	88.81	2059	d	Archaea;p Halobacteriota;c Halobacteria;o Halobacteriales;f Haloferacaceae;g PL-Br10-E2g29;s PL-Br10-E2g29 sp001563965
Gb0054155MAG007	No	95.11	1.32	2554140	144	30042	39.7	90.45	2453	d	Bacteria;p Firmicutes F;c Halanaerobiia;o Halanaerobiales;f Halarsenatibacteraceae;g T1SED10-84;s T1SED10-84 sp003553485
Gb0054155MAG008	Yes	91.99	0	2595155	147	27554	59.9	91.12	2708	d	Archaea;p Halobacteriota;c Halobacteria;o Halobacteriales;f Haloarculaceae;g Halovenus;s Halovenus sp003551265
Gb0054155MAG009	No	86.05	4.03	2744526	426	8724	68.4	87.49	3089	d	Archaea;p Halobacteriota;c Halobacteria;o Halobacteriales;f Natrialbaceae;g Tc-Br11-E2g8;s Tc-Br11-E2g8 sp001564115
Gb0054155MAG010	No	94.5	2.55	2604190	441	7564	55.1	90.13	2994	d	Archaea;p Halobacteriota;c Halobacteria;o Halobacteriales;f Haloarculaceae;g Halovenus;s Halovenus sp001564135
Gb0054155MAG011	No	86.07	4.19	2313360	307	9732	43.6	89.8	2288	d	Bacteria;p Bacteroidota;c Rhodothermia;o Balneolales;f Balneolaceae;g SW132;s SW132 sp007129085
Gb0054155MAG012	Yes	94.45	5.37	2963917	197	21920	63.8	88.32	3174	d	Archaea;p Halobacteriota;c Halobacteria;o Halobacteriales;f Haloarculaceae;g Natronomonas;s
Gb0054155MAG013	No	75.62	0	678497	59	18296	41	90.82	832	d	Archaea;p Nanohaloarchaeota;c Nanosalinia;o Nanosalinales;f Nanosalinaceae;g B1-Br10-U2g19;s B1-Br10-U2g19 sp001563905
Gb0054155MAG015	Yes	92.8	6.54	2973682	211	27853	62.4	89.51	3313	d	Archaea;p Halobacteriota;c Halobacteria;o Halobacteriales;f Natronoarchaeaceae;g Natronoarchaeum;s
Gb0054155MAG016	No	92.04	8.45	1837668	222	11010	64.4	94.99	1956	d	Bacteria;p Proteobacteria;c Gammaproteobacteria;o Nitrococcales;f Nitrococcaceae;g Spiribacter;s Spiribacter sp009676705
Gb0054155MAG017	No	92.2	2.63	3043580	142	33137	64	84.95	3021	d	Archaea;p Halobacteriota;c Halobacteria;o Halobacteriales;f Natrialbaceae;g Tc-Br11;s Tc-Br11 sp001564275
Gb0054155MAG018	No	89.81	2.98	2615209	282	13717	64.8	87.92	2920	d	Archaea;p Halobacteriota;c Halobacteria;o Halobacteriales;f Haloarculaceae;g Natronomonas;s
Gb0054155MAG019	No	76.48	0	772879	20	56196	38.7	89.78	898	d	Archaea;p Nanohaloarchaeota;c Nanosalinia;o Nanosalinales;f Nanosalinaceae;g B1-Br10-U2g21;s
Gb0054155MAG020	Yes	91.56	3.34	2383076	250	14066	61.6	89.77	2649	d	Archaea;p Halobacteriota;c Halobacteria;o Halobacteriales;f Haloarculaceae;g Natronomonas;s
Gb0054155MAG021	No	86.05	8.03	2601653	150	23845	66.1	88.96	2658	d	Archaea;p Halobacteriota;c Halobacteria;o Halobacteriales;f Haloferacaceae;g Halorubrum;s Halorubrum sp003554605
Gb0054155MAG022	No	77.1	6.28	3222806	818	4429	44.3	86.72	3396	d	Bacteria;p Bacteroidota;c Rhodothermia;o Balneolales;f Natronogracilivirgulaceae;g ;s
Gb0054155MAG023	Yes	75.09	4	2701060	327	12732	66.8	87.03	2858	d	Archaea;p Halobacteriota;c Halobacteria;o Halobacteriales;f Haloferacaceae;g Halorubrum;s
Gb0054155MAG024	No	79.26	4.68	2554504	352	10612	63.6	88.32	2837	d	Archaea;p Halobacteriota;c Halobacteria;o Halobacteriales;f Haloferacaceae;g Halorubrum;s
Gb0054155MAG025	No	72.59	3.74	739130	50	25888	40	92.57	931	d	Archaea;p Nanohaloarchaeota;c Nanosalinia;o Nanosalinales;f Nanosalinaceae;g B1-Br10-U2g21;s B1-Br10-U2g21 sp001564145
Gb0054155MAG029	No	81.01	5.17	2919082	556	6414	60.9	87.95	3348	d	Archaea;p Halobacteriota;c Halobacteria;o Halobacteriales;f Natrialbaceae;g Tc-Br11;s Tc-Br11 sp003552325
Gb0054155MAG033	Yes	72.95	5.84	2599659	281	13871	62.2	88.16	2799	d	Archaea;p Halobacteriota;c Halobacteria;o Halobacteriales;f Haloferacaceae;g Halorubrum;s
Gb0054156-1MAG00	No	93.97	2.73	4422865	327	39247	44.1	91.28	4069	d	Bacteria;p Bacteroidota;c T3Sed10-11;o T3Sed10-11;f T3Sed10-11;g T3Sed10-11;s T3Sed10-11 sp003568415
Gb0054156-1MAG00	No	97.31	2.15	3660517	181	37967	45.8	89.8	2928	d	Bacteria;p Bacteroidota;c Bacteroidia;o Bacteroidales;f UBA7960;g PUML01;s PUML01 sp007129255
Gb0054156-1MAG00	No	93.83	1.55	2544298	85	64723	44.4	90.03	2502	d	Bacteria;p Firmicutes F;c Halanaerobiia;o B1SED10-174;f B1SED10-174;g T1SED10-26;s T1SED10-26 sp007122335
Gb0054156-1MAG00	No	92.2	0	2303283	75	44014	50.2	89.92	1891	d	Bacteria;p Bacteroidota;c Bacteroidia;o Bacteroidales;f UBA7960;g PUPG01;s PUPG01 sp007125585
Gb0054156-1MAG00	Yes	92.16	3.74	2536197	193	33557	44.6	86.69	2467	d	Bacteria;p Firmicutes D;c Dethiobacteria;o DTU022;f DTU022;g B1SED10-74M;s B1SED10-74M sp007128625
Gb0054156-1MAG00	Yes	94.52	2.84	4153716	569	9933	45.2	91.26	4002	d	Bacteria;p Bacteroidota;c T3Sed10-11;o T3Sed10-11;f T3Sed10-11;g T3Sed10-11;s T3Sed10-11 sp007131635
Gb0054156-1MAG00	No	88.35	1.2	2191598	133	30943	50.7	88.03	2079	d	Bacteria;p Firmicutes D;c Dethiobacteria;o DTU022;f DTU022;g B1SED10-74M;s B1SED10-74M sp003554395
Gb0054156-1MAG00	No	94.62	2.42	2534671	180	27302	49.6	91.89	2162	d	Bacteria;p Bacteroidota;c Bacteroidia;o Bacteroidales;f UBA7960;g PYGY01;s PYGY01 sp007129945
Gb0054156-1MAG00	No	81.12	1.4	1688633	47	86550	41.6	90.71	1694	d	Bacteria;p Firmicutes A;c Clostridia;o Peptostreptococcales;f T1SED10-28;g T1SED10-28;s T1SED10-28 sp003554105
Gb0054156-1MAG01	No	95.97	2.25	3188771	287	18336	54.9	85.59	3031	d	Bacteria;p Desulfobacterota;c Syntrophobacteria;o Syntrophobacterales;f Syntrophobacteraceae;g SLCH01;s SLCH01 sp007125915
Gb0054156-1MAG01	No	93.28	5.4	1896717	311	8427	44.3	87.05	2080	d	Bacteria;p Firmicutes D;c Dethiobacteria;o SKNC01;f SKNC01;g SKNC01;s SKNC01 sp003560915
Gb0054156-1MAG01	Yes	81.17	1.72	553896	11	72263	37.4	95.01	588	d	Bacteria;p Patescibacteria;c Paceibacteria;o Paceibacterales;f PWPS01;g ;s
Gb0054156-1MAG01	Yes	88.52	2.46	3548325	347	15266	42.9	86.46	3305	d	Bacteria;p Bacteroidota;c Rhodothermia;o Balneolales;f Balneolaceae;g SLAU01;s SLAU01 sp007126675
Gb0054156-1MAG01	Yes	92.53	5.4	3622205	266	25631	66.5	90.48	3492	d	Bacteria;p Proteobacteria;c Gammaproteobacteria;o XJ16;f Halofilaceae;g SLKC01;s
Gb0054156-1MAG01	Yes	98.9	3.3	4251212	249	29417	67.3	91.13	3580	d	Bacteria;p Gemmatimonadota;c Gemmatimonadetes;o Longimicrobiales;f UBA6960;g PWLA01;s PWLA01 sp003567615
Gb0054156-1MAG01	No	97.54	6.28	3862999	266	24284	45	87.07	3429	d	Bacteria;p Bacteroidota;c Rhodothermia;o Balneolales;f Balneolaceae;g QGGB01;s QGGB01 sp007118525

Gb0054156-1MAG01	Yes	93.55	9.59	3963637	469	12979	46.1	88.86	3451	d	Bacteria;p	Bacteroidota;c Bacteroidia;o Bacteroidales;f UBA7960;g PUPG01;s PUPG01 sp007122505
Gb0054156-1MAG01	Yes	79.29	7.45	2683758	601	5180	47.1	87.35	2791	d	Bacteria;p	Chloroflexota;c Anaerolineae;o Anaerolineales;f Anaerolineaceae;g Brevefilum;s
Gb0054156-1MAG01	Yes	89.09	5.64	3090075	271	20872	47	88.13	2855	d	Bacteria;p	Chloroflexota;c Anaerolineae;o Anaerolineales;f Anaerolineaceae;g Brevefilum;s
Gb0054156-1MAG02	Yes	96.49	1.71	4189092	288	25569	70.7	87.27	4147	d	Bacteria;p	Actinobacteriota;c Actinomycetia;o Nitriliruptorales;f Nitriliruptoraceae;g CSSed11-175R1;s
Gb0054156-1MAG02	No	98.06	7.49	4231993	250	33442	44.6	85.35	4027	d	Bacteria;p	Desulfobacterota;c Desulfobacteria;o Desulfobacterales;f SKZT01;g SKZT01;s SKZT01 sp007127235
Gb0054156-1MAG02	No	79.01	3.49	2442984	488	5896	31.6	89.75	2392	d	Bacteria;p	Bacteroidota;c Bacteroidia;o Bacteroidales;f UBA7960;g PWLF01;s PWLF01 sp003559475
Gb0054156-1MAG02	No	88.52	5.79	4712787	665	17240	42.6	88.78	4281	d	Bacteria;p	Bacteroidota;c Rhodothermia;o Balneolales;f Balneolaceae;g SKRC01;s SKRC01 sp007134665
Gb0054156-1MAG02	No	72.78	4.19	1882110	242	11578	42.9	90.34	1787	d	Bacteria;p	Bacteroidota;c Bacteroidia;o Bacteroidales;f PUMT01;g PUMT01;s PUMT01 sp007125955
Gb0054156-1MAG02	Yes	89.62	8.98	2975453	499	10430	46.1	87.73	3113	d	Bacteria;p	Firmicutes D;c Dethiobacteria;o DTU022;f DTU022;g T3SED10-7;s T3SED10-7 sp003564505
Gb0054156-1MAG02	No	81.17	3.76	578864	67	11974	35.4	91.4	648	d	Bacteria;p	Patescibacteria;c Paceibacteria;o Paceibacterales;f PWPS01;g PWHW01;s PWHW01 sp003561025
Gb0054156-1MAG02	No	95.28	4.5	3186696	119	45452	64.9	90.92	2701	d	Bacteria;p	Gemmatimonadota;c Gemmatimonadetes;o Longimicrobiales;f UBA6960;g PWLA01;s PWLA01 sp003562075
Gb0054156-1MAG02	No	91.58	4.1	2817423	337	12345	44.1	88.77	2706	d	Bacteria;p	Bacteroidota;c Rhodothermia;o Balneolales;f Balneolaceae;g SW132;s SW132 sp007129085
Gb0054156-1MAG03	Yes	77.53	1.12	673687	66	20927	29.6	92.03	716	d	Bacteria;p	Patescibacteria;c ABY1;o BM507;f UBA12465;g PWHG01;s
Gb0054156-1MAG03	No	90.79	3.83	2492897	261	14891	66.9	92.41	2488	d	Bacteria;p	Proteobacteria;c Gammaproteobacteria;o Nitrococcales;f Halorhodospiraceae;g Alkalilimnicola;s Alkalilimnicola mobilis
Gb0054156-1MAG03	No	89.98	3.5	3281395	698	6226	69.6	90.02	3556	d	Bacteria;p	Proteobacteria;c Alphaproteobacteria;o Rhodobacterales;f Rhodobacteraceae;g Pararhodobacter;s Pararhodobacter sp007131945
Gb0054156-1MAG03	Yes	83.96	1.12	3325367	485	8463	43	87.42	2892	d	Bacteria;p	Bacteroidota;c Bacteroidia;o Bacteroidales;f UBA7960;g SKVR01;s SKVR01 sp007131245
Gb0054156-1MAG03	No	87.7	6.76	3822319	301	23145	47.4	86.46	3340	d	Bacteria;p	Bacteroidota;c Rhodothermia;o Balneolales;f Balneolaceae;g QGGB01;s QGGB01 sp003561515
Gb0054156-1MAG04	No	96.8	3.27	1566732	174	14241	46.9	92.31	1666	d	Bacteria;p	Firmicutes;c Bacilli;o Izemoplasmatales;f Izemoplasmataceae;g B1SED10-225;s B1SED10-225 sp007135105
Gb0054156-1MAG04	No	88.82	5.61	3344561	419	12558	45.8	86.77	2944	d	Bacteria;p	Bacteroidota;c Bacteroidia;o Chitinophagales;f Saprospiraceae;g PWJY01;s PWJY01 sp007121335
Gb0054156-1MAG04	No	82.64	3.15	2191395	398	6384	54	91.49	2104	d	Bacteria;p	Bacteroidota;c Bacteroidia;o Bacteroidales;f UBA7960;g PWJC01;s PWJC01 sp003560165
Gb0054156-1MAG04	No	84.57	6.71	2666068	528	6336	45.4	88.26	2892	d	Bacteria;p	Firmicutes D;c Dethiobacteria;o DTU022;f DTU022;g T3SED10-7;s T3SED10-7 sp007127255
Gb0054156-1MAG05	No	85.84	6.99	2672462	202	21225	45.4	90.73	2233	d	Bacteria;p	Bacteroidota;c Bacteroidia;o Bacteroidales;f UBA7960;g SKTA01;s SKTA01 sp007132825
Gb0054156-1MAG05	No	82.56	1.11	1413794	429	3976	38.9	92.45	1731	d	Bacteria;p	Firmicutes;c Bacilli;o Izemoplasmatales;f Izemoplasmataceae;g B1SED10-225;s B1SED10-225 sp003559585
Gb0054156-1MAG07	No	81.85	3.23	2484930	310	12313	47.7	90.69	2148	d	Bacteria;p	Bacteroidota;c Bacteroidia;o Bacteroidales;f UBA7960;g PUPG01;s PUPG01 sp007124575
Gb0054156-2MAG00	No	97.33	1.33	1615251	52	88733	46.9	92.21	1583	d	Bacteria;p	Firmicutes;c Bacilli;o Izemoplasmatales;f Izemoplasmataceae;g B1SED10-225;s B1SED10-225 sp007135105
Gb0054156-2MAG00	No	93.83	1.55	2580523	122	72763	44.4	89.82	2550	d	Bacteria;p	Firmicutes F;c Halanaerobiia;o B1SED10-174;f B1SED10-174;g T1SED10-26;s T1SED10-26 sp007122335
Gb0054156-2MAG00	No	85.24	4.76	2389724	117	53269	43.1	90.3	2058	d	Bacteria;p	Bacteroidota;c Bacteroidia;o Bacteroidales;f PUMT01;g PUMT01;s PUMT01 sp007125955
Gb0054156-2MAG00	Yes	96.7	3.3	4082913	205	42376	66.9	90.59	3481	d	Bacteria;p	Gemmatimonadota;c Gemmatimonadetes;o Longimicrobiales;f UBA6960;g PWLA01;s PWLA01 sp007121555
Gb0054156-2MAG00	No	89.19	2.61	2131377	156	27010	44.8	87.73	2055	d	Bacteria;p	Firmicutes D;c Dethiobacteria;o DTU022;f DTU022;g B1SED10-74M;s B1SED10-74M sp007128625
Gb0054156-2MAG00	No	81.12	0.7	1596025	53	79819	41.5	91.19	1587	d	Bacteria;p	Firmicutes A;c Clostridia;o Peptostreptococcales;f T1SED10-28;g T1SED10-28;s T1SED10-28 sp003554105
Gb0054156-2MAG00	Yes	98.55	1.45	3245602	125	56462	55	85.58	2949	d	Bacteria;p	Desulfobacterota;c Syntrophobacteria;o Syntrophobacterales;f Syntrophobacteraceae;g SLCH01;s SLCH01 sp007125915
Gb0054156-2MAG00	Yes	97.85	2.69	3433571	150	44638	45.9	89.76	2785	d	Bacteria;p	Bacteroidota;c Bacteroidia;o Bacteroidales;f UBA7960;g PUML01;s PUML01 sp007129255
Gb0054156-2MAG00	Yes	94.7	3.67	2174302	91	39835	44.2	86.88	2145	d	Bacteria;p	Firmicutes D;c Dethiobacteria;o SKNC01;f SKNC01;g SKNC01;s SKNC01 sp003560915
Gb0054156-2MAG01	Yes	92.47	9.34	2920394	285	24986	31.5	87.85	2748	d	Bacteria;p	Bacteroidota;c Bacteroidia;o Bacteroidales;f UBA7960;g PWLF01;s PWLF01 sp003559475
Gb0054156-2MAG01	Yes	94.81	0	1599737	111	31925	39.1	92.52	1625	d	Bacteria;p	Firmicutes;c Bacilli;o Izemoplasmatales;f Izemoplasmataceae;g B1SED10-225;s B1SED10-225 sp003559585
Gb0054156-2MAG01	No	95.97	1.08	2524921	81	51467	54.1	90.97	2072	d	Bacteria;p	Bacteroidota;c Bacteroidia;o Bacteroidales;f UBA7960;g PWJC01;s PWJC01 sp003560165
Gb0054156-2MAG01	No	94.62	1.61	2487824	89	47561	50.1	90.03	2037	d	Bacteria;p	Bacteroidota;c Bacteroidia;o Bacteroidales;f UBA7960;g PUPG01;s PUPG01 sp007125585
Gb0054156-2MAG01	No	98.9	6.04	4121502	226	35325	67.4	91.55	3459	d	Bacteria;p	Gemmatimonadota;c Gemmatimonadetes;o Longimicrobiales;f UBA6960;g PWLA01;s PWLA01 sp003567615
Gb0054156-2MAG01	No	80.93	2.9	2003115	116	33813	50.5	88.25	1923	d	Bacteria;p	Firmicutes D;c Dethiobacteria;o DTU022;f DTU022;g B1SED10-74M;s B1SED10-74M sp003554395
Gb0054156-2MAG01	No	96.99	6.01	4050633	463	17911	45.1	85.93	3842	d	Bacteria;p	Bacteroidota;c Rhodothermia;o Balneolales;f Balneolaceae;g QGGB01;s QGGB01 sp007118525
Gb0054156-2MAG01	Yes	91.09	7.09	2309546	814	3617	65	90.87	2732	d	Bacteria;p	PUNC01;c PUNC01;o PUNC01;f PWXJ01;g PWXJ01;s PWXJ01 sp003561965
Gb0054156-2MAG01	No	85.81	2.47	3292830	151	40938	44.8	85.72	3125	d	Bacteria;p	Desulfobacterota;c Desulfobacteria;o Desulfobacterales;f SKZT01;g SKZT01;s SKZT01 sp007127235
Gb0054156-2MAG01	No	92.88	6.99	4256592	279	37616	44	91.2	3930	d	Bacteria;p	Bacteroidota;c T3Sed10-11;o T3Sed10-11;f T3Sed10-11;g T3Sed10-11;s T3Sed10-11 sp003568415
Gb0054156-2MAG02	Yes	96.19	9.46	3574045	282	23078	65.6	91.63	3382	d	Bacteria;p	Proteobacteria;c Gammaproteobacteria;o Xanthomonadales;f Wenzhouxiangellaceae;g Wenzhouxiangella;s Wenzhouxiangella sp007121485
Gb0054156-2MAG02	No	77.72	1.72	567580	9	90756	37.4	94.24	605	d	Bacteria;p	Patescibacteria;c Paceibacteria;o Paceibacterales;f PWPS01;g ;s
Gb0054156-2MAG02	No	93.78	3.94	3967788	283	23192	70.7	87.74	3927	d	Bacteria;p	Actinobacteriota;c Actinomycetia;o Nitriliruptorales;f Nitriliruptoraceae;g CSSed11-175R1;s
Gb0054156-2MAG02	No	83.88	2.46	3166457	308	16090	42.8	86.84	2931	d	Bacteria;p	Bacteroidota;c Rhodothermia;o Balneolales;f Balneolaceae;g SLAU01;s SLAU01 sp007126675
Gb0054156-2MAG02	Yes	91.39	5.13	2607729	223	17239	58.4	89.94	2285	d	Bacteria;p	Verrucomicrobiota;c Kiritimatiellae;o SLAD01;f SLAD01;g SLKG01;s SLKG01 sp007134735
Gb0054156-2MAG02	No	82.89	0	579812	39	31990	35.6	90.85	612	d	Bacteria;p	Patescibacteria;c Paceibacteria;o Paceibacterales;f PWPS01;g PWHW01;s PWHW01 sp003561025
Gb0054156-2MAG02	No	92.33	3.1	4048908	304	22305	43.7	89.21	3354	d	Bacteria;p	Bacteroidota;c Bacteroidia;o Bacteroidales;f PUMT01;g PXAP01;s PXAP01 sp007120695
Gb0054156-2MAG02	No	77.53	2.25	733786	71	20866	29.7	91.51	789	d	Bacteria;p	Patescibacteria;c ABY1;o BM507;f UBA12465;g PWHG01;s
Gb0054156-2MAG02	No	89.6	3.01	4269333	471	13613	45.4	91.4	3892	d	Bacteria;p	Bacteroidota;c T3Sed10-11;o T3Sed10-11;f T3Sed10-11;g T3Sed10-11;s T3Sed10-11 sp007131635
Gb0054156-2MAG02	No	90.44	6.01	2858360	330	13854	44.1	89.04	2687	d	Bacteria;p	Bacteroidota;c Rhodothermia;o Balneolales;f Balneolaceae;g SW132;s SW132 sp007129085
Gb0054156-2MAG03	No	97.8	7.69	4546981	402	24957	67	89.98	3953	d	Bacteria;p	Gemmatimonadota;c Gemmatimonadetes;o Longimicrobiales;f UBA6960;g PWLA01;s PWLA01 sp007121775
Gb0054156-2MAG03	No	90.44	0.5	1927999	109	29504	53.5	87.05	1879	d	Bacteria;p	Firmicutes D;c Dethiobacteria;o DTU022;f UBA8154;g SKMY01;s SKMY01 sp003556225
Gb0054156-2MAG03	No	90.71	6.01	3716070	301	23252	47.3	86.64	3324	d	Bacteria;p	Bacteroidota;c Rhodothermia;o Balneolales;f Balneolaceae;g QGGB01;s QGGB01 sp003561515
Gb0054156-2MAG03	No	84.92	2.91	2355301	561	4842	56.7	89.14	2489	d	Bacteria;p	Planctomycetota;c Phycisphaerae;o Sedimentisphaerales;f Anaerohalosphaeraceae;g T3Sed10-213;s T3Sed10-213 sp007121565
Gb0054156-2MAG03	No	96.73	1.35	3561206	251	23827	61.8	89.38	3158	d	Bacteria;p	Verrucomicrobiota;c Verrucomicrobiae;o Opitutales;f Verruco-01;g SLNJ01;s SLNJ01 sp007133065
Gb0054156-2MAG03	Yes	96.84	1.99	3129838	371	13582	45.9	82.52	3169	d	Bacteria;p	Firmicutes;c Bacilli;o Bacillales H;f Salisediminibacteriaceae;g Alkalicoccus;s Alkalicoccus sp003560495
Gb0054156-2MAG03	Yes	91.94	4.3	2580516	180	33859	49.1	93.2	2239	d	Bacteria;p	Bacteroidota;c Bacteroidia;o Bacteroidales;f UBA7960;g PUPG01;s PUPG01 sp003557385
Gb0054156-2MAG03	No	93.06	2.3	2626264	230	19386	49.5	91.76	2280	d	Bacteria;p	Bacteroidota;c Bacteroidia;o Bacteroidales;f UBA7960;g PYGY01;s PYGY01 sp007129945
Gb0054156-2MAG04	No	90.66	8.89	4153393	893	5855	65.8	89.96	4415	d	Bacteria;p	Gemmatimonadota;c Gemmatimonadetes;o Longimicrobiales;f UBA6960;g SKKN01;s SKKN01 sp007121455
Gb0054156-2MAG04	Yes	90.47	4.99	2591037	216	25563	45.7	88.5	2508	d	Bacteria;p	Firmicutes D;c Dethiobacteria;o DTU022;f DTU022;g T3SED10-7;s T3SED10-7 sp007127255
Gb0054156-2MAG04	Yes	80.46	3.45	612751	35	29331	36.8	87.79	663	d	Bacteria;p	Patescibacteria;c Paceibacteria;o UBA9983 A;f CSBR16-193;g ;s
Gb0054156-2MAG04	Yes	87.64	2.81	2082776	252	12891	51.4	95.15	1859	d	Bacteria;p	CG03;c SLGR01;o SLGR01;f SLGR01;g SLGR01;s SLGR01 sp007136585
Gb0054156-2MAG04	Yes	98.07	3.85	3836677	381	17130	69.6	89.8	3792	d	Bacteria;p	Proteobacteria;c Alphaproteobacteria;o Rhodobacterales;f Rhodobacteraceae;g Pararhodobacter;s Pararhodobacter sp007131945
Gb0054156-2MAG04	No	91.27	4.28	3470479	292	21710	66.5	90.36	3368	d	Bacteria;p	Proteobacteria;c Gammaproteobacteria;o XJ16;f Halofilaceae;g SLKC01;s
Gb0054156-2MAG04	Yes	70.69	6.9	558797	19	42846	38.8	90.52	605	d	Bacteria;p	Patescibacteria;c Paceibacteria;o Paceibacterales;f PWPS01;g PWHW01;s PWHW01 sp003560665
Gb0054156-2MAG04	No	84.23	6.34	1373782	317	4890	58.1	88.45	1617	d	Bacteria;p	Chloroflexota;c Dehalococcoidia;o GIF9;f AB-539-J10;g E29-bin54;s E29-bin54 sp003556385
Gb0054156-2MAG04	Yes	77.73	0.93	670560	71	13082	27.3	90.27	793	d	Archaea;p	Iainarchaeota;c Iainarchaeia;o 0-14-0-20-30-16;f CSSED10-239;g CSSED10-239;s CSSED10-239 sp003559395
Gb0054156-2MAG04	No	73.27	3.27	1245408	209	7873	55.3	93.86	1435	d	Archaea;p	Halobacteriota;c Methanomicrobia;o Methanomicrobiales;f Methanocorpusculaceae;g Methanocalculus;s Methanocalculus sp007130455
Gb0054156-2MAG05	Yes	78.15	3.28	3289402	969	4079	64.4	91.43	3174	d	Bacteria;p	Myxococcota;c UBA9042;o PWKZ01;f PWKZ01;g PWKZ01;s PWKZ01 sp003559575
Gb0054156-2MAG05	Yes	80.84	4.2	2087983	890	2641	49.8	92.33	2742	d	Bacteria;p	Proteobacteria;c Gammaproteobacteria;o Pseudomonadales;f Halomonadaceae;g Marinospirillum;s
Gb0054156-2MAG05	Yes	73.29	0	702698	162	6153	41	91.18	924	d	Archaea;p	B1Sed10-29;c B1Sed10-29;o B1Sed10-29;f B1Sed10-29;g CSSED10-269;s CSSED10-269 sp003558235
Gb0054156-2MAG05	No	72.62	3.79	571381	84	9574	41.3	92.31	636	d	Bacteria;p	Patescibacteria;c Paceibacteria;o Paceibacterales;f PWPS01;g PWPS01;s
Gb0054156-2MAG05	Yes	89.91	5.78	5784743	1000	8079	67.3	88.48	5597	d	Bacteria;p	Gemmatimonadota;c Gemmatimonadetes;o Longimicrobiales;f UBA6960;g PWLA01;s PWLA01 sp003559555
Gb0054156-2MAG05	Yes	88.76	0.85	671141	43	26272	30.9	93.62	706	d	Bacteria;p	Firmicutes;c Bacilli;o RF39;f UBA660;g CSSED10-109M;s CSSED10-109M sp003559495
Gb0054156-2MAG05	No	93.31	7.25	2536518	407	8465	48.8	85.84	2711	d	Bacteria;p	Desulfobacterota I;c Desulfovibrionia;o Desulfovibrionales;f Desulfonatronovibrionaceae;g Desulfonatronovibrio;s
Gb0054156-2MAG05	No	82.36	7.33	2702626	487	9327	46.1	88.11	2847	d	Bacteria;p	Firmicutes D;c Dethiobacteria;o DTU022;f DTU022;g T3SED10-7;s T3SED10-7 sp003564505
Gb0054156-2MAG06	No	88.92	7.59	3166182	401	15235	65.3	87.06	3318	d	Bacteria;p	Proteobacteria;c Gammaproteobacteria;o Ectothiorhodospirales;f Thioalkalivibrionaceae;g Thioalkalivibrio B;s Thioalkalivibrio B sp007116035
Gb0054156-2MAG06	No	83.71	8.28	2466085	240	29306	66.7	88.77	2423	d	Bacteria;p	Proteobacteria;c Gammaproteobacteria;o Ectothiorhodospirales;f Thioalkalivibrionaceae;g Thioalkalivibrio B;s Thioalkalivibrio B sp003563455
Gb0054156-2MAG06	No	86.88	3.03	2499314	257	14382	44.9	90.83	2144	d	Bacteria;p	Bacteroidota;c Bacteroidia;o Bacteroidales;f UBA7960;g SKTA01;s SKTA01 sp007132825
Gb0054156-2MAG06	Yes	87.88	4.04	3443619	880	4723	65.4	85.79	4233	d	Archaea;p	Halobacteriota;c Halobacteria;o Halobacteriales;f Natrialbaceae;g Natronococcus;s Natronococcus sp003559215
Gb0054156-2MAG06	No	85.17	3.15	7915600	1928	4714	61.5	86.13	7810	d	Bacteria;p	Planctomycetota;c Planctomycetia;o Pirellulales;f Pirellulaceae;g SLMF01;s SLMF01 sp007124535
Gb0054156-2MAG07	No	84.68	1.4	2136847	223	14116	47.9	91.12	1886	d	Bacteria;p	Bacteroidota;c Bacteroidia;o Bacteroidales;f UBA7960;g PUPG01;s PUPG01 sp007124575
Gb0054156-2MAG07	No	91.28	7.77	3501584	540	10480	46.1	87.49	2972	d	Bacteria;p	Bacteroidota;c Bacteroidia;o Chitinophagales;f Saprospiraceae;g PWJY01;s PWJY01 sp007121335
Gb0054156-2MAG07	No	82.55	3.29	1751906	588	3479	40.9	86.67	2158	d	Archaea;p	Halobacteriota;c Methanosarcinia;o Methanosarcinales;f Methanosarcinaceae;g Methanolobus;s Methanolobus sp003565715
Gb0054156-2MAG07	No	90.94	8.66	2294916	491	5807	35.7	90.61	2590	d	Bacteria;p	Firmicutes F;c Halanaerobiia;o Halanaerobiales;f Halarsenatibacteraceae;g SLSL01;s SLSL01 sp007130465
Gb0054156-2MAG07	Yes	79.16	4.8	2666854	567	5599	61.7	91.21	2782	d	Bacteria;p	Proteobacteria;c Gammaproteobacteria;o SLND01;f SLND01;g SLPV01;s SLPV01 sp003558125
Gb0054156-2MAG07	Yes	91.9	3.78	3017782	205	27635	60.6	87.99	2799	d	Bacteria;p	Proteobacteria;c Gammaproteobacteria;o SLND01;f SLND01;g SLPV01;s

Gb0054156-2MAG078	Yes	89.84	3.81	3811725	329	22063	70.9	91.78	3788	d	Bacteria;p Deinococcota;c Deinococci;o Deinococcales;f Trueperaceae;g CSSed10-48;s CSSed10-48 sp003556805
Gb0054156-2MAG081	No	70.88	1.61	1182416	223	6382	55	92.7	1388	d	Archaea;p Thermoplasmatota;c Thermoplasmata;o Methanomassiliicoccales;f Methanomethylophilaceae;g PWHV01;s PWHV01 sp003557905
Gb0054156-2MAG088	No	72.94	5.65	2827627	1377	2128	63.7	93.78	3787	d	Bacteria;p Spirochaetota;c Spirochaetia;o DSM-27196;f SLST01;g SLST01;s SLST01 sp007127335
Gb0054156-2MAG092	Yes	90.36	1.33	1359521	197	10512	41	93.51	1475	d	Bacteria;p Firmicutes;c Bacilli;o Izemoplasmatales;f Izemoplasmataceae;g PWME01;s PWME01 sp003559235
Gb0054156-2MAG096	Yes	70.52	4.48	2813435	375	10398	45.6	87.29	2445	d	Bacteria;p Bacteroidota;c Bacteroidia;o Chitinophagales;f Saprospiraceae;g PWJY01;s PWJY01 sp003559985
Gb0054156-2MAG111	Yes	91.44	8.88	5847197	578	21579	44.3	86.39	5292	d	Bacteria;p Bacteroidota;c Rhodothermia;o Balneolales;f Balneolaceae;g UBA2664;s UBA2664 sp007123785
Gb0054156-2MAG125	Yes	84.8	1.89	2201696	309	9183	55.2	93.11	2268	d	Bacteria;p Spirochaetota;c Spirochaetia;o PWKH01;f PWKH01;g PWKH01;s PWKH01 sp003559765
Gb0054156-2MAG127	No	88.72	5.73	4007610	600	9537	71.4	88.11	4186	d	Bacteria;p Actinobacteriota;c Actinomycetia;o Nitriliruptorales;f Nitriliruptoraceae;g T1Sed10-7;s T1Sed10-7 sp003561535
Gb0054156-2MAG135	No	94.08	1.88	2643624	181	30470	67	91.72	2547	d	Bacteria;p Proteobacteria;c Gammaproteobacteria;o Nitrococcales;f Halorhodospiraceae;g Alkalilimnicola;s Alkalilimnicola mobilis
Gb0054156-2MAG146	Yes	77.98	3.01	3972569	336	30085	44.2	85.79	3500	d	Bacteria;p Bacteroidota;c Rhodothermia;o Balneolales;f Balneolaceae;g UBA2664;s UBA2664 sp007131725
Gb0054156-2MAG172	No	79.45	6.14	3547244	530	9693	70.8	90.65	3652	d	Bacteria;p Deinococcota;c Deinococci;o Deinococcales;f Trueperaceae;g CSSed10-48;s CSSed10-48 sp003567075
Gb0054157MAG001	Yes	99.4	4.76	2660744	123	54545	49	84.19	2580	d	Bacteria;p Desulfobacterota I;c Desulfovibrionia;o Desulfovibrionales;f Desulfonatronovibrionaceae;g Desulfonatronospira;s Desulfonatronospira sp007125075
Gb0054157MAG002	Yes	97.59	0.54	2876763	113	50296	33.4	89.48	2630	d	Bacteria;p Bacteroidota;c Bacteroidia;o Flavobacteriales;f Flavobacteriaceae;g Psychroflexus;s Psychroflexus sp003564185
Gb0054157MAG003	Yes	97.85	2.42	2968788	171	30402	37.8	89.16	2529	d	Bacteria;p Bacteroidota;c Bacteroidia;o Bacteroidales;f PXBB01;g PXBB01;s PXBB01 sp003565735
Gb0054157MAG004	No	91.87	1.29	3040058	191	25986	55.4	90.85	3025	d	Bacteria;p Proteobacteria;c Gammaproteobacteria;o Competibacterales;f Competibacteraceae;g SKOM01;s SKOM01 sp007120095
Gb0054157MAG005	Yes	90.61	4.73	3493505	253	22504	68	92.7	3340	d	Bacteria;p Proteobacteria;c Gammaproteobacteria;o PWYM01;f PWYM01;g PWYM01;s PWYM01 sp003567855
Gb0054157MAG006	Yes	95.83	3.57	2434644	224	17773	52.8	86.68	2380	d	Bacteria;p Desulfobacterota I;c Desulfovibrionia;o Desulfovibrionales;f Desulfonatronovibrionaceae;g Desulfonatronospira;s Desulfonatronospira sp003553695
Gb0054157MAG007	Yes	93.83	1.55	2661623	149	45926	44.4	89.9	2655	d	Bacteria;p Firmicutes F;c Halanaerobiia;o B1SED10-174;f B1SED10-174;g T1SED10-26;s T1SED10-26 sp007122335
Gb0054157MAG008	Yes	96.11	4.42	2741711	150	31102	58.5	89.6	2537	d	Bacteria;p Verrucomicrobiota;c Kiritimatiellae;o SLAD01;f SLAD01;g SLLF01;s SLLF01 sp003562335
Gb0054157MAG009	No	91.39	1.42	3241922	271	17566	58.4	89.48	2812	d	Bacteria;p Verrucomicrobiota;c Kiritimatiellae;o SS1-B-03-39;f UBA6053;g PXAS01;s PXAS01 sp003565815
Gb0054157MAG010	Yes	93.4	1.91	1990849	197	15057	53.5	87.04	1978	d	Bacteria;p Firmicutes D;c Dethiobacteria;o DTU022;f UBA8154;g CSSED11-109;s CSSED11-109 sp003563385
Gb0054157MAG011	Yes	89.7	4.89	3815634	617	14545	51.6	89.32	3852	d	Bacteria;p Verrucomicrobiota;c Kiritimatiellae;o UBA8416;f UBA8416;g T3Sed10-140;s T3Sed10-140 sp003567805
Gb0054157MAG012	No	95.53	0	2265187	297	10951	47.7	88.05	2342	d	Bacteria;p Thermotogota;c Thermotogae;o Petrotogales;f Kosmotogaceae;g UBA9910;s UBA9910 sp003561215
Gb0054157MAG013	Yes	97.2	2.1	2801086	246	33812	43.9	90.22	2882	d	Bacteria;p Firmicutes A;c Clostridia;o Peptostreptococcales;f T1SED10-28;g T1SED10-28;s T1SED10-28 sp003554105
Gb0054157MAG014	Yes	98.39	1.88	3797395	204	33179	53.3	90.2	3339	d	Bacteria;p Bacteroidota;c Bacteroidia;o Flavobacteriales;f Cryomorphaceae;g T3Sed10-241;s T3Sed10-241 sp003565555
Gb0054157MAG015	Yes	97.33	1.33	1434338	69	33010	51.5	92.56	1407	d	Bacteria;p Firmicutes;c Bacilli;o Izemoplasmatales;f Izemoplasmataceae;g SLQR01;s SLQR01 sp007123975
Gb0054157MAG016	No	93.22	3.39	2192846	305	15456	55.1	88.75	2294	d	Bacteria;p Firmicutes D;c Dethiobacteria;o PWVN01;f PWVN01;g PWVN01;s PWVN01 sp007136165
Gb0054157MAG017	Yes	94.87	2.94	2552595	400	8222	55.6	87.46	2585	d	Bacteria;p Desulfobacterota;c DSM-4660;o Desulfatiglandales;f Desulfatiglandaceae;g DSCZ01;s
Gb0054157MAG018	Yes	92.32	0.65	2044069	338	7507	40.6	85.52	2234	d	Archaea;p Halobacteriota;c Methanosarcinia;o Methanosarcinales;f Methanosarcinaceae;g Methanolobus;s Methanolobus sp003565715
Gb0054157MAG019	No	89.61	0.54	2554516	147	32033	47.8	90.67	2095	d	Bacteria;p Bacteroidota;c Bacteroidia;o Bacteroidales;f UBA7960;g PUPG01;s PUPG01 sp007124575
Gb0054157MAG021	No	98.9	3.3	3999148	168	48398	67.4	91.62	3308	d	Bacteria;p Gemmatimonadota;c Gemmatimonadetes;o Longimicrobiales;f UBA6960;g PWLA01;s PWLA01 sp003567615
Gb0054157MAG022	Yes	95.01	1.09	3450472	272	26100	45.9	86.92	2927	d	Bacteria;p Bacteroidota;c Bacteroidia;o Chitinophagales;f Saprospiraceae;g PWJY01;s PWJY01 sp007121335
Gb0054157MAG024	Yes	95.69	1.26	2925740	256	19264	35.6	90.78	2711	d	Bacteria;p Bacteroidota;c Bacteroidia;o Flavobacteriales;f Flavobacteriaceae;g Psychroflexus;s Psychroflexus sp003565575
Gb0054157MAG025	Yes	95.07	1.27	2257447	118	79287	54.8	86.61	2167	d	Bacteria;p Firmicutes D;c Dethiobacteria;o DTU022;f UBA8154;g SKMY01;s SKMY01 sp003556225
Gb0054157MAG026	No	83.59	1.91	3517662	187	42442	43.8	91.9	3131	d	Bacteria;p Bacteroidota;c T3Sed10-11;o T3Sed10-11;f T3Sed10-11;g T3Sed10-11;s T3Sed10-11 sp003568415
Gb0054157MAG027	Yes	92.9	0	1651921	116	28897	55.5	87.82	1723	d	Bacteria;p Chloroflexota;c Dehalococcoidia;o GIF9;f AB-539-J10;g E29-bin54;s E29-bin54 sp003567655
Gb0054157MAG028	No	88.52	3.11	2932696	224	26240	43.7	90.22	2704	d	Bacteria;p Bacteroidota;c Rhodothermia;o Balneolales;f Balneolaceae;g UBA2664;s UBA2664 sp007134085
Gb0054157MAG029	No	86.23	3.88	1988315	195	22604	44.4	87.37	1973	d	Bacteria;p Firmicutes D;c Dethiobacteria;o DTU022;f DTU022;g B1SED10-74M;s B1SED10-74M sp007128625
Gb0054157MAG030	Yes	86.38	1.08	1457477	141	17066	39.3	90.74	1530	d	Bacteria;p Omnitrophota;c Koll11;o 4484-171;f 4484-171;g PXAH01;s PXAH01 sp003565945
Gb0054157MAG031	Yes	86.61	3.57	3396344	646	6271	49.1	87.43	3226	d	Bacteria;p Bacteroidota;c Rhodothermia;o Balneolales;f PXAI01;g Cyclonatronum;s Cyclonatronum sp003566395
Gb0054157MAG033	No	96.13	7.96	3934831	253	30471	44.6	85.09	3838	d	Bacteria;p Desulfobacterota;c Desulfobacteria;o Desulfobacterales;f SKZT01;g SKZT01;s SKZT01 sp007127235
Gb0054157MAG034	No	98	2.8	3409443	218	25818	60.1	93.9	3268	d	Bacteria;p Spirochaetota;c Spirochaetia;o DSM-27196;f PWMO01;g PWMO01;s PWMO01 sp007123465
Gb0054157MAG035	Yes	97.31	7.8	4905901	469	21027	41	88.51	4279	d	Bacteria;p Bacteroidota;c Bacteroidia;o Bacteroidales;f UBA7960;g SKVR01;s SKVR01 sp007129355
Gb0054157MAG036	No	95.7	1.08	2521786	276	12996	44.8	91.31	2392	d	Bacteria;p Bacteroidota;c Bacteroidia;o Flavobacteriales;f Schleiferiaceae;g CSBr16-58;s CSBr16-58 sp003566715
Gb0054157MAG037	Yes	95.37	5.28	3002240	514	8904	46.9	85.25	3197	d	Bacteria;p Desulfobacterota I;c Desulfovibrionia;o Desulfovibrionales;f Desulfonatronovibrionaceae;g Desulfonatronovibrio;s Desulfonatronovibrio sp003566455
Gb0054157MAG038	No	72.98	0	2747352	159	46103	59	90.05	2796	d	Bacteria;p Proteobacteria;c Alphaproteobacteria;o Rhodobacterales;f Rhodobacteraceae;g Roseinatronobacter;s Roseinatronobacter monicus
Gb0054157MAG039	Yes	84.61	0	624390	38	37696	35.4	90.95	669	d	Bacteria;p Patescibacteria;c Paceibacteria;o Paceibacterales;f PWPS01;g PWHW01;s PWHW01 sp003561025
Gb0054157MAG040	Yes	85.1	2.12	2493590	310	13470	44.4	86.19	2566	d	Bacteria;p Firmicutes D;c Dethiobacteria;o DTU022;f DTU022;g T3SED10-7;s T3SED10-7 sp003564055
Gb0054157MAG041	No	94.62	7.8	2618431	109	49073	50.1	89.85	2134	d	Bacteria;p Bacteroidota;c Bacteroidia;o Bacteroidales;f UBA7960;g PUPG01;s PUPG01 sp007125585
Gb0054157MAG042	Yes	96.19	3.84	4609578	374	23555	43.3	88.06	3973	d	Bacteria;p Bacteroidota;c Bacteroidia;o Bacteroidales;f PUMT01;g PXAP01;s PXAP01 sp007120695
Gb0054157MAG043	Yes	95.6	6.69	4405683	360	22545	65.4	88.62	3942	d	Bacteria;p Gemmatimonadota;c Gemmatimonadetes;o Longimicrobiales;f UBA6960;g PXFI01;s PXFI01 sp003564295
Gb0054157MAG044	Yes	96.96	0.68	2652580	212	22525	55	89.87	2528	d	Bacteria;p Verrucomicrobiota;c Verrucomicrobiae;o Opitutales;f DSM-45221;g T3Sed10-183;s T3Sed10-183 sp003567255
Gb0054157MAG045	Yes	91.53	1.69	1882321	94	54027	61.6	90.58	1910	d	Bacteria;p Bipolaricaulota;c Bipolaricaulia;o Bipolaricaulales;f Bipolaricaulaceae;g T3SED10-47;s T3SED10-47 sp003566155
Gb0054157MAG046	Yes	91.53	0	2539654	286	14088	62.8	90.13	2728	d	Bacteria;p Bipolaricaulota;c Bipolaricaulia;o Bipolaricaulales;f Bipolaricaulaceae;g T3SED10-47;s T3SED10-47 sp003562845
Gb0054157MAG047	Yes	94.81	9.02	3973609	313	31918	42.7	90.43	3636	d	Bacteria;p Bacteroidota;c Rhodothermia;o Balneolales;f Balneolaceae;g UBA2664;s UBA2664 sp007123985
Gb0054157MAG048	No	91.55	2.49	2698152	184	27397	66.3	88.04	2640	d	Bacteria;p Proteobacteria;c Gammaproteobacteria;o Ectothiorhodospirales;f Thioalkalivibrionaceae;g Thioalkalivibrio B;s Thioalkalivibrio B sp003563455
Gb0054157MAG049	Yes	99.2	4.83	3471969	244	29069	55.5	93.62	3275	d	Bacteria;p Spirochaetota;c Spirochaetia;o DSM-27196;f SLAA01;g SLAA01;s SLAA01 sp007127105
Gb0054157MAG050	Yes	94.54	5.19	3388555	243	21587	48.3	88.67	2913	d	Bacteria;p Bacteroidota;c Rhodothermia;o Balneolales;f PXAI01;g PXAI01;s PXAI01 sp003567135
Gb0054157MAG051	Yes	88.39	1.69	774723	90	14650	29.9	93.46	844	d	Bacteria;p Firmicutes;c Bacilli;o RF39;f UBA660;g CSSED10-109M;s CSSED10-109M sp003560455
Gb0054157MAG052	Yes	91.85	2.42	3034585	451	8952	58.4	90.34	2942	d	Bacteria;p Verrucomicrobiota;c Kiritimatiellae;o SS1-B-03-39;f UBA6053;g UBA6053;s
Gb0054157MAG053	Yes	85.94	6.05	2079488	499	4919	57.2	89.72	2246	d	Bacteria;p Verrucomicrobiota;c Verrucomicrobiae;o Opitutales;f Puniceicoccaceae;g ;s
Gb0054157MAG054	No	91.25	4.29	1924291	239	12238	58.2	88.23	2034	d	Bacteria;p Chloroflexota;c Dehalococcoidia;o GIF9;f AB-539-J10;g E29-bin54;s E29-bin54 sp003556385
Gb0054157MAG055	No	80	1.98	2018163	458	5414	61.5	90.9	2208	d	Bacteria;p Proteobacteria;c Gammaproteobacteria;o UBA5335;f UBA5335;g SLRW01;s SLRW01 sp003567955
Gb0054157MAG056	Yes	76.21	6.45	3054621	1102	3232	60.7	90.1	3394	d	Bacteria;p Verrucomicrobiota;c Verrucomicrobiae;o Opitutales;f T3Sed10-336;g T3Sed10-336;s T3Sed10-336 sp003566375
Gb0054157MAG057	Yes	98.67	0.39	1261203	175	9566	54.7	91.47	1372	d	Bacteria;p Firmicutes;c Bacilli;o Izemoplasmatales;f Izemoplasmataceae;g T1SED10-81;s T1SED10-81 sp003568315
Gb0054157MAG058	No	93.33	4.34	3279925	355	12745	45.9	89.92	2871	d	Bacteria;p Bacteroidota;c Bacteroidia;o Bacteroidales;f UBA7960;g PUML01;s PUML01 sp007129255
Gb0054157MAG059	Yes	93.7	3.53	5071293	539	13626	55.4	83.88	4861	d	Bacteria;p Cyanobacteria;c Cyanobacteriia;o Phormidesmiales;f Phormidesmiaceae;g Nodosilinea;s Nodosilinea sp003564835
Gb0054157MAG060	No	93.61	3.23	2923117	482	7884	55.5	86.13	2959	d	Bacteria;p Desulfobacterota;c Syntrophobacteria;o Syntrophobacterales;f Syntrophobacteraceae;g SLCH01;s SLCH01 sp003566995
Gb0054157MAG062	No	80.08	3.37	811151	48	28158	44.2	87.03	824	d	Bacteria;p Patescibacteria;c Paceibacteria;o UBA9983 A;f CSBR16-193;g SKVL01;s SKVL01 sp007125155
Gb0054157MAG063	Yes	91.38	5.13	4512572	751	8501	71.6	88.49	4665	d	Bacteria;p Actinobacteriota;c Actinomycetia;o Nitriliruptorales;f Nitriliruptoraceae;g T1Sed10-7;s T1Sed10-7 sp003565155
Gb0054157MAG064	Yes	97.57	3.07	3594179	293	23870	57.6	94.8	3454	d	Bacteria;p Spirochaetota;c Spirochaetia;o DSM-27196;f SKKC01;g SKKC01;s SKKC01 sp007121695
Gb0054157MAG065	No	94.24	6.52	2834411	371	10495	43.8	81.9	2742	d	Bacteria;p Firmicutes F;c Halanaerobiia;o Halanaerobiales;f Halarsenatibacteraceae;g T1SED10-84;s T1SED10-84 sp007134885
Gb0054157MAG066	No	88.18	4.55	2165253	337	8905	45.4	87.75	2293	d	Bacteria;p Actinobacteriota;c Humimicrobiia;o Humimicrobiales;f Humimicrobiaceae;g Halolacustris;s Halolacustris primus
Gb0054157MAG067	Yes	94.26	2.56	3124290	469	8710	56.9	88.3	3123	d	Bacteria;p Verrucomicrobiota;c Verrucomicrobiae;o Opitutales;f DSM-45221;g WN38;s WN38 sp003566795
Gb0054157MAG068	Yes	91.83	2.93	3602280	289	22127	62.9	90.62	3645	d	Bacteria;p Proteobacteria;c Alphaproteobacteria;o Rhodobacterales;f Rhodobacteraceae;g Roseinatronobacter;s Roseinatronobacter sp007118445
Gb0054157MAG069	Yes	91.43	2.22	5706552	594	14131	65.2	91.94	4858	d	Bacteria;p Myxococcota;c Bradymonadia;o Bradymonadales;f Bradymonadaceae;g SKON01;s SKON01 sp007120035
Gb0054157MAG070	Yes	89.25	4.84	1413399	188	12601	55.3	94.35	1315	d	Bacteria;p Omnitrophota;c Koll11;o SKK-01;f SKK-01;g ;s
Gb0054157MAG071	Yes	91.82	4.09	4247892	356	24732	62.3	84.54	4091	d	Bacteria;p Chloroflexota;c Anaerolineae;o B4-G1;f B4-G1;g SKLM01;s
Gb0054157MAG072	No	76.68	2.03	2461816	674	4036	54.3	91.42	2557	d	Bacteria;p Verrucomicrobiota;c Kiritimatiellae;o UBA8416;f PXDI01;g PXDI01;s PXDI01 sp003565095
Gb0054157MAG073	No	83.54	4.06	3807572	763	6156	44.6	91.04	3998	d	Bacteria;p Bacteroidota;c T3Sed10-11;o T3Sed10-11;f T3Sed10-11;g T3Sed10-11;s T3Sed10-11 sp007128495
Gb0054157MAG074	No	79.2	1.28	1527493	428	3982	41.1	94.55	1734	d	Bacteria;p Delongbacteria;c UBA4055;o UBA4055;f UBA4055;g SLFX01;s SLFX01 sp007124045
Gb0054157MAG079	Yes	93.82	1.76	2698691	167	28771	64.3	89.65	2608	d	Bacteria;p Proteobacteria;c Gammaproteobacteria;o Nitrococcales;f Aquisalimonadaceae;g SKGQ01;s SKGQ01 sp003565775
Gb0054157MAG080	Yes	77.95	3.45	602122	64	18605	34.1	89.42	673	d	Bacteria;p Patescibacteria;c Paceibacteria;o Paceibacterales;f RBG-13-42-11;g CSSED10-83;s CSSED10-83 sp003556525
Gb0054157MAG081	Yes	73.43	2.31	3084424	761	4608	62.1	89.51	3128	d	Bacteria;p Armatimonadota;c UBA5377;o UBA5377;f UBA5352;g UBA5352;s UBA5352 sp003566775
Gb0054157MAG082	No	83.73	1.43	2158856	580	4363	44.7	91.18	2545	d	Bacteria;p Firmicutes F;c Halanaerobiia;o B1SED10-174;f B1SED10-174;g B1SED10-174;s B1SED10-174 sp003551905
Gb0054157MAG083	Yes	79.68	2.25	1253744	347	4253	43.5	93.15	1548	d	Bacteria;p CG03;c SLGR01;o SLGR01;f PWQF01;g PWMP01;s PWMP01 sp003558145
Gb0054157MAG084	Yes	73.8	3.51	1495755	417	3860	52.3	85.42	1594	d	Bacteria;p Planctomycetota;c Brocadiae;o SM23-32;f SM23-32;g PWZK01;s PWZK01 sp003567495
Gb0054157MAG085	Yes	87.28	9.69	3707384	506	15053	64.4	90.92	3333	d	Bacteria;p Verrucomicrobiota;c Verrucomicrobiae;o Verrucomicrobiales;f Akkermansiaceae;g Luteolibacter;s Luteolibacter sp007126655
Gb0054157MAG087	Yes	92.56	1.79	1622638	379	5427	39.8	94.06	1686	d	Bacteria;p Caldisericota;c Caldisericia;o UBA6126;f UBA6126;g T3SED10-260;s T3SED10-260 sp003566815
Gb0054157MAG090	No	73.41	5.51	2722215	385	11485	48	88.26	2377	d	Bacteria;p Bacteroidota;c Rhodothermia;o Balneolales;f PXAI01;g Cyclonatronum;s Cyclonatronum sp003555145

Gb0054157MAG094	Yes	75.28	0.33	2511138	719	3933	58.3	83.92	2962	d	Bacteria;p	Firmicutes E;c SLMV01;o SLMV01;f PUMD01;g PWUC01;s PWUC01 sp003566675
Gb0054157MAG100	No	93.23	4.73	7357179	1642	5339	62.3	85.6	6912	d	Bacteria;p	Planctomycetota;c Planctomycetia;o Pirellulales;f Pirellulaceae;g SLMF01;s SLMF01 sp007124535
Gb0054157MAG102	Yes	82.76	3.62	4175775	603	10131	38.9	86.36	3882	d	Bacteria;p	Bacteroidota;c Bacteroidia;o Chitinophagales;f Saprospiraceae;g PWJY01;s PWJY01 sp007134625
Gb0054157MAG107	No	90.29	8.93	3053496	531	8090	58.9	90.16	2955	d	Bacteria;p	Verrucomicrobiota;c Kiritimatiellae;o SLAD01;f SLAD01;g SKZA01;s SKZA01 sp007136425
Gb0054157MAG108	Yes	91.25	9.08	2055392	443	7542	56.7	86.4	2348	d	Bacteria;p	Chloroflexota;c Dehalococcoidia;o GIF9;f AB-539-J10;g E29-bin54;s
Gb0054157MAG109	No	77.23	5.82	2001114	317	9135	44.4	90.33	1880	d	Bacteria;p	Bacteroidota;c Bacteroidia;o Bacteroidales;f UBA7960;g SKTA01;s SKTA01 sp007123065
Gb0054157MAG112	No	93.26	6.61	3110241	210	23364	68.1	89.54	3017	d	Bacteria;p	Proteobacteria;c Alphaproteobacteria;o Rhodobacterales;f Rhodobacteraceae;g PUOA01;s PUOA01 sp007121115
Gb0054157MAG113	Yes	71.02	3.01	2101846	956	2441	42.5	88.58	2615	d	Bacteria;p	Bacteroidota;c Bacteroidia;o Flavobacteriales;f Salibacteraceae;g Salibacter;s Salibacter sp003564735
Gb0054157MAG114	No	84.96	3.53	7262090	1263	6629	59.7	86.96	6608	d	Bacteria;p	Planctomycetota;c Planctomycetia;o Pirellulales;f Pirellulaceae;g SKKK01;s SKKK01 sp007121535
Gb0054157MAG118	Yes	90.54	8.3	3616641	649	7050	56.7	89.61	3253	d	Bacteria;p	Verrucomicrobiota;c Kiritimatiellae;o PXCX01;f PXCX01;g PXCX01;s PXCX01 sp003567575
Gb0054157MAG125	Yes	92.26	4.42	3323719	204	30337	60.3	92.01	3016	d	Bacteria;p	Proteobacteria;c Gammaproteobacteria;o Xanthomonadales;f Wenzhouxiangellaceae;g Wenzhouxiangella;s Wenzhouxiangella sp003561495
Gb0054157MAG126	No	80.3	4.85	2184715	453	6144	35.8	90.61	2465	d	Bacteria;p	Firmicutes F;c Halanaerobiia;o Halanaerobiales;f Halarsenatibacteraceae;g SLSL01;s SLSL01 sp007130465
Gb0054157MAG131	No	86.91	5.34	3127458	193	33763	66.9	89.94	3051	d	Bacteria;p	Proteobacteria;c Alphaproteobacteria;o Rhodobacterales;f Rhodobacteraceae;g Roseinatronobacter;s Roseinatronobacter sp003564655
Gb0054157MAG163	No	96.99	4.32	3685399	267	22195	45.2	87.03	3238	d	Bacteria;p	Bacteroidota;c Rhodothermia;o Balneolales;f Balneolaceae;g QGGB01;s QGGB01 sp007118525
Gb0054157MAG164	No	85.79	8.2	3728766	373	15259	43.1	86.82	3391	d	Bacteria;p	Bacteroidota;c Rhodothermia;o Balneolales;f Balneolaceae;g SLAU01;s SLAU01 sp007126675
Gb0054157MAG176	No	88.96	0.85	2938080	301	15181	72.3	90.09	2834	d	Bacteria;p	Actinobacteriota;c Actinomycetia;o Nitriliruptorales;f Nitriliruptoraceae;g T1Sed10-7;s T1Sed10-7 sp003554005
Gb0054158MAG001	Yes	94.58	2.78	1747040	68	67806	63	90.54	1717	d	Bacteria;p	Actinobacteriota;c Coriobacteriia;o OPB41;f SLCP01;g PWVH01;s
Gb0054158MAG002	No	95.61	1.37	3948621	182	40802	43.9	91.61	3599	d	Bacteria;p	Bacteroidota;c T3Sed10-11;o T3Sed10-11;f T3Sed10-11;g T3Sed10-11;s T3Sed10-11 sp003568415
Gb0054158MAG003	Yes	98.12	0	2160577	112	35938	47.8	89.28	2095	d	Bacteria;p	Thermotogota;c Thermotogae;o Petrotogales;f Kosmotogaceae;g UBA9910;s UBA9910 sp003561215
Gb0054158MAG004	No	90.44	0	3050937	244	16417	59.3	88.77	2743	d	Bacteria;p	Proteobacteria;c Gammaproteobacteria;o Chromatiales;f Sedimenticolaceae;g SLIM01;s SLIM01 sp007135625
Gb0054158MAG005	No	93.83	2.82	2743076	121	64857	44.3	89.78	2694	d	Bacteria;p	Firmicutes F;c Halanaerobiia;o B1SED10-174;f B1SED10-174;g T1SED10-26;s T1SED10-26 sp007122335
Gb0054158MAG006	Yes	95	0.91	2032290	71	63320	42.2	91.17	2062	d	Bacteria;p	Actinobacteriota;c Humimicrobiia;o Humimicrobiales;f Humimicrobiaceae;g Halolacustris;s Halolacustris secundus
Gb0054158MAG007	No	94.43	1.98	3475667	273	23201	45.7	86.75	2946	d	Bacteria;p	Bacteroidota;c Bacteroidia;o Chitinophagales;f Saprospiraceae;g PWJY01;s PWJY01 sp007121335
Gb0054158MAG008	No	93.22	4.45	1945362	152	28470	44.2	90.04	2024	d	Bacteria;p	Firmicutes D;c Dethiobacteria;o SKNC01;f SKNC01;g SKLX01;s SKLX01 sp007121375
Gb0054158MAG009	Yes	95.47	0.97	3291410	291	18760	64.3	91.45	3193	d	Bacteria;p	Proteobacteria;c Gammaproteobacteria;o Chromatiales;f Chromatiaceae;g Halochromatium;s Halochromatium sp003562815
Gb0054158MAG010	Yes	99.04	3.3	3504533	483	9785	51.5	81.37	3191	d	Bacteria;p	Desulfobacterota I;c Desulfovibrionia;o Desulfovibrionales;f Desulfonatronovibrionaceae;g Desulfonatronospira;s Desulfonatronospira sp003563255
Gb0054158MAG011	No	93.1	5.11	2637615	458	7353	55.6	87.48	2711	d	Bacteria;p	Desulfobacterota;c DSM-4660;o Desulfatiglandales;f Desulfatiglandaceae;g DSCZ01;s
Gb0054158MAG012	Yes	95.7	0.54	2491596	79	55023	50.1	90	2024	d	Bacteria;p	Bacteroidota;c Bacteroidia;o Bacteroidales;f UBA7960;g PUPG01;s PUPG01 sp007125585
Gb0054158MAG013	Yes	94.42	4.31	2538995	235	19622	54.6	80.9	2401	d	Bacteria;p	Firmicutes D;c Dethiobacteria;o DTU022;f UBA8154;g CSSED11-181;s CSSED11-181 sp003563895
Gb0054158MAG014	Yes	91.09	1.98	2313974	234	16324	62.1	90.36	2398	d	Bacteria;p	Firmicutes E;c SLMV01;o ;f ;g ;s
Gb0054158MAG015	Yes	90.32	1.99	4575734	590	10339	68.1	89.4	4794	d	Bacteria;p	Proteobacteria;c Alphaproteobacteria;o Geminicoccales;f Geminicoccaceae;g SLRI01;s
Gb0054158MAG016	No	98.71	5.59	4408782	327	28426	44.7	84.96	4325	d	Bacteria;p	Desulfobacterota;c Desulfobacteria;o Desulfobacterales;f SKZT01;g SKZT01;s SKZT01 sp007127235
Gb0054158MAG017	No	86.62	3.18	2018842	131	31188	52.7	85.06	1982	d	Bacteria;p	Firmicutes D;c Dethiobacteria;o DTU022;f UBA8154;g CSSED11-109;s CSSED11-109 sp003563385
Gb0054158MAG018	No	90.18	3.54	3155828	220	25647	55.4	91.06	3186	d	Bacteria;p	Proteobacteria;c Gammaproteobacteria;o Competibacterales;f Competibacteraceae;g SKOM01;s SKOM01 sp007120095
Gb0054158MAG019	Yes	82.67	0.5	1743034	127	24566	61.4	88.66	1775	d	Bacteria;p	Firmicutes E;c SLMV01;o SLMV01;f PUMD01;g PWUB01;s PWUB01 sp003563755
Gb0054158MAG020	Yes	96.61	0	2791086	213	24524	49.2	87.33	2841	d	Bacteria;p	Atribacterota;c Atribacteria;o Atribacterales;f Atribacteraceae;g SKXZ01;s SKXZ01 sp003561145
Gb0054158MAG021	No	91.25	0.99	1651446	156	18801	58.1	88.16	1683	d	Bacteria;p	Chloroflexota;c Dehalococcoidia;o GIF9;f AB-539-J10;g E29-bin54;s E29-bin54 sp003556385
Gb0054158MAG022	Yes	95	6.46	2240232	381	7370	45.2	87.58	2438	d	Bacteria;p	Actinobacteriota;c Humimicrobiia;o Humimicrobiales;f Humimicrobiaceae;g Halolacustris;s Halolacustris primus
Gb0054158MAG023	No	80.36	1.08	1709767	423	4961	62.9	89.31	1878	d	Bacteria;p	Firmicutes E;c SLMV01;o SLMV01;f SLMV01;g CSSED11-154;s CSSED11-154 sp003563985
Gb0054158MAG024	Yes	85.08	1.67	2194630	562	4397	61.2	90.65	2555	d	Bacteria;p	Actinobacteriota;c Coriobacteriia;o OPB41;f SLCP01;g PWVH01;s PWVH01 sp003563465
Gb0054158MAG025	Yes	86.17	1.48	2113439	285	13958	45.3	86.11	2175	d	Bacteria;p	Firmicutes D;c Dethiobacteria;o DTU022;f UBA8154;g SLJK01;s SLJK01 sp007135125
Gb0054158MAG026	No	78.8	3.11	3462753	690	5971	39.8	87.48	3106	d	Bacteria;p	Bacteroidota;c Bacteroidia;o Chitinophagales;f Saprospiraceae;g PWJY01;s PWJY01 sp007134625
Gb0054158MAG028	Yes	91.75	0.99	2367275	177	22904	58.4	85.86	2471	d	Bacteria;p	Chloroflexota;c Dehalococcoidia;o GIF9;f AB-539-J10;g SKVJ01;s SKVJ01 sp003562475
Gb0054158MAG029	No	85.89	3.13	2333867	213	18865	64.7	90.3	2269	d	Bacteria;p	PUNC01;c PUNC01;o PUNC01;f PWXJ01;g PWXJ01;s PWXJ01 sp003561965
Gb0054158MAG030	Yes	100	3.33	4106933	124	61527	59.3	89.86	3838	d	Bacteria;p	Spirochaetota;c Spirochaetia;o DSM-27196;f SLAA01;g B1Sed10-166;s B1Sed10-166 sp003556605
Gb0054158MAG031	Yes	97.8	8.24	3643661	257	30928	65	90.67	3214	d	Bacteria;p	Gemmatimonadota;c Gemmatimonadetes;o Longimicrobiales;f UBA6960;g PWLA01;s PWLA01 sp003562075
Gb0054158MAG032	Yes	99.95	5.75	3430562	309	16187	60.1	94.04	3366	d	Bacteria;p	Spirochaetota;c Spirochaetia;o DSM-27196;f PWMO01;g PWMO01;s PWMO01 sp007123465
Gb0054158MAG034	Yes	93.33	5.45	3782533	384	20902	63	86.43	3539	d	Bacteria;p	Chloroflexota;c Anaerolineae;o B4-G1;f B4-G1;g SKLM01;s
Gb0054158MAG035	No	81.14	2.05	2221529	204	23290	50.8	87.24	2127	d	Bacteria;p	Firmicutes D;c Dethiobacteria;o DTU022;f DTU022;g B1SED10-74M;s B1SED10-74M sp003554395
Gb0054158MAG036	No	91.25	2.97	1998681	80	50090	56.3	86.28	2051	d	Bacteria;p	Chloroflexota;c Dehalococcoidia;o GIF9;f AB-539-J10;g SKVJ01;s SKVJ01 sp007129495
Gb0054158MAG037	No	88.41	4.07	2619726	581	5192	49.4	85.5	2776	d	Bacteria;p	Desulfobacterota I;c Desulfovibrionia;o Desulfovibrionales;f Desulfonatronovibrionaceae;g Desulfonatronospira;s Desulfonatronospira sp007125075
Gb0054158MAG038	No	80.33	1.28	2678261	222	20489	43.7	89.69	2511	d	Bacteria;p	Bacteroidota;c Rhodothermia;o Balneolales;f Balneolaceae;g UBA2664;s UBA2664 sp007134085
Gb0054158MAG039	Yes	83.05	3.39	1565478	224	14989	65.6	91.14	1678	d	Bacteria;p	Bipolaricaulota;c Bipolaricaulia;o Bipolaricaulales;f Bipolaricaulaceae;g PWTC01;s PWTC01 sp003563005
Gb0054158MAG040	No	82.58	0.91	3824488	877	5338	61.1	88.29	3762	d	Bacteria;p	Chloroflexota;c Anaerolineae;o B4-G1;f SLSP01;g SLSP01;s SLSP01 sp007130345
Gb0054158MAG041	Yes	70.98	0.31	537630	62	12054	40.9	90.6	647	d	Bacteria;p	Patescibacteria;c Paceibacteria;o Paceibacterales;f MHMH01;g ;s
Gb0054158MAG044	Yes	93.1	2.68	5327958	858	8503	65.8	84.11	4981	d	Bacteria;p	Planctomycetota;c Planctomycetia;o Pirellulales;f Thermoguttaceae;g PWXP01;s PWXP01 sp003561895
Gb0054158MAG045	No	89.09	2.41	2277569	462	6111	45	83.99	2253	d	Bacteria;p	Firmicutes F;c Halanaerobiia;o Halanaerobiales;f Halarsenatibacteraceae;g T1SED10-84;s T1SED10-84 sp007134885
Gb0054158MAG046	Yes	93.58	6.05	2239712	315	9941	51.9	86.19	2345	d	Bacteria;p	Desulfobacterota I;c Desulfovibrionia;o Desulfovibrionales;f Desulfonatronovibrionaceae;g Desulfonatronospira;s
Gb0054158MAG048	Yes	92.73	8.82	5803335	890	9429	63.1	84.36	5625	d	Bacteria;p	Chloroflexota;c Anaerolineae;o B4-G1;f B4-G1;g SKLM01;s SKLM01 sp007123205
Gb0054158MAG049	No	82.81	4.44	3614441	623	8174	70.7	88.4	3867	d	Bacteria;p	Actinobacteriota;c Actinomycetia;o Nitriliruptorales;f Nitriliruptoraceae;g CSSed11-175R1;s
Gb0054158MAG050	Yes	76.26	2.27	1835620	542	3883	40.8	86.37	2097	d	Bacteria;p	Actinobacteriota;c Humimicrobiia;o Humimicrobiales;f Humimicrobiaceae;g Halolacustris;s
Gb0054158MAG051	No	89.78	5.38	2399902	187	27247	48.9	93.66	2117	d	Bacteria;p	Bacteroidota;c Bacteroidia;o Bacteroidales;f UBA7960;g PUPG01;s PUPG01 sp003557385
Gb0054158MAG052	No	86.02	7.21	3255484	530	9378	65.6	89.66	3114	d	Bacteria;p	Gemmatimonadota;c Gemmatimonadetes;o Longimicrobiales;f UBA6960;g PXFI01;s PXFI01 sp003564295
Gb0054158MAG055	No	87.48	3.58	2189374	460	6017	57.4	89.7	2262	d	Bacteria;p	Planctomycetota;c Phycisphaerae;o Sedimentisphaerales;f Anaerohalosphaeraceae;g T3Sed10-213;s T3Sed10-213 sp007121565
Gb0054158MAG059	Yes	80.04	2.32	1666487	634	3224	58.2	95.41	2202	d	Bacteria;p	Campylobacterota;c Campylobacteria;o Campylobacterales;f SZUA-545;g CSSED11-302;s CSSED11-302 sp003561815
Gb0054158MAG063	No	88.11	2.44	1916974	382	6065	55	89.1	2100	d	Bacteria;p	Firmicutes D;c Dethiobacteria;o PWVN01;f PWVN01;g PWVN01;s PWVN01 sp007136165
Gb0054158MAG064	Yes	81.36	7.35	1408654	274	6783	66	91.65	1580	d	Bacteria;p	Bipolaricaulota;c Bipolaricaulia;o Bipolaricaulales;f Bipolaricaulaceae;g PWTC01;s
Gb0054158MAG075	Yes	74.12	0.93	881602	216	4786	39.9	91.09	1127	d	Archaea;p	Nanoarchaeota;c Nanoarchaeia;o Woesearchaeales;f CG1-02-33-12;g PWVO01;s PWVO01 sp003561825
Gb0054158MAG076	Yes	72.47	1.36	3573508	800	5009	60.1	88.55	3643	d	Bacteria;p	Chloroflexota;c Anaerolineae;o B4-G1;f SLSP01;g PWVB01;s PWVB01 sp003562365
Gb0054158MAG080	No	79.78	3.94	2208766	855	2969	55.2	93.89	2766	d	Bacteria;p	Proteobacteria;c Gammaproteobacteria;o Chromatiales;f Chromatiaceae;g Thiorhodovibrio B;s Thiorhodovibrio B sp007128075
Gb0054158MAG083	No	75.99	7.01	1437911	366	4701	63.8	91.58	1768	d	Bacteria;p	Bipolaricaulota;c Bipolaricaulia;o Bipolaricaulales;f Bipolaricaulaceae;g T3SED10-47;s T3SED10-47 sp003562845
Gb0054158MAG085	No	84.99	2.22	4600481	485	15278	55.2	83.83	4459	d	Bacteria;p	Cyanobacteria;c Cyanobacteriia;o Phormidesmiales;f Phormidesmiaceae;g Nodosilinea;s Nodosilinea sp007135385
Gb0054158MAG088	No	87.95	9.28	8002119	1825	5275	62.6	85.37	7688	d	Bacteria;p	Planctomycetota;c Planctomycetia;o Pirellulales;f Pirellulaceae;g SLMF01;s SLMF01 sp007124535
Gb0054158MAG097	Yes	80.88	9.09	945866	140	8433	48	89.04	1064	d	Bacteria;p	Patescibacteria;c Saccharimonadia;o Saccharimonadales;f SLHW01;g ;s
Gb0054158MAG107	Yes	76.39	6.94	2591998	1148	2528	47.4	87.66	2918	d	Bacteria;p	Desulfobacterota I;c Desulfovibrionia;o Desulfovibrionales;f Desulfonatronovibrionaceae;g Desulfonatronovibrio;s
Gb0054158MAG111	No	77.1	4.04	4199504	1793	2600	56.8	90.15	4944	d	Bacteria;p	Chloroflexota;c Anaerolineae;o Promineofilales;f Promineofilaceae;g SLGK01;s SLGK01 sp007123775
Gb0054158MAG112	No	85.57	2.4	2989433	1186	2814	57.5	94.52	3691	d	Bacteria;p	Spirochaetota;c Spirochaetia;o DSM-27196;f SKKC01;g SKKC01;s SKKC01 sp007121695
Gb0054158MAG117	No	94.09	5.49	4481605	407	25606	67.2	90.17	3849	d	Bacteria;p	Gemmatimonadota;c Gemmatimonadetes;o Longimicrobiales;f UBA6960;g PWLA01;s PWLA01 sp007121775
Gb0054158MAG120	No	73.67	7.99	2764912	548	6002	67.7	89.71	2927	d	Bacteria;p	Proteobacteria;c Alphaproteobacteria;o Rhodobacterales;f Rhodobacteraceae;g PUOA01;s PUOA01 sp007121115
Gb0054158MAG130	No	90.43	3.4	3302723	530	8699	64.3	90.82	3576	d	Bacteria;p	Proteobacteria;c Alphaproteobacteria;o Rhodobacterales;f Rhodobacteraceae;g Roseinatronobacter;s Roseinatronobacter sp003561595
Gb0054158MAG135	Yes	84.63	9.82	3952991	810	6480	71	90.66	4316	d	Bacteria;p	Deinococcota;c Deinococci;o Deinococcales;f Trueperaceae;g CSSed10-48;s CSSed10-48 sp003567075
Gb0054158MAG142	No	92.95	8.62	3458543	231	24397	67.5	92.16	2946	d	Bacteria;p	Gemmatimonadota;c Gemmatimonadetes;o Longimicrobiales;f UBA6960;g PWLA01;s PWLA01 sp003567615
Gb0054158MAG170	Yes	83.33	5.62	3490817	416	13157	61.5	91.52	3366	d	Bacteria;p	Proteobacteria;c Gammaproteobacteria;o Xanthomonadales;f Wenzhouxiangellaceae;g Wenzhouxiangella;s Wenzhouxiangella sp007123575
SRR7083929MAG001	Yes	92.62	1.31	2307215	50	141828	57.4	89.75	2408	d	Archaea;p	Halobacteriota;c Halobacteria;o Halobacteriales;f SKSH01;g SKSH01;s SKSH01 sp007118145
SRR7083929MAG002	No	97.33	1.33	1516409	32	99588	47.1	92.72	1488	d	Bacteria;p	Firmicutes;c Bacilli;o Izemoplasmatales;f Izemoplasmataceae;g B1SED10-225;s B1SED10-225 sp007135105
SRR7083929MAG003	Yes	98.78	0.91	3014852	118	61005	59.2	91.55	2976	d	Bacteria;p	Proteobacteria;c Alphaproteobacteria;o Rhodobacterales;f Rhodobacteraceae;g Roseinatronobacter;s Roseinatronobacter sp007125005
SRR7083929MAG004	No	92.88	1.64	3970721	163	41583	44	91.46	3595	d	Bacteria;p	Bacteroidota;c T3Sed10-11;o T3Sed10-11;f T3Sed10-11;g T3Sed10-11;s T3Sed10-11 sp003568415
SRR7083929MAG005	Yes	98.39	2.83	2873276	147	37291	52.3	89.76	2741	d	Bacteria;p	Desulfobacterota;c Desulfuromonadia;o Desulfuromonadales;f Geopsychrobacteraceae;g Pelovirga;s Pelovirga sp007125165
SRR7083929MAG006	No	95.02	0.83	3111982	162	34180	64.3	91.49	2941	d	Bacteria;p	Proteobacteria;c Gammaproteobacteria;o Chromatiales;f Chromatiaceae;g Halochromatium;s Halochromatium sp003562815
SRR7083929MAG007	No	92.8	1.98	2296168	173	27903	54.6	89.31	2329	d	Bacteria;p	Firmicutes D;c Dethiobacteria;o PWVN01;f PWVN01;g PWVN01;s PWVN01 sp007136165

SRR7083929MAG008	No	94.67	0	1360317	80	25558	51.7	92.92	1354	d	Bacteria;p	Firmicutes;c	Bacilli;o	Izemoplasmatales;f	Izemoplasmataceae;g	SLQR01;s	SLQR01 sp007123975
SRR7083929MAG009	Yes	93.55	0	1334615	83	24183	55.4	92.92	1429	d	Archaea;p	Thermoplasmatota;c	Thermoplasmata;o	Methanomassiliicoccales;f	Methanomethylophilaceae;g	PWHV01;s	PWHV01 sp003557905
SRR7083929MAG010	No	96.77	0.27	2613422	107	52489	44.9	93.61	2162	d	Bacteria;p	Bacteroidota;c	Bacteroidia;o	Bacteroidales;f	UBA7960;g	SKRM01;s	SKRM01 sp007118495
SRR7083929MAG011	Yes	96.7	4.6	4258091	253	51389	68.6	90.35	3837	d	Bacteria;p	Gemmatimonadota;c	Gemmatimonadetes;o	Longimicrobiales;f	RSA9;g	SLCC01;s	SLCC01 sp007126035
SRR7083929MAG012	Yes	97.01	1.72	2700182	167	31299	69.4	90.69	2965	d	Bacteria;p	Cyanobacteria;c	Cyanobacteriia;o	PCC-6307;f	Cyanobiaceae;g	NIES-981;s	NIES-981 sp007135755
SRR7083929MAG013	No	94.09	2.39	2832454	164	26209	58.1	90.94	2444	d	Bacteria;p	Verrucomicrobiota;c	Kiritimatiellae;o	SLAD01;f	SLAD01;g	SLAD01;s	SLAD01 sp007123265
SRR7083929MAG014	No	95.52	3.4	4869647	443	16657	67.4	85.59	3862	d	Bacteria;p	Verrucomicrobiota;c	Verrucomicrobiae;o	Verrucomicrobiales;f	SKLO01;g	SKLO01;s	SKLO01 sp007123195
SRR7083929MAG015	No	91.58	2.97	1744351	192	13971	63.4	90.21	1768	d	Bacteria;p	Firmicutes E;c	SLMV01;o	SLMV01;f	SLMV01;g	CSSED11-154;s	CSSED11-154 sp003563985
SRR7083929MAG016	No	95.3	5.45	4203931	539	12704	40.2	89.77	3977	d	Bacteria;p	Bacteroidota;c	Bacteroidia;o	Cytophagales;f	Cyclobacteriaceae;g	Cecembia;s	Cecembia sp007126775
SRR7083929MAG017	No	97.9	1.45	3210682	118	42750	54.9	85.71	2893	d	Bacteria;p	Desulfobacterota;c	Syntrophobacteria;o	Syntrophobacterales;f	Syntrophobacteraceae;g	SLCH01;s	SLCH01 sp007125915
SRR7083929MAG018	Yes	95.6	0.18	2123437	140	24998	41.7	95.14	1946	d	Bacteria;p	Delongbacteria;c	UBA4055;o	UBA4055;f	UBA4055;g	SLFX01;s	SLFX01 sp007124045
SRR7083929MAG019	No	95.16	1.89	2091242	208	20022	53.2	90.54	2107	d	Bacteria;p	Proteobacteria;c	Alphaproteobacteria;o	Micavibrionales;f	Micavibrionaceae;g	UM-FILTER-47-13;s	UM-FILTER-47-13 sp007117665
SRR7083929MAG020	Yes	98.48	5.04	3952649	336	19744	64.3	88.29	3851	d	Bacteria;p	Proteobacteria;c	Alphaproteobacteria;o	Kiloniellales;f	Rhodovibrionaceae;g	SKZV01;s	SKZV01 sp007127165
SRR7083929MAG021	Yes	97.04	8.57	3486373	484	9933	49	91.52	2962	d	Bacteria;p	Bacteroidota;c	Bacteroidia;o	Bacteroidales;f	PWNQ01;g	SKPS01;s	SKPS01 sp007119395
SRR7083929MAG022	Yes	95.63	3.55	4327449	224	42299	47.4	86.01	3771	d	Bacteria;p	Bacteroidota;c	Rhodothermia;o	Balneolales;f	Balneolaceae;g	SKRC01;s	SKRC01 sp007118695
SRR7083929MAG023	Yes	96.67	0.54	3438979	143	36337	51.5	90.78	2976	d	Bacteria;p	Bacteroidota;c	Bacteroidia;o	Flavobacteriales;f	Cryomorphaceae;g	T3Sed10-241;s	T3Sed10-241 sp007125175
SRR7083929MAG024	No	91.25	0	1620350	104	27372	58.1	88.06	1635	d	Bacteria;p	Chloroflexota;c	Dehalococcoidia;o	GIF9;f	AB-539-J10;g	E29-bin54;s	E29-bin54 sp003556385
SRR7083929MAG025	Yes	87.2	2.39	2802224	193	20791	51.8	90.84	2550	d	Bacteria;p	Verrucomicrobiota;c	Kiritimatiellae;o	UBA8416;f	UBA8416;g	T3Sed10-140;s	T3Sed10-140 sp007129405
SRR7083929MAG026	No	99.17	5.2	3091361	277	18228	61.4	92.9	2921	d	Bacteria;p	Spirochaetota;c	Spirochaetia;o	DSM-27196;f	SLAA01;g	B1Sed10-166;s	B1Sed10-166 sp007126415
SRR7083929MAG027	Yes	91.36	4.9	2871466	444	13687	54.9	93.46	2925	d	Bacteria;p	Proteobacteria;c	Gammaproteobacteria;o	Chromatiales;f	Chromatiaceae;g	Thiorhodovibrio B;s	Thiorhodovibrio B sp007128075
SRR7083929MAG028	Yes	97.97	2.73	4246724	531	10873	63	88.68	3697	d	Bacteria;p	Verrucomicrobiota;c	Verrucomicrobiae;o	Opitutales;f	Verruco-01;g	SLLG01;s	SLLG01 sp007123395
SRR7083929MAG029	Yes	92.1	4.31	3063328	446	9373	53.8	91.64	3202	d	Bacteria;p	Proteobacteria;c	Gammaproteobacteria;o	Competibacterales;f	Competibacteraceae;g	SKOM01;s	SKOM01 sp003562535
SRR7083929MAG031	No	93.83	4.94	2730375	175	51385	44.2	89.77	2712	d	Bacteria;p	Firmicutes F;c	Halanaerobiia;o	B1SED10-174;f	B1SED10-174;g	T1SED10-26;s	T1SED10-26 sp007122335
SRR7083929MAG032	No	92.2	2.15	3382135	152	46218	45.8	89.48	2755	d	Bacteria;p	Bacteroidota;c	Bacteroidia;o	Bacteroidales;f	UBA7960;g	PUML01;s	PUML01 sp007129255
SRR7083929MAG033	Yes	98.9	6.59	3437134	205	33749	68.2	91.44	3060	d	Bacteria;p	Gemmatimonadota;c	Gemmatimonadetes;o	Longimicrobiales;f	UBA6960;g	SLBA01;s	SLBA01 sp007130605
SRR7083929MAG034	No	96.24	0.54	3474934	193	28115	53.4	90.63	3056	d	Bacteria;p	Bacteroidota;c	Bacteroidia;o	Flavobacteriales;f	Cryomorphaceae;g	T3Sed10-241;s	T3Sed10-241 sp003565555
SRR7083929MAG035	Yes	90.03	2.39	3990035	338	21334	62.2	87.4	3568	d	Bacteria;p	Verrucomicrobiota;c	Kiritimatiellae;o	SLOV01;f	SLOV01;g	SLOV01;s	SLOV01 sp007132345
SRR7083929MAG036	Yes	83.03	0.91	3928529	494	13856	61.7	88.42	3455	d	Bacteria;p	Chloroflexota;c	Anaerolineae;o	B4-G1;f	SLSP01;g	SLSP01;s	SLSP01 sp007130345
SRR7083929MAG037	Yes	94.71	3.27	3425254	399	12066	49.6	92.21	3144	d	Bacteria;p	Bacteroidota;c	Bacteroidia;o	Flavobacteriales;f	Cryomorphaceae;g	SKUL01;s	SKUL01 sp007129985
SRR7083929MAG040	Yes	97.32	8.04	2872150	148	31627	51.3	92.54	2802	d	Bacteria;p	Bdellovibrionota;c	Bdellovibrionia;o	Bdellovibrionales;f	UBA6776;g	SKYF01;s	SKYF01 sp007128005
SRR7083929MAG041	No	91.36	5.37	3052752	192	25685	55.5	91.24	3003	d	Bacteria;p	Proteobacteria;c	Gammaproteobacteria;o	Competibacterales;f	Competibacteraceae;g	SKOM01;s	SKOM01 sp007120095
SRR7083929MAG042	Yes	82.24	2.12	1491726	186	9827	53.1	93.05	1668	d	Bacteria;p	Firmicutes D;c	Proteinivoracia;o	UBA4975;f	PWLI01;g	PWLI01;s	
SRR7083929MAG045	Yes	95.48	2.14	3982294	210	31319	48.8	89.35	3265	d	Bacteria;p	Bacteroidota;c	Bacteroidia;o	Bacteroidales;f	PUMT01;g	PXAP01;s	PXAP01 sp007119075
SRR7083929MAG046	No	96.4	2.94	10018009	701	25189	60.1	85.7	8425	d	Bacteria;p	Planctomycetota;c	Planctomycetia;o	Pirellulales;f	Pirellulaceae;g	SKKK01;s	SKKK01 sp007121535
SRR7083929MAG047	No	93.85	1.98	3402434	246	20552	45.8	87.02	2871	d	Bacteria;p	Bacteroidota;c	Bacteroidia;o	Chitinophagales;f	Saprospiraceae;g	PWJY01;s	PWJY01 sp007121335
SRR7083929MAG048	No	94.11	0.54	3987989	270	20339	52	87.75	3629	d	Bacteria;p	Cyanobacteria;c	Cyanobacteriia;o	Cyanobacteriales;f	Geitlerinemaceae;g	Phormidium A;s	Phormidium A sp007126595
SRR7083929MAG049	No	94.27	5.79	3592636	461	13194	69.7	90.07	3639	d	Bacteria;p	Proteobacteria;c	Alphaproteobacteria;o	Rhodobacterales;f	Rhodobacteraceae;g	Pararhodobacter;s	Pararhodobacter sp007131945
SRR7083929MAG050	Yes	89.21	2.31	3396004	470	9740	65.7	90.62	3774	d	Bacteria;p	Chloroflexota;c	Dehalococcoidia;o	Tepidiformales;f	Tepidiformaceae;g	SLAK01;s	SLAK01 sp007126855
SRR7083929MAG051	Yes	89.29	0	2241812	127	30590	51.8	93.27	2211	d	Bacteria;p	Bdellovibrionota;c	Bdellovibrionia;o	Bdellovibrionales;f	UBA6776;g	SKLV01;s	SKLV01 sp007121385
SRR7083929MAG052	Yes	100	2.2	4106820	387	18918	56.2	89.08	3356	d	Bacteria;p	Hydrogenedentota;c	Hydrogenedentia;o	Hydrogenedentiales;f	CAITNO01;g	SLFT01;s	SLFT01 sp007118765
SRR7083929MAG053	Yes	93.75	4.15	2864413	294	14121	54	91.59	2686	d	Bacteria;p	Verrucomicrobiota;c	Kiritimatiellae;o	SLAD01;f	SLAD01;g	SLAD01;s	SLAD01 sp007126515
SRR7083929MAG054	No	91.33	5.15	2530262	349	10181	65.9	91.88	2554	d	Bacteria;p	Proteobacteria;c	Gammaproteobacteria;o	Nitrococcales;f	Halorhodospiraceae;g	SKYL01;s	SKYL01 sp007121755
SRR7083929MAG055	No	89.55	2.73	1869204	290	9462	42.8	92.04	1987	d	Bacteria;p	Actinobacteriota;c	Humimicrobiia;o	Humimicrobiales;f	Humimicrobiaceae;g	Halolacustris;s	Halolacustris secundus
SRR7083929MAG056	Yes	92.05	1.14	2363141	363	9400	52.4	92.07	2433	d	Bacteria;p	Spirochaetota;c	Spirochaetia;o	PWKH01;f	PWKH01;g	SKXS01;s	SKXS01 sp007128345
SRR7083929MAG057	Yes	98.9	4.4	4168841	225	74757	66.4	90.66	3855	d	Bacteria;p	Gemmatimonadota;c	Gemmatimonadetes;o	Longimicrobiales;f	UBA6960;g	SKKN01;s	SKKN01 sp007121455
SRR7083929MAG058	Yes	97.8	3.3	3554584	215	48908	66.4	90.45	3304	d	Bacteria;p	Gemmatimonadota;c	Gemmatimonadetes;o	Longimicrobiales;f	UBA6960;g	SLCK01;s	SLCK01 sp007125845
SRR7083929MAG059	No	98.4	3.6	3619619	225	36916	55.6	93.42	3407	d	Bacteria;p	Spirochaetota;c	Spirochaetia;o	DSM-27196;f	SLAA01;g	SLAA01;s	SLAA01 sp007127105
SRR7083929MAG060	Yes	93.22	4.1	3628450	311	19685	64.4	86.21	3489	d	Bacteria;p	Deinococcota;c	Deinococci;o	Deinococcales;f	Trueperaceae;g	;s	
SRR7083929MAG061	No	92.6	5.25	3794330	373	14503	45.5	91.7	3420	d	Bacteria;p	Bacteroidota;c	T3Sed10-11;o	T3Sed10-11;f	T3Sed10-11;g	T3Sed10-11;s	T3Sed10-11 sp007131635
SRR7083929MAG062	Yes	91.29	9.65	7966160	912	11300	42.9	90.27	7418	d	Bacteria;p	Bacteroidota;c	T3Sed10-11;o	T3Sed10-11;f	T3Sed10-11;g	T3Sed10-11;s	T3Sed10-11 sp007128495
SRR7083929MAG063	No	90.53	2.84	2241228	367	8023	57.4	90.11	2211	d	Bacteria;p	Planctomycetota;c	Phycisphaerae;o	Sedimentisphaerales;f	Anaerohalosphaeraceae;g	T3Sed10-213;s	T3Sed10-213 sp007121565
SRR7083929MAG064	Yes	94	7.44	5079311	291	53111	64.8	90.96	4767	d	Bacteria;p	Proteobacteria;c	Gammaproteobacteria;o	Pseudomonadales;f	HTCC2089;g	SLTB01;s	SLTB01 sp007131295
SRR7083929MAG065	No	91.13	3.23	3483165	350	13604	45.8	89.33	2939	d	Bacteria;p	Bacteroidota;c	Bacteroidia;o	Bacteroidales;f	UBA7960;g	PUPG01;s	PUPG01 sp007122505
SRR7083929MAG066	No	93.8	1.48	2018446	99	83522	54.8	87.3	1929	d	Bacteria;p	Firmicutes D;c	Dethiobacteria;o	DTU022;f	UBA8154;g	SKMY01;s	SKMY01 sp003556225
SRR7083929MAG068	Yes	94.08	2.04	3717124	322	16086	61.8	88.47	3270	d	Bacteria;p	Verrucomicrobiota;c	Verrucomicrobiae;o	Verrucomicrobiales;f	SLCJ01;g	SLCJ01;s	SLCJ01 sp007125835
SRR7083929MAG069	No	93.24	2.7	3793279	138	51151	60.6	89.47	3062	d	Bacteria;p	Verrucomicrobiota;c	Verrucomicrobiae;o	Opitutales;f	T3Sed10-336;g	PXDC01;s	PXDC01 sp003558065
SRR7083929MAG070	No	95.2	3.6	3928988	449	14876	65.2	94.52	3896	d	Bacteria;p	Spirochaetota;c	Spirochaetia;o	DSM-27196;f	SLST01;g	SLST01;s	SLST01 sp007118855
SRR7083929MAG071	Yes	98.62	4.67	3586403	164	33057	61.9	89.96	3142	d	Bacteria;p	Chloroflexota;c	Anaerolineae;o	UBA1429;f	UBA1429;g	UBA1429;s	UBA1429 sp007132185
SRR7083929MAG072	Yes	97.48	1.04	3129185	288	17103	38	86.97	3091	d	Bacteria;p	Cyanobacteria;c	Cyanobacteriia;o	Cyanobacteriales;f	Cyanobacteriaceae;g	Cyanobacterium;s	
SRR7083929MAG073	Yes	97.25	4.05	4744074	959	6528	64.2	88.26	4829	d	Bacteria;p	Gemmatimonadota;c	Gemmatimonadetes;o	Longimicrobiales;f	UBA6960;g	SLCK01;s	SLCK01 sp007124095
SRR7083929MAG074	Yes	94.09	3.54	2761332	162	41681	58.8	90.19	2445	d	Bacteria;p	Verrucomicrobiota;c	Kiritimatiellae;o	SLAD01;f	SLAD01;g	SKZA01;s	SKZA01 sp007136425
SRR7083929MAG075	Yes	96.7	5.68	3130790	312	17256	68.7	90.75	3073	d	Bacteria;p	Gemmatimonadota;c	Gemmatimonadetes;o	Longimicrobiales;f	Longimicrobiaceae;g	SKRI01;s	SKRI01 sp007118565
SRR7083929MAG076	No	79.99	1.84	3020209	545	7325	70.7	89.46	3180	d	Bacteria;p	Proteobacteria;c	Alphaproteobacteria;o	Rhodobacterales;f	Rhodobacteraceae;g	SKOX01;s	SKOX01 sp007119835
SRR7083929MAG077	Yes	99.35	2.8	3953903	199	34297	44.5	85.2	3784	d	Bacteria;p	Desulfobacterota;c	Desulfobacteria;o	Desulfobacterales;f	SKZT01;g	SKZT01;s	SKZT01 sp007127235
SRR7083929MAG078	No	95.8	2.51	2858879	245	32258	44	90.25	2919	d	Bacteria;p	Firmicutes A;c	Clostridia;o	Peptostreptococcales;f	T1SED10-28;g	T1SED10-28;s	T1SED10-28 sp003554105
SRR7083929MAG079	Yes	80.08	1.12	838008	39	46581	44.2	86.91	852	d	Bacteria;p	Patescibacteria;c	Paceibacteria;o	UBA9983 A;f	CSBR16-193;g	SKVL01;s	SKVL01 sp007125155
SRR7083929MAG081	Yes	99.93	0	5347791	231	45981	60.9	89.18	4019	d	Bacteria;p	Planctomycetota;c	Planctomycetia;o	Pirellulales;f	Pirellulaceae;g	UBA6163;s	UBA6163 sp007127095
SRR7083929MAG082	Yes	89.07	1.91	3312167	277	19555	42.9	88.14	2987	d	Bacteria;p	Bacteroidota;c	Rhodothermia;o	Balneolales;f	Balneolaceae;g	SKRC01;s	SKRC01 sp007134665
SRR7083929MAG083	Yes	87.67	3.75	2741082	242	16183	55.5	90.81	2527	d	Bacteria;p	Verrucomicrobiota;c	Kiritimatiellae;o	SLAD01;f	SLAD01;g	SLAD01;s	SLAD01 sp007125875
SRR7083929MAG084	No	91.2	2.8	5802858	266	34750	65.2	91.64	4663	d	Bacteria;p	Myxococcota;c	Bradymonadia;o	Bradymonadales;f	Bradymonadaceae;g	SKON01;s	SKON01 sp007120035
SRR7083929MAG086	No	96.8	5.2	4528808	362	30343	64.5	94.7	4310	d	Bacteria;p	Spirochaetota;c	Spirochaetia;o	DSM-27196;f	SLST01;g	SLST01;s	SLST01 sp007127335
SRR7083929MAG087	No	98.78	3.55	6567814	601	18014	62.6	85.18	5384	d	Bacteria;p	Planctomycetota;c	Planctomycetia;o	Pirellulales;f	Pirellulaceae;g	SLMF01;s	SLMF01 sp007124535
SRR7083929MAG088	Yes	94.92	9.4	6395618	888	9966	67.8	85.11	5395	d	Bacteria;p	Planctomycetota;c	Planctomycetia;o	Pirellulales;f	Thermoguttaceae;g	SKOZ01;s	SKOZ01 sp007119795
SRR7083929MAG089	Yes	91.36	6.21	5842882	835	9288	60.3	90.07	5439	d	Bacteria;p	Chloroflexota;c	Anaerolineae;o	B4-G1;f	SLSP01;g	PWVB01;s	PWVB01 sp007125545
SRR7083929MAG090	No	95.16	5.99	2697240	99	51698	53.9	90.98	2223	d	Bacteria;p	Bacteroidota;c	Bacteroidia;o	Bacteroidales;f	UBA7960;g	PWJC01;s	PWJC01 sp003560165
SRR7083929MAG092	Yes	95.36	4.73	6002980	425	24407	56.4	89.63	5333	d	Bacteria;p	Chloroflexota;c	Anaerolineae;o	Promineofilales;f	Promineofilaceae;g	SLGK01;s	SLGK01 sp007123775
SRR7083929MAG093	No	82.48	0.85	3270165	252	22662	68.7	89.43	3181	d	Bacteria;p	Actinobacteriota;c	Actinomycetia;o	Nitriliruptorales;f	Nitriliruptoraceae;g	CSSed11-175R1;s	CSSed11-175R1 sp007136095
SRR7083929MAG095	No	98.81	1.2	3523905	135	51960	59.8	91.16	3496	d	Bacteria;p	Proteobacteria;c	Alphaproteobacteria;o	Rhodobacterales;f	Rhodobacteraceae;g	Roseinatronobacter;s	Roseinatronobacter monicus
SRR7083929MAG096	Yes	95.48	1.29	2345864	122	39241	58.5	87.86	2188	d	Bacteria;p	Desulfobacterota;c	Desulfuromonadia;o	Desulfuromonadales;f	Syntrophotaleaceae;g	SLLR01;s	SLLR01 sp007134025
SRR7083929MAG097	No	94.71	3.63	2690034	162	27628	50.1	89.93	2235	d	Bacteria;p	Bacteroidota;c	Bacteroidia;o	Bacteroidales;f	UBA7960;g	PUPG01;s	PUPG01 sp007125585
SRR7083929MAG098	No	70.21	3.68	1710811	341	6942	49.5	87.87	1852	d	Bacteria;p	Desulfobacterota I;c	Desulfovibrionia;o	Desulfovibrionales;f	Desulfonatronovibrionaceae;g	Desulfonatronovibrio;s	
SRR7083929MAG099	Yes	85.3	2.06	2188073	285	11182	56.4	92.32	2076	d	Bacteria;p	Verrucomicrobiota;c	Kiritimatiellae;o	UBA8416;f	UBA8416;g	T3Sed10-140;s	T3Sed10-140 sp007126175
SRR7083929MAG101	Yes	93.41	3.91	4011487	933	5055	56.3	88.24	3744	d	Bacteria;p	Hydrogenedentota;c	Hydrogenedentia;o	Hydrogenedentiales;f	CAITNO01;g	SLFT01;s	SLFT01 sp007124105
SRR7083929MAG103	No	84.48	8.62	1921644	133	26029	50.7	88.53	1854	d	Bacteria;p	Firmicutes D;c	Dethiobacteria;o	DTU022;f	DTU022;g	B1SED10-74M;s	B1SED10-74M sp003554395
SRR7083929MAG104	No	85.81	5.86	2385362	96	56757	52	89	2221	d	Bacteria;p	Firmicutes D;c	Dethiobacteria;o	DTU022;f	DTU022;g	T3SED10-7;s	T3SED10-7 sp007130585
SRR7083929MAG105	Yes	86.99	4.03	2540991	255	14387	44.9	91.18	2170	d	Bacteria;p	Bacteroidota;c	Bacteroidia;o	Bacteroidales;f	UBA7960;g	SKTA01;s	SKTA01 sp007132825
SRR7083929MAG106	No	73.04	0	865052	104	9700	47.3	93.45	1083	d	Archaea;p	Nanoarchaeota;c	Nanoarchaeia;o	Woesearchaeales;f	SKIA01;g	SKIA01;s	SKIA01 sp007128245
SRR7083929MAG107	No	93.41	1.1	4293615	207	60096	67	90.21	3604	d	Bacteria;p	Gemmatimonadota;c	Gemmatimonadetes;o	Longimicrobiales;f	UBA6960;g	PWLA01;s	PWLA01 sp007121555
SRR7083929MAG108	Yes	92.24	1.98	2100240	72	57230	56.4	85.97	2135	d	Bacteria;p	Chloroflexota;c	Dehalococcoidia;o	GIF9;f	AB-539-J10;g	SKVJ01;s	SKVJ01 sp007129495
SRR7083929MAG109	Yes	88.09	5.97	4068764	289	29397	62	91.83	3724	d	Bacteria;p	Proteobacteria;c	Gammaproteobacteria;o	Xanthomonadales;f	Wenzhouxiangellaceae;g	Wenzhouxiangella;s	Wenzhouxiangella sp007131235

SRR7083929MAG11(	No	89.01	3.52	2687734	452	7670	60.7	90.28	2799	d	Bacteria;p	Proteobacteria;c	Gammaproteobacteria;o	Thiohalomonadales;f	Thiohalomonadaceae;g	SLDE01;s	SLDE01 sp007125455
SRR7083929MAG111	Yes	91.4	4.84	3337412	112	74920	66.8	93.71	3123	d	Bacteria;p	Proteobacteria;c	Gammaproteobacteria;o	PWYM01;f	PWYM01;g	PWYM01;s	PWYM01 sp003567475
SRR7083929MAG112	No	93.41	3.46	3778677	358	20245	67.4	91.74	3306	d	Bacteria;p	Gemmatimonadota;c	Gemmatimonadetes;o	Longimicrobiales;f	UBA6960;g	PWLA01;s	PWLA01 sp003567615
SRR7083929MAG114	Yes	90	5.49	3329177	413	10651	61.3	94.54	3329	d	Bacteria;p	Spirochaetota;c	Spirochaetia;o	DSM-27196;f	PWMO01;g	PWMO01;s	PWMO01 sp007130025
SRR7083929MAG115	Yes	84.49	7.14	2138394	157	25358	48.8	85.48	2037	d	Bacteria;p	Desulfobacterota I;c	Desulfovibrionia;o	Desulfovibrionales;f	Desulfonatronovibrionaceae;g	Desulfonatronospira;s	Desulfonatronospira sp007127015
SRR7083929MAG11(	No	90	3.23	2813040	307	12397	46.8	90.25	2498	d	Bacteria;p	Bacteroidota;c	Bacteroidia;o	Bacteroidales;f	UBA7960;g	SKTA01;s	SKTA01 sp007120085
SRR7083929MAG117	No	92.1	2.51	2477421	176	19115	48.4	91.06	2040	d	Bacteria;p	Bacteroidota;c	Bacteroidia;o	Bacteroidales;f	UBA7960;g	PUPG01;s	PUPG01 sp007124575
SRR7083929MAG118	Yes	75.99	5.17	729635	56	16571	53.8	90.75	772	d	Bacteria;p	Patescibacteria;c	Paceibacteria;o	UBA9983 A;f	J119;g	UBA12088;s	UBA12088 sp007116405
SRR7083929MAG119	No	87.2	3.49	3413797	354	16513	67.9	92.52	3370	d	Bacteria;p	Proteobacteria;c	Gammaproteobacteria;o	PWYM01;f	PWYM01;g	PWYM01;s	PWYM01 sp003567855
SRR7083929MAG12(	No	88.73	0	2322467	435	6796	45	83.48	2169	d	Bacteria;p	Firmicutes F;c	Halanaerobiia;o	Halanaerobiales;f	Halarsenatibacteraceae;g	T1SED10-84;s	T1SED10-84 sp007134885
SRR7083929MAG122	No	94.64	3.13	2572583	162	32131	44.3	89.05	2510	d	Bacteria;p	Firmicutes A;c	Clostridia;o	Peptostreptococcales;f	Tindalliaceae;g	Tindallia;s	Tindallia sp007134225
SRR7083929MAG123	No	77.44	4.52	2990867	646	5625	57.7	88.64	3207	d	Bacteria;p	Bacteroidota;c	Rhodothermia;o	Rhodothermales;f	Salinibacteraceae;g	Tc-Br11-B2g6-7;s	Tc-Br11-B2g6-7 sp007124945
SRR7083929MAG124	Yes	88.97	6.17	4155219	261	42696	65.6	91.69	3910	d	Bacteria;p	Proteobacteria;c	Gammaproteobacteria;o	Pseudomonadales;f	HTCC2089;g	SLTB01;s	SLTB01 sp007122315
SRR7083929MAG13(	No	71.31	0.1	2575071	740	3853	65.7	90.95	2607	d	Bacteria;p	Planctomycetota;c	PUPC01;o	PUPC01;f	PUPC01;g	PUPC01;s	PUPC01 sp003559755
SRR7083929MAG13(	Yes	85.79	6.85	3029683	585	7808	69.6	83.55	3202	d	Archaea;p	Halobacteriota;c	Halobacteria;o	Halobacteriales;f	Haloarculaceae;g	Natronomonas;s	Natronomonas sp007135435
SRR7083929MAG138	Yes	76.16	5.44	3196392	548	7964	67.8	90.83	3076	d	Bacteria;p	Gemmatimonadota;c	Gemmatimonadetes;o	Longimicrobiales;f	UBA6960;g	PWLA01;s	PWLA01 sp007129525
SRR7083929MAG141	No	73.02	2.19	2628654	686	4319	48.9	90.06	2491	d	Bacteria;p	Bacteroidota;c	Rhodothermia;o	Balneolales;f	PXAI01;g	PXAI01;s	PXAI01 sp003567135
SRR7083929MAG142	No	79.62	5.42	3895536	938	4876	68.2	89.66	4477	d	Bacteria;p	Proteobacteria;c	Alphaproteobacteria;o	Geminicoccales;f	Geminicoccaceae;g	SLRI01;s	
SRR7083929MAG143	No	86.68	3.48	2887774	479	8387	68	88.92	3409	d	Archaea;p	Halobacteriota;c	Halobacteria;o	Halobacteriales;f	Halalkalicoccaceae;g	SKXI01;s	SKXI01 sp007129135
SRR7083929MAG144	Yes	76.34	7.17	4298168	570	9886	45	89.77	3522	d	Bacteria;p	Bacteroidota;c	Bacteroidia;o	Bacteroidales;f	UBA7960;g	SKVR01;s	SKVR01 sp007135645
SRR7083929MAG145	No	77.93	7.36	3582466	862	4931	60.3	93.39	3828	d	Bacteria;p	Spirochaetota;c	Spirochaetia;o	DSM-27196;f	Alkalispirochaetaceae;g	Alkalispirochaeta;s	Alkalispirochaeta sp007130865
SRR7083929MAG14(	Yes	77.63	7.34	2308551	677	3607	47.9	86.73	2454	d	Bacteria;p	Desulfobacterota I;c	Desulfovibrionia;o	Desulfovibrionales;f	Desulfonatronovibrionaceae;g	Desulfonatronovibrio;s	Desulfonatronovibrio sp007121655
SRR7083929MAG149	No	73.55	0.02	1617136	473	3723	45.6	92.09	1751	d	Bacteria;p	Bacteroidota;c	Bacteroidia;o	Flavobacteriales;f	Schleiferiaceae;g	CSBr16-58;s	CSBr16-58 sp003566715
SRR7083929MAG15(	Yes	73.18	6.55	2405798	670	4268	43.5	88.42	2702	d	Bacteria;p	Firmicutes A;c	Clostridia;o	Peptostreptococcales;f	T1SED10-28;g	Isachenkonia;s	Isachenkonia sp007118655
SRR7083929MAG151	Yes	94.66	6.01	3755251	373	15654	44.4	87.94	3414	d	Bacteria;p	Bacteroidota;c	Rhodothermia;o	Balneolales;f	Balneolaceae;g	UBA2664;s	UBA2664 sp007127745
SRR7083929MAG152	Yes	79.54	4.34	2369648	672	3881	69.6	93.75	2807	d	Bacteria;p	Actinobacteriota;c	Acidimicrobiia;o	Acidimicrobiales;f	SKKL01;g	SKKL01;s	SKKL01 sp007121465
SRR7083929MAG153	Yes	80.89	2.74	1689686	467	3955	48.2	93.66	1850	d	Bacteria;p	Chlamydiota;c	Chlamydiia;o	Chlamydiales;f	SLFE01;g	SLFE01;s	SLFE01 sp007124415
SRR7083929MAG155	No	89.89	2.01	2422780	567	5783	48.8	90.2	2261	d	Bacteria;p	Bacteroidota;c	Bacteroidia;o	Bacteroidales;f	VadinHA17;g	SLNP01;s	SLNP01 sp007132995
SRR7083929MAG15(	Yes	72.09	4.26	3862840	1289	3284	66.9	90.06	3965	d	Bacteria;p	Verrucomicrobiota;c	Verrucomicrobiae;o	Verrucomicrobiales;f	DEV007;g	Arctic95D-9;s	Arctic95D-9 sp007123695
SRR7083929MAG158	Yes	72.05	1.7	2632723	597	5570	68	90.53	2536	d	Bacteria;p	Planctomycetota;c	Phycisphaerae;o	Phycisphaerales;f	UBA1924;g	SLFH01;s	SLFH01 sp007124345
SRR7083929MAG164	Yes	75.71	2.35	4079092	1138	4020	56.1	89.44	3958	d	Bacteria;p	Planctomycetota;c	Planctomycetia;o	Pirellulales;f	Pirellulaceae;g	Roseimaritima;s	Roseimaritima sp007124075
SRR7083929MAG169	Yes	96.11	3.22	4141876	462	12296	41.2	83.65	3363	d	Bacteria;p	Bacteroidota;c	Bacteroidia;o	Chitinophagales;f	Saprospiraceae;g	SLDF01;s	SLDF01 sp007125435
SRR7083929MAG171	No	75.41	5.44	2674456	326	12427	67.8	94.1	2703	d	Bacteria;p	Proteobacteria;c	Gammaproteobacteria;o	PWYM01;f	PWYM01;g	PWYM01;s	PWYM01 sp007127715
SRR7083929MAG177	Yes	72.75	0.98	1202547	354	3881	40.8	90.62	1410	d	Archaea;p	Halobacteriota;c	Methanosarcinia;o	Methanosarcinales;f	Methanosarcinaceae;g	Methanosalsum;s	Methanosalsum sp007127385
SRR7083929MAG191	No	71.27	2.94	2857901	929	3532	64.7	91.82	2806	d	Bacteria;p	Myxococcota;c	UBA9042;o	PWKZ01;f	PWKZ01;g	PWKZ01;s	PWKZ01 sp003559575
SRR7083929MAG195	Yes	73.04	2.24	2028130	366	6505	55.7	91.9	1803	d	Bacteria;p	Bacteroidota;c	Bacteroidia;o	Bacteroidales;f	UBA7960;g	PUPG01;s	PUPG01 sp007120675
SRR7083929MAG199	Yes	75.15	6.77	1038272	327	3510	36.8	93.52	1234	d	Bacteria;p	Firmicutes;c	Bacilli;o	Izemoplasmatales;f	Izemoplasmataceae;g	CSBR16-104;s	CSBR16-104 sp007116245
SRR7083929MAG204	Yes	88.6	2.58	3673716	291	18870	63.5	86.96	3133	d	Bacteria;p	Myxococcota;c	Polyangia;o	Polyangiales;f	SLQV01;g	SLQV01;s	SLQV01 sp007124745
SRR7083929MAG207	Yes	70.68	5.33	1326389	543	2908	51.4	93.14	1678	d	Bacteria;p	Firmicutes;c	Bacilli;o	Izemoplasmatales;f	Izemoplasmataceae;g	SLQR01;s	SLQR01 sp007132465
SRR7083929MAG217	Yes	88.72	3.4	2014383	238	11600	65.8	94.79	2122	d	Bacteria;p	Proteobacteria;c	Gammaproteobacteria;o	SKWO01;f	SKWO01;g	SKWO01;s	SKWO01 sp007128895
SRR7083929MAG224	Yes	89.51	8.53	3924904	282	19014	67.9	91.26	3560	d	Bacteria;p	Gemmatimonadota;c	Gemmatimonadetes;o	Longimicrobiales;f	UBA6960;g	SLBA01;s	SLBA01 sp007126575
SRR7083929MAG237	No	86.4	2.2	3230095	273	15196	69.7	92.06	2785	d	Bacteria;p	Gemmatimonadota;c	Gemmatimonadetes;o	Longimicrobiales;f	UBA6960;g	SKNW01;s	SKNW01 sp007120305
SRR7083929MAG243	Yes	83.04	8.9	3305204	443	11701	71.8	92.58	3335	d	Bacteria;p	Deinococcota;c	Deinococci;o	Deinococcales;f	Trueperaceae;g	SKWV01;s	SKWV01 sp007128755
SRR7083929MAG244	No	78.36	5.12	3915261	477	10581	55.7	83.65	3812	d	Bacteria;p	Cyanobacteria;c	Cyanobacteriia;o	Phormidesmiales;f	Phormidesmiaceae;g	Nodosilinea;s	Nodosilinea sp003564835
SRR7083929MAG247	No	81.57	1.9	3851464	308	18291	55.5	83.43	3671	d	Bacteria;p	Cyanobacteria;c	Cyanobacteriia;o	Phormidesmiales;f	Phormidesmiaceae;g	Nodosilinea;s	Nodosilinea sp007135385
SRR7083929MAG264	No	87.2	6.4	4289826	386	18963	57.7	94.11	4118	d	Bacteria;p	Spirochaetota;c	Spirochaetia;o	DSM-27196;f	SKKC01;g	SKKC01;s	SKKC01 sp007121695
SRR7083929MAG272	Yes	84.12	5.67	2903188	682	5067	67.6	88.24	3088	d	Bacteria;p	Proteobacteria;c	Alphaproteobacteria;o	Rhodobacterales;f	Rhodobacteraceae;g	Pararhodobacter;s	
SRR7083929MAG275	Yes	94.52	4.42	2495078	150	30819	67.1	92.41	2379	d	Bacteria;p	Proteobacteria;c	Gammaproteobacteria;o	Nitrococcales;f	Halorhodospiraceae;g	Alkalilimnicola;s	Alkalilimnicola mobilis
SRR7083929MAG283	Yes	95.99	6.59	3630769	338	20421	67.9	90.71	3251	d	Bacteria;p	Gemmatimonadota;c	Gemmatimonadetes;o	Longimicrobiales;f	UBA6960;g	T3Sed10-66;s	T3Sed10-66 sp003564555
SRR7083929MAG284	No	89.41	5.49	3149424	137	51806	65	91.02	2658	d	Bacteria;p	Gemmatimonadota;c	Gemmatimonadetes;o	Longimicrobiales;f	UBA6960;g	PWLA01;s	PWLA01 sp003562075
SRR7083929MAG305	No	87.16	4.1	3737989	256	27277	43.2	87	3320	d	Bacteria;p	Bacteroidota;c	Rhodothermia;o	Balneolales;f	Balneolaceae;g	UBA2664;s	UBA2664 sp007123715
SRR7083929MAG311	No	92.15	5.5	2722273	307	12495	44.2	89.47	2539	d	Bacteria;p	Bacteroidota;c	Rhodothermia;o	Balneolales;f	Balneolaceae;g	SW132;s	SW132 sp007129085
SRR7083929MAG328	No	82.93	9.84	3179069	673	7424	54.4	89.19	3060	d	Bacteria;p	Bacteroidota;c	Rhodothermia;o	Balneolales;f	Natronogracilivirgulaceae;g	SKNL01;s	SKNL01 sp007120555
SRR7083929MAG345	No	82.76	4.86	4220864	333	25069	67.5	88.74	3843	d	Bacteria;p	Gemmatimonadota;c	Gemmatimonadetes;o	Longimicrobiales;f	UBA6960;g	PWLA01;s	PWLA01 sp003559555
SRR7083929MAG34(	Yes	93.41	9.02	4214345	476	18766	68.1	90.34	3769	d	Bacteria;p	Gemmatimonadota;c	Gemmatimonadetes;o	Longimicrobiales;f	UBA6960;g	PWLA01;s	PWLA01 sp007132965
SRR7083930MAG001	Yes	98.55	3.39	1470846	43	79116	37.5	92.91	1457	d	Bacteria;p	Firmicutes;c	Bacilli;o	Izemoplasmatales;f	Izemoplasmataceae;g	CSBR16-107;s	CSBR16-107 sp007117835
SRR7083930MAG002	Yes	94.92	3.25	2775078	95	78796	42.8	88.91	2646	d	Bacteria;p	Firmicutes G;c	Limnochordia;o	DTU010;f	DTU012;g	CSBR16-88;s	CSBR16-88 sp007116145
SRR7083930MAG003	No	98.1	1.6	3373462	189	46822	65.5	87.38	3391	d	Bacteria;p	Proteobacteria;c	Gammaproteobacteria;o	Ectothiorhodospirales;f	Thioalkalivibrionaceae;g	Thioalkalivibrio B;s	Thioalkalivibrio B sp007116035
SRR7083930MAG004	Yes	99	1.29	1928326	93	37571	58.8	94.41	1924	d	Bacteria;p	Proteobacteria;c	Gammaproteobacteria;o	Burkholderiales;f	Burkholderiaceae;g	;s	
SRR7083930MAG005	Yes	95.16	1.16	1987794	120	26229	53.1	90.67	1985	d	Bacteria;p	Proteobacteria;c	Alphaproteobacteria;o	Micavibrionales;f	Micavibrionaceae;g	UM-FILTER-47-13;s	UM-FILTER-47-13 sp007117665
SRR7083930MAG00(	Yes	98.29	1.47	2588373	116	111597	65.6	93.36	2502	d	Bacteria;p	Actinobacteriota;c	Acidimicrobiia;o	Acidimicrobiales;f	Ilumatobacteraceae;g	CSBr16-110;s	CSBr16-110 sp007117775
SRR7083930MAG007	Yes	98.95	2.62	2510665	100	73651	56	92.77	2455	d	Bacteria;p	Proteobacteria;c	Gammaproteobacteria;o	Burkholderiales;f	Burkholderiaceae;g	Algicoccus;s	Algicoccus sp007116135
SRR7083930MAG008	No	90.67	0	1337875	77	28870	51.7	92.8	1332	d	Bacteria;p	Firmicutes;c	Bacilli;o	Izemoplasmatales;f	Izemoplasmataceae;g	SLQR01;s	SLQR01 sp007123975
SRR7083930MAG009	No	98.29	1.28	2034488	221	51578	59.8	93.99	2292	d	Bacteria;p	Actinobacteriota;c	Actinomycetia;o	Nitriliruptorales;f	Nitriliruptoraceae;g	SLDB01;s	
SRR7083930MAG01(	Yes	98.84	1.1	4346921	213	45520	45.2	86.24	3782	d	Bacteria;p	Fibrobacterota;c	Chitinivibrionia;o	Chitinivibrionales;f	Chitinispirillaceae;g	Chitinispirillum;s	Chitinispirillum sp007115975
SRR7083930MAG011	Yes	98.54	2.68	2742896	116	39788	35.1	88.91	2558	d	Bacteria;p	Bacteroidota;c	Bacteroidia;o	Flavobacteriales;f	Flavobacteriaceae;g	Psychroflexus;s	Psychroflexus sp007116535
SRR7083930MAG013	No	88.44	1.08	2262299	108	34780	44.7	91.62	2024	d	Bacteria;p	Bacteroidota;c	Bacteroidia;o	Flavobacteriales;f	Schleiferiaceae;g	CSBr16-58;s	CSBr16-58 sp003566715
SRR7083930MAG014	No	94	2.79	1182781	126	21206	40.4	94.42	1262	d	Bacteria;p	Firmicutes;c	Bacilli;o	Izemoplasmatales;f	Izemoplasmataceae;g	CSBR16-87;s	
SRR7083930MAG015	Yes	93.39	1.34	2769523	220	19663	44.6	90.33	2377	d	Bacteria;p	Bacteroidota;c	Bacteroidia;o	Bacteroidales;f	UBA7960;g	SKTA01;s	SKTA01 sp007123065
SRR7083930MAG01(	Yes	87.15	4.19	3850289	407	18166	44.1	89.88	3502	d	Bacteria;p	Bacteroidota;c	Bacteroidia;o	Bacteroidales;f	UBA7960;g	SKYG01;s	SKYG01 sp007128045
SRR7083930MAG017	Yes	90.59	7	3072987	710	5516	54.1	92.4	3295	d	Bacteria;p	Proteobacteria;c	Gammaproteobacteria;o	Enterobacterales;f	Alteromonadaceae;g	Alkalimonas;s	Alkalimonas sp007117525
SRR7083930MAG018	Yes	97.53	0.61	1713319	68	63659	49.7	91.9	1729	d	Bacteria;p	Proteobacteria;c	Gammaproteobacteria;o	Nitrosococcales;f	Methylophagaceae;g	Methylophaga;s	Methylophaga sp007117275
SRR7083930MAG019	Yes	98.49	4.98	4265208	450	23418	65.7	90.17	4270	d	Bacteria;p	Proteobacteria;c	Gammaproteobacteria;o	Pseudomonadales;f	Halomonadaceae;g	Halomonas;s	
SRR7083930MAG02(	Yes	73.13	0	856187	81	15497	39.8	92.71	1071	d	Archaea;p	Nanoarchaeota;c	Nanoarchaeia;o	Woesearchaeales;f	SKIA01;g	SKIA01;s	SKIA01 sp007117065
SRR7083930MAG021	Yes	91.02	6.65	4059804	416	12898	67.3	91.47	4321	d	Bacteria;p	Proteobacteria;c	Alphaproteobacteria;o	Rhodobacterales;f	Rhodobacteraceae;g	Pararhodobacter;s	Pararhodobacter sp007117005
SRR7083930MAG022	Yes	77.57	0.93	846403	37	42972	35.4	92.07	916	d	Archaea;p	Nanoarchaeota;c	Nanoarchaeia;o	Woesearchaeales;f	SKGA01;g	SKGA01;s	SKGA01 sp007117735
SRR7083930MAG023	Yes	94.93	1.95	2589494	87	55605	51.6	91.37	2459	d	Bacteria;p	Proteobacteria;c	Gammaproteobacteria;o	Pseudomonadales;f	Natronospirillaceae;g	SKIB01;s	SKIB01 sp007116635
SRR7083930MAG025	Yes	93.55	6.72	3046415	274	33962	44	91.42	2777	d	Bacteria;p	Bacteroidota;c	Bacteroidia;o	Flavobacteriales;f	Schleiferiaceae;g	CSBr16-58;s	CSBr16-58 sp007116495
SRR7083930MAG02(	Yes	96.89	2.67	1271445	64	35623	44.4	93.59	1271	d	Bacteria;p	Firmicutes;c	Bacilli;o	Izemoplasmatales;f	Izemoplasmataceae;g	CSBR16-87;s	
SRR7083930MAG027	Yes	95.77	7.62	3113203	239	24835	33.5	88.66	3078	d	Bacteria;p	Bacteroidota;c	Bacteroidia;o	Flavobacteriales;f	Flavobacteriaceae;g	Psychroflexus;s	Psychroflexus maritimus
SRR7083930MAG029	No	87.16	4.47	1884827	180	16556	54.9	95.83	1995	d	Bacteria;p	Proteobacteria;c	Gammaproteobacteria;o	Nitrococcales;f	Nitrococcaceae;g	Spiribacter;s	
SRR7083930MAG031	Yes	95.45	1.52	2561229	158	25472	51	92.05	2533	d	Bacteria;p	Spirochaetota;c	Spirochaetia;o	PWKH01;f	PWKH01;g	PWKH01;s	PWKH01 sp007116075
SRR7083930MAG032	Yes	90.53	6.59	4128641	776	6861	57.8	86.47	3773	d	Bacteria;p	Hydrogenedentota;c	Hydrogenedentia;o	Hydrogenedentiales;f	SCBR16-9;g	SCBR16-9;s	SCBR16-9 sp007116095
SRR7083930MAG033	Yes	97.06	7.03	2996200	428	10389	62.8	89.91	3125	d	Bacteria;p	Proteobacteria;c	Gammaproteobacteria;o	Nitrococcales;f	Aquisalimonadaceae;g	SKGQ01;s	SKGQ01 sp007117515
SRR7083930MAG034	Yes	96.28	4.65	5073582	627	14703	52.9	92.54	5553	d	Bacteria;p	Verrucomicrobiota;c	Verrucomicrobiae;o	Opitutales;f	DSM-45221;g	SKFT01;s	SKFT01 sp007117865
SRR7083930MAG035	Yes	80.54	1.12	676852	24	71379	36.3	89.01	719	d	Bacteria;p	Patescibacteria;c	Paceibacteria;o	UBA9983 A;f	CSBR16-193;g	CSBR16-193;s	CSBR16-193 sp007117035
SRR7083930MAG037	Yes	73.6	3.37	1166601	129	19054	27.2	89.49	1432	d	Bacteria;p	Firmicutes;c	Bacilli;o	RF39;f	UBA660;g	SKJH01;s	SKJH01 sp007116005
SRR7083930MAG038	Yes	72.26	1.72	998091	98	15554	34.5	90.38	989	d	Bacteria;p	Patescibacteria;c	JAEDAM01;o	Absconditabacterales;f	X112;g	SKIU01;s	SKIU01 sp007116275
SRR7083930MAG039	Yes	77.1	1.87	928535	82	16225	47.2	93.57	1155	d	Archaea;p	Nanoarchaeota;c	Nanoarchaeia;o	Woesearchaeales;f	SKIA01;g	SKIA01;s	SKIA01 sp007128245
SRR7083930MAG04(	Yes	75.71	0	769534	27	47823	40.7	92.13	714	d	Bacteria;p	Patescibacteria;c	JAEDAM01;o	Absconditabacterales;f	X112;g	;s	

SRR7083930MAG041	Yes	91.47	3.25	2326398	190	20822	54.2	93.55	2318	d	Bacteria;p Proteobacteria;c Gammaproteobacteria;o Pseudomonadales;f Halomonadaceae;g Marinospirillum;s Marinospirillum sp007116865
SRR7083930MAG043	Yes	81.61	1.02	1346097	119	17715	46.5	93.72	1407	d	Bacteria;p Proteobacteria;c Gammaproteobacteria;o Thiomicrospirales;f Thiomicrospiraceae;g Thiomicrospira;s
SRR7083930MAG044	Yes	82.14	1.57	2397421	271	14623	50	92.17	2470	d	Bacteria;p Proteobacteria;c Gammaproteobacteria;o Pseudomonadales;f Halomonadaceae;g Marinospirillum;s Marinospirillum sp007116195
SRR7083930MAG045	Yes	92.65	4.35	3752142	677	7049	40.5	90.81	3706	d	Bacteria;p Bacteroidota;c Bacteroidia;o Bacteroidales;f Prolixibacteraceae;g SKHV01;s SKHV01 sp007116765
SRR7083930MAG047	Yes	89.03	2.44	2193353	280	12102	55	92.63	2321	d	Bacteria;p Proteobacteria;c Gammaproteobacteria;o Burkholderiales;f Burkholderiaceae;g Algicoccus;s
SRR7083930MAG048	Yes	79.44	0.93	1004869	118	12547	44.9	93.42	1246	d	Archaea;p Nanoarchaeota;c Nanoarchaeia;o Woesearchaeales;f SKIA01;g SKIA01;s SKIA01 sp007116645
SRR7083930MAG050	Yes	74.83	2.13	2374044	547	5079	58.1	92.36	2600	d	Bacteria;p Proteobacteria;c Gammaproteobacteria;o Pseudomonadales;f Pseudomonadaceae;g Pseudomonas D;s Pseudomonas D sp007117785
SRR7083930MAG051	No	83.63	6.38	2369629	717	4300	53.9	90.79	2733	d	Bacteria;p Proteobacteria;c Alphaproteobacteria;o Micavibrionales;f UBA2020;g CSBR16-224;s CSBR16-224 sp007116755
SRR7083930MAG054	Yes	87.35	6.38	2295251	300	10645	36.3	88.73	2306	d	Bacteria;p Firmicutes A;c Clostridia;o SK-Y3;f SK-Y3;g SKGK01;s SKGK01 sp007117465
SRR7083930MAG055	Yes	80.46	1.72	1233739	112	15167	51.1	88.52	1312	d	Bacteria;p Patescibacteria;c Paceibacteria;o UBA9983 A;f J119;g CSSED11-315;s CSSED11-315 sp003561715
SRR7083930MAG056	Yes	77.57	3.74	1411812	196	11227	33.9	91.8	1741	d	Archaea;p Nanoarchaeota;c Nanoarchaeia;o Woesearchaeales;f JAFGUC01;g ;s
SRR7083930MAG058	Yes	74.27	2.07	609968	72	11228	37.4	89.22	661	d	Bacteria;p Patescibacteria;c Paceibacteria;o UBA9983 A;f CSBR16-119;g CSBR16-119;s CSBR16-119 sp007117675
SRR7083930MAG059	Yes	72.58	4.46	1828641	659	3439	40.2	94.01	2293	d	Bacteria;p Bdellovibrionota;c Bacteriovoracia;o Bacteriovoracales;f Bacteriovoracaceae;g SKGZ01;s SKGZ01 sp007117175
SRR7083930MAG060	Yes	72.82	1.94	991596	297	3559	37.7	88.84	1261	d	Archaea;p Nanoarchaeota;c Nanoarchaeia;o Woesearchaeales;f JAFGUC01;g ;s
SRR7083930MAG063	Yes	76.17	5.82	1507469	192	14695	25.2	89.67	1774	d	Archaea;p Nanoarchaeota;c Nanoarchaeia;o Woesearchaeales;f UBA583;g UBA583;s
SRR7083930MAG065	Yes	88.88	4.34	1584379	111	22544	56	94.68	1651	d	Bacteria;p Proteobacteria;c Gammaproteobacteria;o Ectothiorhodospirales;f Ectothiorhodospiraceae;g Ectothiorhodosinus;s Ectothiorhodosinus mongolicus
SRR7083930MAG066	Yes	90.6	7.43	3197589	609	6903	41	92.21	3023	d	Bacteria;p Bacteroidota;c Rhodothermia;o Balneolales;f Natronogracilivirgulaceae;g SKIC01;s SKIC01 sp007116685
SRR7083930MAG067	Yes	93.42	1.89	3643358	930	5003	61.1	95.13	4017	d	Bacteria;p Spirochaetota;c Spirochaetia;o DSM-27196;f Alkalispirochaetaceae;g Alkalispirochaeta;s Alkalispirochaeta sp007117185
SRR7083930MAG070	Yes	82.84	4.23	2658284	809	3888	53.9	91.03	3034	d	Bacteria;p Proteobacteria;c Gammaproteobacteria;o Pseudomonadales;f Halomonadaceae;g Halomonas;s Halomonas sp007116835
SRR7083930MAG077	No	89.54	5.59	3497285	370	14793	64.5	91.31	3619	d	Bacteria;p Proteobacteria;c Alphaproteobacteria;o Rhodobacterales;f Rhodobacteraceae;g Roseinatronobacter;s Roseinatronobacter sp003561595
SRR7083930MAG079	Yes	72.2	5.93	1929282	500	4548	36.2	88.75	2151	d	Bacteria;p Firmicutes A;c Clostridia;o SK-Y3;f SK-Y3;g SKGK01;s
SRR7083930MAG080	Yes	78.63	0	709223	74	21184	29.6	92.71	805	d	Bacteria;p Firmicutes;c Bacilli;o RF39;f UBA660;g CSBR16-113R1;s CSBR16-113R1 sp007116525
SRR7083930MAG082	Yes	82.17	5.56	2822800	335	18983	64.1	90.55	2971	d	Bacteria;p Proteobacteria;c Alphaproteobacteria;o Rhodobacterales;f Rhodobacteraceae;g Roseinatronobacter;s Roseinatronobacter sp007116575
SRR7083930MAG085	Yes	77.34	7.48	1030693	215	6110	35.9	92.2	1345	d	Archaea;p Nanoarchaeota;c Nanoarchaeia;o Woesearchaeales;f UBA525;g ;s
SRR7083930MAG087	No	75.53	1.67	1105184	84	20945	55.3	93.13	1169	d	Archaea;p Thermoplasmatota;c Thermoplasmata;o Methanomassiliicoccales;f Methanomethylophilaceae;g PWHV01;s PWHV01 sp003557905
SRR7083930MAG093	Yes	75.59	9.14	1717752	255	17400	57.6	93.32	1916	d	Bacteria;p Actinobacteriota;c Actinomycetia;o Actinomycetales;f Microbacteriaceae;g Pontimonas;s
SRR7083930MAG096	Yes	75.63	5.65	1239707	145	12453	53.1	92.38	1306	d	Archaea;p Thermoplasmatota;c Thermoplasmata;o Methanomassiliicoccales;f Methanomethylophilaceae;g PWHV01;s PWHV01 sp007116915
SRR7083930MAG097	Yes	86.02	9.92	2006275	248	11896	54.8	92.39	2142	d	Archaea;p Thermoplasmatota;c Thermoplasmata;o Methanomassiliicoccales;f Methanomethylophilaceae;g PWHV01;s PWHV01 sp007117455
SRR7083930MAG099	Yes	77.5	3.54	2286528	545	4795	43.2	87.77	2592	d	Bacteria;p Firmicutes G;c Limnochordia;o DTU010;f DTU012;g CSBR16-202;s CSBR16-202 sp007116995
SRR7083930MAG106	No	79.71	3.87	2723493	1209	2469	57.6	94.97	3409	d	Bacteria;p Spirochaetota;c Spirochaetia;o DSM-27196;f SKKC01;g SKKC01;s SKKC01 sp007121695
SRR7083930MAG107	Yes	82.41	5.96	2950467	455	8995	61.4	91.45	3215	d	Bacteria;p Proteobacteria;c Alphaproteobacteria;o Rhodobacterales;f Rhodobacteraceae;g Roseinatronobacter;s
SRR7083930MAG118	Yes	87.07	8.62	3275675	508	10067	33.5	89.14	3564	d	Bacteria;p Bacteroidota;c Bacteroidia;o Flavobacteriales;f Flavobacteriaceae;g Psychroflexus;s
SRR7083930MAG167	Yes	88.89	3.23	2937534	204	27221	43.7	85.54	2691	d	Bacteria;p Proteobacteria;c Gammaproteobacteria;o Enterobacterales;f Vibrionaceae;g Vibrio;s Vibrio sp007124475
SRR7083934MAG001	No	81.12	2.1	2161373	146	47608	42.3	90.05	2193	d	Bacteria;p Firmicutes A;c Clostridia;o Peptostreptococcales;f T1SED10-28;g T1SED10-28;s T1SED10-28 sp003554105
SRR7083934MAG002	No	79.38	2.11	2158976	120	46248	62.9	90.95	2029	d	Bacteria;p Proteobacteria;c Gammaproteobacteria;o SLND01;f SLND01;g SLND01;s SLND01 sp007133205
SRR7083934MAG003	No	97.42	2.47	2910625	146	43707	52.3	89.66	2763	d	Bacteria;p Desulfobacterota;c Desulfuromonadia;o Desulfuromonadales;f Geopsychrobacteraceae;g Pelovirga;s Pelovirga sp007125165
SRR7083934MAG004	No	94.52	1.64	3926276	166	40957	43.9	91.5	3544	d	Bacteria;p Bacteroidota;c T3Sed10-11;o T3Sed10-11;f T3Sed10-11;g T3Sed10-11;s T3Sed10-11 sp003568415
SRR7083934MAG005	Yes	94.49	0.64	2489724	135	36474	54.1	88.91	2485	d	Bacteria;p Firmicutes D;c Dethiobacteria;o PWVN01;f PWVN01;g PWVN01;s PWVN01 sp007136165
SRR7083934MAG006	No	95.6	0.28	2101672	160	22150	41.6	95.05	1927	d	Bacteria;p Delongbacteria;c UBA4055;o UBA4055;f UBA4055;g SLFX01;s SLFX01 sp007124045
SRR7083934MAG007	Yes	92.35	0.94	2852594	222	16920	59.6	89	2565	d	Bacteria;p Proteobacteria;c Gammaproteobacteria;o Chromatiales;f Sedimenticolaceae;g SLIM01;s SLIM01 sp007135625
SRR7083934MAG008	No	95.25	3.49	3140780	278	15548	48.9	90.76	2662	d	Bacteria;p Bacteroidota;c Bacteroidia;o Bacteroidales;f PWNQ01;g SKPS01;s SKPS01 sp007119395
SRR7083934MAG009	No	97.89	1.51	3470399	363	15126	59.3	90.97	3604	d	Bacteria;p Proteobacteria;c Alphaproteobacteria;o Rhodobacterales;f Rhodobacteraceae;g Roseinatronobacter;s Roseinatronobacter sp007125005
SRR7083934MAG010	Yes	94.43	3.48	2695150	321	15044	66	91.52	2663	d	Bacteria;p Proteobacteria;c Gammaproteobacteria;o Nitrococcales;f Halorhodospiraceae;g SKYL01;s SKYL01 sp007121755
SRR7083934MAG011	No	94.52	6.56	5701282	1374	7053	45.3	91.54	5650	d	Bacteria;p Bacteroidota;c T3Sed10-11;o T3Sed10-11;f T3Sed10-11;g T3Sed10-11;s T3Sed10-11 sp007131635
SRR7083934MAG012	No	93.83	1.55	2570173	145	65157	44.6	90.13	2560	d	Bacteria;p Firmicutes F;c Halanaerobiia;o B1SED10-174;f B1SED10-174;g T1SED10-26;s T1SED10-26 sp007122335
SRR7083934MAG013	Yes	93.86	1.48	2240516	65	53455	52.1	89.63	2065	d	Bacteria;p Firmicutes D;c Dethiobacteria;o DTU022;f DTU022;g T3SED10-7;s T3SED10-7 sp007130585
SRR7083934MAG014	No	76.93	0.3	2404463	89	51625	59.6	91.01	2386	d	Bacteria;p Proteobacteria;c Alphaproteobacteria;o Rhodobacterales;f Rhodobacteraceae;g Roseinatronobacter;s Roseinatronobacter monicus
SRR7083934MAG015	Yes	92.8	1.68	2668476	153	34095	38.5	93.72	2397	d	Bacteria;p UBP6;c UBA1177;o UBA1177;f UBA1177;g SLGQ01;s SLGQ01 sp007132725
SRR7083934MAG016	Yes	95.44	2.7	2934368	198	25653	58.1	90.76	2526	d	Bacteria;p Verrucomicrobiota;c Kiritimatiellae;o SLAD01;f SLAD01;g SLAD01;s SLAD01 sp007123265
SRR7083934MAG017	No	90.88	1.64	2834599	174	25566	55.4	91.59	2796	d	Bacteria;p Proteobacteria;c Gammaproteobacteria;o Competibacterales;f Competibacteraceae;g SKOM01;s SKOM01 sp007120095
SRR7083934MAG018	Yes	93.07	4.46	1848061	146	23258	63.1	89.88	1802	d	Bacteria;p Firmicutes E;c SLMV01;o SLMV01;f SLMV01;g CSSED11-154;s CSSED11-154 sp003563985
SRR7083934MAG019	Yes	91.19	9.92	4449790	375	22219	44.3	87.23	3880	d	Bacteria;p Bacteroidota;c Bacteroidia;o Bacteroidales;f PUMT01;g PXAP01;s PXAP01 sp003556985
SRR7083934MAG020	Yes	96.77	3.76	2746432	101	49277	53.8	90.61	2236	d	Bacteria;p Bacteroidota;c Bacteroidia;o Bacteroidales;f UBA7960;g PWJC01;s PWJC01 sp003560165
SRR7083934MAG021	Yes	96.98	3.49	3324189	130	47953	48.5	91.64	2760	d	Bacteria;p Bacteroidota;c Bacteroidia;o Bacteroidales;f PUMT01;g PXAP01;s PXAP01 sp007132715
SRR7083934MAG022	Yes	93.87	4.03	3799433	194	33244	60	90.96	3199	d	Bacteria;p Myxococcota;c Myxococcia;o SLRQ01;f SLRQ01;g SLRQ01;s SLRQ01 sp007130885
SRR7083934MAG023	Yes	89.2	0	1840517	111	27960	50.1	93.8	1659	d	Archaea;p Thermoplasmatota;c Thermoplasmata;o PWKY01;f PWKY01;g PWKY01;s PWKY01 sp007133925
SRR7083934MAG024	Yes	98.33	0.95	2577329	80	53432	48.5	88.85	2083	d	Bacteria;p Bacteroidota;c Bacteroidia;o Bacteroidales;f VadinHA17;g SLNP01;s SLNP01 sp007132995
SRR7083934MAG025	No	92.47	0	2395629	84	49264	50.1	90.08	1966	d	Bacteria;p Bacteroidota;c Bacteroidia;o Bacteroidales;f UBA7960;g PUPG01;s PUPG01 sp007125585
SRR7083934MAG026	No	84.72	1.91	1654061	87	45184	44.9	86.91	1615	d	Bacteria;p Firmicutes D;c Dethiobacteria;o DTU022;f UBA8154;g SLJK01;s SLJK01 sp007135125
SRR7083934MAG027	Yes	94.89	0.81	2620429	184	30215	49.5	91.61	2270	d	Bacteria;p Bacteroidota;c Bacteroidia;o Bacteroidales;f UBA7960;g PYGY01;s PYGY01 sp007129945
SRR7083934MAG028	Yes	92.17	2.55	3330119	214	28247	63.8	85.1	3143	d	Bacteria;p Proteobacteria;c Gammaproteobacteria;o Nitrococcales;f Aquisalimonadaceae;g SLSJ01;s SLSJ01 sp007130525
SRR7083934MAG029	Yes	92.08	1.98	1962187	140	21099	53.9	87.29	1878	d	Bacteria;p Firmicutes E;c SLMV01;o SLMV01;f SLMV01;g SLMV01;s SLMV01 sp007133365
SRR7083934MAG030	Yes	98.78	3.45	6672935	558	18636	62.6	84.94	5491	d	Bacteria;p Planctomycetota;c Planctomycetia;o Pirellulales;f Pirellulaceae;g SLMF01;s SLMF01 sp007124535
SRR7083934MAG031	No	94.51	3.3	4200383	195	78702	66.3	90.07	3842	d	Bacteria;p Gemmatimonadota;c Gemmatimonadetes;o Longimicrobiales;f UBA6960;g SKKN01;s SKKN01 sp007121455
SRR7083934MAG032	Yes	98.39	2.15	2704296	129	46840	44.8	93.5	2281	d	Bacteria;p Bacteroidota;c Bacteroidia;o Bacteroidales;f UBA7960;g SKRM01;s SKRM01 sp007118495
SRR7083934MAG033	Yes	97.6	1.07	3838412	198	45463	60.3	93.47	3583	d	Bacteria;p Spirochaetota;c Spirochaetia;o DSM-27196;f Alkalispirochaetaceae;g Alkalispirochaeta;s Alkalispirochaeta sp007130865
SRR7083934MAG034	Yes	96.5	1.63	2497191	130	40776	44.4	89.37	2429	d	Bacteria;p Firmicutes A;c Clostridia;o Peptostreptococcales;f Tindalliaceae;g Tindallia;s Tindallia sp007134225
SRR7083934MAG035	No	95.6	5.49	3186322	144	39136	68.2	91.66	2776	d	Bacteria;p Gemmatimonadota;c Gemmatimonadetes;o Longimicrobiales;f UBA6960;g SLBA01;s SLBA01 sp007130605
SRR7083934MAG036	Yes	95.2	2.26	3746849	271	21670	57.9	88.13	3481	d	Bacteria;p Bacteroidota;c Rhodothermia;o Rhodothermales;f Salinibacteraceae;g Tc-Br11-B2g6-7;s Tc-Br11-B2g6-7 sp007124945
SRR7083934MAG037	Yes	90.08	3.76	5683124	546	19267	60.6	90.27	4670	d	Bacteria;p Myxococcota;c Bradymonadia;o Bradymonadales;f Bradymonadaceae;g SLJM01;s SLJM01 sp007135085
SRR7083934MAG038	Yes	94.09	9.5	5069978	534	17796	39.1	86.21	4333	d	Bacteria;p Bacteroidota;c Bacteroidia;o Bacteroidales;f UBA7960;g ;s
SRR7083934MAG039	Yes	97.6	3.6	4668665	474	17342	64.8	93.69	4581	d	Bacteria;p Spirochaetota;c Spirochaetia;o DSM-27196;f SLST01;g SLST01;s SLST01 sp007118855
SRR7083934MAG040	No	83.95	9.4	2625359	284	13464	58.5	90.59	2268	d	Bacteria;p Verrucomicrobiota;c Kiritimatiellae;o SLAD01;f SLAD01;g SLKG01;s SLKG01 sp007134735
SRR7083934MAG041	Yes	92.23	2.27	3643900	900	4991	55.3	89.99	3663	d	Bacteria;p Planctomycetota;c Phycisphaerae;o Sedimentisphaerales;f Anaerohalosphaeraceae;g T3Sed10-213;s T3Sed10-213 sp007121565
SRR7083934MAG042	No	97.9	2.1	3122898	111	49902	55	85.78	2807	d	Bacteria;p Desulfobacterota;c Syntrophobacteria;o Syntrophobacterales;f Syntrophobacteraceae;g SLCH01;s SLCH01 sp007125915
SRR7083934MAG043	No	95.7	6	3444971	152	41130	45.8	89.7	2807	d	Bacteria;p Bacteroidota;c Bacteroidia;o Bacteroidales;f UBA7960;g PUML01;s PUML01 sp007129255
SRR7083934MAG044	Yes	98.13	3.37	4232490	364	19399	58.9	89.35	4023	d	Bacteria;p Chloroflexota;c Chloroflexia;o Thermomicrobiales;f Thermomicrobiaceae;g SLMJ01;s SLMJ01 sp007133615
SRR7083934MAG045	Yes	92.52	1.87	3583533	606	10590	58.8	89.68	3598	d	Bacteria;p Chloroflexota;c Chloroflexia;o Thermomicrobiales;f Thermomicrobiaceae;g SLMJ01;s SLMJ01 sp007124395
SRR7083934MAG047	No	90.24	3.41	3706008	213	28881	49	89.41	3046	d	Bacteria;p Bacteroidota;c Bacteroidia;o Bacteroidales;f PUMT01;g PXAP01;s PXAP01 sp007119075
SRR7083934MAG048	Yes	82.18	0	867403	61	18344	42.3	93.14	842	d	Bacteria;p Patescibacteria;c Gracilibacteria;o UBA1369;f LOWO2-01-FULL-3;g SLNK01;s SLNK01 sp007133095
SRR7083934MAG049	No	98.4	1.25	3241365	306	17538	61.3	92.73	3071	d	Bacteria;p Spirochaetota;c Spirochaetia;o DSM-27196;f SLAA01;g B1Sed10-166;s B1Sed10-166 sp007126415
SRR7083934MAG050	No	94.35	2.15	3864623	299	17415	56.2	89.02	3108	d	Bacteria;p Hydrogenedentota;c Hydrogenedentia;o Hydrogenedentiales;f CAITNO01;g SLFT01;s SLFT01 sp007118765
SRR7083934MAG051	No	75.39	0.21	1379575	183	10526	52.9	92.89	1540	d	Bacteria;p Firmicutes D;c Proteinivoracia;o UBA4975;f PWLI01;g PWLI01;s
SRR7083934MAG052	No	87.62	1.98	2164695	381	7317	62.2	90.34	2374	d	Bacteria;p Firmicutes E;c SLMV01;o ;f ;g ;s
SRR7083934MAG053	Yes	85.31	1.68	2604622	555	6007	38.6	94.31	2623	d	Bacteria;p UBP6;c UBA1177;o UBA1177;f UBA1177;g SLGQ01;s SLGQ01 sp007136595
SRR7083934MAG054	No	78.48	2.73	3184596	635	5868	62.2	89.26	2912	d	Bacteria;p Chloroflexota;c Anaerolineae;o B4-G1;f SLSP01;g SLSP01;s SLSP01 sp007130345
SRR7083934MAG055	Yes	95.36	0.82	2676401	126	34253	49.9	90.76	2349	d	Bacteria;p Bacteroidota;c Rhodothermia;o Balneolales;f Balneolaceae;g SLK101;s SLK101 sp007134695
SRR7083934MAG056	Yes	96.4	1.76	10472218	717	27576	60.2	85.87	8687	d	Bacteria;p Planctomycetota;c Planctomycetia;o Pirellulales;f Pirellulaceae;g SKKK01;s SKKK01 sp007121535
SRR7083934MAG058	No	93.07	8.04	2827005	220	21966	58.8	90.24	2540	d	Bacteria;p Verrucomicrobiota;c Kiritimatiellae;o SLAD01;f SLAD01;g SKZA01;s SKZA01 sp007136425
SRR7083934MAG059	Yes	94.06	2.38	2815621	243	19002	55.8	86.75	2817	d	Bacteria;p Firmicutes E;c DTU015;o UBA8153;f UBA8153;g SLSY01;s SLSY01 sp007130145

SRR7083934MAG060	Yes	97.66	0.44	2494446	314	11941	44.9	82.75	2269	d	Bacteria;p	Firmicutes F;c Halanaerobiia;o Halanaerobiales;f Halarsenatibacteraceae;g T1SED10-84;s T1SED10-84 sp007134885
SRR7083934MAG061	Yes	94.97	0.54	1664831	207	10263	49	91.64	1504	d	Bacteria;p	Omnitrophota;c Koll11;o SKK-01;f SKK-01;g SLID01;s SLID01 sp007135805
SRR7083934MAG062	No	83.29	2.08	1663550	362	5069	53.5	91.23	1830	d	Bacteria;p	Proteobacteria;c Alphaproteobacteria;o Micavibrionales;f Micavibrionaceae;g UM-FILTER-47-13;s UM-FILTER-47-13 sp007117665
SRR7083934MAG063	No	86.31	2.81	2308914	631	4675	51.3	95.51	2150	d	Bacteria;p	CG03;c SLGR01;o SLGR01;f SLGR01;g SLGR01;s SLGR01 sp007136585
SRR7083934MAG065	Yes	85.71	5.24	2316413	103	40943	43.3	90.11	1992	d	Bacteria;p	Bacteroidota;c Bacteroidia;o Bacteroidales;f PUMT01;g PUMT01;s PUMT01 sp007125955
SRR7083934MAG066	No	82.42	2.71	2033196	217	25706	50.8	87.88	1951	d	Bacteria;p	Firmicutes D;c Dethiobacteria;o DTU022;f DTU022;g B1SED10-74M;s B1SED10-74M sp003554395
SRR7083934MAG067	No	96.29	0.66	2948729	223	17395	38.1	87.01	2863	d	Bacteria;p	Cyanobacteria;c Cyanobacteriia;o Cyanobacteriales;f Cyanobacteriaceae;g Cyanobacterium;s
SRR7083934MAG068	No	92.4	4.27	4538977	425	14805	56	87.47	3806	d	Bacteria;p	Hydrogenedentota;c Hydrogenedentia;o Hydrogenedentiales;f CAITNO01;g SLFT01;s SLFT01 sp007124105
SRR7083934MAG069	Yes	74.07	8.47	2154234	441	11467	61	90.5	2297	d	Bacteria;p	Proteobacteria;c Gammaproteobacteria;o UBA5335;f UBA5335;g SLRW01;s SLRW01 sp007130745
SRR7083934MAG070	Yes	98.67	1.89	1509538	40	99590	46.9	92.58	1492	d	Bacteria;p	Firmicutes;c Bacilli;o Izemoplasmatales;f Izemoplasmataceae;g B1SED10-225;s B1SED10-225 sp007135105
SRR7083934MAG072	Yes	94.49	3.26	2130536	205	14849	44.4	83.57	2069	d	Bacteria;p	SLNR01;c SLNR01;o SLNR01;f SLNR01;g SLNR01;s SLNR01 sp007132905
SRR7083934MAG073	Yes	97.3	0.61	3653584	233	30346	61.8	88.92	3199	d	Bacteria;p	Verrucomicrobiota;c Verrucomicrobiae;o Opitutales;f Verruco-01;g SLNJ01;s SLNJ01 sp007133065
SRR7083934MAG074	Yes	97.3	8.33	4773924	259	45497	60.2	88.62	3953	d	Bacteria;p	Verrucomicrobiota;c Verrucomicrobiae;o Opitutales;f T3Sed10-336;g PXDC01;s PXDC01 sp003558065
SRR7083934MAG075	No	88.33	2.68	3386774	202	34250	68	92.85	3215	d	Bacteria;p	Proteobacteria;c Gammaproteobacteria;o PWYM01;f PWYM01;g PWYM01;s PWYM01 sp003567855
SRR7083934MAG076	Yes	91.39	2.46	3467977	232	26041	47.3	87.02	3087	d	Bacteria;p	Bacteroidota;c Rhodothermia;o Balneolales;f Balneolaceae;g QGGB01;s QGGB01 sp003561515
SRR7083934MAG077	No	83.05	3.25	2237107	172	22699	44.9	87.53	2119	d	Bacteria;p	Firmicutes D;c Dethiobacteria;o DTU022;f DTU022;g B1SED10-74M;s B1SED10-74M sp007128625
SRR7083934MAG078	No	84.23	1.88	2686206	310	12014	44.9	90.95	2355	d	Bacteria;p	Bacteroidota;c Bacteroidia;o Bacteroidales;f UBA7960;g SKTA01;s SKTA01 sp007132825
SRR7083934MAG079	Yes	81.71	9.81	2521237	425	8833	60.7	90.6	2550	d	Bacteria;p	Proteobacteria;c Gammaproteobacteria;o UBA5335;f UBA5335;g SLRW01;s
SRR7083934MAG080	No	77.72	3.45	495250	117	5131	35.7	91.6	576	d	Bacteria;p	Patescibacteria;c Paceibacteria;o Paceibacterales;f PWPS01;g PWHW01;s PWHW01 sp003561025
SRR7083934MAG081	Yes	91.69	3.85	5223190	219	43892	62.8	87.35	4247	d	Bacteria;p	Latescibacterota;c UBA2968;o UBA8231;f UBA8231;g SLHA01;s SLHA01 sp007136405
SRR7083934MAG082	Yes	85.84	2.33	2529472	455	7201	32.1	90.38	2155	d	Bacteria;p	Bacteroidota;c Bacteroidia;o Bacteroidales;f ML635J-15;g SLSH01;s SLSH01 sp007130555
SRR7083934MAG083	No	92.2	8.65	3501186	200	30602	62	89.81	3111	d	Bacteria;p	Chloroflexota;c Anaerolineae;o UBA1429;f UBA1429;g UBA1429;s UBA1429 sp007132185
SRR7083934MAG084	Yes	100	6.8	4506069	334	28513	64.4	94.57	4278	d	Bacteria;p	Spirochaetota;c Spirochaetia;o DSM-27196;f SLST01;g SLST01;s SLST01 sp007127335
SRR7083934MAG085	Yes	98.37	7.63	4383683	377	27465	61	91.91	4171	d	Bacteria;p	Spirochaetota;c Spirochaetia;o DSM-27196;f SKKC01;g SLAN01;s SLAN01 sp007135605
SRR7083934MAG086	Yes	98.9	5.49	4556234	385	29938	67.2	89.95	3926	d	Bacteria;p	Gemmatimonadota;c Gemmatimonadetes;o Longimicrobiales;f UBA6960;g PWLA01;s PWLA01 sp007121775
SRR7083934MAG087	Yes	95.6	2.2	5203611	581	12883	60.2	87.47	4462	d	Bacteria;p	Hydrogenedentota;c Hydrogenedentia;o Hydrogenedentiales;f SLHB01;g UBA2224;s UBA2224 sp007130675
SRR7083934MAG089	Yes	95.01	5.89	3924812	430	13473	67	85.11	4383	d	Archaea;p	Halobacteriota;c Halobacteria;o Halobacteriales;f Halalkalicoccaceae;g SKXI01;s SKXI01 sp007128515
SRR7083934MAG090	No	89.41	3.21	3379942	119	117855	66.7	93.61	3166	d	Bacteria;p	Proteobacteria;c Gammaproteobacteria;o PWYM01;f PWYM01;g PWYM01;s PWYM01 sp003567475
SRR7083934MAG091	No	94.19	2.15	2429559	170	24143	58.3	88.22	2289	d	Bacteria;p	Desulfobacterota;c Desulfuromonadia;o Desulfuromonadales;f Syntrophotaleaceae;g SLLR01;s SLLR01 sp007134025
SRR7083934MAG092	Yes	94.4	1.06	2771067	180	21167	60.5	90.14	2662	d	Bacteria;p	Proteobacteria;c Gammaproteobacteria;o Thiohalomonadales;f Thiohalomonadaceae;g SLDE01;s SLDE01 sp007125455
SRR7083934MAG093	No	92.21	2.08	2678148	313	13537	69.5	90.96	3051	d	Bacteria;p	Cyanobacteria;c Cyanobacteriia;o PCC-6307;f Cyanobiaceae;g NIES-981;s NIES-981 sp007135755
SRR7083934MAG094	No	84.54	4.65	3992984	378	13609	70	88.3	4120	d	Bacteria;p	Proteobacteria;c Alphaproteobacteria;o Rhodobacterales;f Rhodobacteraceae;g SKOX01;s SKOX01 sp007119835
SRR7083934MAG095	No	89.32	7.26	3042980	340	14615	65.9	92.33	2959	d	Bacteria;p	Proteobacteria;c Gammaproteobacteria;o Xanthomonadales;f Wenzhouxiangellaceae;g Wenzhouxiangella;s Wenzhouxiangella sp007121485
SRR7083934MAG096	Yes	79.33	6.22	6071940	1011	10333	60.8	88.84	5183	d	Bacteria;p	Myxococcota;c Bradymonadia;o Bradymonadales;f Bradymonadaceae;g SLJM01;s SLJM01 sp007131755
SRR7083934MAG097	No	90.23	4.82	3503388	488	9586	62.1	88.82	3167	d	Bacteria;p	Verrucomicrobiota;c Verrucomicrobiae;o Verrucomicrobiales;f SLCJ01;g SLCJ01;s SLCJ01 sp007125835
SRR7083934MAG098	Yes	94.71	8.02	2952807	627	7239	60	90.2	3037	d	Bacteria;p	Planctomycetota;c DG-23;o SLPQ01;f SLPQ01;g SLPQ01;s SLPQ01 sp007131925
SRR7083934MAG099	Yes	89.71	2.47	1632461	173	14702	54.4	93.28	1844	d	Archaea;p	Halobacteriota;c Methanomicrobia;o Methanomicrobiales;f Methanocorpusculaceae;g Methanocalculus;s Methanocalculus sp007130455
SRR7083934MAG100	Yes	85.94	5.72	5984796	704	12440	51.6	90.86	5070	d	Bacteria;p	Myxococcota;c Polyangia;o HGW-17;f SLPX01;g SLPX01;s SLPX01 sp007131745
SRR7083934MAG101	Yes	89.32	3.2	1611836	214	9436	44.1	93.81	1730	d	Archaea;p	Thermoplasmatota;c Thermoplasmata;o PWKY01;f PWKY01;g SKVC01;s
SRR7083934MAG102	No	86.2	1.69	1797796	55	82307	53.5	86.82	1711	d	Bacteria;p	Firmicutes D;c Dethiobacteria;o DTU022;f UBA8154;g SKMY01;s SKMY01 sp003556225
SRR7083934MAG103	No	91.21	7.14	4152613	633	9411	65.8	89.44	4001	d	Bacteria;p	Gemmatimonadota;c Gemmatimonadetes;o Longimicrobiales;f UBA6960;g PXFI01;s PXFI01 sp003564295
SRR7083934MAG104	No	83.74	7.34	2023529	353	7848	65.2	91.29	2105	d	Bacteria;p	PUNC01;c PUNC01;o PUNC01;f PWXJ01;g PWXJ01;s PWXJ01 sp003561965
SRR7083934MAG105	No	98.37	4.85	3417956	402	12097	55.7	93.6	3297	d	Bacteria;p	Spirochaetota;c Spirochaetia;o DSM-27196;f SLAA01;g SLAA01;s SLAA01 sp007127105
SRR7083934MAG106	No	95.2	6.26	4396180	1065	6893	52.4	87.45	4487	d	Bacteria;p	Cyanobacteria;c Cyanobacteriia;o Cyanobacteriales;f Geitlerinemaceae;g Phormidium A;s Phormidium A sp007126595
SRR7083934MAG107	Yes	90.83	5.3	4267276	945	5669	62.9	90.76	4228	d	Bacteria;p	Chloroflexota;c Anaerolineae;o UBA1429;f UBA1429;g SKLG01;s SKLG01 sp007123335
SRR7083934MAG108	Yes	96.96	5.47	4647816	180	56743	70.5	88.27	4342	d	Bacteria;p	Actinobacteriota;c Actinomycetia;o Nitriliruptorales;f Nitriliruptoraceae;g PWLR01;s PWLR01 sp003558435
SRR7083934MAG109	Yes	97.81	1.53	4152886	176	41442	71.5	90.68	4049	d	Bacteria;p	Actinobacteriota;c Actinomycetia;o Mycobacteriales;f Micromonosporaceae;g SLSW01;s SLSW01 sp007130245
SRR7083934MAG110	Yes	87.08	3.06	2300745	145	29245	50.3	88.82	2146	d	Bacteria;p	Firmicutes D;c Dethiobacteria;o DTU022;f DTU022;g SLPH01;s SLPH01 sp007132095
SRR7083934MAG111	Yes	76.17	0	973259	94	13143	40.8	92.72	1168	d	Archaea;p	Nanoarchaeota;c Nanoarchaeia;o Woesearchaeales;f 21-14-0-10-32-9;g PWWA01;s PWWA01 sp007131205
SRR7083934MAG113	Yes	93.39	2.45	3046684	361	12078	67.8	88.83	3494	d	Archaea;p	Halobacteriota;c Halobacteria;o Halobacteriales;f Halalkalicoccaceae;g SKXI01;s SKXI01 sp007129135
SRR7083934MAG114	Yes	95.83	3.14	3344275	133	74712	66.1	91.64	3219	d	Bacteria;p	Proteobacteria;c Gammaproteobacteria;o Nitrococcales;f Aquisalimonadaceae;g Aquisalimonas;s Aquisalimonas sp007126095
SRR7083934MAG115	Yes	83.46	7.4	3730613	782	5761	62.9	85.45	3489	d	Bacteria;p	Planctomycetota;c Phycisphaerae;o UBA1845;f PWPN01;g PWPN01;s PWPN01 sp003557125
SRR7083934MAG116	Yes	92.42	2.04	3373962	424	11313	65.3	90.84	2932	d	Bacteria;p	Planctomycetota;c PUPC01;o PUPC01;f PUPC01;g PUPC01;s PUPC01 sp003559755
SRR7083934MAG117	Yes	91.02	6.67	3099097	406	9751	54.3	87.22	2906	d	Bacteria;p	Desulfobacterota;c Desulfobacteria;o Desulfobacterales;f SURF-3;g B1SED10-16;s
SRR7083934MAG118	No	87.92	5.83	4282154	254	63720	64.9	91.52	4049	d	Bacteria;p	Proteobacteria;c Gammaproteobacteria;o Pseudomonadales;f HTCC2089;g SLTB01;s SLTB01 sp007131295
SRR7083934MAG119	No	75.66	1.77	2227204	957	2553	46.6	85.05	2641	d	Bacteria;p	Firmicutes;c Bacilli;o Bacillales H;f Salisediminibacteriaceae;g Alkalicoccus;s Alkalicoccus sp003560495
SRR7083934MAG126	No	72.08	2.37	2315635	94	52191	65.3	91.21	2247	d	Bacteria;p	Proteobacteria;c Alphaproteobacteria;o Rhodobacterales;f Rhodobacteraceae;g Roseinatronobacter;s Roseinatronobacter sp007134805
SRR7083934MAG127	No	77.87	5.52	3577866	568	7852	64.7	91.7	2986	d	Bacteria;p	Myxococcota;c UBA9042;o PWKZ01;f PWKZ01;g PWKZ01;s PWKZ01 sp003559575
SRR7083934MAG129	Yes	82.38	8.53	4288268	292	29648	44.9	88.59	3489	d	Bacteria;p	Bacteroidota;c Bacteroidia;o Bacteroidales;f PUMT01;g PXAP01;s PXAP01 sp007123885
SRR7083934MAG132	No	76.07	0.41	4655623	1163	4834	65.3	92.45	4490	d	Bacteria;p	Myxococcota;c Bradymonadia;o Bradymonadales;f Bradymonadaceae;g SKON01;s SKON01 sp007120035
SRR7083934MAG133	Yes	95.46	4.67	4013947	763	7436	60.7	94.17	4153	d	Bacteria;p	Spirochaetota;c Spirochaetia;o DSM-27196;f PXBQ01;g PXBQ01;s PXBQ01 sp003566875
SRR7083934MAG135	No	77.74	1.51	2076148	494	5010	52.6	92.52	2271	d	Bacteria;p	Spirochaetota;c Spirochaetia;o PWKH01;f PWKH01;g SKXS01;s SKXS01 sp007128345
SRR7083934MAG137	No	88.63	8.79	4329959	857	6237	62.3	87.9	4335	d	Bacteria;p	Verrucomicrobiota;c Kiritimatiellae;o SLOV01;f SLOV01;g SLOV01;s SLOV01 sp007132345
SRR7083934MAG143	No	80.22	3.3	3558722	252	28100	68.3	90.56	3109	d	Bacteria;p	Gemmatimonadota;c Gemmatimonadetes;o Longimicrobiales;f UBA6960;g PWLA01;s PWLA01 sp003559555
SRR7083934MAG144	Yes	81.6	3.64	2484402	185	22962	63.4	91.8	2324	d	Bacteria;p	Proteobacteria;c Gammaproteobacteria;o Xanthomonadales;f Wenzhouxiangellaceae;g Wenzhouxiangella;s Wenzhouxiangella sp007123055
SRR7083934MAG145	No	86.89	0.85	2417481	421	7456	74.4	91.08	2535	d	Bacteria;p	Actinobacteriota;c Actinomycetia;o Euzebyales;f Egibacteraceae;g T1Sed10-49;s T1Sed10-49 sp003553795
SRR7083934MAG147	Yes	71.34	8.57	3584385	1807	2148	41.9	91.57	4330	d	Archaea;p	Asgardarchaeota;c Lokiarchaeia;o CR-4;f MK-D1;g ;s
SRR7083934MAG148	Yes	71.53	7.5	2172382	426	6034	43.1	89.44	2213	d	Bacteria;p	Bacteroidota;c Rhodothermia;o Balneolales;f Balneolaceae;g UBA2664;s
SRR7083934MAG151	No	79.19	1.47	2507343	690	4014	64.7	91.73	2823	d	Bacteria;p	Proteobacteria;c Gammaproteobacteria;o Chromatiales;f Chromatiaceae;g Halochromatium;s Halochromatium sp003562815
SRR7083934MAG153	Yes	71.14	4.47	2415245	239	20929	64.3	89.45	2474	d	Bacteria;p	Proteobacteria;c Gammaproteobacteria;o Ectothiorhodospirales;f Thioalkalivibrionaceae;g Thioalkalivibrio B;s Thioalkalivibrio B sp007133115
SRR7083934MAG154	No	84.04	5.89	2965064	604	7307	54.2	89.06	2899	d	Bacteria;p	Bacteroidota;c Rhodothermia;o Balneolales;f Natronogracilivirgulaceae;g SKNL01;s SKNL01 sp007120555
SRR7083934MAG156	No	72.53	5.49	3755874	349	19319	67.5	91.63	3264	d	Bacteria;p	Gemmatimonadota;c Gemmatimonadetes;o Longimicrobiales;f UBA6960;g PWLA01;s PWLA01 sp003567615
SRR7083934MAG157	No	78.19	6.31	3238881	493	7495	61.2	92.89	3274	d	Bacteria;p	Spirochaetota;c Spirochaetia;o DSM-27196;f PWMO01;g PWMO01;s PWMO01 sp007130025
SRR7083934MAG159	No	71.59	5.52	4264205	1904	2423	56.1	89.8	4803	d	Bacteria;p	Planctomycetota;c Planctomycetia;o Pirellulales;f Pirellulaceae;g Roseimaritima;s Roseimaritima sp007124075
SRR7083934MAG161	Yes	85.66	2.63	2206193	441	6286	51.6	90.33	2281	d	Bacteria;p	Verrucomicrobiota;c Kiritimatiellae;o SLAD01;f SLAD01;g SLRP01;s SLRP01 sp007130845
SRR7083934MAG164	No	89.25	6.11	4885318	806	9720	55.8	83.79	4911	d	Bacteria;p	Cyanobacteria;c Cyanobacteriia;o Phormidesmiales;f Phormidesmiaceae;g Nodosilinea;s Nodosilinea sp003564835
SRR7083934MAG167	Yes	83.26	5.57	3400873	342	12121	60.6	87.07	3034	d	Bacteria;p	Myxococcota;c Polyangia;o Polyangiales;f SLQV01;g SLQV01;s SLQV01 sp007131285
SRR7083934MAG168	No	79.01	0	1388737	242	6796	39.9	94.01	1385	d	Bacteria;p	Caldisericota;c Caldisericia;o UBA6126;f UBA6126;g T3SED10-260;s T3SED10-260 sp003566815
SRR7083934MAG170	No	81.57	2.16	2333414	144	33142	46.2	88.63	2245	d	Bacteria;p	Firmicutes D;c Dethiobacteria;o DTU022;f DTU022;g T3SED10-7;s T3SED10-7 sp007127255
SRR7083934MAG171	No	72.09	3.09	2472383	728	3769	69.8	83.16	2833	d	Archaea;p	Halobacteriota;c Halobacteria;o Halobacteriales;f Haloarculaceae;g Natronomonas;s Natronomonas sp007135435
SRR7083934MAG180	No	84.09	6	2823877	296	14054	69.6	91.79	3015	d	Bacteria;p	Proteobacteria;c Alphaproteobacteria;o Rhodobacterales;f Rhodobacteraceae;g Rhodobaculum;s Rhodobaculum sp007131685
SRR7083934MAG182	No	91.68	4.1	2524649	420	8425	36.3	90.66	2589	d	Bacteria;p	Firmicutes F;c Halanaerobiia;o Halanaerobiales;f Halarsenatibacteraceae;g SLSL01;s SLSL01 sp007130465
SRR7083934MAG183	No	81.57	9.65	3137969	605	5763	46	87.65	2852	d	Bacteria;p	Bacteroidota;c Bacteroidia;o Chitinophagales;f Saprospiraceae;g PWJY01;s PWJY01 sp007121335
SRR7083934MAG184	Yes	85.2	5.83	3526588	1044	3976	63.8	88.48	3444	d	Bacteria;p	Planctomycetota;c UBA11346;o UBA11346;f UBA11346;g SLQH01;s SLQH01 sp007131555
SRR7083934MAG186	Yes	87.5	5.36	5918602	1104	6753	62.3	87.43	5161	d	Bacteria;p	Planctomycetota;c Planctomycetia;o Pirellulales;f Pirellulaceae;g SLMF01;s SLMF01 sp007133715
SRR7083934MAG188	No	84.98	8.22	4065249	1270	3952	68.4	90.3	4842	d	Bacteria;p	Proteobacteria;c Alphaproteobacteria;o Geminicoccales;f Geminicoccaceae;g SLRI01;s
SRR7083934MAG192	No	78.74	2.74	3989490	1499	3062	61.3	90.82	3973	d	Bacteria;p	Planctomycetota;c Planctomycetia;o Pirellulales;f Pirellulaceae;g UBA6163;s UBA6163 sp007127095
SRR7083934MAG193	Yes	73.27	9.53	3640254	534	8979	60.2	83.98	4039	d	Archaea;p	Halobacteriota;c Halobacteria;o Halobacteriales;f Natrialbaceae;g Natronorubrum;s
SRR7083934MAG194	Yes	93.78	5.3	3883277	421	13512	65.4	89.46	3867	d	Bacteria;p	Proteobacteria;c Alphaproteobacteria;o Rhizobiales;f Beijerinckiaceae;g Salinarimonas;s
SRR7083934MAG202	Yes	85.56	8.2	5531289	857	10259	68.3	90.42	5870	d	Bacteria;p	Proteobacteria;c Alphaproteobacteria;o Geminicoccales;f Geminicoccaceae;g SLRI01;s SLRI01 sp007131045

SRR7083934MAG204	Yes	92.95	7.99	5584641	631	17093	70.8	86.84	5573	d	Bacteria;p	Actinobacteriota;c	Actinomycetia;o	Nitriliruptorales;f	Nitriliruptoraceae;g	SKJO01;s	SKJO01 sp007134165
SRR7083934MAG205	No	88.07	4.52	3294837	289	17918	69.8	90.35	3243	d	Bacteria;p	Proteobacteria;c	Alphaproteobacteria;o	Rhodobacterales;f	Rhodobacteraceae;g	Pararhodobacter;s	Pararhodobacter sp007131945
SRR7083934MAG210	Yes	75.79	2.83	2722003	678	4641	67	89.7	3084	d	Bacteria;p	Proteobacteria;c	Alphaproteobacteria;o	Rhodobacterales;f	Rhodobacteraceae;g	Rhodobaculum;s	Rhodobaculum sp003558415
SRR7083934MAG216	No	86.11	8.44	3288852	296	20895	66.6	91.07	3215	d	Bacteria;p	Proteobacteria;c	Gammaproteobacteria;o	XJ16;f	Halofilaceae;g	SLKC01;s	
SRR7083934MAG219	Yes	72.79	8.79	3491011	735	5389	63.7	88.43	3338	d	Bacteria;p	Hydrogenedentota;c	Hydrogenedentia;o	Hydrogenedentiales;f	SLHB01;g	SLHB01;s	SLHB01 sp007136375
SRR7083934MAG226	Yes	78.42	0.58	1661013	388	5166	66.9	92.74	1924	d	Bacteria;p	Actinobacteriota;c	Coriobacteriia;o	OPB41;f	SLCP01;g	SLCP01;s	SLCP01 sp007125735
SRR7083934MAG229	Yes	74.17	5.28	1526936	442	3775	53.1	92.76	1924	d	Archaea;p	Halobacteriota;c	Methanomicrobia;o	Methanomicrobiales;f	Methanocorpusculaceae;g	Methanocalculus;s	
SRR7083934MAG242	No	78.06	6.14	2574084	314	16303	65.6	87.71	2693	d	Bacteria;p	Proteobacteria;c	Gammaproteobacteria;o	Ectothiorhodospirales;f	Thioalkalivibrionaceae;g	Thioalkalivibrio B;s	Thioalkalivibrio B sp007116035
SRR7083934MAG249	Yes	74.45	7.74	1935108	518	3969	49.4	87.48	2068	d	Bacteria;p	Firmicutes D;c	Dethiobacteria;o	DTU022;f	UBA8154;g	UBA8154;s	
SRR7083934MAG257	Yes	72.57	8.88	3171118	705	5000	48.8	85.26	3215	d	Bacteria;p	Desulfobacterota;c	Desulfobacteria;o	Desulfobacterales;f	SURF-3;g	B1SED10-16;s	B1SED10-16 sp007135795
SRR7083934MAG259	Yes	94.54	6.83	5270817	425	23151	43.4	86.57	4688	d	Bacteria;p	Bacteroidota;c	Rhodothermia;o	Balneolales;f	Balneolaceae;g	UBA2664;s	UBA2664 sp007123715
SRR7083934MAG274	No	76.07	0.85	2893132	195	23113	69.8	90.89	2829	d	Bacteria;p	Actinobacteriota;c	Actinomycetia;o	Nitriliruptorales;f	Nitriliruptoraceae;g	CSSed11-175R1;s	CSSed11-175R1 sp007136095
SRR7083934MAG282	Yes	82.79	4.92	3477665	204	37181	55.4	89.83	2839	d	Bacteria;p	Bacteroidota;c	Rhodothermia;o	Balneolales;f	Natronogracilivirgulaceae;g	SKNL01;s	SKNL01 sp007132975
SRR7083934MAG283	Yes	92.62	3.28	3235293	191	30064	44	90.11	2886	d	Bacteria;p	Bacteroidota;c	Rhodothermia;o	Balneolales;f	Balneolaceae;g	UBA2664;s	UBA2664 sp007134085
SRR7083934MAG292	No	73.9	2.42	1989463	199	14438	47.5	90.95	1691	d	Bacteria;p	Bacteroidota;c	Bacteroidia;o	Bacteroidales;f	UBA7960;g	SKTA01;s	SKTA01 sp007120085
SRR7083934MAG297	Yes	86.5	4.31	3241226	1148	3273	48.6	85.09	3567	d	Bacteria;p	Proteobacteria;c	Gammaproteobacteria;o	Methylococcales;f	Methylomonadaceae;g	Methylotuvimicrobium;s	Methylotuvimicrobium sp007135635
SRR7083934MAG310	Yes	70.3	2.15	1980669	417	5780	45.9	91.27	1833	d	Bacteria;p	Bacteroidota;c	Bacteroidia;o	Bacteroidales;f	UBA7960;g	PUML01;s	PUML01 sp007131125
SRR7083934MAG316	No	92.2	2.53	2388311	133	35888	67.1	92.29	2272	d	Bacteria;p	Proteobacteria;c	Gammaproteobacteria;o	Nitrococcales;f	Halorhodospiraceae;g	Alkalilimnicola;s	Alkalilimnicola mobilis
SRR7083934MAG318	Yes	72.75	2.2	2935099	929	3652	58.8	91.93	3083	d	Bacteria;p	Fibrobacterota;c	Chitinivibrionia;o	Chitinivibrionales;f	;g	;s	
SRR7083934MAG323	No	84.64	3.45	3044895	221	23990	65.6	93.14	2857	d	Bacteria;p	Proteobacteria;c	Gammaproteobacteria;o	Xanthomonadales;f	Wenzhouxiangellaceae;g	Wenzhouxiangella;s	Wenzhouxiangella sp003560975
SRR7083934MAG325	No	72.43	7.26	2979521	348	14157	72.3	89.9	2927	d	Bacteria;p	Actinobacteriota;c	Actinomycetia;o	Nitriliruptorales;f	Nitriliruptoraceae;g	T1Sed10-7;s	T1Sed10-7 sp003561535
SRR7083934MAG339	Yes	82.7	1.87	2292681	427	6800	62	92.96	2348	d	Bacteria;p	Proteobacteria;c	Gammaproteobacteria;o	Xanthomonadales;f	Wenzhouxiangellaceae;g	SLIZ01;s	SLIZ01 sp007135395
SRR7083934MAG364	Yes	84.62	4.27	3568589	256	25663	73	89.78	3377	d	Bacteria;p	Actinobacteriota;c	Actinomycetia;o	Nitriliruptorales;f	Nitriliruptoraceae;g	SLMP01;s	SLMP01 sp007133465
SRR7083934MAG380	No	77.76	3.4	2761283	764	4065	66.7	91.32	3073	d	Bacteria;p	Proteobacteria;c	Gammaproteobacteria;o	Pseudomonadales;f	Halomonadaceae;g	Halomonas;s	Halomonas sp003553625
SRR7901803MAG001	Yes	88.07	0.42	2493966	135	42468	49.5	91.39	2408	d	Bacteria;p	Bdellovibrionota;c	UBA2394;o	UBA2394;f	UBA2394;g	REDG01;s	REDG01 sp007693605
SRR7901803MAG002	Yes	97.85	1.22	4172282	223	36159	49.2	90.04	3668	d	Bacteria;p	Bacteroidota;c	Bacteroidia;o	Flavobacteriales;f	Cryomorphaceae;g	T3Sed10-241;s	
SRR7901803MAG003	Yes	98.67	2.67	1892223	125	25419	49	91.64	1793	d	Bacteria;p	Firmicutes;c	Bacilli;o	Izemoplasmatales;f	Izemoplasmataceae;g	SKGI01;s	SKGI01 sp007694145
SRR7901803MAG004	Yes	96.16	0.5	3684221	273	23385	38.3	87.3	3209	d	Bacteria;p	Bacteroidota;c	Bacteroidia;o	Chitinophagales;f	Saprospiraceae;g	PWJY01;s	PWJY01 sp007693915
SRR7901803MAG005	Yes	97.73	0	3506409	88	61482	65.8	87.83	2978	d	Bacteria;p	Planctomycetota;c	Phycisphaerae;o	Phycisphaerales;f	UBA1924;g	RECQ01;s	RECQ01 sp007693965
SRR7901803MAG006	Yes	91.75	2.61	2503279	46	87818	53.4	91.01	2412	d	Bacteria;p	Proteobacteria;c	Alphaproteobacteria;o	Micavibrionales;f	UBA2020;g	CSBR16-224;s	CSBR16-224 sp007116755
SRR7901803MAG007	Yes	97.85	0.81	3237958	144	41899	49.7	93.62	2810	d	Bacteria;p	Bacteroidota;c	Bacteroidia;o	Flavobacteriales;f	UA16;g	M30B41;s	
SRR7901803MAG008	Yes	97.33	0.44	1472255	72	38344	49.4	93.33	1403	d	Bacteria;p	Firmicutes;c	Bacilli;o	Acholeplasmatales;f	Acholeplasmataceae;g	RECG01;s	RECG01 sp007692425
SRR7901803MAG009	Yes	91.8	4.32	4764196	408	21037	43.6	86.7	4242	d	Bacteria;p	Bacteroidota;c	Rhodothermia;o	Balneolales;f	Balneolaceae;g	UBA2664;s	UBA2664 sp007694095
SRR7901803MAG010	Yes	93.22	2.57	4188414	441	14625	50.8	88.18	3614	d	Bacteria;p	Bacteroidota;c	Rhodothermia;o	Balneolales;f	PXAI01;g	RECM01;s	RECM01 sp007694035
SRR7901803MAG011	Yes	99.34	0.76	3440035	142	35510	38.1	90.8	3409	d	Bacteria;p	Cyanobacteria;c	Cyanobacteriia;o	Cyanobacteriales;f	Gloeocapsaceae;g	Gloeocapsa;s	Gloeocapsa sp007693955
SRR7901803MAG012	Yes	95.94	4.46	3711432	207	30400	42.2	86.68	2984	d	Bacteria;p	Bacteroidota;c	Bacteroidia;o	Chitinophagales;f	Saprospiraceae;g	PWJY01;s	PWJY01 sp007695145
SRR7901803MAG013	No	92.73	0	3576059	441	14856	59	92.2	3252	d	Bacteria;p	Chloroflexota;c	Anaerolineae;o	Aggregatilineales;f	A4b;g	J038;s	J038 sp007693385
SRR7901803MAG014	Yes	87.5	1.7	3017918	64	107489	69.2	88.32	2454	d	Bacteria;p	Planctomycetota;c	Phycisphaerae;o	Phycisphaerales;f	UBA1924;g	RECY01;s	RECY01 sp007693785
SRR7901803MAG015	Yes	88.16	4.43	2649041	477	6658	44.1	92.2	2819	d	Bacteria;p	Proteobacteria;c	Gammaproteobacteria;o	Pseudomonadales;f	Nitrincolaceae;g	Nitrincola;s	Nitrincola sp007693685
SRR7901803MAG016	Yes	98.9	5.49	3488388	84	79875	70.3	90.83	2876	d	Bacteria;p	Gemmatimonadota;c	Gemmatimonadetes;o	Longimicrobiales;f	UBA6960;g	SKNW01;s	
SRR7901803MAG017	Yes	100	3.6	3774783	118	67699	61.1	92.18	3472	d	Bacteria;p	Spirochaetota;c	Spirochaetia;o	DSM-27196;f	PWMO01;g	PWMO01;s	PWMO01 sp007693945
SRR7901803MAG018	Yes	93.6	6.27	4402036	811	7776	64.5	94.21	4635	d	Bacteria;p	Spirochaetota;c	Spirochaetia;o	DSM-27196;f	SLST01;g	SLST01;s	SLST01 sp007694025
SRR7901803MAG019	Yes	96.63	1.93	2778170	321	11756	63.9	91.83	2803	d	Bacteria;p	Proteobacteria;c	Gammaproteobacteria;o	Ectothiorhodospirales;f	Ectothiorhodospiraceae;g	Ectothiorhodospira;s	Ectothiorhodospira sp001632845
SRR7901803MAG020	Yes	93.33	0	1368468	226	7298	43.9	93.1	1507	d	Bacteria;p	Firmicutes;c	Bacilli;o	Izemoplasmatales;f	Izemoplasmataceae;g	CSBR16-87;s	
SRR7901803MAG021	No	91.79	3.21	3918401	731	6807	63.1	88.69	4271	d	Bacteria;p	Proteobacteria;c	Gammaproteobacteria;o	Competibacterales;f	Competibacteraceae;g	REEE01;s	REEE01 sp007695245
SRR7901803MAG023	Yes	97.44	1.24	4444977	195	40864	67	84.22	3891	d	Bacteria;p	Proteobacteria;c	Gammaproteobacteria;o	Chromatiales;f	Chromatiaceae;g	Halochromatium;s	Halochromatium sp007693535
SRR7901803MAG024	No	91.99	1.76	5150903	930	9242	56.3	87.85	4544	d	Bacteria;p	Planctomycetota;c	Planctomycetia;o	Pirellulales;f	Pirellulaceae;g	Roseimaritima;s	
SRR7901803MAG025	Yes	84.67	1.33	1282385	332	4753	36.4	93.28	1471	d	Bacteria;p	Firmicutes;c	Bacilli;o	Izemoplasmatales;f	Izemoplasmataceae;g	CSBR16-104;s	
SRR7901803MAG026	Yes	96.51	2.6	2699640	238	17606	43.1	91.04	2478	d	Bacteria;p	Bacteroidota;c	Bacteroidia;o	Flavobacteriales;f	Schleiferiaceae;g	REDE01;s	REDE01 sp007693635
SRR7901803MAG027	No	97.33	1.63	3909188	285	27482	60.9	93.8	3774	d	Bacteria;p	Spirochaetota;c	Spirochaetia;o	DSM-27196;f	Alkalispirochaetaceae;g	Alkalispirochaeta;s	Alkalispirochaeta sp007695175
SRR7901803MAG029	Yes	93.17	5.48	3200052	406	12248	42.4	89.66	3007	d	Bacteria;p	Bacteroidota;c	Rhodothermia;o	Balneolales;f	Balneolaceae;g	UBA2664;s	
SRR7901803MAG030	No	92.03	0.61	2359191	427	7007	45.6	89.81	2604	d	Bacteria;p	Firmicutes;c	Bacilli;o	Bacillales H;f	Salisediminibacteriaceae;g	Salisediminibacterium;s	
SRR7901803MAG031	Yes	96.5	2.86	4511757	351	20650	68.3	89.99	4339	d	Bacteria;p	Proteobacteria;c	Alphaproteobacteria;o	Geminicoccales;f	Geminicoccaceae;g	RECO01;s	RECO01 sp007694015
SRR7901803MAG032	No	82.25	2.08	3480492	527	9763	55.6	83.83	3494	d	Bacteria;p	Cyanobacteria;c	Cyanobacteriia;o	Phormidesmiales;f	Phormidesmiaceae;g	Nodosilinea;s	Nodosilinea sp007135385
SRR7901803MAG033	Yes	94.02	2.72	4041430	561	9667	59.1	85.04	4009	d	Bacteria;p	Cyanobacteria;c	Cyanobacteriia;o	Phormidesmiales;f	Phormidesmiaceae;g	Nodosilinea;s	
SRR7901803MAG034	Yes	93.8	1.45	4433477	311	20997	56.5	83.43	4049	d	Bacteria;p	Cyanobacteria;c	Cyanobacteriia;o	Phormidesmiales;f	Phormidesmiaceae;g	PCC-6406;s	
SRR7901803MAG035	Yes	86.85	2.03	3343330	663	6019	53.5	89.08	3448	d	Bacteria;p	Cyanobacteria;c	Cyanobacteriia;o	Cyanobacteriales;f	Spirulinaceae;g	Spirulina;s	Spirulina sp007693875
SRR7901803MAG036	Yes	92.2	0	2794523	265	15644	69.7	88.76	2506	d	Bacteria;p	Planctomycetota;c	Phycisphaerae;o	Phycisphaerales;f	UBA1924;g	RECZ01;s	RECZ01 sp007693765
SRR7901803MAG037	Yes	98.75	0.53	5637723	231	43087	62.3	86.81	4181	d	Bacteria;p	Planctomycetota;c	Planctomycetia;o	Pirellulales;f	Pirellulaceae;g	UBA6163;s	UBA6163 sp007694185
SRR7901803MAG039	Yes	90.49	0.18	1602382	589	3405	42.2	92.7	2006	d	Bacteria;p	Proteobacteria;c	Alphaproteobacteria;o	;f	;g	;s	
SRR7901803MAG040	Yes	89.76	7.26	4841931	357	23840	58.6	83.56	4487	d	Bacteria;p	Cyanobacteria;c	Cyanobacteriia;o	Phormidesmiales;f	Phormidesmiaceae;g	Nodosilinea;s	Nodosilinea sp007694115
SRR7901803MAG041	Yes	81.01	2.81	2172575	856	2835	55.8	94.55	2758	d	Bacteria;p	Proteobacteria;c	Gammaproteobacteria;o	Chromatiales;f	Chromatiaceae;g	Thiorhodovibrio B;s	
SRR7901803MAG043	Yes	99.17	4.83	4077749	341	21761	57.2	93.93	3955	d	Bacteria;p	Spirochaetota;c	Spirochaetia;o	DSM-27196;f	SKKC01;g	SKKC01;s	SKKC01 sp007694005
SRR7901803MAG044	Yes	94.49	2.83	4928224	454	15468	53.8	82.11	4529	d	Bacteria;p	Cyanobacteria;c	Cyanobacteriia;o	Elainellales;f	Elainellaceae;g	RECH01;s	RECH01 sp007694125
SRR7901803MAG045	Yes	90.34	5.35	3479098	461	11876	61.3	87.84	3323	d	Bacteria;p	Planctomycetota;c	Phycisphaerae;o	Phycisphaerales;f	SM1A02;g	SKZB01;s	SKZB01 sp007693845
SRR7901803MAG046	Yes	85.42	6.89	2754614	524	6768	64.6	90.83	2741	d	Bacteria;p	Planctomycetota;c	Phycisphaerae;o	Phycisphaerales;f	UBA1924;g	REDD01;s	REDD01 sp007693725
SRR7901803MAG047	Yes	95.68	3.93	3729969	543	10605	71.4	90.78	3989	d	Bacteria;p	Proteobacteria;c	Alphaproteobacteria;o	Rhodobacterales;f	Rhodobacteraceae;g	Rubrimonas;s	Rubrimonas sp007693835
SRR7901803MAG048	Yes	72.23	2.3	5982443	1912	3670	59.1	88.2	5809	d	Bacteria;p	Planctomycetota;c	Planctomycetia;o	Pirellulales;f	Pirellulaceae;g	VGZC01;s	
SRR7901803MAG049	No	95.05	9.89	4061484	768	7337	67.6	90.65	3871	d	Bacteria;p	Gemmatimonadota;c	Gemmatimonadetes;o	Longimicrobiales;f	UBA6960;g	PWLA01;s	PWLA01 sp007695195
SRR7901803MAG050	Yes	76.87	2.34	1100022	151	10206	55.9	92.72	1308	d	Archaea;p	Nanoarchaeota;c	Nanoarchaeia;o	Woesearchaeales;f	WAPJ01;g	;s	
SRR7901803MAG051	No	78.1	3.86	2381055	804	3413	35.5	91.61	2557	d	Bacteria;p	Bacteroidota;c	Bacteroidia;o	Chitinophagales;f	REEL01;g	REEL01;s	REEL01 sp007695095
SRR7901803MAG054	Yes	87.34	1.35	5475964	1356	5073	57.1	90.27	5104	d	Bacteria;p	Planctomycetota;c	Planctomycetia;o	Pirellulales;f	Pirellulaceae;g	UBA6163;s	
SRR7901803MAG055	Yes	78.33	1.77	2249593	324	10128	49	94.09	2350	d	Bacteria;p	Bdellovibrionota;c	UBA2394;o	UBA2394;f	UBA2394;g	;s	
SRR7901803MAG058	Yes	75.59	3.64	2690172	498	6880	60.5	91	2633	d	Bacteria;p	Planctomycetota;c	Phycisphaerae;o	Phycisphaerales;f	UBA1924;g	;s	
SRR7901803MAG059	No	85.1	7.46	4897207	867	6894	56	84.62	4796	d	Bacteria;p	Cyanobacteria;c	Cyanobacteriia;o	Phormidesmiales;f	Phormidesmiaceae;g	PXDR01;s	
SRR7901803MAG060	Yes	74.24	3.89	2466092	666	4324	33.6	91.15	2591	d	Bacteria;p	Bacteroidota;c	Bacteroidia;o	Chitinophagales;f	UBA8649;g	;s	
SRR7901803MAG063	Yes	73.3	2.33	2980000	697	4660	47.1	90.63	2905	d	Bacteria;p	Bacteroidota;c	Bacteroidia;o	Bacteroidales;f	4484-276;g	;s	
SRR7901803MAG066	Yes	73.53	3.94	2617915	970	3105	64.1	91.19	3178	d	Bacteria;p	Proteobacteria;c	Gammaproteobacteria;o	Chromatiales;f	Chromatiaceae;g	Halochromatium;s	
SRR7901803MAG067	No	79.25	0.33	2529536	620	4690	46.3	81.58	2741	d	Bacteria;p	Firmicutes;c	Bacilli;o	Bacillales H;f	Salisediminibacteriaceae;g	Alkalicoccus;s	Alkalicoccus sp007692495
SRR7901803MAG075	Yes	81.4	3.34	2641253	173	22955	69.3	91.27	2608	d	Bacteria;p	Proteobacteria;c	Alphaproteobacteria;o	Rhodobacterales;f	Rhodobacteraceae;g	Rubrimonas;s	
SRR7901803MAG078	Yes	80.72	2.51	2466908	769	3687	49.9	92.21	2885	d	Bacteria;p	Proteobacteria;c	Gammaproteobacteria;o	Pseudomonadales;f	Nitrincolaceae;g	Nitrincola;s	
SRR7901803MAG080	Yes	94.41	7.97	3531433	422	12028	54.6	89.6	3087	d	Bacteria;p	Bacteroidota;c	Rhodothermia;o	Balneolales;f	Natronogracilivirgulaceae;g	SKNL01;s	SKNL01 sp007693825
SRR7901803MAG085	Yes	96.45	2.28	3030650	178	44151	47.7	89.37	2642	d	Bacteria;p	Bacteroidota;c	Rhodothermia;o	Balneolales;f	Balneolaceae;g	UBA2664;s	UBA2664 sp007693745
SRR7901803MAG089	Yes	90.25	5.3	4300233	417	18877	71	91.22	4317	d	Bacteria;p	Deinococcota;c	Deinococci;o	Deinococcales;f	Trueperaceae;g	CSSed10-48;s	CSSed10-48 sp007695125
SRR7901803MAG090	Yes	77.53	2.39	4801324	1449	3987	65	92.4	4983	d	Bacteria;p	Myxococcota;c	Bradymonadia;o	Bradymonadales;f	Bradymonadaceae;g	SKON01;s	
SRR7901803MAG100	Yes	88.26	7.91	4173587	917	5598	64.8	86.34	4727	d	Bacteria;p	Proteobacteria;c	Gammaproteobacteria;o	Ectothiorhodospirales;f	Thioalkalivibrionaceae;g	Thioalkalivibrio B;s	
SRR7901803MAG107	Yes	82.07	4.56	2483191	1133	2420	59.4	89.09	3094	d	Bacteria;p	Desulfobacterota I;c	Desulfovibrionia;o	Desulfovibrionales;f	Desulfonatronaceae;g	Desulfonatronum;s	
SRR7901803MAG111	Yes	92.85	5.5	3719918	512	10567	72.2	87.72	3550	d	Bacteria;p	Proteobacteria;c	Alphaproteobacteria;o	Rhodobacterales;f	Rhodobacteraceae;g	Rhodobaculum;s	
SRR7901803MAG112	No	87.05	1.82	3861878	587	9800	38.9	89.17	3814	d	Bacteria;p	Bacteroidota;c	Bacteroidia;o	Cytophagales;f	Cyclobacteriaceae;g	Mongoliibacter;s	Mongoliibacter sp007692705
SRR7901803MAG122	Yes	74.39	7.6	2899238	1189	2768	42.8	91.74	3461	d	Bacteria;p	Spirochaetota;c	Spirochaetia;o	DSM-27196;f	Salinispiraceae;g	SKVS01;s	

SRR7901803MAG124	Yes	74.72	1.42	2729627	1296	2293	48.6	87.26	3311	d	Bacteria;p	Proteobacteria;c	Gammaproteobacteria;o	Methylococcales;f	Methylomonadaceae;g	Methylotuvimicrobium;s	
SRR7901803MAG143	Yes	81.42	6.27	4488323	1172	4691	69.5	92.24	5147	d	Bacteria;p	Proteobacteria;c	Alphaproteobacteria;o	Geminicoccales;f	Geminicoccaceae;g	RECO01;s	
SRR7901804MAG001	No	95.92	2.31	3614379	282	19431	38.4	87.37	3181	d	Bacteria;p	Bacteroidota;c	Bacteroidia;o	Chitinophagales;f	Saprospiraceae;g	PWJY01;s	PWJY01 sp007693915
SRR7901804MAG002	Yes	97.32	0.99	3428396	93	74121	45.5	87.27	2781	d	Bacteria;p	Bacteroidota;c	Bacteroidia;o	Chitinophagales;f	Saprospiraceae;g	PWJY01;s	PWJY01 sp007695035
SRR7901804MAG003	Yes	95.36	8.47	4053532	367	15368	43.2	84.46	3694	d	Bacteria;p	Bacteroidota;c	Rhodothermia;o	Balneolales;f	Balneolaceae;g	UBA2664;s	
SRR7901804MAG004	Yes	90	1.94	4753295	208	40332	69.8	90.55	3558	d	Bacteria;p	Myxococcota;c	Bradymonadia;o	REDH01;f	REDH01;g	REDH01;s	REDH01 sp007693585
SRR7901804MAG005	No	86.59	1.19	3535339	139	56981	57.6	87.86	3264	d	Bacteria;p	Desulfobacterota I;c	Desulfovibrionia;o	Desulfovibrionales;f	Desulfonatronaceae;g	Desulfonatronum;s	Desulfonatronum sp007693505
SRR7901804MAG006	Yes	98.18	0.91	3356616	133	44272	58.7	92.34	2893	d	Bacteria;p	Chloroflexota;c	Anaerolineae;o	Aggregatilineales;f	A4b;g	J038;s	J038 sp007693385
SRR7901804MAG007	No	94.54	1.7	3970339	256	26776	67.2	84.19	3588	d	Bacteria;p	Proteobacteria;c	Gammaproteobacteria;o	Chromatiales;f	Chromatiaceae;g	Halochromatium;s	Halochromatium sp007693535
SRR7901804MAG008	No	93.37	2.96	4091249	296	24439	40.3	89.1	3404	d	Bacteria;p	Bacteroidota;c	Bacteroidia;o	Bacteroidales;f	UBA7960;g	SKVR01;s	SKVR01 sp007693555
SRR7901804MAG009	Yes	91.94	1.96	4946905	96	112570	67.3	92.02	4138	d	Bacteria;p	Myxococcota A;c	UBA796;o	UBA796;f	GCA-2862545;g	REDI01;s	REDI01 sp007693595
SRR7901804MAG010	Yes	91.69	5.91	3917677	331	17751	53.8	87.94	3457	d	Bacteria;p	Bacteroidota;c	Bacteroidia;o	Flavobacteriales;f	Cryomorphaceae;g	T3Sed10-241;s	
SRR7901804MAG011	Yes	98.21	2.87	3722394	260	27375	49.2	92.22	3286	d	Bacteria;p	Bacteroidota;c	Bacteroidia;o	Flavobacteriales;f	Cryomorphaceae;g	SKUL01;s	SKUL01 sp007695365
SRR7901804MAG012	No	88.78	0.59	2740821	287	13228	69.2	91.41	2747	d	Bacteria;p	Proteobacteria;c	Alphaproteobacteria;o	Rhodobacterales;f	Rhodobacteraceae;g	PUOA01;s	
SRR7901804MAG013	Yes	94.62	3.23	3417766	403	12968	55.8	88.5	2983	d	Bacteria;p	Planctomycetota;c	UBA11346;o	UBA11346;f	UBA11346;g	REDP01;s	REDP01 sp007693455
SRR7901804MAG014	No	93.66	2.22	4551408	230	30332	55.4	83.74	4220	d	Bacteria;p	Cyanobacteria;c	Cyanobacteriia;o	Phormidesmiales;f	Phormidesmiaceae;g	Nodosilinea;s	Nodosilinea sp007135385
SRR7901804MAG015	No	94.51	4.4	3696952	436	16638	67.7	91.12	3305	d	Bacteria;p	Gemmatimonadota;c	Gemmatimonadetes;o	Longimicrobiales;f	UBA6960;g	PWLA01;s	PWLA01 sp007695195
SRR7901804MAG016	No	95.95	2.8	3057923	321	12727	54.7	93.53	3026	d	Bacteria;p	Spirochaetota;c	Spirochaetia;o	DSM-27196;f	SLAA01;g	SLAA01;s	SLAA01 sp007695055
SRR7901804MAG017	Yes	90.91	7.95	3831084	476	11482	64.2	84.59	3391	d	Bacteria;p	Planctomycetota;c	Phycisphaerae;o	Phycisphaerales;f	SM1A02;g	SKZB01;s	
SRR7901804MAG018	No	86.93	7.39	3419620	337	14834	61.3	87.48	3121	d	Bacteria;p	Planctomycetota;c	Phycisphaerae;o	Phycisphaerales;f	SM1A02;g	SKZB01;s	SKZB01 sp007693845
SRR7901804MAG019	Yes	99.15	4.2	3763234	194	54204	69.2	93.51	3652	d	Bacteria;p	Actinobacteriota;c	Acidimicrobiia;o	Acidimicrobiales;f	Ilumatobacteraceae;g	SKSP01;s	SKSP01 sp007693335
SRR7901804MAG020	Yes	97.77	4.68	3561066	211	41703	62.6	90.16	3589	d	Bacteria;p	Proteobacteria;c	Alphaproteobacteria;o	Rhodobacterales;f	Rhodobacteraceae;g	Roseinatronobacter;s	Roseinatronobacter sp007694945
SRR7901804MAG021	No	99.2	5.63	4540768	441	18413	60.4	93.12	4423	d	Bacteria;p	Spirochaetota;c	Spirochaetia;o	DSM-27196;f	PXBQ01;g	PXBQ01;s	PXBQ01 sp007695285
SRR7901804MAG022	Yes	89.15	4.99	3520090	353	15100	53.6	89.92	3135	d	Bacteria;p	Bacteroidota;c	Bacteroidia;o	Flavobacteriales;f	Cryomorphaceae;g	T3Sed10-241;s	
SRR7901804MAG023	No	86.75	1.17	2651740	113	51411	67.2	92.9	2521	d	Bacteria;p	Proteobacteria;c	Gammaproteobacteria;o	GCA-2729495;f	GCA-2729495;g	;s	
SRR7901804MAG024	Yes	96.76	4.27	3410755	228	26978	72	89.07	3238	d	Bacteria;p	Actinobacteriota;c	Actinomycetia;o	Nitriliruptorales;f	Nitriliruptoraceae;g	T1Sed10-7;s	T1Sed10-7 sp003561535
SRR7901804MAG025	Yes	97.25	3.42	3999094	460	13442	69.7	91.99	4013	d	Bacteria;p	Actinobacteriota;c	Acidimicrobiia;o	Acidimicrobiales;f	Ilumatobacteraceae;g	;s	
SRR7901804MAG026	No	90.2	0.59	4301513	452	13226	65.2	90.56	3659	d	Bacteria;p	Verrucomicrobiota;c	Verrucomicrobiae;o	Opitutales;f	Opitutaceae;g	REEB01;s	REEB01 sp007695295
SRR7901804MAG027	Yes	98.75	1.18	5611736	265	33709	64.7	89.41	4204	d	Bacteria;p	Planctomycetota;c	Planctomycetia;o	Pirellulales;f	Pirellulaceae;g	;s	
SRR7901804MAG028	Yes	82.42	4.4	3153993	359	16805	66.1	89.94	2966	d	Bacteria;p	Gemmatimonadota;c	Gemmatimonadetes;o	Longimicrobiales;f	UBA6960;g	PWLA01;s	
SRR7901804MAG029	Yes	96.53	1.2	2922935	213	25806	61.3	94.39	2759	d	Bacteria;p	Spirochaetota;c	Spirochaetia;o	DSM-27196;f	PWMO01;g	PWMO01;s	PWMO01 sp007693485
SRR7901804MAG030	Yes	99.2	3.2	3491149	191	37111	60	93.72	3325	d	Bacteria;p	Spirochaetota;c	Spirochaetia;o	DSM-27196;f	PWMO01;g	PWMO01;s	PWMO01 sp007693325
SRR7901804MAG031	Yes	76.61	4.57	2616305	421	7499	46.3	90.23	2243	d	Bacteria;p	Bacteroidota;c	Bacteroidia;o	Bacteroidales;f	UBA7960;g	SKTA01;s	
SRR7901804MAG032	No	86.56	4.75	2984006	190	25431	46.2	89.65	2420	d	Bacteria;p	Bacteroidota;c	Bacteroidia;o	Bacteroidales;f	UBA7960;g	PUPG01;s	PUPG01 sp007695305
SRR7901804MAG033	Yes	94.72	9.95	3595241	162	36616	66.7	89.99	3433	d	Bacteria;p	Proteobacteria;c	Alphaproteobacteria;o	Rhizobiales;f	Beijerinckiaceae;g	Salinarimonas;s	Salinarimonas sp007693435
SRR7901804MAG034	Yes	95.33	1.87	3675263	603	8624	58.6	89.3	3757	d	Bacteria;p	Chloroflexota;c	Chloroflexia;o	Thermomicrobiales;f	Thermomicrobiaceae;g	SLMJ01;s	SLMJ01 sp007695115
SRR7901804MAG035	Yes	85.26	6.58	2845564	783	4217	35.5	89.47	3058	d	Bacteria;p	Bacteroidota;c	Bacteroidia;o	Chitinophagales;f	REEL01;g	REEL01;s	REEL01 sp007695095
SRR7901804MAG036	No	96.26	9.66	3004300	287	15952	67.6	87.24	2910	d	Bacteria;p	Proteobacteria;c	Gammaproteobacteria;o	Ectothiorhodospirales;f	Thioalkalivibrionaceae;g	Thioalkalivibrio B;s	Thioalkalivibrio B sp007692435
SRR7901804MAG037	Yes	78.92	5.41	2907051	740	4567	62.4	92.7	2947	d	Bacteria;p	Verrucomicrobiota;c	Kiritimatiellae;o	SS1-B-03-39;f	UBA6053;g	PXAS01;s	
SRR7901804MAG038	Yes	89.93	2.25	3750858	534	9811	60.3	89.15	3446	d	Bacteria;p	Planctomycetota;c	UBA11346;o	UBA11346;f	UBA11346;g	REDP01;s	REDP01 sp007695355
SRR7901804MAG039	No	96.77	4.8	4114610	592	11079	57.1	93.87	4188	d	Bacteria;p	Spirochaetota;c	Spirochaetia;o	DSM-27196;f	SKKC01;g	SKKC01;s	SKKC01 sp007694005
SRR7901804MAG040	No	85.12	4.78	2170681	373	6972	53.8	91.75	2359	d	Bacteria;p	Proteobacteria;c	Alphaproteobacteria;o	Micavibrionales;f	UBA2020;g	CSBR16-224;s	
SRR7901804MAG041	No	77.11	3.38	3665090	802	6455	52.8	87.54	3729	d	Bacteria;p	Cyanobacteria;c	Cyanobacteriia;o	Cyanobacteriales;f	Geitlerinemaceae;g	Phormidium A;s	Phormidium A sp007693465
SRR7901804MAG042	Yes	86.73	3.43	3742823	609	7757	57	84.07	3674	d	Bacteria;p	Cyanobacteria;c	Cyanobacteriia;o	Phormidesmiales;f	Phormidesmiaceae;g	PXDR01;s	
SRR7901804MAG044	No	82.05	1.79	3226697	330	17428	69.8	89.82	3235	d	Bacteria;p	Actinobacteriota;c	Actinomycetia;o	Nitriliruptorales;f	Nitriliruptoraceae;g	CSSed11-175R1;s	CSSed11-175R1 sp007136095
SRR7901804MAG045	No	79.82	4.31	2785353	768	4351	39	90.95	2714	d	Bacteria;p	Bacteroidota;c	Bacteroidia;o	Chitinophagales;f	REEL01;g	REEL01;s	
SRR7901804MAG046	Yes	88.68	4.19	3509712	628	10613	71.7	91.78	3648	d	Bacteria;p	Deinococcota;c	Deinococci;o	Deinococcales;f	Trueperaceae;g	CSSed10-48;s	
SRR7901804MAG047	No	86.89	9.45	3357186	584	8040	68.3	92.99	3511	d	Bacteria;p	Proteobacteria;c	Gammaproteobacteria;o	PWYM01;f	PWYM01;g	PWYM01;s	
SRR7901804MAG048	Yes	80.48	1.68	2122374	605	4299	44	91.62	2300	d	Bacteria;p	Bacteroidota;c	Bacteroidia;o	Flavobacteriales;f	Schleiferiaceae;g	CSBr16-58;s	
SRR7901804MAG053	Yes	89.64	0	2684518	364	10098	65.7	88.71	2423	d	Bacteria;p	Planctomycetota;c	UBA11346;o	UBA11346;f	UBA11346;g	;s	
SRR7901804MAG054	Yes	93.29	9.88	3716837	337	21634	64	90.74	3823	d	Bacteria;p	Proteobacteria;c	Alphaproteobacteria;o	Rhodobacterales;f	Rhodobacteraceae;g	Roseinatronobacter;s	Roseinatronobacter sp007695345
SRR7901804MAG056	Yes	96.88	9.58	5107006	731	9790	63.9	93.91	5158	d	Bacteria;p	Spirochaetota;c	Spirochaetia;o	DSM-27196;f	SLST01;g	SLST01;s	
SRR7901804MAG058	Yes	76.63	9.9	4127781	492	16708	65.1	89.79	4107	d	Bacteria;p	Proteobacteria;c	Gammaproteobacteria;o	Pseudomonadales;f	HTCC2089;g	SLTB01;s	SLTB01 sp007694905
SRR7901804MAG059	No	76.78	4.33	3040085	1097	3235	68.5	91.44	3617	d	Bacteria;p	Proteobacteria;c	Alphaproteobacteria;o	Geminicoccales;f	Geminicoccaceae;g	RECO01;s	RECO01 sp007694015
SRR7901804MAG066	No	83.33	4.7	3370863	395	12850	69.9	92.47	3488	d	Bacteria;p	Actinobacteriota;c	Acidimicrobiia;o	Acidimicrobiales;f	Microtrichaceae;g	SKRR01;s	
SRR7901804MAG068	Yes	84.6	7.58	3851339	1015	4438	71.4	93.52	4272	d	Bacteria;p	Actinobacteriota;c	Acidimicrobiia;o	Acidimicrobiales;f	Ilumatobacteraceae;g	SKSP01;s	
SRR7901804MAG071	No	70.36	7.76	2599700	1267	2206	61.6	94.99	3346	d	Bacteria;p	Spirochaetota;c	Spirochaetia;o	DSM-27196;f	Alkalispirochaetaceae;g	Alkalispirochaeta;s	Alkalispirochaeta sp007695175
SRR7901804MAG098	Yes	95.33	5.77	2887046	263	16501	63.1	90.6	2942	d	Bacteria;p	Proteobacteria;c	Alphaproteobacteria;o	Rhodobacterales;f	Rhodobacteraceae;g	Roseinatronobacter;s	
SRR7901805MAG001	Yes	96.43	2.93	2922604	270	18396	67.7	86.97	2861	d	Bacteria;p	Proteobacteria;c	Gammaproteobacteria;o	Ectothiorhodospirales;f	Thioalkalivibrionaceae;g	Thioalkalivibrio B;s	Thioalkalivibrio B sp007692435
SRR7901805MAG002	Yes	96.79	2.37	1674110	159	23761	69.8	92.56	1650	d	Bacteria;p	Actinobacteriota;c	Actinomycetia;o	Nitriliruptorales;f	Nitriliruptoraceae;g	CSBr16-57R1;s	
SRR7901805MAG003	No	92.26	8.73	3730580	375	22771	64.2	91.15	3840	d	Bacteria;p	Proteobacteria;c	Alphaproteobacteria;o	Rhodobacterales;f	Rhodobacteraceae;g	Roseinatronobacter;s	Roseinatronobacter sp007695345
SRR7901805MAG004	Yes	99.77	2.99	3194945	292	17014	65.6	87.23	3175	d	Bacteria;p	Proteobacteria;c	Gammaproteobacteria;o	Ectothiorhodospirales;f	Thioalkalivibrionaceae;g	Thioalkalivibrio B;s	Thioalkalivibrio B sp007692535
SRR7901805MAG005	Yes	88.62	1.91	2927680	445	7552	45.1	86.31	2825	d	Bacteria;p	Bacteroidota;c	Rhodothermia;o	Balneolales;f	Balneolaceae;g	QGGB01;s	QGGB01 sp007693295
SRR7901805MAG006	Yes	92.16	2.7	2516875	227	14880	55.8	90.48	2497	d	Bacteria;p	Verrucomicrobiota;c	Verrucomicrobiae;o	Opitutales;f	DSM-45221;g	UBA7441;s	UBA7441 sp007692545
SRR7901805MAG007	Yes	96.03	1.04	2519374	326	11965	45.5	89.43	2673	d	Bacteria;p	Firmicutes;c	Bacilli;o	Bacillales H;f	Salisediminibacteriaceae;g	Salisediminibacterium;s	
SRR7901805MAG008	No	95.06	3.42	3367818	348	15075	72.2	89.49	3293	d	Bacteria;p	Actinobacteriota;c	Actinomycetia;o	Nitriliruptorales;f	Nitriliruptoraceae;g	T1Sed10-7;s	T1Sed10-7 sp003561535
SRR7901805MAG009	Yes	93.26	4.27	2901371	468	8122	57.5	91.8	3036	d	Bacteria;p	Proteobacteria;c	Gammaproteobacteria;o	Pseudomonadales;f	Pseudomonadaceae;g	Pseudomonas D;s	Pseudomonas D sp007692485
SRR7901805MAG010	Yes	93.73	1.19	4163789	695	7978	53.4	87.87	3935	d	Bacteria;p	Bacteroidota;c	Bacteroidia;o	Flavobacteriales;f	Cryomorphaceae;g	T3Sed10-241;s	
SRR7901805MAG011	Yes	95.21	3.32	4390905	446	14956	39	88.95	4045	d	Bacteria;p	Bacteroidota;c	Bacteroidia;o	Cytophagales;f	Cyclobacteriaceae;g	Mongoliibacter;s	Mongoliibacter sp007692705
SRR7901805MAG012	Yes	98.77	5.68	4355717	225	33570	69.6	91.6	3731	d	Bacteria;p	Gemmatimonadota;c	Gemmatimonadetes;o	Longimicrobiales;f	UBA6960;g	SKNW01;s	SKNW01 sp007692665
SRR7901805MAG013	Yes	95.64	7.01	3316698	393	19071	46.2	80.89	3256	d	Bacteria;p	Firmicutes;c	Bacilli;o	Bacillales H;f	Salisediminibacteriaceae;g	Alkalicoccus;s	Alkalicoccus sp007692495
SRR7901805MAG014	Yes	93.96	7.99	3679024	433	12858	67.6	91.16	3425	d	Bacteria;p	Gemmatimonadota;c	Gemmatimonadetes;o	Longimicrobiales;f	UBA6960;g	SLBA01;s	SLBA01 sp007692605
SRR7901805MAG016	Yes	95.35	1.23	3608824	193	31405	53.9	90.26	3459	d	Bacteria;p	Proteobacteria;c	Gammaproteobacteria;o	Pseudomonadales;f	Halomonadaceae;g	Halomonas;s	Halomonas sp007692655
SRR7901805MAG018	Yes	87.25	1.49	2466206	410	7499	64.1	91.81	2625	d	Bacteria;p	Proteobacteria;c	Alphaproteobacteria;o	Sphingomonadales;f	Sphingomonadaceae;g	Alteraurantiacibacter;s	
SRR7901805MAG019	Yes	91.23	5.56	2870032	138	35210	63	91.6	2582	d	Bacteria;p	Proteobacteria;c	Gammaproteobacteria;o	Xanthomonadales;f	Wenzhouxiangellaceae;g	Wenzhouxiangella;s	
SRR7901805MAG020	Yes	74.24	2	2339851	446	6033	41.4	88.21	2643	d	Bacteria;p	Cyanobacteria;c	Cyanobacteriia;o	Cyanobacteriales;f	;g	;s	
SRR7901805MAG021	No	97.64	3.99	5070443	339	21756	55.3	83.69	4827	d	Bacteria;p	Cyanobacteria;c	Cyanobacteriia;o	Phormidesmiales;f	Phormidesmiaceae;g	Nodosilinea;s	Nodosilinea sp007135385
SRR7901805MAG022	Yes	74.83	0.33	1714697	483	4022	47.1	92.4	2090	d	Bacteria;p	Proteobacteria;c	Gammaproteobacteria;o	Nitrosococcales;f	Methylophagaceae;g	Methylophaga;s	
SRR7901805MAG023	Yes	93.86	7.05	3635643	131	41611	61	91.09	3182	d	Bacteria;p	Proteobacteria;c	Gammaproteobacteria;o	Xanthomonadales;f	Wenzhouxiangellaceae;g	Wenzhouxiangella;s	
SRR7901805MAG025	Yes	84.42	4.08	2058907	371	6834	63.4	92.69	2186	d	Bacteria;p	Actinobacteriota;c	Actinomycetia;o	Actinomycetales;f	Micrococcaceae;g	Nesterenkonia;s	
SRR7901805MAG027	Yes	86.81	5.31	2743364	449	8019	70.6	91.91	2617	d	Bacteria;p	Gemmatimonadota;c	Gemmatimonadetes;o	Longimicrobiales;f	UBA6960;g	REBX01;s	REBX01 sp007692505
SRR7901805MAG028	No	85.29	2.72	3155236	500	8380	38.2	86.63	3024	d	Bacteria;p	Bacteroidota;c	Bacteroidia;o	Chitinophagales;f	Saprospiraceae;g	PWJY01;s	PWJY01 sp007693915
SRR7901805MAG029	Yes	78.49	7.37	2333626	839	3055	69.1	91.74	2815	d	Bacteria;p	Gemmatimonadota;c	Gemmatimonadetes;o	Longimicrobiales;f	Longimicrobiaceae;g	SKRI01;s	
SRR7901805MAG030	Yes	94.4	5.09	2803342	406	9746	65.1	90.51	3376	d	Archaea;p	Halobacteriota;c	Halobacteria;o	Halobacteriales;f	Halalkalicoccaceae;g	Halalkalicoccus;s	
SRR7901805MAG031	Yes	83.38	9.47	3817335	1116	3769	69.1	88.25	4151	d	Bacteria;p	Gemmatimonadota;c	Gemmatimonadetes;o	Longimicrobiales;f	Longimicrobiaceae;g	JACDHR01;s	
SRR7901805MAG032	Yes	91.02	6.36	3522694	702	6469	59.3	90.86	3584	d	Bacteria;p	Verrucomicrobiota;c	Verrucomicrobiae;o	Verrucomicrobiales;f	Akkermansiaceae;g	Luteolibacter;s	
SRR7901805MAG033	Yes	91.17	6.57	2453087	296	21266	41.3	86.2	2505	d	Bacteria;p	Firmicutes;c	Bacilli;o	Lactobacillales;f	Carnobacteriaceae;g	Alkalibacterium;s	Alkalibacterium sp007692455
SRR7901805MAG037	Yes	85.98	2.93	3480789	438	10213	59.7	90.81	3629	d	Bacteria;p	Proteobacteria;c	Alphaproteobacteria;o	Rhodobacterales;f	Rhodobacteraceae;g	Roseinatronobacter;s	
SRR7901805MAG044	Yes	93.35	4.6	3103412	429	10717	33.9	88.39	2999	d	Bacteria;p	Bacteroidota;c	Bacteroidia;o	Flavobacteriales;f	Flavobacteriaceae;g	Psychroflexus;s	
SRR7901805MAG045	No	84.11	9.47	3292495	770	5804	60	90.1	3695	d	Bacteria;p	Proteobacteria;c	Alphaproteobacteria;o	Rhodobacterales;f	Rhodobacteraceae;g	Roseinatronobacter;s	Roseinatronobacter monicus

SRR7901805MAG046	Yes	94.48	1.27	3573079	408	12122	58.2	90.64	3479	d Bacteria;p Proteobacteria;c Gammaproteobacteria;o Pseudomonadales;f Oleiphilaceae;g REBR01;s REBR01 sp007692645
SRR7901805MAG048	Yes	81.49	7.18	2546356	435	8320	64.9	92.66	2639	d Bacteria;p Proteobacteria;c Gammaproteobacteria;o Xanthomonadales;f Wenzhouxiangellaceae;g Wenzhouxiangella;s Wenzhouxiangella sp007694885
SRR7901805MAG050	Yes	81.77	4.26	3342011	642	6545	70.3	90.8	3706	d Bacteria;p Proteobacteria;c Alphaproteobacteria;o Rhodobacterales;f Rhodobacteraceae;g Rhodobaculum;s
SRR7901805MAG056	Yes	85.85	5.75	3567106	586	8221	64.9	89.07	3683	d Bacteria;p Proteobacteria;c Alphaproteobacteria;o Rhizobiales;f Beijerinckiaceae;g Salinarimonas;s
SRR7901805MAG059	Yes	85.88	4.33	3628415	287	22121	57.4	88.76	3804	d Bacteria;p Proteobacteria;c Alphaproteobacteria;o Rhodobacterales;f Rhodobacteraceae;g Roseinatronobacter;s
SRR7901805MAG065	Yes	86.98	5.78	3481832	812	5151	64.4	91.44	3854	d Bacteria;p Proteobacteria;c Gammaproteobacteria;o Pseudomonadales;f Halomonadaceae;g Halomonas B;s Halomonas B sp014779595
SRR7901805MAG066	Yes	91.63	2.41	5205891	429	19575	66.7	89.26	4778	d Bacteria;p Proteobacteria;c Gammaproteobacteria;o Pseudomonadales;f HTCC2089;g SLTB01;s
SRR7901805MAG068	Yes	82.02	5.34	3457221	409	12964	60.8	90.86	3685	d Bacteria;p Proteobacteria;c Alphaproteobacteria;o Rhodobacterales;f Rhodobacteraceae;g Roseinatronobacter;s
SRR7901805MAG080	Yes	89.55	5.56	3004459	281	19277	72.2	90.84	2920	d Bacteria;p Actinobacteriota;c Actinomycetia;o Nitriliruptorales;f Nitriliruptoraceae;g T1Sed10-7;s
SRR7901806MAG001	Yes	97.39	7.01	3178047	128	55705	67.4	88.71	3029	d Bacteria;p Proteobacteria;c Alphaproteobacteria;o Rhodospirillales;f UXAT02;g REEP01;s REEP01 sp007694935
SRR7901806MAG002	Yes	94.28	1.12	3296658	115	50914	66.2	88.86	3066	d Bacteria;p Proteobacteria;c Alphaproteobacteria;o Rhodospirillales;f UXAT02;g REEP01;s REEP01 sp007695045
SRR7901806MAG003	No	94.68	2.85	3545539	142	50906	42.3	87.03	2755	d Bacteria;p Bacteroidota;c Bacteroidia;o Chitinophagales;f Saprospiraceae;g PWJY01;s PWJY01 sp007695145
SRR7901806MAG004	Yes	97.85	6.45	3340190	314	19661	46.2	89.48	2852	d Bacteria;p Bacteroidota;c Bacteroidia;o Bacteroidales;f UBA7960;g PUPG01;s PUPG01 sp007695305
SRR7901806MAG005	Yes	98.36	0.22	4729991	172	37458	44.4	86.67	4004	d Bacteria;p Cyanobacteria;c Cyanobacteriia;o Cyanobacteriales;f Microcoleaceae;g Limnospira;s Limnospira platensis
SRR7901806MAG006	Yes	98.49	2.49	3926010	246	28928	57.6	88.13	3722	d Bacteria;p Desulfobacterota I;c Desulfovibrionia;o Desulfovibrionales;f Desulfonatronaceae;g Desulfonatronum;s Desulfonatronum sp007693505
SRR7901806MAG007	No	95.08	3.72	2752682	237	17757	47.8	89.4	2437	d Bacteria;p Bacteroidota;c Rhodothermia;o Balneolales;f Balneolaceae;g UBA2664;s UBA2664 sp007693745
SRR7901806MAG008	Yes	97.58	1.18	5716469	339	28147	56.1	87.63	4518	d Bacteria;p Planctomycetota;c Planctomycetia;o Pirellulales;f Pirellulaceae;g Roseimaritima;s
SRR7901806MAG009	No	93.64	0	3433095	211	25028	58.9	92.39	2966	d Bacteria;p Chloroflexota;c Anaerolineae;o Aggregatilineales;f A4b;g J038;s J038 sp007693385
SRR7901806MAG010	Yes	97.6	3.25	3868268	418	14099	60.8	93.67	3875	d Bacteria;p Spirochaetota;c Spirochaetia;o DSM-27196;f Alkalispirochaetaceae;g Alkalispirochaeta;s Alkalispirochaeta sp007695175
SRR7901806MAG012	Yes	83.27	5.34	2726849	362	12813	64.6	92.66	2717	d Bacteria;p Proteobacteria;c Gammaproteobacteria;o Xanthomonadales;f Wenzhouxiangellaceae;g Wenzhouxiangella;s
SRR7901806MAG013	No	95.6	4.4	4291529	303	26280	67.8	89.9	3787	d Bacteria;p Gemmatimonadota;c Gemmatimonadetes;o Longimicrobiales;f UBA6960;g T3Sed10-66;s T3Sed10-66 sp003564555
SRR7901806MAG014	Yes	96.99	3.18	4514047	448	14811	65.1	90.18	3835	d Bacteria;p Verrucomicrobiota;c Verrucomicrobiae;o Opitutales;f Opitutaceae;g REEB01;s REEB01 sp007695295
SRR7901806MAG015	No	87.98	2.77	4022863	630	12040	45.5	86.23	3691	d Bacteria;p Bacteroidota;c Rhodothermia;o Balneolales;f Balneolaceae;g QGGB01;s QGGB01 sp007693295
SRR7901806MAG016	Yes	91.1	2.37	6764709	1169	7006	60	87.71	5889	d Bacteria;p Planctomycetota;c Planctomycetia;o Pirellulales;f Pirellulaceae;g SKKK01;s
SRR7901806MAG017	Yes	74.86	6.67	3059934	852	3988	58.7	91.14	3219	d Bacteria;p Verrucomicrobiota;c Kiritimatiellae;o SS1-B-03-39;f UBA6053;g PXAS01;s
SRR7901806MAG018	No	96.3	1.82	3433572	157	44967	45.5	87.34	2827	d Bacteria;p Bacteroidota;c Bacteroidia;o Chitinophagales;f Saprospiraceae;g PWJY01;s PWJY01 sp007695035
SRR7901806MAG019	No	95.25	2.17	3103518	366	11280	49.5	92.58	2953	d Bacteria;p Bacteroidota;c Bacteroidia;o Flavobacteriales;f Cryomorphaceae;g SKUL01;s SKUL01 sp007695365
SRR7901806MAG020	Yes	98.23	1.36	2596546	121	38681	69.7	90.76	2824	d Bacteria;p Cyanobacteria;c Cyanobacteriia;o PCC-6307;f Cyanobiaceae;g NIES-981;s NIES-981 sp007694915
SRR7901806MAG021	No	92.67	3.08	3286232	200	35200	62.5	90.23	3309	d Bacteria;p Proteobacteria;c Alphaproteobacteria;o Rhodobacterales;f Rhodobacteraceae;g Roseinatronobacter;s Roseinatronobacter sp007694945
SRR7901806MAG022	Yes	94.29	7.77	4255483	536	32129	48.7	90.94	3469	d Bacteria;p Bacteroidota;c Bacteroidia;o Bacteroidales;f PUMT01;g PXAP01;s PXAP01 sp007695015
SRR7901806MAG023	Yes	84.97	7.63	3180054	560	7645	55	87.93	3146	d Bacteria;p Verrucomicrobiota;c Kiritimatiellae;o B70-G9;f ;g ;s
SRR7901806MAG024	No	97.69	3.27	3787775	239	23972	69.5	89.95	3659	d Bacteria;p Proteobacteria;c Alphaproteobacteria;o Rhodobacterales;f Rhodobacteraceae;g Pararhodobacter;s Pararhodobacter sp007131945
SRR7901806MAG025	No	96.77	1.28	3679555	179	58322	69.3	93.66	3551	d Bacteria;p Actinobacteriota;c Acidimicrobiia;o Acidimicrobiales;f Ilumatobacteraceae;g SKSP01;s SKSP01 sp007693335
SRR7901806MAG026	No	96.65	4.26	4921275	333	21505	55.4	83.59	4636	d Bacteria;p Cyanobacteria;c Cyanobacteriia;o Phormidesmiales;f Phormidesmiaceae;g Nodosilinea;s Nodosilinea sp007135385
SRR7901806MAG027	Yes	88.03	2.67	2814978	128	67116	67.1	92.74	2662	d Bacteria;p Proteobacteria;c Gammaproteobacteria;o GCA-2729495;f GCA-2729495;g ;s
SRR7901806MAG028	No	90.35	6.12	4651666	507	14907	43.3	86.89	4301	d Bacteria;p Bacteroidota;c Rhodothermia;o Balneolales;f Balneolaceae;g UBA2664;s UBA2664 sp007694095
SRR7901806MAG029	Yes	99.73	3.2	3458995	428	11604	54.8	93.27	3484	d Bacteria;p Spirochaetota;c Spirochaetia;o DSM-27196;f SLAA01;g SLAA01;s SLAA01 sp007695055
SRR7901806MAG030	Yes	93.82	3.01	4050228	427	19532	40.4	89.11	3459	d Bacteria;p Bacteroidota;c Bacteroidia;o Bacteroidales;f UBA7960;g SKVR01;s SKVR01 sp007693555
SRR7901806MAG031	Yes	99.2	4.45	4447313	462	15964	60.4	92.96	4333	d Bacteria;p Spirochaetota;c Spirochaetia;o DSM-27196;f PXBQ01;g PXBQ01;s PXBQ01 sp007695285
SRR7901806MAG032	No	94.08	0.93	3516157	535	9593	58.7	89.05	3591	d Bacteria;p Chloroflexota;c Chloroflexia;o Thermomicrobiales;f Thermomicrobiaceae;g SLMJ01;s SLMJ01 sp007695115
SRR7901806MAG033	Yes	88.64	4.73	2869404	476	7915	48.2	87.77	2766	d Bacteria;p Chloroflexota;c Anaerolineae;o Anaerolineales;f Anaerolineaceae;g Brevefilum;s
SRR7901806MAG034	Yes	74.9	1.15	2956284	544	7179	53.2	91.42	3251	d Bacteria;p Proteobacteria;c Gammaproteobacteria;o Competibacterales;f Competibacteraceae;g SKOM01;s
SRR7901806MAG035	Yes	97.3	3.91	4282103	481	19695	67.7	90.54	3854	d Bacteria;p Gemmatimonadota;c Gemmatimonadetes;o Longimicrobiales;f UBA6960;g PWLA01;s PWLA01 sp007695195
SRR7901806MAG037	No	85.97	5.61	3610358	641	7058	38.2	87.3	3516	d Bacteria;p Bacteroidota;c Bacteroidia;o Chitinophagales;f Saprospiraceae;g PWJY01;s PWJY01 sp007693915
SRR7901806MAG038	Yes	91.24	1.71	3818280	391	16017	69.6	92.02	3859	d Bacteria;p Actinobacteriota;c Acidimicrobiia;o Acidimicrobiales;f Microtrichaceae;g SKRR01;s
SRR7901806MAG039	Yes	82.02	5.28	4062097	1057	4425	43.4	89.25	4360	d Bacteria;p Bacteroidota;c Bacteroidia;o Cytophagales;f Cyclobacteriaceae;g Negadavirga;s
SRR7901806MAG040	Yes	95.55	2.3	3666181	418	14798	63.1	88.99	3775	d Bacteria;p Proteobacteria;c Gammaproteobacteria;o Competibacterales;f Competibacteraceae;g REEE01;s REEE01 sp007695245
SRR7901806MAG041	No	95.9	2.8	3451207	462	10205	57.3	94.74	3453	d Bacteria;p Spirochaetota;c Spirochaetia;o DSM-27196;f SKKC01;g SKKC01;s SKKC01 sp007694005
SRR7901806MAG042	No	84.15	2.33	3120918	713	5143	53.9	90.31	3212	d Bacteria;p Bacteroidota;c Bacteroidia;o Flavobacteriales;f Cryomorphaceae;g T3Sed10-241;s
SRR7901806MAG043	Yes	73.86	4.75	2647873	465	6949	68	89.98	2507	d Bacteria;p Planctomycetota;c Phycisphaerae;o Phycisphaerales;f UBA1924;g SLFH01;s SLFH01 sp007694925
SRR7901806MAG044	Yes	91.75	4.41	3243575	580	9722	44.9	90.3	2872	d Bacteria;p Bacteroidota;c Bacteroidia;o Bacteroidales;f UBA7960;g SKTA01;s
SRR7901806MAG045	Yes	87.43	7.74	3408201	542	9842	68.2	92.8	3532	d Bacteria;p Proteobacteria;c Gammaproteobacteria;o PWYM01;f PWYM01;g PWYM01;s
SRR7901806MAG046	Yes	78.27	6.55	2408449	304	14061	63.8	94.06	2469	d Bacteria;p Proteobacteria;c Gammaproteobacteria;o Xanthomonadales;f Wenzhouxiangellaceae;g Wenzhouxiangella;s
SRR7901806MAG047	No	90	7.77	3656481	439	12654	64.4	84.91	3167	d Bacteria;p Planctomycetota;c Phycisphaerae;o Phycisphaerales;f SM1A02;g SKZB01;s
SRR7901806MAG048	Yes	86.03	2.17	3092657	205	29806	62.7	92.38	2803	d Bacteria;p Proteobacteria;c Gammaproteobacteria;o Xanthomonadales;f Wenzhouxiangellaceae;g Wenzhouxiangella;s Wenzhouxiangella sp007695005
SRR7901806MAG049	No	85.73	5.16	3145044	421	12347	71.9	89.1	3162	d Bacteria;p Actinobacteriota;c Actinomycetia;o Nitriliruptorales;f Nitriliruptoraceae;g T1Sed10-7;s T1Sed10-7 sp003561535
SRR7901806MAG051	Yes	74.72	0.5	662567	198	3562	29.2	92.01	780	d Bacteria;p Patescibacteria;c ABY1;o BM507;f UBA12465;g PWHG01;s
SRR7901806MAG052	Yes	82	5.67	2729746	281	17997	64.3	92.34	2680	d Bacteria;p Proteobacteria;c Gammaproteobacteria;o Xanthomonadales;f Wenzhouxiangellaceae;g Wenzhouxiangella;s
SRR7901806MAG054	No	82.08	4.34	2525615	1166	2388	44.7	92.1	3246	d Bacteria;p Proteobacteria;c Gammaproteobacteria;o Pseudomonadales;f Nitrincolaceae;g Nitrincola;s Nitrincola sp007693685
SRR7901806MAG057	Yes	90.17	6.67	3154933	517	8408	67.3	88.08	3274	d Bacteria;p Proteobacteria;c Alphaproteobacteria;o Rhodobacterales;f Rhodobacteraceae;g Pararhodobacter;s
SRR7901806MAG059	Yes	87.58	2.61	2727759	565	5784	66.3	91.76	2771	d Bacteria;p Acidobacteriota;c Thermoanaerobaculia;o Thermoanaerobaculales;f Thermoanaerobaculaceae;g ;s
SRR7901806MAG060	No	94.77	7.65	4807274	748	7886	63.7	93.67	4972	d Bacteria;p Spirochaetota;c Spirochaetia;o DSM-27196;f SLST01;g SLST01;s
SRR7901806MAG064	Yes	76.75	9.06	4588318	751	9696	70.9	86.62	4806	d Bacteria;p Actinobacteriota;c Actinomycetia;o Nitriliruptorales;f Nitriliruptoraceae;g SKJO01;s SKJO01 sp007134165
SRR7901806MAG065	Yes	82.99	5.82	2489937	525	5830	54.3	89.94	2309	d Bacteria;p Bacteroidota;c Bacteroidia;o Bacteroidales;f UBA7960;g PUPG01;s
SRR7901806MAG066	Yes	84.47	7.11	3263830	727	5572	68.6	89.09	3528	d Bacteria;p Proteobacteria;c Alphaproteobacteria;o Rhodobacterales;f Rhodobacteraceae;g PUOA01;s
SRR7901806MAG067	Yes	87.85	8.88	4485157	693	8407	51.8	87.77	4417	d Bacteria;p Cyanobacteria;c Cyanobacteriia;o Cyanobacteriales;f Geitlerinemaceae;g Phormidium A;s Phormidium A sp007693465
SRR7901806MAG071	No	76.06	4.37	2801665	913	3411	64.4	94.92	3253	d Bacteria;p Spirochaetota;c Spirochaetia;o DSM-27196;f SLST01;g SLST01;s SLST01 sp007694025
SRR7901806MAG072	Yes	87.92	1.75	2618062	609	5013	39.1	90.53	2596	d Bacteria;p Bacteroidota;c Bacteroidia;o Chitinophagales;f REEL01;g REEL01;s
SRR7901806MAG075	No	75.68	5.88	2807203	534	12621	66	89.08	2996	d Bacteria;p Proteobacteria;c Gammaproteobacteria;o Ectothiorhodospirales;f Thioalkalivibrionaceae;g Thioalkalivibrio B;s Thioalkalivibrio B sp003550945
SRR7901806MAG084	Yes	92.05	7.17	3162117	333	15226	69.3	91.21	3193	d Bacteria;p Proteobacteria;c Alphaproteobacteria;o Rhodobacterales;f Rhodobacteraceae;g PUOA01;s
SRR7901806MAG092	Yes	83.53	4.15	3156777	260	16292	67	93.76	3077	d Bacteria;p Proteobacteria;c Gammaproteobacteria;o PWYM01;f PWYM01;g PWYM01;s PWYM01 sp007693855
SRR7901806MAG108	Yes	70.71	1.31	1876546	610	3600	51.5	92.52	1970	d Bacteria;p Bacteroidota;c Bacteroidia;o Bacteroidales;f UBA7960;g PUPG01;s
SRR9330139MAG001	Yes	98.67	0	1398752	56	42892	40.2	92.49	1403	d Bacteria;p Firmicutes;c Bacilli;o Izemoplasmatales;f Izemoplasmataceae;g B1SED10-225;s B1SED10-225 sp003558105
SRR9330139MAG002	No	98.67	1.33	1468532	69	40915	42.2	91.72	1495	d Bacteria;p Firmicutes;c Bacilli;o Izemoplasmatales;f Izemoplasmataceae;g B1SED10-225;s B1SED10-225 sp003551405
SRR9330139MAG003	No	96.52	1.14	2840948	159	30416	51.8	90.04	3030	d Archaea;p Halobacteriota;c Halobacteria;o Halobacteriales;f Haloferacaceae;g Halalkalirubrum;s Halalkalirubrum sp003551725
SRR9330139MAG004	No	86.69	0.03	1259769	186	9525	39.1	92.14	1297	d Bacteria;p Proteobacteria;c Gammaproteobacteria;o Francisellales;f Francisellaceae;g ;s
SRR9330139MAG005	Yes	93.99	8.47	3617911	193	44054	44.6	86.33	3147	d Bacteria;p Bacteroidota;c Rhodothermia;o Balneolales;f Natronogracilivirgulaceae;g ;s
SRR9330139MAG006	No	94.7	1.98	1987910	91	89986	40.4	88.62	2003	d Bacteria;p Firmicutes D;c Dethiobacteria;o SKNC01;f SKNC01;g PWHX01;s PWHX01 sp003555415
SRR9330139MAG007	Yes	98.34	2.48	3299790	145	47788	65.2	92.12	3039	d Bacteria;p Proteobacteria;c Gammaproteobacteria;o Xanthomonadales;f Wenzhouxiangellaceae;g Wenzhouxiangella;s Wenzhouxiangella sp003560975
SRR9330139MAG009	No	94.62	0.54	2355954	181	18930	47.3	88.84	2025	d Bacteria;p Bacteroidota;c Bacteroidia;o Bacteroidales;f UBA7960;g PYGY01;s
SRR9330139MAG010	No	83.33	4.37	3402135	308	15092	43	87.37	3009	d Bacteria;p Bacteroidota;c Rhodothermia;o Balneolales;f Balneolaceae;g SLAU01;s SLAU01 sp007126675
SRR9330139MAG011	No	92.37	3.86	3191682	271	27004	62.5	87.37	2987	d Bacteria;p Bacteroidota;c Rhodothermia;o Rhodothermales;f Salinibacteraceae;g Tc-Br11-B2g6-7;s Tc-Br11-B2g6-7 sp001564055
SRR9330139MAG012	Yes	82.24	0	829399	22	54433	38.5	92.04	900	d Archaea;p Nanohaloarchaeota;c Nanosalinia;o Nanosalinales;f ;g ;s
SRR9330139MAG013	No	80.37	0	902683	60	26767	38.4	92.04	1019	d Archaea;p Nanohaloarchaeota;c Nanosalinia;o Nanosalinales;f ;g ;s
SRR9330139MAG014	No	98.29	3.5	2926109	210	25563	74.6	90.64	2804	d Bacteria;p Actinobacteriota;c Actinomycetia;o Euzebyales;f Egibacteraceae;g T1Sed10-49;s T1Sed10-49 sp003553795
SRR9330139MAG015	Yes	99.05	2.99	3103519	132	62698	73.8	90.27	2902	d Bacteria;p Actinobacteriota;c Actinomycetia;o Euzebyales;f Egibacteraceae;g PUKE01;s PUKE01 sp003553565
SRR9330139MAG016	No	93.77	3.23	1782995	266	10737	48.9	91.98	1931	d Bacteria;p Firmicutes;c Bacilli;o Izemoplasmatales;f Izemoplasmataceae;g B1SED10-225;s B1SED10-225 sp003554735
SRR9330139MAG017	No	98.71	4.97	3061044	237	19784	54.4	87.37	2748	d Bacteria;p Desulfobacterota;c Desulfobacteria;o Desulfobacterales;f SURF-3;g B1SED10-16;s B1SED10-16 sp003551985
SRR9330139MAG018	Yes	80.53	0	918018	26	56006	26	92.11	881	d Bacteria;p Patescibacteria;c JAEDAM01;o Absconditabacterales;f X112;g Vampirococcus A;s
SRR9330139MAG019	Yes	80.46	3.45	610953	33	19955	41.2	92.36	665	d Bacteria;p Patescibacteria;c Paceibacteria;o Paceibacterales;f PWPS01;g PWPS01;s

SRR9330139MAG020	No	87.85	0.93	645123	22	57578	43.4	92.76	764	d	Archaea;p	Aenigmatarchaeota;c Aenigmatarchaeia;o PWEA01;f PWEA01;g PWEA01;s PWEA01 sp003554845
SRR9330139MAG021	Yes	82.24	0.47	618900	27	53979	45.1	92.46	699	d	Archaea;p	Aenigmatarchaeota;c Aenigmatarchaeia;o PWEA01;f PWEA01;g PWEA01;s PWEA01 sp003550625
SRR9330139MAG022	No	95.34	1.48	3397403	231	40360	49.2	85.66	3138	d	Bacteria;p	Firmicutes D;c Dethiobacteria;o DTU022;f UBA8154;g SKMY01;s
SRR9330139MAG023	Yes	95.08	2.46	3977659	213	33244	44.7	84.91	3458	d	Bacteria;p	Bacteroidota;c Rhodothermia;o Balneolales;f Balneolaceae;g SW132;s
SRR9330139MAG024	Yes	88.32	0.93	776602	56	40556	33.6	89.27	943	d	Archaea;p	B1Sed10-29;c B1Sed10-29;o B1Sed10-29;f B1Sed10-29;g B1Sed10-29;s B1Sed10-29 sp003551125
SRR9330139MAG025	No	93.13	2.93	2911141	173	24032	68.6	87.35	3004	d	Archaea;p	Halobacteriota;c Halobacteria;o Halobacteriales;f Natrialbaceae;g Tc-Br11-E2g8;s Tc-Br11-E2g8 sp001564115
SRR9330139MAG026	Yes	96.59	3.45	3145739	806	5551	41	88.26	3747	d	Bacteria;p	Cyanobacteria;c Cyanobacteriia;o Cyanobacteriales;f Rubidibacteraceae;g Halothece;s Halothece natronophila
SRR9330139MAG027	No	81.15	0	1126609	83	25690	25.8	90.12	1044	d	Archaea;p	Nanoarchaeota;c Nanoarchaeia;o Woesearchaeales;f ;g ;s
SRR9330139MAG028	Yes	88.37	4.25	1677224	273	9083	35	87.37	1832	d	Archaea;p	Halobacteriota;c Methanosarcinia;o Methanosarcinales;f Methanosarcinaceae;g Methanosalsum;s Methanosalsum natronophilum
SRR9330139MAG029	No	78.74	2.59	801532	125	7852	46.3	83.53	844	d	Bacteria;p	Patescibacteria;c Paceibacteria;o UBA9983 A;f CSBR16-193;g ;s
SRR9330139MAG030	Yes	92.24	4.4	3831492	679	7739	61.1	86.17	3567	d	Bacteria;p	Hydrogenedentota;c Hydrogenedentia;o Hydrogenedentiales;f SLHB01;g SKRY01;s SKRY01 sp003552765
SRR9330139MAG031	No	94.3	1.78	2328767	313	9911	40.3	90.63	2384	d	Bacteria;p	Firmicutes F;c Halanaerobiia;o Halanaerobiales;f Halarsenatibacteraceae;g T1SED10-84;s T1SED10-84 sp003553485
SRR9330139MAG032	Yes	84.58	0	597367	19	48892	42	92.09	703	d	Archaea;p	Aenigmatarchaeota;c Aenigmatarchaeia;o PWEA01;f PWEA01;g PWEA01;s PWEA01 sp003553905
SRR9330139MAG033	Yes	96.9	4.18	2775611	126	38037	64.9	87.74	2929	d	Archaea;p	Halobacteriota;c Halobacteria;o Halobacteriales;f Haloarculaceae;g Natronomonas;s
SRR9330139MAG034	No	94.1	3.04	2052574	143	22955	62	88.88	2201	d	Archaea;p	Halobacteriota;c Halobacteria;o Halobacteriales;f Haloferacaceae;g PL-Br10-E2g29;s PL-Br10-E2g29 sp001563965
SRR9330139MAG035	No	96.36	3.68	2524657	72	130614	58.3	89.22	2657	d	Archaea;p	Halobacteriota;c Halobacteria;o Halobacteriales;f Haloferacaceae;g PL-Br10-E2g29;s
SRR9330139MAG036	Yes	85.99	4.45	2523116	281	11657	45.5	86.54	2596	d	Bacteria;p	Firmicutes D;c Dethiobacteria;o DTU022;f UBA8154;g SLJK01;s
SRR9330139MAG037	No	93.93	2.47	3422699	373	12975	59.8	90.61	3608	d	Bacteria;p	Proteobacteria;c Alphaproteobacteria;o Rhodobacterales;f Rhodobacteraceae;g Roseinatronobacter;s Roseinatronobacter monicus
SRR9330139MAG038	No	97.09	2	1177082	208	6909	49.3	92.9	1318	d	Bacteria;p	Firmicutes;c Bacilli;o Izemoplasmatales;f Izemoplasmataceae;g T1SED10-81;s T1SED10-81 sp003554025
SRR9330139MAG039	No	83.6	1.87	843021	73	18295	40.9	91.36	1052	d	Archaea;p	Nanohaloarchaeota;c Nanosalinia;o Nanosalinales;f Nanosalinaceae;g B1-Br10-U2g19;s B1-Br10-U2g19 sp001563905
SRR9330139MAG040	Yes	92.04	7.65	3533812	472	10663	54.4	89.95	3155	d	Bacteria;p	Bacteroidota;c Rhodothermia;o Balneolales;f Natronogracilivirgulaceae;g SKNL01;s SKNL01 sp007127695
SRR9330139MAG042	Yes	79.24	2.66	1869396	488	4215	47.6	90.29	2036	d	Bacteria;p	Firmicutes F;c Halanaerobiia;o ;f ;g ;s
SRR9330139MAG043	No	87.91	6.58	2312345	288	11730	61.5	90.08	2642	d	Archaea;p	Halobacteriota;c Halobacteria;o Halobacteriales;f Haloferacaceae;g Halohasta;s
SRR9330139MAG044	No	83.57	2.8	854753	61	28622	39.9	92	1117	d	Archaea;p	Nanohaloarchaeota;c Nanosalinia;o Nanosalinales;f Nanosalinaceae;g B1-Br10-U2g21;s B1-Br10-U2g21 sp001564145
SRR9330139MAG047	No	97	1.72	2698827	304	14325	49.7	90.86	2795	d	Bacteria;p	Firmicutes F;c Halanaerobiia;o Halanaerobiales;f Halarsenatibacteraceae;g Halarsenatibacter;s
SRR9330139MAG048	No	86.63	1.45	3310136	977	4094	57.1	85.57	3555	d	Bacteria;p	Bacteroidota;c Rhodothermia;o Rhodothermales;f Salinibacteraceae;g Longimonas;s
SRR9330139MAG049	Yes	86.34	2.67	1638217	219	12328	39	92.22	1810	d	Archaea;p	Thermoplasmatota;c Thermoplasmata;o PWKY01;f PWKY01;g B1SED10-34;s
SRR9330139MAG050	No	88.31	5.11	2756707	175	28438	64.3	88.12	2880	d	Archaea;p	Halobacteriota;c Halobacteria;o Halobacteriales;f Haloferacaceae;g Halorubrum;s Halorubrum sp001564205
SRR9330139MAG051	No	84.13	1.99	2961228	412	10770	71.3	89.74	2913	d	Bacteria;p	Actinobacteriota;c Actinomycetia;o Nitriliruptorales;f Nitriliruptoraceae;g T1Sed10-7;s
SRR9330139MAG053	No	83.74	2.73	1146817	428	3219	62.5	92.26	1525	d	Archaea;p	Thermoplasmatota;c Thermoplasmata;o Methanomassiliicoccales;f Methanomethylophilaceae;g PWHV01;s PWHV01 sp003555025
SRR9330139MAG054	No	88.89	1.17	3181689	179	27509	69.6	89.3	3201	d	Bacteria;p	Actinobacteriota;c Actinomycetia;o Nitriliruptorales;f Nitriliruptoraceae;g CSSed11-175R1;s CSSed11-175R1 sp007136095
SRR9330139MAG055	No	94.61	4.9	3282373	157	43469	72.1	89.74	3083	d	Bacteria;p	Actinobacteriota;c Actinomycetia;o Nitriliruptorales;f Nitriliruptoraceae;g T1Sed10-7;s T1Sed10-7 sp003554005
SRR9330139MAG056	No	72.92	1.45	845197	260	3463	51.3	92.46	1016	d	Bacteria;p	Firmicutes;c Bacilli;o Izemoplasmatales;f Izemoplasmataceae;g M55B118;s
SRR9330139MAG057	No	88.79	9.11	2470657	633	5435	43.8	90.24	2863	d	Bacteria;p	Firmicutes A;c Clostridia;o Peptostreptococcales;f T1SED10-28;g T1SED10-28;s T1SED10-28 sp003554105
SRR9330139MAG059	Yes	100	8.07	2235215	125	39472	33.5	90.82	2190	d	Bacteria;p	Firmicutes F;c Halanaerobiia;o Halanaerobiales;f Halanaerobiaceae;g Halanaerobium;s
SRR9330139MAG060	Yes	93.55	6.99	3099915	620	7413	68.7	91.83	3505	d	Bacteria;p	Proteobacteria;c Alphaproteobacteria;o Rhodobacterales;f Rhodobacteraceae;g Rhodobaculum;s
SRR9330139MAG064	Yes	71.65	1.4	1120711	162	9593	44.2	92.78	1291	d	Archaea;p	Nanoarchaeota;c Nanoarchaeia;o Woesearchaeales;f JAFGUC01;g ;s
SRR9330139MAG065	Yes	76.09	4.81	1844689	456	4833	43.4	87.53	2013	d	Bacteria;p	Firmicutes D;c Dethiobacteria;o SKNC01;f SKNC01;g PWHX01;s
SRR9330139MAG066	Yes	85.81	7.32	2954359	1099	3009	30.7	87.34	3347	d	Bacteria;p	Firmicutes F;c Halanaerobiia;o Halobacteroidales;f Halobacteroidaceae;g ;s
SRR9330139MAG068	No	94.71	3.36	2410593	257	16590	35.9	90.9	2493	d	Bacteria;p	Firmicutes F;c Halanaerobiia;o Halanaerobiales;f Halarsenatibacteraceae;g SLSL01;s SLSL01 sp007130465
SRR9330139MAG069	Yes	73.03	7.26	3588048	635	6395	34.6	89.74	3877	d	Bacteria;p	Firmicutes F;c Halanaerobiia;o Halanaerobiales;f Halarsenatibacteraceae;g SLSL01;s
SRR9330139MAG071	Yes	80.55	9.47	2616268	540	6409	58.9	89.04	3124	d	Archaea;p	Halobacteriota;c Halobacteria;o Halobacteriales;f Haloferacaceae;g Halohasta;s
SRR9330139MAG078	No	90.78	0	1130729	231	6481	53.4	93.26	1267	d	Bacteria;p	Firmicutes;c Bacilli;o Izemoplasmatales;f Izemoplasmataceae;g T1SED10-81;s T1SED10-81 sp003553945
SRR9330139MAG080	Yes	71.02	5.93	3453045	1114	3373	40.4	88.35	3623	d	Bacteria;p	Bacteroidota;c Bacteroidia;o Bacteroidales;f UBA7960;g SKVR01;s SKVR01 sp007132585
SRR9330139MAG089	Yes	83.11	8.89	1684656	296	8901	38.9	90.84	1906	d	Archaea;p	Thermoplasmatota;c Thermoplasmata;o PWKY01;f PWKY01;g B1SED10-34;s
SRR9330139MAG091	No	80.91	6.93	3330394	678	5808	57	91.5	3507	d	Bacteria;p	Spirochaetota;c Spirochaetia;o DSM-27196;f SKKC01;g SLAN01;s
SRR9330139MAG108	No	76.38	3.45	1389543	269	6308	64.8	95.43	1543	d	Bacteria;p	Proteobacteria;c Gammaproteobacteria;o Nitrococcales;f Nitrococcaceae;g Spiribacter;s Spiribacter sp009676705
SRR9330139MAG112	Yes	82.29	9.78	3606820	552	8894	44.3	87.63	3464	d	Bacteria;p	Bacteroidota;c Rhodothermia;o Balneolales;f Balneolaceae;g UBA2664;s
SRR9330140MAG001	No	95.92	0.65	1259659	34	68325	42.5	87.06	1341	d	Archaea;p	Halobacteriota;c Methanonatronarchaeia;o Methanonatronarchaeales;f Methanonatronarchaeaceae;g Methanonatronarchaeum;s Methanonatronarchaeum sp004212035
SRR9330140MAG002	No	96.89	2.73	3635596	301	27949	62.4	87.8	3351	d	Bacteria;p	Bacteroidota;c Rhodothermia;o Rhodothermales;f Salinibacteraceae;g Tc-Br11-B2g6-7;s Tc-Br11-B2g6-7 sp001564055
SRR9330140MAG003	Yes	96.01	3.42	4443587	518	20637	69.8	88.62	4577	d	Bacteria;p	Actinobacteriota;c Actinomycetia;o Nitriliruptorales;f Nitriliruptoraceae;g CSSed11-175R1;s CSSed11-175R1 sp007136095
SRR9330140MAG004	No	96.4	2.8	1869804	96	33560	42.4	90.52	1911	d	Archaea;p	Thermoplasmatota;c Thermoplasmata;o PWKY01;f PWKY01;g Natronoplasma;s
SRR9330140MAG005	No	98.22	2.61	3128746	141	39394	65.4	92.6	2912	d	Bacteria;p	Proteobacteria;c Gammaproteobacteria;o Xanthomonadales;f Wenzhouxiangellaceae;g Wenzhouxiangella;s Wenzhouxiangella sp003560975
SRR9330140MAG006	No	94.92	5.72	3357308	347	15808	48.9	85.46	3232	d	Bacteria;p	Firmicutes D;c Dethiobacteria;o DTU022;f UBA8154;g SKMY01;s
SRR9330140MAG007	No	85.38	2.12	1718604	118	25329	44.7	90.66	1759	d	Bacteria;p	Firmicutes D;c Dethiobacteria;o SKNC01;f SKNC01;g SKLX01;s SKLX01 sp007121375
SRR9330140MAG008	No	98.78	5.17	2995630	214	28674	66.2	87.39	2959	d	Bacteria;p	Proteobacteria;c Gammaproteobacteria;o Ectothiorhodospirales;f Thioalkalivibrionaceae;g Thioalkalivibrio B;s Thioalkalivibrio B sp003563455
SRR9330140MAG009	No	95.21	3.52	3151490	265	22343	65.9	90.76	3119	d	Bacteria;p	Proteobacteria;c Gammaproteobacteria;o Nitrococcales;f Aquisalimonadaceae;g Aquisalimonas;s Aquisalimonas sp012044895
SRR9330140MAG010	Yes	90	3.41	2639234	222	18426	50.7	90.08	2350	d	Bacteria;p	Verrucomicrobiota;c Kiritimatiellae;o UBA8416;f UBA8416;g T3Sed10-140;s
SRR9330140MAG011	Yes	93.39	2.05	1367463	179	12420	39	92.01	1392	d	Bacteria;p	Proteobacteria;c Gammaproteobacteria;o Francisellales;f Francisellaceae;g ;s
SRR9330140MAG012	No	97.42	4.36	3129578	282	17277	54.1	86.94	2925	d	Bacteria;p	Desulfobacterota;c Desulfobacteria;o Desulfobacterales;f SURF-3;g B1SED10-16;s B1SED10-16 sp003551985
SRR9330140MAG013	Yes	83.05	4.47	698142	35	41187	37.7	91.84	761	d	Bacteria;p	Patescibacteria;c Paceibacteria;o Paceibacterales;f PWPS01;g PWPS01;s PWPS01 sp003554445
SRR9330140MAG014	No	92.11	1.54	2325679	195	20344	41.4	87.49	2289	d	Bacteria;p	Firmicutes F;c Halanaerobiia;o Halanaerobiales;f Halarsenatibacteraceae;g Halarsenatibacter;s
SRR9330140MAG015	No	91.46	3.34	2329873	509	5793	66.4	89.32	2707	d	Archaea;p	Halobacteriota;c Halobacteria;o Halobacteriales;f Haloferacaceae;g Halorubrum;s Halorubrum sp003554605
SRR9330140MAG016	Yes	75.7	0.93	940264	288	4268	33.2	92.21	1271	d	Archaea;p	Nanoarchaeota;c Nanoarchaeia;o Woesearchaeales;f 21-14-0-10-32-9;g ;s
SRR9330140MAG017	Yes	98.28	2.17	3348052	169	33239	65.6	90.52	3178	d	Bacteria;p	Proteobacteria;c Gammaproteobacteria;o Pseudomonadales;f Halomonadaceae;g Halomonas;s
SRR9330140MAG019	No	93.55	5.73	4138155	603	12398	35.3	83.83	3991	d	Bacteria;p	Bacteroidota;c Bacteroidia;o Bacteroidales;f UBA7960;g PUKZ01;s
SRR9330140MAG020	No	89.65	6.55	2405719	553	5414	73.6	90.58	2620	d	Bacteria;p	Actinobacteriota;c Actinomycetia;o Euzebyales;f Egibacteraceae;g PUKE01;s PUKE01 sp003553565
SRR9330140MAG021	Yes	87.93	5.46	2011616	292	9216	53.8	87.16	2073	d	Bacteria;p	Desulfobacterota I;c Desulfovibrionia;o Desulfovibrionales;f Desulfonatronovibrionaceae;g Desulfonatronospira;s Desulfonatronospira sp003554505
SRR9330140MAG022	Yes	98.39	4.64	2611286	179	31913	49.8	91.12	2623	d	Bacteria;p	Firmicutes F;c Halanaerobiia;o Halanaerobiales;f Halarsenatibacteraceae;g Halarsenatibacter;s
SRR9330140MAG023	No	95.83	4.46	2105750	193	16160	35.8	91.46	2177	d	Bacteria;p	Firmicutes F;c Halanaerobiia;o Halanaerobiales;f Halarsenatibacteraceae;g SLSL01;s SLSL01 sp007130465
SRR9330140MAG027	No	73.22	7.03	2404465	608	4610	43.6	89.37	2608	d	Bacteria;p	Bacteroidota;c Rhodothermia;o Balneolales;f Balneolaceae;g SW132;s
SRR9330140MAG028	No	89.96	8.16	2732690	455	7798	48.2	90.63	2516	d	Bacteria;p	Bacteroidota;c Bacteroidia;o Bacteroidales;f UBA7960;g PUPG01;s PUPG01 sp007124575
SRR9330140MAG029	Yes	91.22	3.26	2055827	439	6274	53.2	91.24	2209	d	Bacteria;p	Proteobacteria;c Gammaproteobacteria;o Halothiobacillales;f Halothiobacillaceae;g Guyparkeria;s
SRR9330140MAG033	No	89.35	3.01	1689676	381	5996	55.2	95.96	2025	d	Bacteria;p	Proteobacteria;c Gammaproteobacteria;o Nitrococcales;f Nitrococcaceae;g Spiribacter;s
SRR9330140MAG034	No	70.04	0.91	2009976	561	4107	40	86.87	2119	d	Bacteria;p	Firmicutes F;c Halanaerobiia;o Halanaerobiales;f CSSED10-376;g T1SED10-99;s T1SED10-99 sp003553225
SRR9330141MAG001	Yes	98.55	0	1308333	28	62093	46.7	92.63	1298	d	Bacteria;p	Firmicutes;c Bacilli;o Izemoplasmatales;f Izemoplasmataceae;g B1SED10-225;s
SRR9330141MAG002	No	93.82	1.61	2312517	198	16864	47.4	89.18	1983	d	Bacteria;p	Bacteroidota;c Bacteroidia;o Bacteroidales;f UBA7960;g PYGY01;s
SRR9330141MAG003	No	93.17	1.64	3260582	138	43252	44.7	86.61	2804	d	Bacteria;p	Bacteroidota;c Rhodothermia;o Balneolales;f Natronogracilivirgulaceae;g ;s
SRR9330141MAG004	Yes	96.19	4.03	3364597	200	39040	49.1	85.66	3099	d	Bacteria;p	Firmicutes D;c Dethiobacteria;o DTU022;f UBA8154;g SKMY01;s
SRR9330141MAG005	Yes	97.07	3.33	2431990	212	26028	68.9	93.22	2399	d	Bacteria;p	Proteobacteria;c Gammaproteobacteria;o Nitrococcales;f Halorhodospiraceae;g Halorhodospira;s Halorhodospira halophila A
SRR9330141MAG006	Yes	92.91	5.33	1566183	59	98028	35.7	90.25	1538	d	Bacteria;p	Firmicutes;c Bacilli;o Izemoplasmatales;f Izemoplasmataceae;g B1SED10-225;s
SRR9330141MAG008	No	97.62	0.6	2528140	109	39383	51.4	85.05	2340	d	Bacteria;p	Desulfobacterota I;c Desulfovibrionia;o Desulfovibrionales;f Desulfonatronovibrionaceae;g ;s
SRR9330141MAG009	No	97.21	1.33	1365986	64	35921	49.3	92.71	1368	d	Bacteria;p	Firmicutes;c Bacilli;o Izemoplasmatales;f Izemoplasmataceae;g T1SED10-81;s T1SED10-81 sp003554025
SRR9330141MAG010	No	93.8	2.06	2972326	201	23438	54.3	87.15	2681	d	Bacteria;p	Desulfobacterota;c Desulfobacteria;o Desulfobacterales;f SURF-3;g B1SED10-16;s B1SED10-16 sp003551985
SRR9330141MAG011	No	93.22	1.69	3168335	282	17605	62.5	87.9	2918	d	Bacteria;p	Bacteroidota;c Rhodothermia;o Rhodothermales;f Salinibacteraceae;g Tc-Br11-B2g6-7;s Tc-Br11-B2g6-7 sp001564055
SRR9330141MAG012	Yes	98.27	2.34	2921082	211	23166	55.1	89.48	3107	d	Archaea;p	Halobacteriota;c Halobacteria;o Halobacteriales;f Haloarculaceae;g Halovenus;s Halovenus sp001564135
SRR9330141MAG013	No	95.83	6.34	2930944	329	12033	51.7	90.32	3284	d	Archaea;p	Halobacteriota;c Halobacteria;o Halobacteriales;f Haloferacaceae;g Halalkalirubrum;s Halalkalirubrum sp003551725
SRR9330141MAG014	No	82.89	3.45	619674	8	97368	40.3	90.69	649	d	Bacteria;p	Patescibacteria;c Paceibacteria;o Paceibacterales;f PWPS01;g PWHW01;s PWHW01 sp003555075
SRR9330141MAG015	No	76.57	0	728714	35	62663	33.5	92.49	738	d	Bacteria;p	Patescibacteria;c Dojkabacteria;o SC72;f T1SED10-24;g T1SED10-24;s T1SED10-24 sp003554185
SRR9330141MAG016	No	96.61	8.71	3551683	256	24088	38.1	87.14	3507	d	Bacteria;p	Firmicutes D;c Natranaerobiia;o Natranaerobiales;f ;g ;s

SRR9330141MAG017	Yes	91.88	0	1442190	113	21501	50.9	91.54	1477	d Bacteria;p Firmicutes;c Bacilli;o Izemoplasmatales;f Izemoplasmataceae;g M55B118;s
SRR9330141MAG019	Yes	98.68	0.66	2199148	83	68832	41.4	88.45	2058	d Bacteria;p Firmicutes F;c Halanaerobiia;o Halanaerobiales;f Halarsenatibacteraceae;g Halarsenatibacter;s
SRR9330141MAG020	Yes	91.94	1.08	2678728	339	10186	51.7	89.71	2317	d Bacteria;p Bacteroidota;c Bacteroidia;o Bacteroidales;f UBA7960;g PUPG01;s PUPG01 sp003553005
SRR9330141MAG021	No	88.14	5.48	1998320	260	12548	64.5	95.14	2121	d Bacteria;p Proteobacteria;c Gammaproteobacteria;o Nitrococcales;f Nitrococcaceae;g Spiribacter;s Spiribacter sp009676705
SRR9330141MAG022	No	98.67	1.7	1968803	121	150153	51.8	91.22	1995	d Bacteria;p Firmicutes;c Bacilli;o Izemoplasmatales;f Izemoplasmataceae;g T1SED10-81;s T1SED10-81 sp003553945
SRR9330141MAG024	Yes	94.92	5.57	3011803	226	23213	63.7	91.73	2946	d Bacteria;p Proteobacteria;c Gammaproteobacteria;o Ectothiorhodospirales;f Ectothiorhodospiraceae;g Ectothiorhodospira;s Ectothiorhodospira haloalkaliphila
SRR9330141MAG025	Yes	94.28	2.25	1785145	123	22102	38.9	92.25	1816	d Bacteria;p CG03;c SLGR01;o ;f ;g ;s
SRR9330141MAG026	Yes	76.34	1.12	613887	43	25503	30.8	92.44	639	d Bacteria;p Patescibacteria;c ABY1;o BM507;f UBA12465;g PWHG01;s PWHG01 sp003554785
SRR9330141MAG027	Yes	79.91	2.34	985689	51	36483	33.4	90.58	1067	d Archaea;p Nanoarchaeota;c Nanoarchaeia;o Woesearchaeales;f 21-14-0-10-32-9;g ;s
SRR9330141MAG028	Yes	82.24	0	850068	43	28692	38.3	92.12	946	d Archaea;p Nanohaloarchaeota;c Nanosalinia;o Nanosalinales;f ;g ;s
SRR9330141MAG029	No	98.68	1.48	2894578	125	81811	39.8	89.63	2720	d Bacteria;p Firmicutes F;c Halanaerobiia;o Halanaerobiales;f Halarsenatibacteraceae;g T1SED10-84;s T1SED10-84 sp003553485
SRR9330141MAG030	Yes	82.09	0.93	1028535	51	38362	25.2	88.55	1044	d Archaea;p Nanoarchaeota;c Nanoarchaeia;o Woesearchaeales;f ;g ;s
SRR9330141MAG031	No	93.03	5.24	2845519	379	10513	42.8	88.44	2584	d Bacteria;p Bacteroidota;c Bacteroidia;o Bacteroidales;f PUMT01;g PUMT01;s PUMT01 sp003554865
SRR9330141MAG032	Yes	77.19	0	2017351	132	31961	44	85.34	1858	d Bacteria;p Firmicutes F;c Halanaerobiia;o Halanaerobiales;f Halarsenatibacteraceae;g Halarsenatibacter;s
SRR9330141MAG033	No	84.12	2.31	1716377	483	3902	60.7	88.87	1868	d Bacteria;p Desulfobacterota;c Desulfobulbia;o Desulfobulbales;f Desulfurivibrionaceae;g Desulfurivibrio;s
SRR9330141MAG034	No	78.19	0	1096196	303	4115	41	92.55	1332	d Bacteria;p Firmicutes;c Bacilli;o Izemoplasmatales;f Izemoplasmataceae;g PWME01;s PWME01 sp003559235
SRR9330141MAG035	No	92.37	5.48	3261482	319	15706	66.1	91.33	3248	d Bacteria;p Proteobacteria;c Gammaproteobacteria;o Nitrococcales;f Aquisalimonadaceae;g Aquisalimonas;s Aquisalimonas sp012044895
SRR9330141MAG036	Yes	93.6	1.76	2200632	123	38379	38	89.12	2177	d Archaea;p Thermoplasmatota;c Thermoplasmata;o PWKY01;f PWKY01;g Natronoplasma;s
SRR9330141MAG037	No	84.58	1.87	673710	26	41080	41.8	91.93	797	d Archaea;p Aenigmatarchaeota;c Aenigmatarchaeia;o PWEA01;f PWEA01;g PWEA01;s PWEA01 sp003553905
SRR9330141MAG038	No	91.6	4	1752431	88	33561	42.6	91.16	1784	d Archaea;p Thermoplasmatota;c Thermoplasmata;o PWKY01;f PWKY01;g Natronoplasma;s
SRR9330141MAG039	No	90.98	4.58	2736997	158	27661	67.5	87.8	2857	d Archaea;p Halobacteriota;c Halobacteria;o Halobacteriales;f Haloferacaceae;g Halorubrum;s
SRR9330141MAG040	Yes	77.33	0	1384785	47	48519	39.3	93.03	1351	d Bacteria;p Firmicutes;c Bacilli;o Izemoplasmatales;f Izemoplasmataceae;g B1SED10-225;s
SRR9330141MAG041	Yes	91.53	2.03	1974567	260	9556	49.3	85.89	1940	d Bacteria;p T1Sed10-126;c T1Sed10-126;o T1Sed10-126;f T1Sed10-126;g T1Sed10-126;s
SRR9330141MAG043	Yes	96	7.05	3141784	295	16310	69.6	91.8	3311	d Bacteria;p Proteobacteria;c Alphaproteobacteria;o Rhodobacterales;f Rhodobacteraceae;g Rhodobaculum;s Rhodobaculum sp007131685
SRR9330141MAG044	Yes	87.38	1.87	742599	41	40058	35.9	89.27	882	d Archaea;p B1Sed10-29;c B1Sed10-29;o B1Sed10-29;f B1Sed10-29;g B1Sed10-29;s
SRR9330141MAG045	No	98.11	6.8	3649061	135	66328	57.1	91.77	3282	d Bacteria;p Spirochaetota;c Spirochaetia;o DSM-27196;f SKKC01;g SLAN01;s
SRR9330141MAG047	No	79.44	1.87	960587	70	22470	38.7	91.93	1057	d Archaea;p Nanohaloarchaeota;c Nanosalinia;o Nanosalinales;f ;g ;s
SRR9330141MAG048	No	84.4	6.33	2879381	320	15769	63.1	87.65	3150	d Archaea;p Halobacteriota;c Halobacteria;o Halobacteriales;f Haloferacaceae;g Halorubrum;s
SRR9330141MAG049	Yes	85.67	0.93	1622237	256	8556	33.8	85.79	1831	d Archaea;p Nanoarchaeota;c Nanoarchaeia;o Woesearchaeales;f DSVV01;g ;s
SRR9330141MAG052	Yes	76.54	5.33	586823	130	6096	43.1	91.79	676	d Bacteria;p Patescibacteria;c Paceibacteria;o Paceibacterales;f PWPS01;g PWHW01;s
SRR9330141MAG053	Yes	88.42	5.42	2686677	422	8137	42.1	86.77	2760	d Bacteria;p Firmicutes D;c Dethiobacteria;o DTU022;f PWGO01;g T1SED10-157;s
SRR9330141MAG054	No	86.08	2.22	4564153	377	19580	61.8	91.25	3819	d Bacteria;p Myxococcota;c Bradymonadia;o Bradymonadales;f Bradymonadaceae;g SLJM01;s
SRR9330141MAG055	Yes	95.15	6.79	1709733	239	10842	48.4	91.45	1838	d Bacteria;p Firmicutes;c Bacilli;o Izemoplasmatales;f Izemoplasmataceae;g B1SED10-225;s B1SED10-225 sp003554735
SRR9330141MAG056	Yes	74.3	2.8	863406	72	20382	38.1	91.77	1001	d Archaea;p Nanohaloarchaeota;c Nanosalinia;o Nanosalinales;f ;g ;s
SRR9330141MAG057	No	70.87	2.34	1153903	209	7812	44	93.17	1289	d Archaea;p Nanoarchaeota;c Nanoarchaeia;o Woesearchaeales;f JAFGUC01;g ;s
SRR9330141MAG058	Yes	90.61	3.61	1928495	133	23341	62	87.84	2089	d Archaea;p Halobacteriota;c Halobacteria;o Halobacteriales;f Haloferacaceae;g PL-Br10-E2g29;s
SRR9330141MAG059	No	91.95	6.54	2889580	300	25141	47.7	87.07	2812	d Bacteria;p Firmicutes D;c Dethiobacteria;o DTU022;f DTU022;g B1SED10-74M;s
SRR9330141MAG060	No	82.24	3.22	2546585	145	45567	66.1	88.12	2505	d Bacteria;p Proteobacteria;c Gammaproteobacteria;o Ectothiorhodospirales;f Thioalkalivibrionaceae;g Thioalkalivibrio B;s Thioalkalivibrio B sp003563455
SRR9330141MAG061	Yes	75.36	7.76	1230053	113	14554	29.5	89.34	1209	d Bacteria;p Patescibacteria;c JAEDAM01;o Absconditabacterales;f X112;g ;s
SRR9330141MAG062	No	96.08	6.16	2980857	613	6475	74.5	90.9	3252	d Bacteria;p Actinobacteriota;c Actinomycetia;o Euzebyales;f Egibacteraceae;g T1Sed10-49;s T1Sed10-49 sp003553795
SRR9330141MAG064	Yes	71.39	2.59	576436	56	15079	40.7	92.79	678	d Bacteria;p Patescibacteria;c Paceibacteria;o Paceibacterales;f PWPS01;g T1SED10-78;s
SRR9330141MAG065	No	79.07	6.92	1922424	291	8677	48.9	86.33	1906	d Bacteria;p Firmicutes D;c Dethiobacteria;o DTU022;f PWGO01;g PWGO01;s
SRR9330141MAG066	No	93.04	9.13	2337912	198	34097	33.5	90.89	2373	d Bacteria;p Firmicutes F;c Halanaerobiia;o Halanaerobiales;f Halanaerobiaceae;g Halanaerobium;s
SRR9330141MAG067	No	77.67	3.7	2106759	269	10849	61.5	89.1	2425	d Archaea;p Halobacteriota;c Halobacteria;o Halobacteriales;f Haloferacaceae;g Halohasta;s
SRR9330141MAG068	Yes	91.76	6.39	2651466	290	19927	66.4	93.34	2736	d Bacteria;p Proteobacteria;c Gammaproteobacteria;o Nitrococcales;f Halorhodospiraceae;g Halorhodospira;s Halorhodospira sp003552625
SRR9330141MAG069	No	74.86	5.59	2196909	506	5159	43.6	89.28	2366	d Bacteria;p Bacteroidota;c Rhodothermia;o Balneolales;f Balneolaceae;g SW132;s
SRR9330141MAG072	No	76.19	3.62	1556276	289	6993	62.6	89.42	1834	d Archaea;p Halobacteriota;c Halobacteria;o Halobacteriales;f Haloferacaceae;g PL-Br10-E2g29;s PL-Br10-E2g29 sp001563965
SRR9330141MAG074	No	98.15	7.78	2565429	384	9364	50.1	91.5	2721	d Bacteria;p Firmicutes F;c Halanaerobiia;o Halanaerobiales;f Halarsenatibacteraceae;g Halarsenatibacter;s
SRR9330141MAG076	No	79.69	2.4	2122962	225	14930	66.3	89.77	2258	d Archaea;p Halobacteriota;c Halobacteria;o Halobacteriales;f Haloferacaceae;g Halorubrum;s Halorubrum sp003554605
SRR9330141MAG083	Yes	70.49	2.34	1261210	178	10271	41.2	90.27	1428	d Archaea;p Nanoarchaeota;c Nanoarchaeia;o Woesearchaeales;f JAFGUC01;g ;s
SRR9330141MAG085	Yes	77.52	6.66	2655789	514	6416	46.9	90.12	2602	d Bacteria;p Bacteroidota;c Rhodothermia;o Balneolales;f Natronogracilivirgulaceae;g SLLW01;s
SRR9330141MAG086	Yes	70.72	5.61	758989	161	5300	45.4	91.48	951	d Archaea;p Nanoarchaeota;c Nanoarchaeia;o Woesearchaeales;f JAFGUC01;g ;s
SRR9330141MAG087	Yes	76.58	1.32	1910193	301	8128	46.4	88.55	1922	d Bacteria;p Firmicutes F;c Halanaerobiia;o Halanaerobiales;f Halarsenatibacteraceae;g Halarsenatibacter;s
SRR9330141MAG088	No	84.97	6.54	862289	161	6296	39.8	91.72	1216	d Archaea;p Nanohaloarchaeota;c Nanosalinia;o Nanosalinales;f Nanosalinaceae;g B1-Br10-U2g21;s B1-Br10-U2g21 sp001564145
SRR9330141MAG089	No	94.32	5.69	2905615	187	27924	64.6	88.31	3036	d Archaea;p Halobacteriota;c Halobacteria;o Halobacteriales;f Haloferacaceae;g Halorubrum;s Halorubrum sp001564205
SRR9330141MAG100	Yes	86.27	4.82	2139203	270	13697	45.1	84.07	1995	d Bacteria;p Firmicutes F;c Halanaerobiia;o Halanaerobiales;f Halarsenatibacteraceae;g T1SED10-84;s
SRR9330141MAG150	No	82.62	7.43	2715395	286	12661	40.8	85.29	2461	d Bacteria;p Firmicutes F;c Halanaerobiia;o Halanaerobiales;f CSSED10-376;g T1SED10-99;s T1SED10-99 sp003553225
SRR9330142MAG001	Yes	94.18	1.29	2647878	64	107746	63.1	90.46	2395	d Bacteria;p Proteobacteria;c Gammaproteobacteria;o SLND01;f SLND01;g SLND01;s SLND01 sp007133205
SRR9330142MAG002	Yes	95.23	3.09	2868472	157	34934	62.6	89.18	2929	d Archaea;p Halobacteriota;c Halobacteria;o Halobacteriales;f Haloferacaceae;g Halalkalirubrum;s
SRR9330142MAG003	Yes	98.4	0.8	1924534	130	27396	42.6	90.42	2020	d Archaea;p Thermoplasmatota;c Thermoplasmata;o PWKY01;f PWKY01;g Natronoplasma;s
SRR9330142MAG004	Yes	96.29	0.85	3294205	150	51649	66.8	90.38	3127	d Bacteria;p Proteobacteria;c Gammaproteobacteria;o XJ16;f Halofilaceae;g SLKC01;s SLKC01 sp007134775
SRR9330142MAG005	No	98.2	1.71	2691654	117	47642	74.1	90.71	2526	d Bacteria;p Actinobacteriota;c Actinomycetia;o Euzebyales;f Egibacteraceae;g PUKE01;s PUKE01 sp003553565
SRR9330142MAG006	Yes	86.13	1.88	2446749	126	44518	68.3	83.76	2334	d Archaea;p Halobacteriota;c Halobacteria;o Halobacteriales;f Natrialbaceae;g ;s
SRR9330142MAG007	Yes	98.6	5.45	3924866	177	49300	65	87.88	3972	d Archaea;p Halobacteriota;c Halobacteria;o Halobacteriales;f SKNY01;g SKNY01;s
SRR9330142MAG008	Yes	98.12	3.63	3159089	111	41282	63.1	89.06	3277	d Archaea;p Halobacteriota;c Halobacteria;o Halobacteriales;f Haloarculaceae;g Natronomonas;s
SRR9330142MAG009	Yes	93.46	5.88	2277289	295	12733	59.8	92	2675	d Archaea;p Halobacteriota;c Halobacteria;o JAHENH01;f ;g ;s
SRR9330142MAG010	No	86.34	2.19	3110852	257	26736	44.7	86.32	2824	d Bacteria;p Bacteroidota;c Rhodothermia;o Balneolales;f Natronogracilivirgulaceae;g ;s
SRR9330142MAG011	Yes	83.61	2.46	3022545	205	23204	44	86.59	2729	d Bacteria;p Bacteroidota;c Rhodothermia;o Balneolales;f Natronogracilivirgulaceae;g ;s
SRR9330142MAG012	Yes	91.95	0.9	2021501	56	67842	59.4	89.63	2140	d Archaea;p Halobacteriota;c Halobacteria;o Halobacteriales;f SKSH01;g SKSH01;s
SRR9330142MAG013	Yes	95.21	3.66	3390417	189	29684	65.2	91.33	3535	d Bacteria;p Chloroflexota;c Dehalococcoidia;o Tepidiformales;f Tepidiformaceae;g SLAK01;s SLAK01 sp007118095
SRR9330142MAG014	No	92.66	0.85	3191199	268	25744	62.7	88.29	2942	d Bacteria;p Bacteroidota;c Rhodothermia;o Rhodothermales;f Salinibacteraceae;g Tc-Br11-B2g6-7;s Tc-Br11-B2g6-7 sp001564055
SRR9330142MAG015	Yes	92.78	1.31	1901029	69	124065	58.9	91.06	2021	d Archaea;p Halobacteriota;c Halobacteria;o Halobacteriales;f SKSH01;g SKSH01;s
SRR9330142MAG017	No	83.59	4.39	2620080	213	19397	61.3	90.89	2785	d Archaea;p Halobacteriota;c Halobacteria;o Halobacteriales;f Haloarculaceae;g Halovenus;s
SRR9330142MAG018	Yes	91.3	4.96	2568695	224	18372	64.7	89.31	2742	d Archaea;p Halobacteriota;c Halobacteria;o Halobacteriales;f Haloferacaceae;g Halalkaliarchaeum;s
SRR9330142MAG019	Yes	90.27	2.13	2979192	369	11545	60.6	92.76	3061	d Bacteria;p Proteobacteria;c Gammaproteobacteria;o Pseudomonadales;f Oleiphilaceae;g Halospina;s
SRR9330142MAG020	Yes	80.84	0.93	840902	33	36249	34.9	92.39	1009	d Archaea;p Nanoarchaeota;c Nanoarchaeia;o Woesearchaeales;f 21-14-0-10-32-9;g SKIS01;s
SRR9330142MAG021	No	88.52	9.04	3130020	249	16142	67.7	87.06	3271	d Archaea;p Halobacteriota;c Halobacteria;o Halobacteriales;f Natrialbaceae;g Tc-Br11-E2g8;s Tc-Br11-E2g8 sp001564115
SRR9330142MAG022	No	90.82	5.23	2244731	95	58296	57.6	90.04	2380	d Archaea;p Halobacteriota;c Halobacteria;o Halobacteriales;f SKSH01;g SKSH01;s SKSH01 sp007118145
SRR9330142MAG024	Yes	93.44	2.24	3059448	675	5808	40.2	88.37	3080	d Bacteria;p Bacteroidota;c Bacteroidia;o CAILMK01;f ;g ;s
SRR9330142MAG025	Yes	91.62	1.7	1961246	88	45858	54.3	90.21	2142	d Archaea;p Halobacteriota;c Halobacteria;o Halobacteriales;f SKNY01;g SKNY01;s
SRR9330142MAG026	Yes	92.71	3.17	2511823	130	29990	64.3	87.39	2683	d Archaea;p Halobacteriota;c Halobacteria;o Halobacteriales;f Haloarculaceae;g Natronomonas;s
SRR9330142MAG027	No	88.07	6.93	2409499	303	12534	61.6	89.65	2773	d Archaea;p Halobacteriota;c Halobacteria;o Halobacteriales;f Haloferacaceae;g Halohasta;s
SRR9330142MAG028	Yes	95.74	2.54	3076510	199	29578	53.5	85.79	3198	d Archaea;p Halobacteriota;c Halobacteria;o Halobacteriales;f SKNY01;g SKNY01;s
SRR9330142MAG029	Yes	96.45	3.95	3850105	318	22802	57.5	87.94	4174	d Archaea;p Halobacteriota;c Halobacteria;o Halobacteriales;f Haloarculaceae;g Halovenus;s
SRR9330142MAG030	Yes	94.47	2.35	2858213	39	154224	62.4	91.48	2578	d Bacteria;p Proteobacteria;c Gammaproteobacteria;o SLND01;f SLND01;g SLND01;s
SRR9330142MAG031	Yes	92.42	3.83	2900371	242	17379	56.9	87.37	2935	d Archaea;p Halobacteriota;c Halobacteria;o Halobacteriales;f Haloarculaceae;g Halovenus;s
SRR9330142MAG032	No	93.04	2.8	2922261	128	39254	64.2	84.85	2907	d Archaea;p Halobacteriota;c Halobacteria;o Halobacteriales;f Natrialbaceae;g Tc-Br11;s Tc-Br11 sp001564275
SRR9330142MAG033	Yes	84.82	6.16	5223887	558	14525	60.8	86.81	4299	d Bacteria;p Myxococcota;c Bradymonadia;o Bradymonadales;f Bradymonadaceae;g SLJM01;s
SRR9330142MAG035	No	89.51	3.16	1845594	153	17317	61.9	87.84	2018	d Archaea;p Halobacteriota;c Halobacteria;o Halobacteriales;f Haloferacaceae;g PL-Br10-E2g29;s
SRR9330142MAG036	Yes	83.45	3.03	5771356	585	14452	62	87.48	4679	d Bacteria;p Myxococcota;c Bradymonadia;o Bradymonadales;f Bradymonadaceae;g SLJM01;s SLJM01 sp007120915

SRR9330142MAG037	Yes	79.33	5.31	1783484	361	5847	51.6	90.1	2082	d Archaea;p Halobacteriota;c Halobacteria;o Halobacteriales;f SKNY01;g SKNY01;s
SRR9330142MAG039	No	80.61	3.19	2271492	501	5304	55.5	89.9	2675	d Archaea;p Halobacteriota;c Halobacteria;o Halobacteriales;f Haloarculaceae;g Halovenus;s Halovenus sp001564135
SRR9330142MAG042	Yes	92.31	2.14	3458479	94	63809	72.8	89.2	3193	d Bacteria;p Actinobacteriota;c Actinomycetia;o Nitriliruptorales;f Nitriliruptoraceae;g SKLC01;s
SRR9330142MAG044	No	71.89	4.24	1440249	291	6252	35.9	91.96	1589	d Bacteria;p Firmicutes F;c Halanaerobiia;o Halanaerobiales;f Halarsenatibacteraceae;g SLSL01;s SLSL01 sp003552825
SRR9330142MAG046	No	76.55	5.36	1855157	758	2810	38.5	89.56	2118	d Bacteria;p Proteobacteria;c Gammaproteobacteria;o UBA12402;f UBA12402;g ;s
SRR9330142MAG047	No	88.17	7.18	3067637	410	15169	59.8	90.66	3392	d Archaea;p Halobacteriota;c Halobacteria;o Halobacteriales;f Haloarculaceae;g Halovenus;s Halovenus sp003551265
SRR9330142MAG048	Yes	79.97	2.96	2112352	284	11609	65.1	92.66	2403	d Archaea;p Halobacteriota;c Halobacteria;o Halobacteriales;f Natrialbaceae;g ;s
SRR9330142MAG054	No	85.32	7.12	2426956	248	12506	65	87.85	2721	d Archaea;p Halobacteriota;c Halobacteria;o Halobacteriales;f Haloarculaceae;g Natronomonas;s
SRR9330142MAG057	No	82.54	3.87	1914446	168	25325	62	88.96	2129	d Archaea;p Halobacteriota;c Halobacteria;o Halobacteriales;f Haloferacaceae;g PL-Br10-E2g29;s PL-Br10-E2g29 sp001563965
SRR9330142MAG058	Yes	78.16	2.94	2844437	705	4662	62.4	87.05	2925	d Bacteria;p Bacteroidota;c Rhodothermia;o Rhodothermales;f Salinibacteraceae;g JAAAPH01;s
SRR9330142MAG061	Yes	81.61	7.69	2753049	528	6658	61.6	89.07	3119	d Archaea;p Halobacteriota;c Halobacteria;o Halobacteriales;f Haloarculaceae;g Halovenus;s
SRR9330142MAG066	Yes	83.33	9.94	3111501	265	24401	71.9	89.15	3078	d Bacteria;p Actinobacteriota;c Actinomycetia;o Nitriliruptorales;f Nitriliruptoraceae;g SKTG01;s
SRR9330142MAG067	Yes	76.31	1.71	2644022	251	12919	73.9	90.61	2600	d Bacteria;p Actinobacteriota;c Actinomycetia;o Euzebyales;f Egibacteraceae;g Egibacter;s
SRR9330142MAG071	Yes	79.76	9.3	2402986	579	4653	54.7	88.51	2934	d Archaea;p Halobacteriota;c Halobacteria;o Halobacteriales;f SKNY01;g SKNY01;s
SRR9330143MAG001	No	95.88	2.67	1300079	37	58091	46.7	92.54	1296	d Bacteria;p Firmicutes;c Bacilli;o Izemoplasmatales;f Izemoplasmataceae;g B1SED10-225;s
SRR9330143MAG002	Yes	96.61	2.23	2849668	167	35388	38.4	86.86	2746	d Bacteria;p Firmicutes D;c Natranaerobiia;o Natranaerobiales;f ;g ;s
SRR9330143MAG003	Yes	99.4	0.6	2502713	135	27895	51.5	85.03	2343	d Bacteria;p Desulfobacterota I;c Desulfovibrionia;o Desulfovibrionales;f Desulfonatronovibrionaceae;g Desulfonatronospira;s
SRR9330143MAG004	Yes	95.16	2.15	2433876	175	22859	47.2	88.86	2098	d Bacteria;p Bacteroidota;c Bacteroidia;o Bacteroidales;f UBA7960;g PYGY01;s
SRR9330143MAG005	No	98.67	0	1303409	17	118075	53	92.36	1284	d Bacteria;p Firmicutes;c Bacilli;o Izemoplasmatales;f Izemoplasmataceae;g T1SED10-81;s T1SED10-81 sp003553945
SRR9330143MAG006	No	90.44	1.64	3244073	157	39745	44.7	86.43	2790	d Bacteria;p Bacteroidota;c Rhodothermia;o Balneolales;f Natronogracilivirgulaceae;g ;s
SRR9330143MAG007	Yes	98.67	0.67	1408927	75	39939	42.3	92.26	1446	d Bacteria;p Firmicutes;c Bacilli;o Izemoplasmatales;f Izemoplasmataceae;g B1SED10-225;s B1SED10-225 sp003551405
SRR9330143MAG008	Yes	96.85	3.27	3055351	248	22337	68.5	87.76	3166	d Archaea;p Halobacteriota;c Halobacteria;o Halobacteriales;f Natrialbaceae;g Tc-Br11-E2g8;s Tc-Br11-E2g8 sp001564115
SRR9330143MAG009	No	95.6	3.2	1970612	113	31530	42.7	91.02	2003	d Archaea;p Thermoplasmatota;c Thermoplasmata;o PWKY01;f PWKY01;g Natronoplasma;s
SRR9330143MAG010	No	97.35	1.91	2754907	216	20123	55.3	89.77	2939	d Archaea;p Halobacteriota;c Halobacteria;o Halobacteriales;f Haloarculaceae;g Halovenus;s Halovenus sp001564135
SRR9330143MAG011	Yes	98.71	3.03	2922229	189	24150	54.5	87.56	2628	d Bacteria;p Desulfobacterota;c Desulfobacteria;o Desulfobacterales;f SURF-3;g B1SED10-16;s B1SED10-16 sp003551985
SRR9330143MAG012	No	98.29	2.28	2662464	85	68379	74.1	90.75	2456	d Bacteria;p Actinobacteriota;c Actinomycetia;o Euzebyales;f Egibacteraceae;g PUKE01;s PUKE01 sp003553565
SRR9330143MAG013	No	97.44	1.28	3430831	116	55905	71.4	90.36	3160	d Bacteria;p Actinobacteriota;c Actinomycetia;o Nitriliruptorales;f Nitriliruptoraceae;g PWLR01;s
SRR9330143MAG014	No	89.14	6.14	3204837	293	26581	66.2	88.53	3201	d Bacteria;p Proteobacteria;c Gammaproteobacteria;o Ectothiorhodospirales;f Thioalkalivibrionaceae;g Thioalkalivibrio B;s Thioalkalivibrio B sp003563455
SRR9330143MAG016	No	94.92	8.19	3618530	378	21046	62.7	88.19	3334	d Bacteria;p Bacteroidota;c Rhodothermia;o Rhodothermales;f Salinibacteraceae;g Tc-Br11-B2g6-7;s Tc-Br11-B2g6-7 sp001564055
SRR9330143MAG017	No	82.24	0	918543	18	71571	38.8	91.73	989	d Archaea;p Nanohaloarchaeota;c Nanosalinia;o Nanosalinales;f ;g ;s
SRR9330143MAG018	No	96.11	2.38	2852095	296	13014	51.8	90.1	3175	d Archaea;p Halobacteriota;c Halobacteria;o Halobacteriales;f Haloferacaceae;g Halalkalirubrum;s Halalkalirubrum sp003551725
SRR9330143MAG019	No	90.79	0.44	2307198	52	91786	40.4	90.73	2138	d Bacteria;p Firmicutes F;c Halanaerobiia;o Halanaerobiales;f Halarsenatibacteraceae;g T1SED10-84;s T1SED10-84 sp003553485
SRR9330143MAG021	Yes	76.5	7.74	2169502	486	5633	43.8	89.33	2289	d Bacteria;p Bacteroidota;c Rhodothermia;o Balneolales;f Balneolaceae;g SW132;s
SRR9330143MAG022	Yes	98.09	4.64	3986253	212	70267	41.8	88.23	3545	d Bacteria;p Bacteroidota;c Rhodothermia;o Balneolales;f Balneolaceae;g ;s
SRR9330143MAG023	No	95.18	1.85	2599381	180	23974	39.6	86.93	2306	d Bacteria;p Firmicutes F;c Halanaerobiia;o Halanaerobiales;f CSSED10-376;g T1SED10-99;s T1SED10-99 sp003553225
SRR9330143MAG024	No	98.28	2.6	3454056	176	34330	65.5	90.48	3286	d Bacteria;p Proteobacteria;c Gammaproteobacteria;o Pseudomonadales;f Halomonadaceae;g Halomonas;s
SRR9330143MAG025	Yes	96.77	8.62	3729427	445	13316	35.7	85.48	3244	d Bacteria;p Bacteroidota;c Bacteroidia;o Bacteroidales;f UBA7960;g PUKZ01;s
SRR9330143MAG027	Yes	91.27	4.38	3203513	557	7597	55.7	86.53	3125	d Bacteria;p Desulfobacterota;c Desulfobacteria;o Desulfobacterales;f SURF-3;g B1SED10-16;s
SRR9330143MAG028	Yes	95.1	3.85	3000788	231	25904	33	87.08	2870	d Bacteria;p Firmicutes A;c Clostridia;o Acetivibrionales;f ;g ;s
SRR9330143MAG029	Yes	97.62	2.82	2354893	135	32125	68.5	92.54	2326	d Bacteria;p Proteobacteria;c Gammaproteobacteria;o Thiohalospirales;f Thiohalospiraceae;g Thiohalospira;s
SRR9330143MAG030	Yes	86.21	3.23	4538667	223	43962	61.8	91.1	3734	d Bacteria;p Myxococcota;c Bradymonadia;o Bradymonadales;f Bradymonadaceae;g SLJM01;s
SRR9330143MAG031	No	79.6	3.45	648772	28	43437	37.3	92.63	717	d Bacteria;p Patescibacteria;c Paceibacteria;o Paceibacterales;f PWPS01;g PWPS01;s PWPS01 sp003554445
SRR9330143MAG032	Yes	81.32	1.72	886995	114	10598	46.2	83.53	898	d Bacteria;p Patescibacteria;c Paceibacteria;o UBA9983 A;f CSBR16-193;g ;s
SRR9330143MAG034	No	97.53	1.84	3121190	112	42043	64	84.98	3073	d Archaea;p Halobacteriota;c Halobacteria;o Halobacteriales;f Natrialbaceae;g Tc-Br11;s Tc-Br11 sp001564275
SRR9330143MAG035	No	85.19	2.8	619506	81	9941	46	92.64	767	d Archaea;p Aenigmatarchaeota;c Aenigmatarchaeia;o PWEA01;f PWEA01;g PWEA01;s PWEA01 sp003555465
SRR9330143MAG036	Yes	70.87	0.1	768187	148	5908	38.6	89.67	854	d Bacteria;p Patescibacteria;c Paceibacteria;o SW-4-49-11;f SW-4-49-11;g ;s
SRR9330143MAG040	No	90.31	9.3	1878025	325	8437	64.6	94.83	2049	d Bacteria;p Proteobacteria;c Gammaproteobacteria;o Nitrococcales;f Nitrococcaceae;g Spiribacter;s Spiribacter sp009676705
SRR9330143MAG041	No	96.93	6.36	3162302	232	25095	41.4	86.08	3026	d Bacteria;p Firmicutes F;c Halanaerobiia;o Halanaerobiales;f Halarsenatibacteraceae;g Halarsenatibacter;s
SRR9330143MAG042	No	87.38	2.8	709935	21	51130	36	89.2	816	d Archaea;p B1Sed10-29;c B1Sed10-29;o B1Sed10-29;f B1Sed10-29;g B1Sed10-29;s
SRR9330143MAG043	No	75.81	0.31	868695	121	9764	33.6	91.25	993	d Archaea;p Nanoarchaeota;c Nanoarchaeia;o Woesearchaeales;f 21-14-0-10-32-9;g ;s
SRR9330143MAG044	Yes	83.94	3.98	2263332	253	11596	48.6	86.28	2158	d Bacteria;p Firmicutes D;c Dethiobacteria;o DTU022;f PWGO01;g PWGO01;s
SRR9330143MAG045	No	73.34	2.25	710946	67	23984	30.8	91.97	743	d Bacteria;p Patescibacteria;c ABY1;o BM507;f UBA12465;g PWHG01;s PWHG01 sp003554785
SRR9330143MAG046	No	89.38	2.64	3591292	785	5462	57.5	86.09	3589	d Bacteria;p Bacteroidota;c Rhodothermia;o Rhodothermales;f Salinibacteraceae;g Longimonas;s
SRR9330143MAG047	Yes	98.68	9.5	2794466	390	14059	35.8	90.05	2957	d Bacteria;p Firmicutes F;c Halanaerobiia;o Halanaerobiales;f Halarsenatibacteraceae;g SLSL01;s SLSL01 sp007130465
SRR9330143MAG049	Yes	82.84	0.5	2201583	591	4469	48.6	84.66	2447	d Bacteria;p Firmicutes E;c SLMV01;o SLMV01;f PUMD01;g PUMD01;s
SRR9330143MAG050	No	93.28	1.71	3781610	567	11700	71	88.59	3830	d Bacteria;p Actinobacteriota;c Actinomycetia;o Nitriliruptorales;f Nitriliruptoraceae;g T1Sed10-7;s
SRR9330143MAG051	Yes	79.62	7.34	2205361	404	8593	68.2	93.29	2394	d Bacteria;p Proteobacteria;c Gammaproteobacteria;o Ectothiorhodospirales;f Thioalkalivibrionaceae;g Thioalkalivibrio;s Thioalkalivibrio halophilus
SRR9330143MAG052	No	89.09	5.29	3301039	612	6875	64.4	90.91	3609	d Bacteria;p Proteobacteria;c Alphaproteobacteria;o Rhodobacterales;f Rhodobacteraceae;g Roseinatronobacter;s Roseinatronobacter sp003561595
SRR9330143MAG053	No	74.77	3.12	579824	132	4987	38.5	89.08	763	d Archaea;p B1Sed10-29;c B1Sed10-29;o B1Sed10-29;f B1Sed10-29;g B1Sed10-29;s B1Sed10-29 sp003553825
SRR9330143MAG054	No	81.78	9.13	780868	115	27771	43.6	91.83	977	d Archaea;p Aenigmatarchaeota;c Aenigmatarchaeia;o PWEA01;f PWEA01;g PWEA01;s PWEA01 sp003554235
SRR9330143MAG056	No	91.85	6.78	2483263	222	17389	66.2	89.06	2625	d Archaea;p Halobacteriota;c Halobacteria;o Halobacteriales;f Haloferacaceae;g Halorubrum;s Halorubrum sp003554605
SRR9330143MAG058	No	94.2	7.5	2923045	133	37264	67.5	87.77	3004	d Archaea;p Halobacteriota;c Halobacteria;o Halobacteriales;f Haloferacaceae;g Halorubrum;s
SRR9330143MAG060	No	74.7	5.69	3039968	814	4600	57.1	91.68	3345	d Bacteria;p Spirochaetota;c Spirochaetia;o DSM-27196;f SKKC01;g SLAN01;s
SRR9330143MAG063	Yes	73.25	8.55	2120763	522	4598	41.4	84.54	2302	d Bacteria;p Firmicutes D;c Natranaerobiia;o Natranaerobiales;f ;g ;s
SRR9330143MAG066	No	89.44	8.11	2732278	419	10549	49.8	91.25	2954	d Bacteria;p Firmicutes F;c Halanaerobiia;o Halanaerobiales;f Halarsenatibacteraceae;g Halarsenatibacter;s
SRR9330143MAG068	Yes	80.28	1.87	1419404	216	9037	34.3	87.76	1537	d Archaea;p Nanoarchaeota;c Nanoarchaeia;o Woesearchaeales;f DSVV01;g ;s
SRR9330143MAG070	No	70.1	1.33	908143	469	2096	49.5	93.26	1272	d Bacteria;p Firmicutes;c Bacilli;o Izemoplasmatales;f Izemoplasmataceae;g T1SED10-81;s T1SED10-81 sp003554025
SRR9330143MAG072	No	87.1	9.68	2713738	463	9739	61.5	89.32	3200	d Archaea;p Halobacteriota;c Halobacteria;o Halobacteriales;f Haloferacaceae;g Halohasta;s
SRR9330143MAG079	No	94.06	6.42	2381128	323	10227	71.6	92.78	2435	d Bacteria;p Proteobacteria;c Gammaproteobacteria;o Nitrococcales;f Halorhodospiraceae;g Halorhodospira;s
SRR9330143MAG089	No	71.1	2.9	4008918	512	10405	61	87.4	3364	d Bacteria;p Myxococcota;c Bradymonadia;o Bradymonadales;f Bradymonadaceae;g SLJM01;s
SRR9330143MAG093	No	70.85	0.24	1096563	187	7574	49.8	92.72	1203	d Bacteria;p Firmicutes;c Bacilli;o Izemoplasmatales;f Izemoplasmataceae;g B1SED10-225;s B1SED10-225 sp003554735
SRR9330143MAG107	No	77.91	4.87	2380953	458	6200	63.5	90	2742	d Archaea;p Halobacteriota;c Halobacteria;o Halobacteriales;f Haloarculaceae;g Natronomonas;s
SRR9330143MAG108	Yes	80.14	3.98	5102076	1048	6068	62.5	88.07	4557	d Bacteria;p Myxococcota;c Bradymonadia;o Bradymonadales;f Bradymonadaceae;g SLJM01;s
SRR9330143MAG120	No	77.35	0	1224538	221	6697	51.1	92.62	1328	d Bacteria;p Firmicutes;c Bacilli;o Izemoplasmatales;f Izemoplasmataceae;g M55B118;s
SRR9330144MAG001	Yes	100	0.89	2587913	186	22538	49.1	85.42	2532	d Bacteria;p Desulfobacterota I;c Desulfovibrionia;o Desulfovibrionales;f Desulfonatronovibrionaceae;g Desulfonatronovibrio;s
SRR9330144MAG002	Yes	98.99	2.16	2739360	132	38742	57.3	92.09	2631	d Bacteria;p Proteobacteria;c Gammaproteobacteria;o Pseudomonadales;f Oleiphilaceae;g Marinobacter;s
SRR9330144MAG003	No	92.2	2.23	3030352	232	25457	55.5	90.72	3052	d Bacteria;p Proteobacteria;c Gammaproteobacteria;o Competibacterales;f Competibacteraceae;g SKOM01;s SKOM01 sp007120095
SRR9330144MAG004	No	95.2	7.06	4759177	566	13880	61.2	87.55	4479	d Bacteria;p Bacteroidota;c Rhodothermia;o Rhodothermales;f Salinibacteraceae;g Tc-Br11-B2g6-7;s Tc-Br11-B2g6-7 sp001564055
SRR9330144MAG005	Yes	94.76	4.08	4288972	343	26009	59	91.53	3539	d Bacteria;p Verrucomicrobiota;c Kiritimatiellae;o SS1-B-03-39;f UBA6053;g PXAS01;s
SRR9330144MAG006	No	92.78	3.72	2863321	189	25379	65.6	93.63	2818	d Bacteria;p Proteobacteria;c Gammaproteobacteria;o XN24;f XN24;g XN24;s
SRR9330144MAG007	No	91.41	1.93	2730807	184	23818	66.5	88.62	2632	d Bacteria;p Proteobacteria;c Gammaproteobacteria;o Ectothiorhodospirales;f Thioalkalivibrionaceae;g Thioalkalivibrio B;s Thioalkalivibrio B sp003563455
SRR9330144MAG008	Yes	85.25	1.37	3457991	299	18639	51.8	89.37	2898	d Bacteria;p Bacteroidota;c Rhodothermia;o Balneolales;f PXAI01;g RECM01;s
SRR9330144MAG009	Yes	98.1	1.18	4221265	225	29374	52.1	87.62	3794	d Bacteria;p Cyanobacteria;c Cyanobacteriia;o Cyanobacteriales;f Geitlerinemaceae;g Phormidium A;s Phormidium A sp007126595
SRR9330144MAG010	Yes	95.52	1.7	4775965	295	23150	67.4	85.56	3667	d Bacteria;p Verrucomicrobiota;c Verrucomicrobiae;o Verrucomicrobiales;f SKLO01;g SKLO01;s SKLO01 sp007123195
SRR9330144MAG011	Yes	97.2	3.6	3853694	364	17802	70.9	88.71	3932	d Bacteria;p Proteobacteria;c Alphaproteobacteria;o Rhodobacterales;f Rhodobacteraceae;g SKOX01;s SKOX01 sp007119835
SRR9330144MAG012	No	94.7	3.42	3885466	228	38930	69.2	93.89	3868	d Bacteria;p Actinobacteriota;c Acidimicrobiia;o Acidimicrobiales;f Ilumatobacteraceae;g SKSP01;s SKSP01 sp007693335
SRR9330144MAG014	Yes	96.4	1.18	5699055	259	57098	65.2	89.15	4255	d Bacteria;p Planctomycetota;c Planctomycetia;o Pirellulales;f Pirellulaceae;g ;s
SRR9330144MAG015	Yes	93.49	1.2	6850973	719	14055	48.7	78.22	5546	d Bacteria;p Cyanobacteria;c Cyanobacteriia;o Cyanobacteriales;f Oscillatoriaceae;g Oscillatoria;s
SRR9330144MAG016	Yes	97.63	4.79	7942415	958	11985	60.1	87.27	6860	d Bacteria;p Planctomycetota;c Planctomycetia;o Pirellulales;f Pirellulaceae;g SKKK01;s
SRR9330144MAG017	Yes	90	3.21	3943663	387	13644	50.7	89.7	3596	d Bacteria;p Chloroflexota;c Anaerolineae;o Promineofilales;f Promineofilaceae;g SLGK01;s

SRR9330144MAG018	No	98.75	0	5294959	274	33098	61	89.26	3986	d Bacteria;p Planctomycetota;c Planctomycetia;o Pirellulales;f Pirellulaceae;g UBA6163;s UBA6163 sp007127095
SRR9330144MAG019	Yes	79.32	0.41	2623824	627	4998	62.5	90.01	2730	d Bacteria;p Planctomycetota;c Phycisphaerae;o Phycisphaerales;f SM1A02;g ;s
SRR9330144MAG020	Yes	93.58	2.74	3158761	456	9860	57.3	91.32	2913	d Bacteria;p Verrucomicrobiota;c Verrucomicrobiae;o Opitutales;f ;g ;s
SRR9330144MAG021	Yes	86.38	0.18	3026170	134	57410	63.7	89.54	2978	d Bacteria;p Proteobacteria;c Gammaproteobacteria;o Burkholderiales;f Rhodocyclaceae;g Azoarcus F;s
SRR9330144MAG022	Yes	72.57	0.23	2683134	239	19047	48.4	88.8	2613	d Bacteria;p Firmicutes A;c Clostridia;o Peptostreptococcales;f Tindalliaceae;g JAABSW01;s
SRR9330144MAG023	No	93.64	2.27	2674730	273	19681	44	89.97	2796	d Bacteria;p Firmicutes A;c Clostridia;o Peptostreptococcales;f T1SED10-28;g T1SED10-28;s T1SED10-28 sp003554105
SRR9330144MAG024	No	94.08	9.43	3867791	268	44493	59.9	91.21	3918	d Bacteria;p Proteobacteria;c Alphaproteobacteria;o Rhodobacterales;f Rhodobacteraceae;g Roseinatronobacter;s Roseinatronobacter monicus
SRR9330144MAG025	No	91.35	8.93	3198471	343	16753	65.5	87.59	3306	d Bacteria;p Proteobacteria;c Gammaproteobacteria;o Ectothiorhodospirales;f Thioalkalivibrionaceae;g Thioalkalivibrio B;s Thioalkalivibrio B sp007116035
SRR9330144MAG026	Yes	77.49	4.77	2965777	701	4834	73.2	90.12	3132	d Bacteria;p Proteobacteria;c Alphaproteobacteria;o Rhodobacterales;f Rhodobacteraceae;g ;s
SRR9330144MAG027	Yes	94.36	4.16	4894006	391	20410	68.2	89.51	4980	d Bacteria;p Proteobacteria;c Alphaproteobacteria;o Geminicoccales;f Geminicoccaceae;g SLRI01;s
SRR9330144MAG028	Yes	84.83	3.06	3844777	803	5688	58.1	83.3	4072	d Bacteria;p Cyanobacteria;c Cyanobacteriia;o Phormidesmiales;f Phormidesmiaceae;g Nodosilinea;s
SRR9330144MAG029	Yes	83.34	7.66	3549883	648	7357	53.8	90.46	3536	d Bacteria;p Verrucomicrobiota;c Kiritimatiellae;o UBA8416;f UBA8416;g T3Sed10-140;s
SRR9330144MAG030	Yes	94.87	4.4	5352835	1078	8269	49.7	85.1	4966	d Bacteria;p Planctomycetota;c Planctomycetia;o Pirellulales;f Pirellulaceae;g Mariniblastus;s
SRR9330144MAG032	Yes	86.23	7.35	4260952	751	8434	71	89.29	4550	d Bacteria;p Actinobacteriota;c Actinomycetia;o Nitriliruptorales;f Nitriliruptoraceae;g ;s
SRR9330144MAG034	No	84.68	5.53	2416104	375	8295	58.8	90.7	2431	d Bacteria;p Verrucomicrobiota;c Kiritimatiellae;o SLAD01;f SLAD01;g SLLF01;s SLLF01 sp003562335
SRR9330144MAG036	Yes	71.55	0.72	2419020	80	67184	61.6	92.39	2184	d Bacteria;p Proteobacteria;c Gammaproteobacteria;o Xanthomonadales;f Wenzhouxiangellaceae;g Wenzhouxiangella;s
SRR9330144MAG038	Yes	75.8	2.96	4928910	1239	4579	58.5	88.33	4574	d Bacteria;p Bacteroidota;c Bacteroidia;o Chitinophagales;f Saprospiraceae;g ;s
SRR9330144MAG039	Yes	81.42	10	2616192	391	9112	54.5	91.95	2441	d Bacteria;p Verrucomicrobiota;c Kiritimatiellae;o UBA8416;f PXDI01;g PXDI01;s PXDI01 sp003565095
SRR9330144MAG040	No	80.37	0.51	1905361	881	2391	60	91.11	2658	d Archaea;p Halobacteriota;c Halobacteria;o Halobacteriales;f SKSH01;g SKSH01;s
SRR9330144MAG041	Yes	81.17	3.75	2932240	655	5424	38.2	87.02	2902	d Bacteria;p Bacteroidota;c Bacteroidia;o Chitinophagales;f Saprospiraceae;g PWJY01;s
SRR9330144MAG043	No	84.48	7.37	3898423	504	13914	69.5	87.55	4155	d Bacteria;p Actinobacteriota;c Actinomycetia;o Nitriliruptorales;f Nitriliruptoraceae;g CSSed11-175R1;s CSSed11-175R1 sp007136095
SRR9330144MAG045	Yes	88.79	5.41	1599423	415	5729	38.1	94.63	1971	d Bacteria;p Campylobacterota;c Campylobacteria;o Campylobacterales;f Sulfurovaceae;g TCS-49;s
SRR9330144MAG046	Yes	84.62	8.57	3043180	690	5229	65.3	89.56	3001	d Bacteria;p Verrucomicrobiota;c Kiritimatiellae;o SLAD01;f SLAD01;g ;s
SRR9330144MAG047	Yes	72.04	1.72	2239121	457	6153	54.1	91.72	2436	d Bacteria;p Proteobacteria;c Gammaproteobacteria;o Competibacterales;f Competibacteraceae;g SKOM01;s
SRR9330144MAG054	Yes	91.22	7.99	4127063	553	11635	67.4	91.12	4458	d Bacteria;p Proteobacteria;c Alphaproteobacteria;o Rhodobacterales;f Rhodobacteraceae;g UBA996;s
SRR9330144MAG058	Yes	96.55	5.8	3404388	188	36649	72.6	92.39	3262	d Bacteria;p Deinococcota;c Deinococci;o Deinococcales;f Trueperaceae;g JAABTL01;s
SRR9330144MAG060	No	89.07	1.91	3717346	203	39402	43.6	87.37	3171	d Bacteria;p Bacteroidota;c Rhodothermia;o Balneolales;f Balneolaceae;g UBA2664;s UBA2664 sp007123715
SRR9330144MAG066	Yes	92.49	2.68	3617257	552	8665	59.2	87.44	3603	d Bacteria;p Desulfobacterota I;c Desulfovibrionia;o Desulfovibrionales;f Desulfonatronaceae;g Desulfonatronum;s Desulfonatronum sp007127655
SRR9330144MAG067	Yes	74.88	4.43	2108153	604	3710	52.9	93.18	2457	d Bacteria;p Proteobacteria;c Gammaproteobacteria;o Pseudomonadales;f Halomonadaceae;g Marinospirillum;s
SRR9330144MAG071	No	79.51	4.64	2661907	221	25453	55	89.74	2305	d Bacteria;p Bacteroidota;c Rhodothermia;o Balneolales;f Natronogracilivirgulaceae;g SKNL01;s
SRR9330144MAG075	Yes	89.31	7.24	4030686	864	6638	69.8	91.88	3942	d Bacteria;p Gemmatimonadota;c Gemmatimonadetes;o Longimicrobiales;f UBA6960;g SKNW01;s SKNW01 sp007120305
SRR9330144MAG093	Yes	84.96	9.89	3766529	322	20231	71.3	91.74	3704	d Bacteria;p Deinococcota;c Deinococci;o Deinococcales;f Trueperaceae;g CSSed10-48;s
SRR9330145MAG001	No	95.48	0.56	3276079	192	27497	62.6	87.81	2942	d Bacteria;p Bacteroidota;c Rhodothermia;o Rhodothermales;f Salinibacteraceae;g Tc-Br11-B2g6-7;s Tc-Br11-B2g6-7 sp001564055
SRR9330145MAG002	Yes	92.4	3.13	2521984	177	26148	47.2	84.84	2576	d Archaea;p Halobacteriota;c Halobacteria;o Halobacteriales;f Salinarchaeaceae;g Salinarchaeum;s
SRR9330145MAG003	Yes	80.84	0	1044093	95	15418	41.5	92.11	1153	d Archaea;p Nanohaloarchaeota;c Nanosalinia;o Nanosalinales;f ;g ;s
SRR9330145MAG004	No	80.78	3.04	1763914	82	37944	62.2	88.88	1856	d Archaea;p Halobacteriota;c Halobacteria;o Halobacteriales;f Haloferacaceae;g PL-Br10-E2g29;s PL-Br10-E2g29 sp001563965
SRR9330145MAG005	No	88.85	6.13	1780793	232	10839	64.7	95.41	1899	d Bacteria;p Proteobacteria;c Gammaproteobacteria;o Nitrococcales;f Nitrococcaceae;g Spiribacter;s Spiribacter sp009676705
SRR9330145MAG006	No	89.97	8.53	1956108	149	19141	61.9	88.01	2142	d Archaea;p Halobacteriota;c Halobacteria;o Halobacteriales;f Haloferacaceae;g PL-Br10-E2g29;s
SRR9330145MAG008	Yes	96.83	5.73	3524580	235	25610	66.1	85.3	3659	d Archaea;p Halobacteriota;c Halobacteria;o Halobacteriales;f Natrialbaceae;g ;s
SRR9330145MAG009	No	90.15	9.4	2518902	152	25337	64.3	87.37	2676	d Archaea;p Halobacteriota;c Halobacteria;o Halobacteriales;f Haloarculaceae;g Natronomonas;s
SRR9330145MAG010	No	84.19	3.08	2200786	174	20306	66.6	89.4	2327	d Archaea;p Halobacteriota;c Halobacteria;o Halobacteriales;f Haloferacaceae;g Halorubrum;s Halorubrum sp003554605
SRR9330145MAG011	Yes	80.71	3.56	2368581	129	28951	67.2	85.87	2384	d Archaea;p Halobacteriota;c Halobacteria;o Halobacteriales;f Haloferacaceae;g Halorubrum;s
SRR9330145MAG012	Yes	77.06	2.34	1915501	504	4448	64.7	91.41	2446	d Archaea;p Halobacteriota;c Halobacteria;o Halobacteriales;f Haloarculaceae;g Natronomonas;s Natronomonas pharaonis
SRR9330145MAG014	No	74.05	8.36	2317370	266	12603	65	87.46	2511	d Archaea;p Halobacteriota;c Halobacteria;o Halobacteriales;f Haloferacaceae;g Halorubrum;s
SRR9330145MAG020	Yes	71.44	6.03	2067109	542	4355	68.2	87.9	2457	d Archaea;p Halobacteriota;c Halobacteria;o Halobacteriales;f Haloferacaceae;g Halorubrum;s
SRR9330146MAG001	No	98.67	0	1411655	59	57931	40.2	92.34	1411	d Bacteria;p Firmicutes;c Bacilli;o Izemoplasmatales;f Izemoplasmataceae;g B1SED10-225;s B1SED10-225 sp003558105
SRR9330146MAG002	Yes	98.12	0.54	2499447	104	43887	44.9	91.39	2211	d Bacteria;p Bacteroidota;c Bacteroidia;o Flavobacteriales;f Schleiferiaceae;g CSBr16-58;s CSBr16-58 sp003566715
SRR9330146MAG003	No	91.96	1.89	3212605	237	22549	55.4	90.98	3235	d Bacteria;p Proteobacteria;c Gammaproteobacteria;o Competibacterales;f Competibacteraceae;g SKOM01;s SKOM01 sp007120095
SRR9330146MAG004	Yes	97.39	6.08	2677744	347	18783	47.5	92.06	2620	d Bacteria;p Proteobacteria;c Gammaproteobacteria;o Enterobacterales;f Alteromonadaceae;g Aliidiomarina;s
SRR9330146MAG005	No	93.28	2.53	2539776	242	15535	48.2	90.86	2131	d Bacteria;p Bacteroidota;c Bacteroidia;o Bacteroidales;f UBA7960;g PUPG01;s PUPG01 sp007124575
SRR9330146MAG006	No	91.88	5.52	1808745	199	20539	64.6	95.12	1921	d Bacteria;p Proteobacteria;c Gammaproteobacteria;o Nitrococcales;f Nitrococcaceae;g Spiribacter;s Spiribacter sp009676705
SRR9330146MAG007	No	98.15	2.88	2978594	201	31312	66.4	88.36	2899	d Bacteria;p Proteobacteria;c Gammaproteobacteria;o Ectothiorhodospirales;f Thioalkalivibrionaceae;g Thioalkalivibrio B;s Thioalkalivibrio B sp003563455
SRR9330146MAG008	No	98.43	4.07	4559403	291	29680	55.3	90.3	4326	d Bacteria;p Proteobacteria;c Gammaproteobacteria;o Pseudomonadales;f Natronospirillaceae;g ;s
SRR9330146MAG009	No	95.76	1.98	3722457	345	24822	62.3	87.74	3448	d Bacteria;p Bacteroidota;c Rhodothermia;o Rhodothermales;f Salinibacteraceae;g Tc-Br11-B2g6-7;s Tc-Br11-B2g6-7 sp001564055
SRR9330146MAG010	Yes	98.33	5.38	3352048	240	24728	65.5	87.5	3366	d Bacteria;p Proteobacteria;c Gammaproteobacteria;o Ectothiorhodospirales;f Thioalkalivibrionaceae;g Thioalkalivibrio B;s Thioalkalivibrio B sp007116035
SRR9330146MAG011	Yes	99.2	0.8	3086882	148	56880	51.4	94.39	2846	d Bacteria;p Spirochaetota;c Spirochaetia;o DSM-27196;f DSM-8902;g Spirochaeta B;s
SRR9330146MAG012	Yes	93.64	0	4053404	178	34560	54.4	80.9	3223	d Bacteria;p Deinococcota;c Deinococci;o Deinococcales;f Trueperaceae;g ;s
SRR9330146MAG013	No	95.49	2.46	3480005	203	32570	67.8	89.26	3307	d Bacteria;p Proteobacteria;c Alphaproteobacteria;o Rhodobacterales;f Rhodobacteraceae;g PUOA01;s PUOA01 sp007121115
SRR9330146MAG014	No	92.06	9.68	2190727	451	8639	58.8	95.47	2312	d Bacteria;p Proteobacteria;c Gammaproteobacteria;o Xanthomonadales;f Wenzhouxiangellaceae;g Wenzhouxiangella;s
SRR9330146MAG015	No	89.83	0.82	1813421	117	46116	55.1	94.93	1861	d Bacteria;p Proteobacteria;c Gammaproteobacteria;o Nitrococcales;f Nitrococcaceae;g Spiribacter;s
SRR9330146MAG016	No	95.22	1.18	5635410	274	37797	65.2	88.96	4220	d Bacteria;p Planctomycetota;c Planctomycetia;o Pirellulales;f Pirellulaceae;g ;s
SRR9330146MAG017	Yes	90.98	9.43	3639433	529	9262	46.2	86.01	3372	d Bacteria;p Bacteroidota;c Rhodothermia;o Balneolales;f Balneolaceae;g SW132;s
SRR9330146MAG018	Yes	94.3	4.69	2987577	151	47018	67.6	87.92	3074	d Archaea;p Halobacteriota;c Halobacteria;o Halobacteriales;f Haloferacaceae;g Halorubrum;s
SRR9330146MAG019	No	98.75	1.18	5295794	200	46923	61.1	89.12	3923	d Bacteria;p Planctomycetota;c Planctomycetia;o Pirellulales;f Pirellulaceae;g UBA6163;s UBA6163 sp007127095
SRR9330146MAG020	No	88.18	1.09	3592189	539	8583	52.4	87.65	3467	d Bacteria;p Cyanobacteria;c Cyanobacteriia;o Cyanobacteriales;f Geitlerinemaceae;g Phormidium A;s Phormidium A sp007126595
SRR9330146MAG021	Yes	93.89	5.1	3953466	549	9980	58.9	90.52	3509	d Bacteria;p Verrucomicrobiota;c Kiritimatiellae;o SS1-B-03-39;f UBA6053;g PXAS01;s PXAS01 sp003565815
SRR9330146MAG023	Yes	88.14	2.03	1892929	606	3959	50	89.28	2138	d Bacteria;p T1Sed10-126;c T1Sed10-126;o T1Sed10-126;f T1Sed10-126;g ;s
SRR9330146MAG024	Yes	96.07	6.67	2866241	194	30801	67.3	87.71	2994	d Archaea;p Halobacteriota;c Halobacteria;o Halobacteriales;f Haloferacaceae;g Halorubrum;s
SRR9330146MAG025	Yes	89.19	4.4	3041048	713	5167	68.4	91.48	3111	d Bacteria;p Gemmatimonadota;c Gemmatimonadetes;o Longimicrobiales;f UBA6960;g SLBA01;s
SRR9330146MAG026	No	95.73	4.61	3754252	343	19100	71.4	90.27	3648	d Bacteria;p Actinobacteriota;c Actinomycetia;o Nitriliruptorales;f Nitriliruptoraceae;g PWLR01;s
SRR9330146MAG027	Yes	85.88	8.65	3956807	601	11501	60.6	90.81	3854	d Bacteria;p Proteobacteria;c Gammaproteobacteria;o Xanthomonadales;f S139-57;g ;s
SRR9330146MAG028	Yes	91.16	4.8	3456988	649	7089	39.3	87.04	3275	d Bacteria;p Bacteroidota;c Bacteroidia;o Chitinophagales;f Saprospiraceae;g PWJY01;s
SRR9330146MAG029	No	96.2	4.68	3515865	306	20191	54.5	89.43	3053	d Bacteria;p Bacteroidota;c Rhodothermia;o Balneolales;f Natronogracilivirgulaceae;g SKNL01;s SKNL01 sp007120555
SRR9330146MAG030	No	93.39	9.77	3077339	205	25926	65.3	92.3	2931	d Bacteria;p Proteobacteria;c Gammaproteobacteria;o Xanthomonadales;f Wenzhouxiangellaceae;g Wenzhouxiangella;s Wenzhouxiangella sp003560975
SRR9330146MAG031	No	96.25	8.5	3696685	186	42775	60	90.92	3692	d Bacteria;p Proteobacteria;c Alphaproteobacteria;o Rhodobacterales;f Rhodobacteraceae;g Roseinatronobacter;s Roseinatronobacter monicus
SRR9330146MAG032	Yes	84.82	3.73	2700732	325	13763	63.3	93.21	2705	d Bacteria;p Proteobacteria;c Gammaproteobacteria;o Xanthomonadales;f Wenzhouxiangellaceae;g Wenzhouxiangella;s
SRR9330146MAG033	No	82	0.67	1210151	432	3397	49.4	92.88	1523	d Bacteria;p Firmicutes;c Bacilli;o Izemoplasmatales;f Izemoplasmataceae;g T1SED10-81;s T1SED10-81 sp003554025
SRR9330146MAG035	Yes	94.49	4.42	4108638	230	34966	61.4	90.74	3626	d Bacteria;p Verrucomicrobiota;c Verrucomicrobiae;o Verrucomicrobiales;f Akkermansiaceae;g Luteolibacter;s
SRR9330146MAG036	No	96.93	9.37	2788244	499	10109	35.9	90.02	3110	d Bacteria;p Firmicutes F;c Halanaerobiia;o Halanaerobiales;f Halarsenatibacteraceae;g SLSL01;s SLSL01 sp007130465
SRR9330146MAG037	No	84.54	5.73	1438258	413	4115	71.7	93.08	1739	d Bacteria;p Actinobacteriota;c Actinomycetia;o Nitriliruptorales;f Nitriliruptoraceae;g CSBr16-57R1;s
SRR9330146MAG038	Yes	89.46	3.63	2391638	278	12536	61.3	89.49	2724	d Archaea;p Halobacteriota;c Halobacteria;o Halobacteriales;f Haloferacaceae;g Halohasta;s
SRR9330146MAG039	Yes	74	2.26	4790789	1158	4775	57.5	90.68	4683	d Bacteria;p Myxococcota;c Bradymonadia;o Bradymonadales;f Bradymonadaceae;g SKNI01;s
SRR9330146MAG040	No	74.28	7.41	1275289	391	3650	58.7	94.74	1609	d Bacteria;p Actinobacteriota;c Actinomycetia;o Actinomycetales;f Microbacteriaceae;g Pontimonas;s
SRR9330146MAG041	No	91.09	6.55	3125366	339	17470	64.1	87.93	3289	d Archaea;p Halobacteriota;c Halobacteria;o Halobacteriales;f Haloferacaceae;g Halorubrum;s Halorubrum sp001564205
SRR9330146MAG044	Yes	89.57	4.4	4194767	780	9388	58.7	89.79	4715	d Bacteria;p Proteobacteria;c Alphaproteobacteria;o Rhodobacterales;f Rhodobacteraceae;g Roseinatronobacter;s Roseinatronobacter sp007126715
SRR9330146MAG045	No	84	3.51	2004104	724	3353	50.8	91.75	2433	d Bacteria;p Firmicutes F;c Halanaerobiia;o Halanaerobiales;f Halarsenatibacteraceae;g Halarsenatibacter;s
SRR9330146MAG048	No	83.33	8.6	4666010	967	6027	51.5	89.03	4578	d Bacteria;p Chloroflexota;c Anaerolineae;o Promineofilales;f Promineofilaceae;g SLGK01;s
SRR9330146MAG061	Yes	95.12	4.82	2417619	362	10211	71.6	92.88	2539	d Bacteria;p Proteobacteria;c Gammaproteobacteria;o Nitrococcales;f Halorhodospiraceae;g Halorhodospira;s
SRR9330146MAG075	No	88.52	1.91	2926136	252	19128	47.5	88.5	2584	d Bacteria;p Bacteroidota;c Rhodothermia;o Balneolales;f PXAI01;g Cyclonatronum;s Cyclonatronum sp003555145
SRR9330146MAG076	No	92.62	3.83	3611387	278	24835	45.1	86.93	3274	d Bacteria;p Bacteroidota;c Rhodothermia;o Balneolales;f Balneolaceae;g QGGB01;s QGGB01 sp007118525

SRR9330147MAG001	Yes	99.45	0.55	2867967	71	87570	45.1	88.66	2477	d	Bacteria;p Bacteroidota;c Bacteroidia;o CAILMK01;f ;g ;s
SRR9330147MAG002	Yes	95.75	1.03	2587446	150	33944	65.6	89.58	2513	d	Bacteria;p Proteobacteria;c Gammaproteobacteria;o XJ16;f Halofilaceae;g SLKC01;s
SRR9330147MAG003	Yes	95.93	0.48	2490831	148	31809	38.4	88.71	2088	d	Bacteria;p Proteobacteria;c Gammaproteobacteria;o UBA12402;f UBA12402;g ;s
SRR9330147MAG004	No	98.04	2.85	3190804	157	46471	51.9	90.05	3412	d	Archaea;p Halobacteriota;c Halobacteria;o Halobacteriales;f Haloferacaceae;g Halalkalirubrum;s Halalkalirubrum sp003551725
SRR9330147MAG005	No	97.46	1.69	3735426	186	36605	62.3	87.45	3267	d	Bacteria;p Bacteroidota;c Rhodothermia;o Rhodothermales;f Salinibacteraceae;g Tc-Br11-B2g6-7;s Tc-Br11-B2g6-7 sp001564055
SRR9330147MAG007	Yes	96.45	2.67	3460860	236	26838	60.5	89.55	3685	d	Archaea;p Halobacteriota;c Halobacteria;o Halobacteriales;f Haloarculaceae;g Halovenus;s
SRR9330147MAG008	Yes	76.64	0	806959	11	103030	39.7	92.93	868	d	Archaea;p Nanoarchaeota;c Nanoarchaeia;o Woesearchaeales;f 21-14-0-10-32-9;g ;s
SRR9330147MAG010	No	82.24	5.28	2519006	571	5373	43.8	89.16	2660	d	Bacteria;p Bacteroidota;c Rhodothermia;o Balneolales;f Balneolaceae;g SW132;s
SRR9330147MAG011	No	86.25	2.61	2488254	95	46311	67.9	87.87	2539	d	Archaea;p Halobacteriota;c Halobacteria;o Halobacteriales;f Haloferacaceae;g Halorubrum;s
SRR9330147MAG012	Yes	84.12	3.26	2439176	189	25913	61.8	88.54	2680	d	Archaea;p Halobacteriota;c Halobacteria;o Halobacteriales;f Haloferacaceae;g Halorubrum;s
SRR9330147MAG013	No	82.45	3.35	5178074	535	14357	60.7	86.87	4277	d	Bacteria;p Myxococcota;c Bradymonadia;o Bradymonadales;f Bradymonadaceae;g SLJM01;s
SRR9330147MAG014	No	70.17	1.74	2298701	338	8153	57.2	85.16	2218	d	Bacteria;p Bacteroidota;c Rhodothermia;o Rhodothermales;f Salinibacteraceae;g Longimonas;s
SRR9330147MAG015	No	87.94	5.78	2651136	449	13664	59.6	87.52	3250	d	Archaea;p Halobacteriota;c Halobacteria;o Halobacteriales;f Haloferacaceae;g PL-Br10-E2g29;s
SRR9330147MAG016	No	82.5	3.33	2587413	228	20343	61.1	88.22	2830	d	Archaea;p Halobacteriota;c Halobacteria;o Halobacteriales;f Haloarculaceae;g Natronomonas;s
SRR9330147MAG017	No	83.2	4.2	2276227	63	92641	58.3	89.3	2437	d	Archaea;p Halobacteriota;c Halobacteria;o Halobacteriales;f Haloferacaceae;g PL-Br10-E2g29;s
SRR9330147MAG018	Yes	84.96	2.38	3281908	645	6805	59.4	85.85	3144	d	Bacteria;p Bacteroidota;c Rhodothermia;o Rhodothermales;f Salinibacteraceae;g Longimonas;s
SRR9330147MAG020	No	75.97	4.13	2194680	107	32377	66.9	85.52	2195	d	Archaea;p Halobacteriota;c Halobacteria;o Halobacteriales;f Haloferacaceae;g Halorubrum;s
SRR9330147MAG021	No	90.96	6.91	2539173	157	25593	64.3	87.6	2734	d	Archaea;p Halobacteriota;c Halobacteria;o Halobacteriales;f Haloarculaceae;g Natronomonas;s
SRR9330147MAG022	Yes	79.28	4.41	2102543	323	9099	68	88.9	2424	d	Archaea;p Halobacteriota;c Halobacteria;o Halobacteriales;f Haloferacaceae;g Halorubrum;s
SRR9330147MAG026	No	80.66	8.28	2456302	330	10520	60.8	89.51	2782	d	Archaea;p Halobacteriota;c Halobacteria;o Halobacteriales;f Haloferacaceae;g Halohasta;s
SRR9330147MAG028	Yes	78.48	5.14	1211450	231	6543	38.2	90.09	1627	d	Archaea;p Nanohaloarchaeota;c Nanosalinia;o Nanosalinales;f Nanosalinaceae;g ;s
SRR9330147MAG030	Yes	84.85	1.66	2105717	236	12075	66.2	87.9	2239	d	Archaea;p Halobacteriota;c Halobacteria;o Halobacteriales;f Haloferacaceae;g Halorubrum;s
SRR9330147MAG035	Yes	78.58	3.44	2281591	255	30299	65.3	88.33	2539	d	Archaea;p Halobacteriota;c Halobacteria;o Halobacteriales;f Haloferacaceae;g Halorubrum;s
SRR9330148MAG001	No	96.9	0.76	2729927	103	43289	51.9	90.37	2900	d	Archaea;p Halobacteriota;c Halobacteria;o Halobacteriales;f Haloferacaceae;g Halalkalirubrum;s Halalkalirubrum sp003551725
SRR9330148MAG002	No	94.59	1.97	2607569	138	39603	65.5	89.71	2512	d	Bacteria;p Proteobacteria;c Gammaproteobacteria;o XJ16;f Halofilaceae;g SLKC01;s
SRR9330148MAG003	No	88.02	4.28	2393054	61	141982	58.2	89.23	2525	d	Archaea;p Halobacteriota;c Halobacteria;o Halobacteriales;f Haloferacaceae;g PL-Br10-E2g29;s
SRR9330148MAG006	No	82.63	0.93	787696	56	34817	39.9	92.65	1016	d	Archaea;p Nanohaloarchaeota;c Nanosalinia;o Nanosalinales;f Nanosalinaceae;g B1-Br10-U2g21;s B1-Br10-U2g21 sp001564145
SRR9330148MAG007	No	85.3	0.93	901753	37	47815	40.7	91.74	1078	d	Archaea;p Nanohaloarchaeota;c Nanosalinia;o Nanosalinales;f Nanosalinaceae;g B1-Br10-U2g19;s B1-Br10-U2g19 sp001563905
SRR9330148MAG011	No	84.73	4.1	3862469	1469	3033	54.6	91.06	5638	d	Bacteria;p Proteobacteria;c Gammaproteobacteria;o Nitrococcales;f Nitrococcaceae;g Spiribacter;s
SRR9330148MAG012	No	89.34	2.66	1850875	95	27088	62.2	89.14	1992	d	Archaea;p Halobacteriota;c Halobacteria;o Halobacteriales;f Haloferacaceae;g PL-Br10-E2g29;s PL-Br10-E2g29 sp001563965
SRR9330148MAG013	No	83.13	6.77	2301139	195	19717	59.4	89.51	2544	d	Archaea;p Halobacteriota;c Halobacteria;o Halobacteriales;f Haloferacaceae;g Halohasta;s Halohasta sp003554115
SRR9330148MAG014	No	92.93	7.15	2804618	202	24072	64.7	88.59	2942	d	Archaea;p Halobacteriota;c Halobacteria;o Halobacteriales;f Haloferacaceae;g Halorubrum;s Halorubrum sp001564205
SRR9330148MAG016	No	88.94	1.74	3012219	761	4633	57.4	84.86	3270	d	Bacteria;p Bacteroidota;c Rhodothermia;o Rhodothermales;f Salinibacteraceae;g Longimonas;s
SRR9330148MAG018	No	90.4	9.03	2768131	351	19081	64.8	89.06	3074	d	Archaea;p Halobacteriota;c Halobacteria;o Halobacteriales;f Haloferacaceae;g Halorubrum;s Halorubrum sp003554605
SRR9330148MAG019	No	73.02	2.55	2213508	988	2398	55	89.65	3007	d	Archaea;p Halobacteriota;c Halobacteria;o Halobacteriales;f Haloarculaceae;g Halovenus;s Halovenus sp001564135
SRR9330148MAG020	No	83.91	4.4	2405139	111	35500	64.8	87.92	2586	d	Archaea;p Halobacteriota;c Halobacteria;o Halobacteriales;f Haloarculaceae;g Natronomonas;s
SRR9330149MAG001	Yes	99.6	2	3365480	112	93929	67	91.63	3296	d	Bacteria;p Proteobacteria;c Alphaproteobacteria;o Rhodobacterales;f Rhodobacteraceae;g Rhodobaculum;s
SRR9330149MAG002	No	95.64	1.84	2325544	300	25708	55.6	94.85	2657	d	Bacteria;p Proteobacteria;c Gammaproteobacteria;o Nitrococcales;f Nitrococcaceae;g Spiribacter;s
SRR9330149MAG003	Yes	96.45	0.86	4017826	235	25441	65.3	88.58	3734	d	Bacteria;p Proteobacteria;c Alphaproteobacteria;o Rhizobiales;f Beijerinckiaceae;g Salinarimonas;s Salinarimonas sp003567055
SRR9330149MAG004	No	97.46	2.54	3569075	289	21394	62.6	88.13	3271	d	Bacteria;p Bacteroidota;c Rhodothermia;o Rhodothermales;f Salinibacteraceae;g Tc-Br11-B2g6-7;s Tc-Br11-B2g6-7 sp001564055
SRR9330149MAG005	Yes	98.23	4.49	2951794	216	168830	58.1	89.09	3279	d	Archaea;p Halobacteriota;c Halobacteria;o Halobacteriales;f Haloferacaceae;g PL-Br10-E2g29;s
SRR9330149MAG006	No	97.54	6.19	3893854	238	55654	41.8	88.03	3623	d	Bacteria;p Bacteroidota;c Rhodothermia;o Balneolales;f Balneolaceae;g ;s
SRR9330149MAG007	No	93.69	5.22	2748435	169	38062	65.4	89.76	2725	d	Bacteria;p Proteobacteria;c Gammaproteobacteria;o XJ16;f Halofilaceae;g SLKC01;s
SRR9330149MAG008	No	96.58	3.18	3317233	132	49001	71.3	90.3	3134	d	Bacteria;p Actinobacteriota;c Actinomycetia;o Nitriliruptorales;f Nitriliruptoraceae;g PWLR01;s
SRR9330149MAG010	No	94.24	5.11	1991922	130	28418	62	88.89	2190	d	Archaea;p Halobacteriota;c Halobacteria;o Halobacteriales;f Haloferacaceae;g PL-Br10-E2g29;s PL-Br10-E2g29 sp001563965
SRR9330149MAG011	No	71.71	4.67	4273792	1279	3878	51.9	86.62	5360	d	Archaea;p Halobacteriota;c Halobacteria;o Halobacteriales;f Haloferacaceae;g Halalkalirubrum;s Halalkalirubrum sp003551725
SRR9330149MAG013	No	86.48	2.37	2297312	235	17392	61.6	89.82	2629	d	Archaea;p Halobacteriota;c Halobacteria;o Halobacteriales;f Haloferacaceae;g Halohasta;s
SRR9330149MAG014	Yes	89.25	1.05	1369843	55	92808	61.6	93.08	1391	d	Bacteria;p Actinobacteriota;c Actinomycetia;o Actinomycetales;f Microbacteriaceae;g Pontimonas;s
SRR9330149MAG015	No	91.51	0.38	2385657	148	25047	66.4	89.5	2484	d	Archaea;p Halobacteriota;c Halobacteria;o Halobacteriales;f Haloferacaceae;g Halorubrum;s Halorubrum sp003554605
SRR9330149MAG016	No	94.92	1.93	2605791	226	22001	63	89.39	2610	d	Bacteria;p Proteobacteria;c Gammaproteobacteria;o Nitrococcales;f Aquisalimonadaceae;g SKGQ01;s SKGQ01 sp007117515
SRR9330149MAG017	Yes	92.31	5.49	1658858	295	8269	72.7	93.77	1865	d	Bacteria;p Actinobacteriota;c Actinomycetia;o Nitriliruptorales;f Nitriliruptoraceae;g CSBr16-57R1;s
SRR9330149MAG018	No	93.19	6.99	3620136	622	9021	65.7	90.73	3832	d	Bacteria;p Proteobacteria;c Gammaproteobacteria;o Nitrococcales;f Aquisalimonadaceae;g Aquisalimonas;s Aquisalimonas sp012044895
SRR9330149MAG019	Yes	91.92	7.97	2608409	222	28009	59.6	89.66	2856	d	Archaea;p Halobacteriota;c Halobacteria;o Halobacteriales;f Haloferacaceae;g Halohasta;s Halohasta sp003554115
SRR9330149MAG021	Yes	90.38	6.56	3407239	614	7516	41.7	89.47	3420	d	Bacteria;p Bacteroidota;c Rhodothermia;o Balneolales;f Balneolaceae;g ;s
SRR9330150MAG001	Yes	100	0	2481893	51	78193	40.4	92.65	2061	d	Bacteria;p Bacteroidota;c Bacteroidia;o NS11-12g;f UBA955;g UBA6161;s
SRR9330150MAG002	Yes	98.39	0.73	3768863	234	31463	53.8	91.69	3580	d	Bacteria;p Proteobacteria;c Gammaproteobacteria;o Pseudomonadales;f Nitrincolaceae;g Nitrincola;s Nitrincola alkalilacustris
SRR9330150MAG003	Yes	95.71	4.61	3851521	244	27655	62.7	91.36	3455	d	Bacteria;p Proteobacteria;c Gammaproteobacteria;o Xanthomonadales;f Wenzhouxiangellaceae;g Wenzhouxiangella;s
SRR9330150MAG004	No	98.35	1.57	2905895	210	22809	35.1	89.32	2686	d	Bacteria;p Bacteroidota;c Bacteroidia;o Flavobacteriales;f Flavobacteriaceae;g Psychroflexus;s Psychroflexus sp007116535
SRR9330150MAG005	Yes	98.55	0	1015445	56	38748	40.7	94.33	1042	d	Bacteria;p Firmicutes;c Bacilli;o Izemoplasmatales;f Izemoplasmataceae;g CSBR16-87;s
SRR9330150MAG006	Yes	96.24	2.34	2781886	259	16993	66.1	91.34	2841	d	Bacteria;p Proteobacteria;c Alphaproteobacteria;o Caulobacterales;f Maricaulaceae;g Oceanicaulis;s
SRR9330150MAG007	No	89.56	2.13	1944962	300	8947	59	95.02	2210	d	Bacteria;p Actinobacteriota;c Actinomycetia;o Nanopelagicales;f S36-B12;g S36-B12;s
SRR9330150MAG008	Yes	97.3	2.03	2795123	80	57500	56.1	92.46	2370	d	Bacteria;p Verrucomicrobiota;c Verrucomicrobiae;o Opitutales;f Verruco-01;g ;s
SRR9330150MAG009	Yes	99.32	1.24	3367931	121	51128	56.1	90.59	3009	d	Bacteria;p Verrucomicrobiota;c Verrucomicrobiae;o Opitutales;f JABDGR01;g ;s
SRR9330150MAG010	Yes	99.32	3.04	4190535	219	33383	56.2	90.33	3767	d	Bacteria;p Verrucomicrobiota;c Verrucomicrobiae;o Opitutales;f DSM-45221;g BACL24;s
SRR9330150MAG011	Yes	89.7	2.06	3635180	305	22857	59.1	90.94	3214	d	Bacteria;p Verrucomicrobiota;c Kiritimatiellae;o SS1-B-03-39;f UBA6053;g PXAS01;s PXAS01 sp003565035
SRR9330150MAG012	No	97.46	2.13	1989206	107	30597	58.8	94.32	2013	d	Bacteria;p Proteobacteria;c Gammaproteobacteria;o Burkholderiales;f Burkholderiaceae;g ;s
SRR9330150MAG013	Yes	98.38	1.08	4309023	488	17760	34.4	88.35	4368	d	Bacteria;p Bacteroidota;c Bacteroidia;o Flavobacteriales;f Crocinitomicaceae;g ;s
SRR9330150MAG014	Yes	86.73	2.19	1192918	87	24311	56.7	94.63	1266	d	Bacteria;p Actinobacteriota;c Actinomycetia;o Actinomycetales;f Microbacteriaceae;g Pontimonas;s
SRR9330150MAG015	Yes	98.75	1.18	5612205	161	61018	65.4	88.88	4151	d	Bacteria;p Planctomycetota;c Planctomycetia;o Pirellulales;f Pirellulaceae;g ;s
SRR9330150MAG016	Yes	98.73	1.31	5289971	374	22785	44.5	84.72	4812	d	Bacteria;p Cyanobacteria;c Cyanobacteriia;o Cyanobacteriales;f Microcoleaceae;g Limnospira;s Limnospira fusiformis
SRR9330150MAG017	Yes	93.2	2.64	6118182	118	99782	55	89.47	4704	d	Bacteria;p Myxococcota;c Bradymonadia;o Bradymonadales;f Bradymonadaceae;g SKON01;s
SRR9330150MAG018	Yes	97.6	2.4	2679802	142	29453	55.1	95.03	2550	d	Bacteria;p Spirochaetota;c Spirochaetia;o DSM-27196;f Alkalispirochaetaceae;g Alkalispirochaeta;s
SRR9330150MAG019	Yes	98.31	0.36	5091420	416	18670	40.9	80.81	4662	d	Bacteria;p Cyanobacteria;c Cyanobacteriia;o Cyanobacteriales;f Nostocaceae;g Nodularia;s
SRR9330150MAG020	Yes	92.84	6.6	3971152	78	129091	66.4	92.51	3742	d	Bacteria;p Proteobacteria;c Gammaproteobacteria;o PWYM01;f PWYM01;g PWYM01;s PWYM01 sp007127715
SRR9330150MAG021	Yes	95.3	1.75	3186384	220	23557	60.2	91.22	3206	d	Bacteria;p Proteobacteria;c Alphaproteobacteria;o Rhodobacterales;f Rhodobacteraceae;g Roseinatronobacter;s
SRR9330150MAG022	Yes	87.61	0.71	2367873	103	35629	58.8	92.44	2162	d	Bacteria;p Verrucomicrobiota;c Kiritimatiellae;o SS1-B-03-39;f UBA6053;g PXAS01;s
SRR9330150MAG023	No	90.98	0.54	2196381	393	6875	45	91.5	2209	d	Bacteria;p Bacteroidota;c Bacteroidia;o Flavobacteriales;f Schleiferiaceae;g CSBr16-58;s CSBr16-58 sp003566715
SRR9330150MAG024	Yes	97.22	5.23	4066306	337	30533	60.2	90.78	3765	d	Bacteria;p Proteobacteria;c Gammaproteobacteria;o Xanthomonadales;f ;g ;s
SRR9330150MAG025	No	94.35	4.84	2321946	306	11383	47.4	92.17	2296	d	Bacteria;p Proteobacteria;c Gammaproteobacteria;o Enterobacterales;f Alteromonadaceae;g Aliidiomarina;s
SRR9330150MAG026	Yes	96.59	1.01	5006423	222	53356	70.3	92.37	4172	d	Bacteria;p Myxococcota A;c UBA796;o UBA796;f GCA-2862545;g ;s
SRR9330150MAG027	Yes	99.12	3.73	5651020	364	82471	54.6	89.57	5677	d	Bacteria;p Proteobacteria;c Gammaproteobacteria;o Pseudomonadales;f Natronospirillaceae;g ;s
SRR9330150MAG029	Yes	81.33	6	922093	266	5131	43.6	94.17	1196	d	Bacteria;p Firmicutes;c Bacilli;o MAG-NZ;f MAG-NZ;g M30B81;s M30B81 sp018401215
SRR9330150MAG030	Yes	85.76	3.74	3038401	598	6191	62.6	90.63	3069	d	Bacteria;p Verrucomicrobiota;c Verrucomicrobiae;o Verrucomicrobiales;f Akkermansiaceae;g Luteolibacter;s
SRR9330150MAG031	No	77.42	1.57	1371057	286	6814	72.6	93.89	1551	d	Bacteria;p Actinobacteriota;c Actinomycetia;o Nitriliruptorales;f Nitriliruptoraceae;g CSBr16-57R1;s
SRR9330150MAG033	Yes	74.06	2.65	1070235	136	14889	57.9	94.35	1202	d	Bacteria;p Actinobacteriota;c Actinomycetia;o Actinomycetales;f Microbacteriaceae;g Pontimonas;s
SRR9330150MAG034	Yes	85.22	5.06	4084520	1007	4910	30.9	89.63	4392	d	Bacteria;p Bacteroidota;c Bacteroidia;o AKYH767;f JACCXN01;g ;s
SRR9330150MAG035	Yes	78.49	9.39	1422796	215	8654	58.4	94.66	1571	d	Bacteria;p Actinobacteriota;c Actinomycetia;o Actinomycetales;f Microbacteriaceae;g Pontimonas;s
SRR9330150MAG036	No	81.99	7.86	2159924	272	19690	65.7	93.55	2254	d	Bacteria;p Actinobacteriota;c Acidimicrobiia;o Acidimicrobiales;f Ilumatobacteraceae;g CSBr16-110;s CSBr16-110 sp007117775
SRR9330150MAG037	No	84.48	8.83	3910657	742	7070	65.7	89.92	4115	d	Bacteria;p Proteobacteria;c Alphaproteobacteria;o Rhizobiales;f Beijerinckiaceae;g Salinarimonas;s Salinarimonas sp003567055
SRR9330150MAG039	No	92.11	3.58	3280679	467	10073	43	91.02	3134	d	Bacteria;p Bacteroidota;c Rhodothermia;o Balneolales;f Balneolaceae;g UBA2664;s UBA2664 sp007123985

SRR9330150MAG040	Yes	87.95	4.47	3497167	782	5641	61.5	93.84	3836	d	Bacteria;p	Spirochaetota;c Spirochaetia;o DSM-27196;f PWMO01;g PWMO01;s
SRR9330150MAG041	No	82.54	3.51	1917301	488	4428	55.2	92.87	2193	d	Bacteria;p	Proteobacteria;c Gammaproteobacteria;o Burkholderiales;f Burkholderiaceae;g Algicoccus;s
SRR9330150MAG042	Yes	95.39	1.92	2570890	277	13090	59.9	92.23	2639	d	Bacteria;p	Proteobacteria;c Alphaproteobacteria;o Sphingomonadales;f Sphingomonadaceae;g Erythrobacter;s
SRR9330150MAG043	Yes	85.02	2.8	3506441	991	4375	43.9	91.04	3718	d	Bacteria;p	Spirochaetota;c Spirochaetia;o DSM-27196;f Salinispiraceae;g SKVS01;s
SRR9330150MAG044	Yes	80.48	9.54	2313300	799	3389	41	88.76	2732	d	Bacteria;p	Firmicutes A;c Clostridia;o Peptostreptococcales;f T1SED10-28;g Isachenkonia;s
SRR9330150MAG047	No	70.52	0.43	1193569	134	19417	65.8	93.81	1232	d	Bacteria;p	Actinobacteriota;c Acidimicrobiia;o Acidimicrobiales;f Ilumatobacteraceae;g CSBr16-110;s
SRR9330150MAG049	No	76.77	5.4	3107380	1178	2957	41.5	85.91	3179	d	Bacteria;p	Bacteroidota;c Bacteroidia;o Chitinophagales;f Saprospiraceae;g SLDF01;s SLDF01 sp007125435
SRR9330150MAG050	Yes	94.51	3.33	3126477	289	19421	49.6	91.93	3079	d	Bacteria;p	Proteobacteria;c Gammaproteobacteria;o Enterobacterales;f Alteromonadaceae;g Alkalimonas;s
SRR9330150MAG051	Yes	92.79	3.91	1888766	220	15696	52.4	93.69	1966	d	Bacteria;p	Proteobacteria;c Gammaproteobacteria;o Pseudomonadales;f Natronospirillaceae;g Natronospirillum;s
SRR9330150MAG052	Yes	85.89	3.64	3337583	864	4591	63.1	91.48	3801	d	Bacteria;p	Proteobacteria;c Gammaproteobacteria;o Burkholderiales;f Rhodocyclaceae;g Azoarcus F;s
SRR9330150MAG065	No	73.96	6.4	2094292	592	3801	56.2	91.59	2397	d	Bacteria;p	Proteobacteria;c Gammaproteobacteria;o Pseudomonadales;f Alcanivoracaceae;g Alcanivorax;s
SRR9330151MAG002	No	92.77	2.4	2476569	165	21262	55.4	90.13	2654	d	Archaea;p	Halobacteriota;c Halobacteria;o Halobacteriales;f Haloarculaceae;g Halovenus;s Halovenus sp001564135
SRR9330151MAG003	Yes	84.27	2.34	921650	35	37444	44.8	91.36	1045	d	Archaea;p	Nanohaloarchaeota;c Nanosalinia;o Nanosalinales;f Nanosalinaceae;g Nanosalina;s
SRR9330151MAG004	Yes	93.31	1.45	3378336	252	19828	57.4	85.13	3016	d	Bacteria;p	Bacteroidota;c Rhodothermia;o Rhodothermales;f Salinibacteraceae;g Longimonas;s
SRR9330151MAG005	No	79.71	4.47	2565983	692	4142	60.8	92.85	2920	d	Bacteria;p	Proteobacteria;c Gammaproteobacteria;o Pseudomonadales;f Oleiphilaceae;g Halospina;s
SRR9330151MAG006	No	83.75	0.93	738125	32	32032	41	91.46	896	d	Archaea;p	Nanohaloarchaeota;c Nanosalinia;o Nanosalinales;f Nanosalinaceae;g B1-Br10-U2g19;s B1-Br10-U2g19 sp001563905
SRR9330151MAG008	Yes	86.6	2.9	2460428	189	19251	63.6	88.91	2573	d	Archaea;p	Halobacteriota;c Halobacteria;o Halobacteriales;f Haloferacaceae;g Halorubrum;s
SRR9330151MAG009	No	79.44	8.84	2502086	88	47156	64.8	88.77	2620	d	Archaea;p	Halobacteriota;c Halobacteria;o Halobacteriales;f Haloarculaceae;g Natronomonas;s
SRR9330151MAG010	No	89.96	3.91	3016187	445	9854	64	88.56	3390	d	Archaea;p	Halobacteriota;c Halobacteria;o Halobacteriales;f Haloarculaceae;g Natronomonas;s
SRR9330151MAG011	No	79.04	2.28	1849640	99	30612	61.9	88.63	1971	d	Archaea;p	Halobacteriota;c Halobacteria;o Halobacteriales;f Haloferacaceae;g PL-Br10-E2g29;s PL-Br10-E2g29 sp001563965
SRR9330151MAG012	No	86.9	3.2	2782952	289	12909	64.2	84.94	2884	d	Archaea;p	Halobacteriota;c Halobacteria;o Halobacteriales;f Natrialbaceae;g Tc-Br11;s Tc-Br11 sp001564275
SRR9330151MAG016	No	75.86	6.58	2316766	220	15337	66	88.14	2413	d	Archaea;p	Halobacteriota;c Halobacteria;o Halobacteriales;f Haloferacaceae;g Halorubrum;s
SRR9330151MAG017	No	76.39	1.42	2157501	146	23815	66.2	89.4	2222	d	Archaea;p	Halobacteriota;c Halobacteria;o Halobacteriales;f Haloferacaceae;g Halorubrum;s Halorubrum sp003554605
SRR9330151MAG018	No	87.55	9.23	2481842	313	12809	58.4	89.15	2829	d	Archaea;p	Halobacteriota;c Halobacteria;o Halobacteriales;f Haloferacaceae;g PL-Br10-E2g29;s
SRR9330151MAG019	No	77.38	4.94	2357849	137	31412	67	85.54	2406	d	Archaea;p	Halobacteriota;c Halobacteria;o Halobacteriales;f Haloferacaceae;g Halorubrum;s
SRR9330151MAG024	No	85.91	9.69	3014336	881	4007	67.9	90.73	3430	d	Bacteria;p	Proteobacteria;c Gammaproteobacteria;o Pseudomonadales;f Halomonadaceae;g Halomonas;s Halomonas sp003552795
SRR9330151MAG026	No	88.03	7.02	2527420	128	29898	64.2	87.71	2668	d	Archaea;p	Halobacteriota;c Halobacteria;o Halobacteriales;f Haloarculaceae;g Natronomonas;s
SRR9330151MAG027	No	70.39	3.15	2133568	177	16963	64.9	88.57	2234	d	Archaea;p	Halobacteriota;c Halobacteria;o Halobacteriales;f Haloferacaceae;g Halorubrum;s Halorubrum sp001564205
SRR9330151MAG037	No	79.28	9.36	2375551	376	9413	67.8	88.43	2601	d	Archaea;p	Halobacteriota;c Halobacteria;o Halobacteriales;f Haloferacaceae;g Halorubrum;s
SRR9330152MAG001	Yes	96.16	0.52	2025399	74	103924	55.1	96.54	2005	d	Bacteria;p	Proteobacteria;c Gammaproteobacteria;o Nitrococcales;f Nitrococcaceae;g Spiribacter;s
SRR9330152MAG002	Yes	98.04	1.14	2832731	80	57972	51.7	90.12	2994	d	Archaea;p	Halobacteriota;c Halobacteria;o Halobacteriales;f Haloferacaceae;g Halalkalirubrum;s Halalkalirubrum sp003551725
SRR9330152MAG003	Yes	98.29	2.56	3515143	114	79963	71.4	90.32	3269	d	Bacteria;p	Actinobacteriota;c Actinomycetia;o Nitriliruptorales;f Nitriliruptoraceae;g PWLR01;s
SRR9330152MAG004	Yes	99.38	2.47	3565857	216	31956	64.4	90.71	3577	d	Bacteria;p	Proteobacteria;c Alphaproteobacteria;o Rhodobacterales;f Rhodobacteraceae;g Roseinatronobacter;s Roseinatronobacter sp003561595
SRR9330152MAG005	No	95.2	3.67	3758821	470	14291	62.7	88.14	3533	d	Bacteria;p	Bacteroidota;c Rhodothermia;o Rhodothermales;f Salinibacteraceae;g Tc-Br11-B2g6-7;s Tc-Br11-B2g6-7 sp001564055
SRR9330152MAG006	No	94.81	4.1	3741525	177	75014	41.7	87.8	3325	d	Bacteria;p	Bacteroidota;c Rhodothermia;o Balneolales;f Balneolaceae;g ;s
SRR9330152MAG007	No	96.01	2.29	2937827	240	19920	62.8	89.48	2861	d	Bacteria;p	Proteobacteria;c Gammaproteobacteria;o Nitrococcales;f Aquisalimonadaceae;g SKGQ01;s SKGQ01 sp007117515
SRR9330152MAG008	No	80.76	2.93	1674215	279	10226	63.8	94.04	1839	d	Bacteria;p	Proteobacteria;c Gammaproteobacteria;o Nitrococcales;f Nitrococcaceae;g Spiribacter;s Spiribacter sp009676705
SRR9330152MAG009	No	86.89	5.48	3963650	484	10664	46.1	86.24	3588	d	Bacteria;p	Bacteroidota;c Rhodothermia;o Balneolales;f Balneolaceae;g SW132;s
SRR9330152MAG010	No	93.95	2.57	2493684	82	47894	64.9	89.03	2630	d	Archaea;p	Halobacteriota;c Halobacteria;o Halobacteriales;f Haloarculaceae;g Natronomonas;s
SRR9330152MAG012	No	98.29	1.32	3610346	144	57520	71	88.12	3347	d	Bacteria;p	Actinobacteriota;c Actinomycetia;o Nitriliruptorales;f Nitriliruptoraceae;g T1Sed10-7;s
SRR9330152MAG013	No	81.47	7.87	2026796	195	20213	60.9	87.37	2236	d	Archaea;p	Halobacteriota;c Halobacteria;o Halobacteriales;f Haloferacaceae;g PL-Br10-E2g29;s
SRR9330152MAG015	No	89.15	6.9	2430583	156	25841	66.3	89.37	2531	d	Archaea;p	Halobacteriota;c Halobacteria;o Halobacteriales;f Haloferacaceae;g Halorubrum;s Halorubrum sp003554605
SRR9330152MAG017	No	81.11	5.99	2182435	240	15633	61.5	90.02	2461	d	Archaea;p	Halobacteriota;c Halobacteria;o Halobacteriales;f Haloferacaceae;g Halohasta;s
SRR9330152MAG018	No	79.47	0.93	915009	279	4276	40.6	91.17	1305	d	Archaea;p	Nanohaloarchaeota;c Nanosalinia;o Nanosalinales;f Nanosalinaceae;g B1-Br10-U2g19;s B1-Br10-U2g19 sp001563905
SRR9330152MAG020	No	73.19	1.4	728414	116	7656	39.7	92.26	1004	d	Archaea;p	Nanohaloarchaeota;c Nanosalinia;o Nanosalinales;f Nanosalinaceae;g B1-Br10-U2g21;s B1-Br10-U2g21 sp001564145
SRR9330152MAG022	No	71.29	2.85	2049847	66	54721	67.8	88.24	2074	d	Archaea;p	Halobacteriota;c Halobacteria;o Halobacteriales;f Haloferacaceae;g Halorubrum;s
SRR9330152MAG023	Yes	82.65	2.66	5542446	1290	5309	70.8	88.15	6420	d	Bacteria;p	Actinobacteriota;c Actinomycetia;o Nitriliruptorales;f Nitriliruptoraceae;g T1Sed10-7;s
SRR9330152MAG027	Yes	86	6.42	2599125	202	30843	60.3	88.85	2877	d	Archaea;p	Halobacteriota;c Halobacteria;o Halobacteriales;f Haloferacaceae;g Halohasta;s
SRR9330153MAG001	Yes	97.31	1.08	2942849	105	59766	48	90.24	2353	d	Bacteria;p	Bacteroidota;c Bacteroidia;o Bacteroidales;f UBA7960;g PUPG01;s PUPG01 sp007124575
SRR9330153MAG002	No	95.75	1.29	2534642	301	50227	55.2	94.07	2881	d	Bacteria;p	Proteobacteria;c Gammaproteobacteria;o Nitrococcales;f Nitrococcaceae;g Spiribacter;s
SRR9330153MAG003	Yes	95.7	0	1424106	63	45785	41.3	86.85	1385	d	Bacteria;p	Proteobacteria;c Alphaproteobacteria;o UBA7879;f VGCX01;g ;s
SRR9330153MAG004	Yes	84.27	0	820901	5	207672	38.8	88.69	773	d	Bacteria;p	Dependentiae;c Babeliae;o Babeliales;f Babeliaceae;g ;s
SRR9330153MAG005	No	95.75	5.11	3230195	188	36201	62.7	92.7	3053	d	Bacteria;p	Proteobacteria;c Gammaproteobacteria;o Nitrococcales;f Aquisalimonadaceae;g ;s
SRR9330153MAG006	Yes	99.48	1.27	3202970	189	35274	66.1	87.28	3143	d	Bacteria;p	Proteobacteria;c Gammaproteobacteria;o Ectothiorhodospirales;f Thioalkalivibrionaceae;g Thioalkalivibrio B;s Thioalkalivibrio B sp003563455
SRR9330153MAG007	No	96.81	0.2	3853386	178	76862	59.8	90.3	4011	d	Bacteria;p	Proteobacteria;c Alphaproteobacteria;o Rhodobacterales;f Rhodobacteraceae;g Roseinatronobacter;s Roseinatronobacter monicus
SRR9330153MAG008	Yes	98.78	0.85	3477716	205	33675	71.1	88.24	3246	d	Bacteria;p	Actinobacteriota;c Actinomycetia;o Nitriliruptorales;f Nitriliruptoraceae;g T1Sed10-7;s
SRR9330153MAG009	Yes	92.75	2.33	1959731	169	23906	58.9	95.09	1919	d	Bacteria;p	Proteobacteria;c Gammaproteobacteria;o Xanthomonadales;f Wenzhouxiangellaceae;g Wenzhouxiangella;s
SRR9330153MAG010	Yes	99.46	0.54	3348488	259	20346	40.1	90.63	2975	d	Bacteria;p	Bacteroidota;c Bacteroidia;o Flavobacteriales;f GCA-002722245;g ;s
SRR9330153MAG011	Yes	86.43	3.87	5765967	153	81607	58.8	90.21	4447	d	Bacteria;p	Myxococcota;c Bradymonadia;o Bradymonadales;f Bradymonadaceae;g SLJM01;s
SRR9330153MAG012	Yes	78.5	0	970063	48	29921	40.3	93.78	1159	d	Archaea;p	Nanoarchaeota;c Nanoarchaeia;o Woesearchaeales;f SKIA01;g SKIA01;s
SRR9330153MAG013	No	93.79	1.75	3278231	246	25693	62.6	88.35	2985	d	Bacteria;p	Bacteroidota;c Rhodothermia;o Rhodothermales;f Salinibacteraceae;g Tc-Br11-B2g6-7;s Tc-Br11-B2g6-7 sp001564055
SRR9330153MAG014	No	94.41	2.33	2674955	355	16420	33.3	88.02	2649	d	Bacteria;p	Firmicutes A;c Clostridia;o Acetivibrionales;f ;g ;s
SRR9330153MAG015	Yes	86.59	1.62	2490576	124	40177	35.7	91.01	2261	d	Bacteria;p	Bacteroidota;c Bacteroidia;o Flavobacteriales;f Crocinitomicaceae;g ;s
SRR9330153MAG016	Yes	88.89	1.03	1553088	131	19249	71.6	93.33	1593	d	Bacteria;p	Actinobacteriota;c Actinomycetia;o Nitriliruptorales;f Nitriliruptoraceae;g CSBr16-57R1;s
SRR9330153MAG018	Yes	94.52	3.27	3460465	509	9446	59.5	88.31	3267	d	Bacteria;p	Verrucomicrobiota;c Verrucomicrobiae;o Opitutales;f T3Sed10-336;g SLOW01;s
SRR9330153MAG019	Yes	79.55	6.05	3847324	728	9763	34.4	88.89	4291	d	Bacteria;p	Bacteroidota;c Bacteroidia;o Flavobacteriales;f Crocinitomicaceae;g ;s
SRR9330153MAG021	No	98.17	3.93	4128738	521	15384	64.2	90.7	4530	d	Bacteria;p	Proteobacteria;c Alphaproteobacteria;o Rhodobacterales;f Rhodobacteraceae;g Roseinatronobacter;s Roseinatronobacter sp003561595
SRR9330153MAG022	Yes	75.93	0.93	1198043	148	15239	47.6	93.31	1373	d	Archaea;p	Nanoarchaeota;c Nanoarchaeia;o Woesearchaeales;f SKIA01;g SKIA01;s
SRR9330153MAG023	Yes	76.4	2.8	919308	76	20776	48.2	93.53	1129	d	Archaea;p	Nanoarchaeota;c Nanoarchaeia;o Woesearchaeales;f SKIA01;g SKIA01;s
SRR9330153MAG024	No	71.86	1.51	1957755	820	2819	36.6	90.61	2496	d	Bacteria;p	Firmicutes F;c Halanaerobiia;o Halanaerobiales;f Halarsenatibacteraceae;g SLSL01;s SLSL01 sp007130465
SRR9330153MAG028	Yes	87.18	3.17	2381118	688	4182	34.7	90.11	2628	d	Bacteria;p	Bacteroidota;c Bacteroidia;o Flavobacteriales;f Flavobacteriaceae;g Psychroflexus;s
SRR9330153MAG030	Yes	74.35	4.19	5478189	1157	5812	61.5	90.87	5080	d	Bacteria;p	Myxococcota;c Bradymonadia;o Bradymonadales;f Bradymonadaceae;g SLJM01;s
SRR9330153MAG034	No	71.8	2.05	2158409	842	2926	35.6	91.24	2550	d	Bacteria;p	Bacteroidota;c Bacteroidia;o Flavobacteriales;f Flavobacteriaceae;g Psychroflexus;s Psychroflexus sp003565575
SRR9330153MAG035	Yes	76	6.24	3075620	557	6826	44.4	86.13	2890	d	Bacteria;p	Bacteroidota;c Bacteroidia;o Chitinophagales;f Saprospiraceae;g PWJY01;s
SRR9330153MAG044	Yes	72.09	1.71	2336825	336	8702	59.1	92.7	2345	d	Bacteria;p	Proteobacteria;c Gammaproteobacteria;o Pseudomonadales;f Pseudohongiellaceae;g PWQG01;s
SRR9330153MAG048	No	88.8	5.74	3617673	211	41757	44.9	86.5	3107	d	Bacteria;p	Bacteroidota;c Rhodothermia;o Balneolales;f Natronogracilivirgulaceae;g ;s
SRR9330154MAG001	No	96.45	1.37	3256778	100	57616	50.1	89.68	2746	d	Bacteria;p	Bacteroidota;c Rhodothermia;o Balneolales;f Natronogracilivirgulaceae;g Natronogracilivirgula;s Natronogracilivirgula saccharolytica
SRR9330154MAG002	Yes	99.45	0.55	3072448	120	48418	40.2	85.48	2519	d	Bacteria;p	Bacteroidota;c Bacteroidia;o CAILMK01;f JAAYUY01;g ;s
SRR9330154MAG003	No	93.15	3.75	3011791	314	15148	62.5	92.36	3011	d	Bacteria;p	Proteobacteria;c Gammaproteobacteria;o Nitrococcales;f Aquisalimonadaceae;g ;s
SRR9330154MAG004	Yes	97.54	0.82	3390707	117	80724	52.5	87.61	2771	d	Bacteria;p	Bacteroidota;c Rhodothermia;o Balneolales;f PXAI01;g ;s
SRR9330154MAG005	Yes	95.63	1.14	2789527	238	18043	74.9	90	2630	d	Bacteria;p	Actinobacteriota;c Actinomycetia;o Euzebyales;f Egibacteraceae;g PUKE01;s PUKE01 sp003550425
SRR9330154MAG006	No	95.2	0.85	3491111	170	36715	62.5	87.76	3076	d	Bacteria;p	Bacteroidota;c Rhodothermia;o Rhodothermales;f Salinibacteraceae;g Tc-Br11-B2g6-7;s Tc-Br11-B2g6-7 sp001564055
SRR9330154MAG007	Yes	79.44	0.93	947963	56	44242	34.3	90.55	1054	d	Archaea;p	Nanoarchaeota;c Nanoarchaeia;o Woesearchaeales;f 21-14-0-10-32-9;g SKIS01;s
SRR9330154MAG008	No	92.24	3.23	1913037	192	16552	64.5	95.32	1982	d	Bacteria;p	Proteobacteria;c Gammaproteobacteria;o Nitrococcales;f Nitrococcaceae;g Spiribacter;s Spiribacter sp009676705
SRR9330154MAG009	Yes	98.36	6.55	3505799	320	20567	65.7	90.7	3537	d	Bacteria;p	Proteobacteria;c Gammaproteobacteria;o Nitrococcales;f Aquisalimonadaceae;g Aquisalimonas;s Aquisalimonas sp012044895
SRR9330154MAG010	Yes	79.91	0	1084662	20	217560	35.5	91.59	1234	d	Archaea;p	Nanoarchaeota;c Nanoarchaeia;o Woesearchaeales;f 21-14-0-10-32-9;g ;s
SRR9330154MAG011	Yes	98.11	4	3646354	212	31630	57.1	91.78	3338	d	Bacteria;p	Spirochaetota;c Spirochaetia;o DSM-27196;f SKKC01;g SLAN01;s
SRR9330154MAG012	Yes	94.87	3.51	2495104	143	43739	71.2	90.06	2385	d	Bacteria;p	Actinobacteriota;c Actinomycetia;o Euzebyales;f Egibacteraceae;g T1Sed10-49;s
SRR9330154MAG013	No	91.86	2.03	3688256	473	10767	57.3	85.1	3436	d	Bacteria;p	Bacteroidota;c Rhodothermia;o Rhodothermales;f Salinibacteraceae;g Longimonas;s
SRR9330154MAG014	No	89.65	4.92	4060490	790	6126	46	85.91	4005	d	Bacteria;p	Bacteroidota;c Rhodothermia;o Balneolales;f Balneolaceae;g SW132;s

SRR9330154MAG015	No	96.27	2.2	3005274	235	20678	51.6	89.53	3267	d	Archaea;p	Halobacteriota;c	Halobacteria;o	Halobacteriales;f	Haloferacaceae;g	Halalkalirubrum;s	Halalkalirubrum sp003551725
SRR9330154MAG016	No	76.64	0	756120	13	71690	39.8	93.6	824	d	Archaea;p	Nanoarchaeota;c	Nanoarchaeia;o	Woesearchaeales;f	21-14-0-10-32-9;g	;s	
SRR9330154MAG020	Yes	86.23	2.67	2487347	143	26636	67.6	88.43	2525	d	Archaea;p	Halobacteriota;c	Halobacteria;o	Halobacteriales;f	Haloferacaceae;g	Halorubrum;s	
SRR9330154MAG021	No	78.83	4.34	2571698	754	3936	64.1	88.9	3236	d	Archaea;p	Halobacteriota;c	Halobacteria;o	Halobacteriales;f	Haloarculaceae;g	Natronomonas;s	
SRR9330154MAG023	No	77.35	3.74	1030535	243	5055	39.9	91.27	1463	d	Archaea;p	Nanohaloarchaeota;c	Nanosalinia;o	Nanosalinales;f	Nanosalinaceae;g	B1-Br10-U2g21;s	B1-Br10-U2g21 sp001564145
SRR9330155MAG001	Yes	99.25	1.32	4072202	162	49902	40	89.72	3645	d	Bacteria;p	Bacteroidota;c	Bacteroidia;o	Cytophagales;f	Cyclobacteriaceae;g	Cecembia;s	Cecembia sp007126775
SRR9330155MAG002	No	96.7	2.73	4064390	236	35761	43.9	91.43	3757	d	Bacteria;p	Bacteroidota;c	T3Sed10-11;o	T3Sed10-11;f	T3Sed10-11;g	T3Sed10-11;s	T3Sed10-11 sp003568415
SRR9330155MAG003	Yes	98.74	0.91	2080551	110	33914	46.2	92.98	2036	d	Bacteria;p	Proteobacteria;c	Gammaproteobacteria;o	Thiomicrospirales;f	Thiomicrospiraceae;g	Thiomicrospira;s	
SRR9330155MAG004	Yes	95.06	1.09	4638251	332	29342	46.8	91.36	4143	d	Bacteria;p	Bacteroidota;c	T3Sed10-11;o	T3Sed10-11;f	T3Sed10-11;g	T3Sed10-11;s	
SRR9330155MAG005	No	99.46	3.76	3047983	233	21268	40.3	90.57	2722	d	Bacteria;p	Bacteroidota;c	Bacteroidia;o	Flavobacteriales;f	;g	;s	
SRR9330155MAG006	No	92.27	1.39	3084142	230	19329	59.5	88.83	2763	d	Bacteria;p	Proteobacteria;c	Gammaproteobacteria;o	Chromatiales;f	Sedimenticolaceae;g	SLIM01;s	SLIM01 sp007135625
SRR9330155MAG007	Yes	92.67	3.4	3172882	278	16809	55.4	91.04	3220	d	Bacteria;p	Proteobacteria;c	Gammaproteobacteria;o	Competibacterales;f	Competibacteraceae;g	SKOM01;s	SKOM01 sp007120095
SRR9330155MAG008	Yes	98.39	0.65	4214022	319	28984	57.8	89.52	4117	d	Bacteria;p	Desulfobacterota;c	Desulfuromonadia;o	Desulfuromonadales;f	Geoalkalibacteraceae;g	Geoalkalibacter;s	Geoalkalibacter ferrihydriticus
SRR9330155MAG009	Yes	86.23	3.18	2369078	242	14195	47.6	90.72	2353	d	Bacteria;p	Firmicutes D;c	Dethiobacteria;o	DTU022;f	DTU022;g	JAABTH01;s	
SRR9330155MAG010	No	97.61	5.87	2876965	189	24591	65.6	92.91	2748	d	Bacteria;p	Proteobacteria;c	Gammaproteobacteria;o	Xanthomonadales;f	Wenzhouxiangellaceae;g	Wenzhouxiangella;s	Wenzhouxiangella sp003560975
SRR9330155MAG011	Yes	96.13	3.87	2792206	185	22167	52.6	90.57	2703	d	Bacteria;p	Desulfobacterota;c	Desulfuromonadia;o	Desulfuromonadales;f	Geopsychrobacteraceae;g	Pelovirga;s	
SRR9330155MAG012	Yes	92.33	2.65	4506692	402	22100	60.5	88.49	4342	d	Bacteria;p	J088;c	J088;o	;f	;g	;s	
SRR9330155MAG013	Yes	99.18	0.57	2376101	107	32941	70.1	92.13	2597	d	Bacteria;p	Cyanobacteria;c	Cyanobacteriia;o	PCC-6307;f	Cyanobiaceae;g	NIES-981;s	
SRR9330155MAG014	Yes	97.36	2.35	3458591	271	27882	63.4	88.77	3367	d	Bacteria;p	Proteobacteria;c	Gammaproteobacteria;o	Ectothiorhodospirales;f	Thioalkalivibrionaceae;g	Thioalkalivibrio B;s	
SRR9330155MAG015	No	97.33	0	1367277	164	11954	44	93.92	1488	d	Bacteria;p	Firmicutes;c	Bacilli;o	Izemoplasmatales;f	Izemoplasmataceae;g	CSBR16-87;s	
SRR9330155MAG016	No	93.47	4.43	2925346	283	21804	43.9	90.02	3008	d	Bacteria;p	Firmicutes A;c	Clostridia;o	Peptostreptococcales;f	T1SED10-28;g	T1SED10-28;s	T1SED10-28 sp003554105
SRR9330155MAG017	Yes	94.31	1.71	2853225	281	21416	65.4	93.44	2814	d	Bacteria;p	Proteobacteria;c	Gammaproteobacteria;o	XN24;f	XN24;g	XN24;s	
SRR9330155MAG018	No	93.78	4.26	3153362	460	13239	60	88.05	3165	d	Bacteria;p	Proteobacteria;c	Gammaproteobacteria;o	Thiohalomonadales;f	Thiohalomonadaceae;g	SLDE01;s	SLDE01 sp007125455
SRR9330155MAG019	No	93.92	7.09	2174588	278	11036	44.2	86.95	2290	d	Bacteria;p	Firmicutes D;c	Dethiobacteria;o	SKNC01;f	SKNC01;g	SKNC01;s	SKNC01 sp003560915
SRR9330155MAG020	Yes	80.98	3.74	1029610	77	20023	32.7	90.58	1168	d	Archaea;p	Nanoarchaeota;c	Nanoarchaeia;o	Woesearchaeales;f	21-14-0-10-32-9;g	;s	
SRR9330155MAG021	Yes	85.74	3.09	2123552	170	22667	61.7	90.54	2098	d	Bacteria;p	Proteobacteria;c	Gammaproteobacteria;o	UBA5335;f	UBA5335;g	SLRW01;s	SLRW01 sp003567955
SRR9330155MAG024	Yes	90.56	1.13	3691103	400	11946	40.8	90.41	3505	d	Bacteria;p	Bacteroidota;c	Bacteroidia;o	Cytophagales;f	Cyclobacteriaceae;g	Cecembia;s	Cecembia lonarensis
SRR9330155MAG025	Yes	95.61	5.4	3458814	555	11423	51.1	91.69	3646	d	Bacteria;p	Proteobacteria;c	Gammaproteobacteria;o	Pseudomonadales;f	Nitrincolaceae;g	Nitrincola;s	
SRR9330155MAG026	Yes	93.64	8.18	4887694	338	25132	55	88.69	4189	d	Bacteria;p	Chloroflexota;c	Anaerolineae;o	Promineofilales;f	Promineofilaceae;g	SLGK01;s	
SRR9330155MAG027	Yes	91.78	5.79	3343832	517	8447	45.4	91.71	3583	d	Bacteria;p	Proteobacteria;c	Gammaproteobacteria;o	Pseudomonadales;f	Nitrincolaceae;g	Nitrincola;s	Nitrincola schmidtii
SRR9330155MAG028	Yes	94.76	1.99	4773495	857	7477	64.1	88.91	4626	d	Bacteria;p	Desulfobacterota;c	Desulfobacteria;o	Desulfobacterales;f	Desulfococcaceae;g	;s	
SRR9330155MAG030	No	75.37	4.84	1080721	476	2485	55.7	93.18	1488	d	Archaea;p	Thermoplasmatota;c	Thermoplasmata;o	Methanomassiliicoccales;f	Methanomethylophilaceae;g	PWHV01;s	PWHV01 sp003557905
SRR9330155MAG031	Yes	82.9	3.23	2802162	395	9628	52.3	89.03	2920	d	Bacteria;p	Desulfobacterota;c	Desulfuromonadia;o	Desulfuromonadales;f	Geopsychrobacteraceae;g	Pelovirga;s	
SRR9330155MAG033	No	97.97	4.17	4420126	361	24007	55.2	90.51	4259	d	Bacteria;p	Proteobacteria;c	Gammaproteobacteria;o	Pseudomonadales;f	Natronospirillaceae;g	;s	
SRR9330155MAG034	No	90.47	5.23	3694065	640	7801	64.2	85.88	3890	d	Bacteria;p	Deinococcota;c	Deinococci;o	Deinococcales;f	Trueperaceae;g	;s	
SRR9330155MAG035	No	92.88	8.85	4024314	390	15777	70.9	87.86	4003	d	Bacteria;p	Actinobacteriota;c	Actinomycetia;o	Nitriliruptorales;f	Nitriliruptoraceae;g	CSSed11-175R1;s	
SRR9330155MAG036	Yes	88.03	3.99	2395218	171	26412	67.8	94.23	2356	d	Bacteria;p	Proteobacteria;c	Gammaproteobacteria;o	XN24;f	XN24;g	XN24;s	
SRR9330155MAG037	No	86.43	4.76	3746564	592	8174	58.5	87.18	3757	d	Bacteria;p	Desulfobacterota I;c	Desulfovibrionia;o	Desulfovibrionales;f	Desulfonatronaceae;g	Desulfonatronum;s	Desulfonatronum sp007127655
SRR9330155MAG038	No	85.37	2.53	5368871	691	9999	65.2	89.3	4342	d	Bacteria;p	Planctomycetota;c	Planctomycetia;o	Pirellulales;f	Pirellulaceae;g	;s	
SRR9330155MAG039	No	83.79	4.22	4287866	706	7945	50.5	89.44	4117	d	Bacteria;p	Chloroflexota;c	Anaerolineae;o	Promineofilales;f	Promineofilaceae;g	SLGK01;s	
SRR9330155MAG040	Yes	93.16	0.62	2782866	398	9568	55.9	90.75	2897	d	Bacteria;p	Proteobacteria;c	Gammaproteobacteria;o	Pseudomonadales;f	Alcanivoracaceae;g	Alcanivorax;s	
SRR9330155MAG042	Yes	91.86	5.99	3082070	137	40809	64.8	90.65	3033	d	Bacteria;p	Proteobacteria;c	Alphaproteobacteria;o	Rhodobacterales;f	Rhodobacteraceae;g	Roseinatronobacter;s	Roseinatronobacter sp007134805
SRR9330155MAG043	Yes	85.08	6.92	2439594	313	10701	59.2	90.06	2488	d	Bacteria;p	Proteobacteria;c	Gammaproteobacteria;o	Thiohalomonadales;f	Thiohalomonadaceae;g	SLDE01;s	
SRR9330155MAG044	No	70.31	4.06	2488515	893	3097	39.7	88.59	2567	d	Bacteria;p	Bacteroidota;c	Bacteroidia;o	Chitinophagales;f	Saprospiraceae;g	PWJY01;s	
SRR9330155MAG047	Yes	76.4	2.01	2092900	622	4147	44.4	89.67	2216	d	Bacteria;p	Firmicutes D;c	Dethiobacteria;o	DTU022;f	PWGO01;g	;s	
SRR9330155MAG049	No	92.46	4.42	3368511	312	17317	68	89.46	3300	d	Bacteria;p	Proteobacteria;c	Alphaproteobacteria;o	Rhodobacterales;f	Rhodobacteraceae;g	PUOA01;s	PUOA01 sp007121115
SRR9330155MAG051	No	74.46	6.33	4190794	1872	2490	44.7	90.55	5074	d	Bacteria;p	Bacteroidota;c	T3Sed10-11;o	T3Sed10-11;f	T3Sed10-11;g	T3Sed10-11;s	
SRR9330155MAG054	Yes	95.56	4	1554923	208	11847	36.3	92.65	1656	d	Bacteria;p	Firmicutes;c	Bacilli;o	Izemoplasmatales;f	Izemoplasmataceae;g	CSBR16-104;s	
SRR9330155MAG055	Yes	94.83	3.92	3251738	279	18612	46.8	90.04	2743	d	Bacteria;p	Bacteroidota;c	Bacteroidia;o	Bacteroidales;f	UBA7960;g	SKTA01;s	SKTA01 sp007120085
SRR9330155MAG060	No	94.83	6.66	3819727	396	24311	34.8	88.52	3760	d	Bacteria;p	Bacteroidota;c	Bacteroidia;o	Flavobacteriales;f	Crocinitomicaceae;g	;s	
SRR9330155MAG062	Yes	89.25	6.1	2475379	193	21904	57.5	91.34	2444	d	Bacteria;p	Proteobacteria;c	Gammaproteobacteria;o	Thiohalomonadales;f	Thiohalomonadaceae;g	SLDE01;s	
SRR9330155MAG064	Yes	92.74	3.77	2760669	413	9758	51.1	91.89	2841	d	Bacteria;p	Proteobacteria;c	Gammaproteobacteria;o	Pseudomonadales;f	DT-91;g	DT-91;s	
SRR9330155MAG066	Yes	93.14	7.97	3131196	330	15629	47.2	92.77	2752	d	Bacteria;p	Bacteroidota;c	Bacteroidia;o	Bacteroidales;f	UBA7960;g	SKRM01;s	
SRR9330155MAG075	No	75.7	2.11	2349204	742	3638	48.1	89.18	2437	d	Bacteria;p	Bacteroidota;c	Rhodothermia;o	Balneolales;f	PXAI01;g	Cyclonatronum;s	Cyclonatronum sp003555145
SRR9330155MAG077	Yes	76.38	2.7	2807861	567	6360	60	91.07	3200	d	Bacteria;p	Proteobacteria;c	Alphaproteobacteria;o	Rhodobacterales;f	Rhodobacteraceae;g	Roseinatronobacter;s	
SRR9330155MAG078	Yes	78.68	6.63	3052008	812	4385	69.1	89.11	3318	d	Bacteria;p	Proteobacteria;c	Alphaproteobacteria;o	Rhodobacterales;f	Rhodobacteraceae;g	Pararhodobacter;s	
SRR9330155MAG081	Yes	87.7	0.2	3120512	121	45579	59.7	91.19	3055	d	Bacteria;p	Proteobacteria;c	Alphaproteobacteria;o	Rhodobacterales;f	Rhodobacteraceae;g	Roseinatronobacter;s	Roseinatronobacter sp007128995
SRR9330155MAG111	Yes	79.23	5.01	3339202	946	4956	72	92.78	3853	d	Bacteria;p	Deinococcota;c	Deinococci;o	Deinococcales;f	Trueperaceae;g	CSSed10-48;s	
SRR9330155MAG115	Yes	85.92	5.66	2356781	542	5333	65.5	90.22	2619	d	Bacteria;p	Proteobacteria;c	Gammaproteobacteria;o	DSM-19610;f	DSM-19610;g	;s	
SRR9330156MAG001	No	87.97	1.97	2676495	145	34536	62.3	89.66	2882	d	Archaea;p	Halobacteriota;c	Halobacteria;o	Halobacteriales;f	Natronoarchaeaceae;g	Natronoarchaeum;s	
SRR9330156MAG002	Yes	97.46	0.56	3683572	153	37568	62.3	87.32	3264	d	Bacteria;p	Bacteroidota;c	Rhodothermia;o	Rhodothermales;f	Salinibacteraceae;g	Tc-Br11-B2g6-7;s	Tc-Br11-B2g6-7 sp001564055
SRR9330156MAG003	No	85.3	0.93	953623	28	76278	40.7	91.55	1111	d	Archaea;p	Nanohaloarchaeota;c	Nanosalinia;o	Nanosalinales;f	Nanosalinaceae;g	B1-Br10-U2g19;s	B1-Br10-U2g19 sp001563905
SRR9330156MAG004	No	82.43	2.85	2410582	182	19210	63.3	88.45	2554	d	Archaea;p	Halobacteriota;c	Halobacteria;o	Halobacteriales;f	Haloferacaceae;g	Halorubrum;s	
SRR9330156MAG005	No	89.28	4.47	2602306	214	18489	47.1	84.61	2640	d	Archaea;p	Halobacteriota;c	Halobacteria;o	Halobacteriales;f	Salinarchaeaceae;g	Salinarchaeum;s	
SRR9330156MAG006	Yes	95.25	3.36	2586894	95	47873	64.5	88.79	2758	d	Archaea;p	Halobacteriota;c	Halobacteria;o	Halobacteriales;f	Haloarculaceae;g	Natronomonas;s	
SRR9330156MAG010	No	90.26	7.24	2068267	130	26164	62	88.09	2237	d	Archaea;p	Halobacteriota;c	Halobacteria;o	Halobacteriales;f	Haloferacaceae;g	PL-Br10-E2g29;s	
SRR9330156MAG013	No	74.02	2.16	1420941	660	2343	64.9	95.39	1890	d	Bacteria;p	Proteobacteria;c	Gammaproteobacteria;o	Nitrococcales;f	Nitrococcaceae;g	Spiribacter;s	Spiribacter sp009676705
SRR9330156MAG014	No	79.09	1.2	1697017	88	37616	62	88.75	1824	d	Archaea;p	Halobacteriota;c	Halobacteria;o	Halobacteriales;f	Haloferacaceae;g	PL-Br10-E2g29;s	PL-Br10-E2g29 sp001563965
SRR9330156MAG015	No	85.12	2.7	2454045	268	13473	65.1	88.07	2731	d	Archaea;p	Halobacteriota;c	Halobacteria;o	Halobacteriales;f	Haloarculaceae;g	Natronomonas;s	
SRR9330156MAG017	No	75.4	5.75	2142942	523	4809	66.8	89.66	2599	d	Archaea;p	Halobacteriota;c	Halobacteria;o	Halobacteriales;f	Haloferacaceae;g	Halorubrum;s	Halorubrum sp003554605
SRR9330156MAG019	No	70.46	0.42	1840666	149	17869	66.2	87.73	1929	d	Archaea;p	Halobacteriota;c	Halobacteria;o	Halobacteriales;f	Haloferacaceae;g	Halorubrum;s	
