# Supplementary material for: Biogeography of soda lake microbiome and uneven cross-continent transition rates
Source: Front Microbiol. 2025 Jul 24;16:1614302. doi: 10.3389/fmicb.2025.1614302 (PMC12330390; doi:10.3389/fmicb.2025.1614302)
Supplement: Supplementary file 2 [file Supplementary_file_2.docx]

Biogeography of soda lake microbiome and uneven cross-continent transition rates

Minglei Ren^1^, Jianjun Wang^1, #^

^1^State Key Laboratory of Lake and Watershed Science for Water Security, Nanjing Institute of Geography and Limnology, Chinese Academy of Sciences, Nanjing 211135, China.

^#^ Correspondence: [jjwang@niglas.ac.cn](mailto:jjwang@niglas.ac.cn)

Contents

Supplementary figure S1-S2.


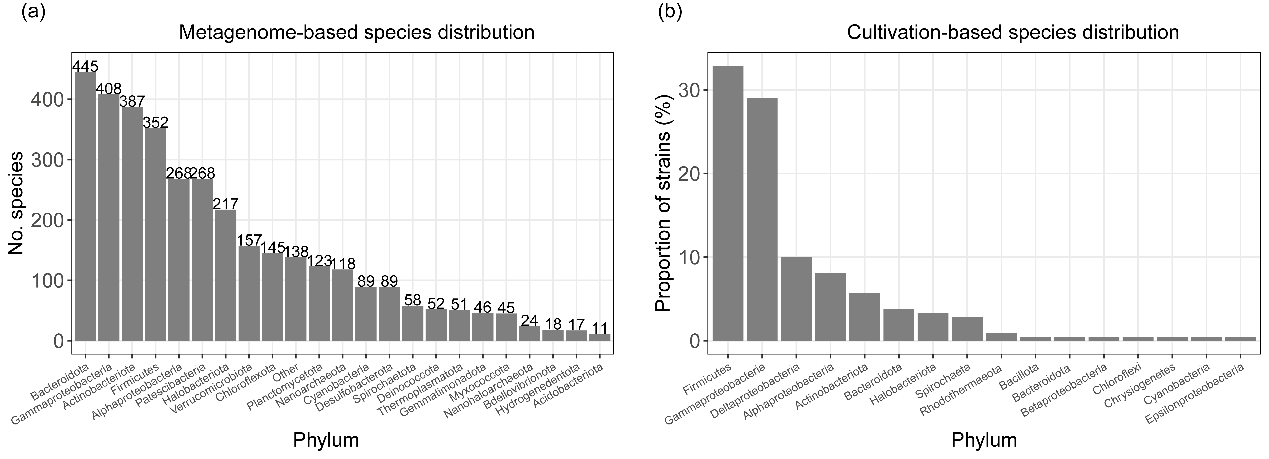


**Figure S1. The distribution of metagenome-based and cultivation-based species in each phylum across global soda lakes.** The metagenome-based species were identified using the *rpS3*-based taxonomy profiling across soda lakes samples (See the method), whereas the cultivation-based alkaliphilic strains was retrieved and compiled based on literature search in this study and previous studies (Table S2). The literatures were collected in the NCBI PubMed database, especially focusing the classical microbiology journal “International Journal of Systematic and Evolutionary Microbiology” with the keywords “soda lake”, “alkaline lake”, “alkaliphilic”. The phyla in (a) were ordered by the number of species they have, with the ones less than 10 species assigned as the group ‘Other’.


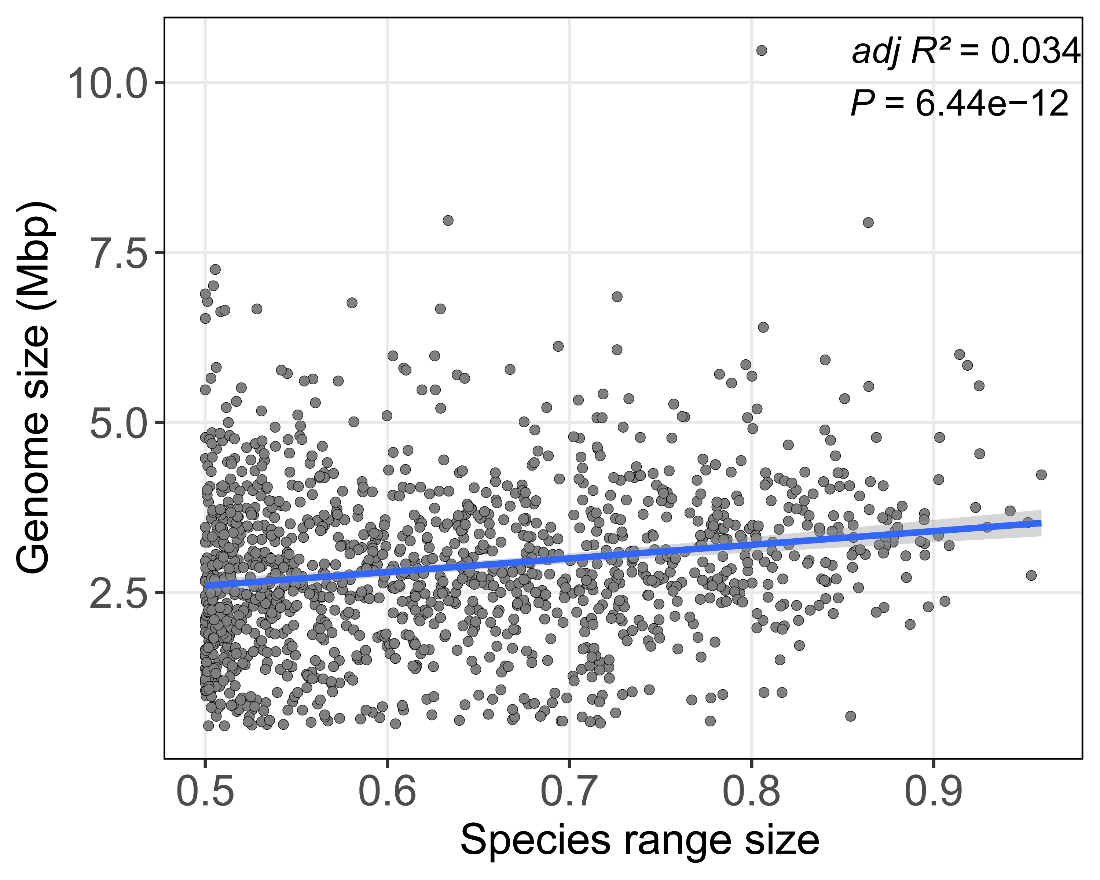


**Figure S2. Microbial genome size increased with their geographic range.** The linear relationships between genomic size and species range size across all representative species in global soda lakes. The adjusted coefficients and significance results of regression models were appended.
